# 1-Imidoalkylphosphonium salts with modulated C_α_–P^+^ bond strength: synthesis and application as new active α-imidoalkylating agents

**DOI:** 10.3762/bjoc.13.142

**Published:** 2017-07-24

**Authors:** Jakub Adamek, Roman Mazurkiewicz, Anna Węgrzyk, Karol Erfurt

**Affiliations:** 1 Department of Organic Chemistry, Bioorganic Chemistry and Biotechnology, Silesian University of Technology, B. Krzywoustego 4, 44-100 Gliwice, Poland; 2 Biotechnology Centre of Silesian University of Technology, B. Krzywoustego 8, 44-100 Gliwice, Poland; 3 Department of Chemical Organic Technology and Petrochemistry, Silesian University of Technology, B. Krzywoustego 4, 44-100 Gliwice, Poland

**Keywords:** *N*-(1-arylalkyl)imides, α-imidoalkylating agents, imidoalkylation reactions, 1-imidoalkylphosphonium salts, Tscherniac–Einhorn-type reaction

## Abstract

An effective synthesis of the hitherto unknown 1-imidoalkylphosphonium salts has been developed in the reported study. The crucial step in the method included the decarboxylative α-methoxylation of *N*-phthaloyl- or *N*-succinylamino acids to the corresponding *N*-(1-methoxyalkyl)imides, followed by the displacement of the methoxy group by the triarylphosphonium group through melting of the imide derivative with triarylphosphonium tetrafluoroborate. The imidoalkylating properties of the obtained 1-imidoalkylphosphonium salts were tested using the Tscherniac–Einhorn-type reaction with aromatic hydrocarbons as a model reaction. It was found that the C_α_–P^+^ bond strength can be considerably reduced and the imidoalkylation of arenes can be markedly facilitated using 1-imidoalkylphosphonium salts derived from triarylphosphines with electron-withdrawing substituents such as tris(*m*-chorophenyl)phosphine, tris(*p*-chlorophenyl)phosphine and tris[*p*-(trifluoromethyl)phenyl]phosphine. Microwave irradiation also considerably facilitates the cleavage of the highly polar C_α_–P^+^ bond.

## Introduction

**Scheme 1:**
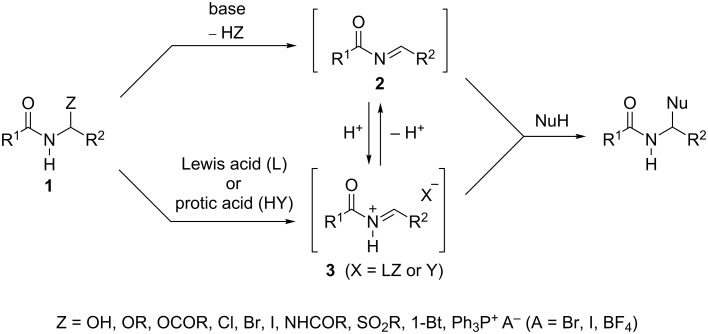
α-Amidoalkylation reactions under basic or acidic conditions.

The aminomethylation of C–H acidic compounds by the condensation of non-enolizable aldehydes or ketones (mainly formaldehyde) with ammonia or aliphatic amines and CH-acidic carbonyl compounds, known as the Mannich reaction, plays an important role in organic synthesis, despite some limitations of this reaction. α-Amidoalkylation reactions are considered an important extension of the Mannich reaction [1–3]. They are crucial methods for the formation of >NC–C and >NC–Het bonds used, inter alia, for the generation of the β-aminocarbonyl substructure and for the construction of new carbo- or heterocyclic systems, especially in pharmaceutical chemistry and in the synthesis of natural products [3–12]. Most of the α-amidoalkylating reagents possess the structure of α-functionalized *N*-alkylamides **1**, where Z = OH, OR, OCOR, Cl, Br, I, NHCOR, SO_2_Ar, 1-benzotriazolyl or PPh_3_^+^ X^−^ and acts as a nucleofugal leaving group (Scheme 1) [3,8–10,13–18]. *N*-Acylimine **2** or the much more reactive *N*-acyliminium cation **3** are active reagents in α-amidoalkylation reactions, usually generated in situ from a precursor of structure **1** under basic or acidic conditions, respectively (Scheme 1) [3].

The limitations and disadvantages of the most commonly used amidoalkylating agents have been comprehensively discussed in the literature [3,8]. Many of these (Z = OR, OCOR, Cl, Br, I) are quite unstable compounds that cannot be stored for a prolonged time and thus have to be prepared in situ. Further, the preparation of some of these reagents (Z = Cl, Br, I) is often difficult and elaborate. Most of these, including the most frequently used α-alkoxy derivatives (Z = OR), *N*-[1-(benzotriazol-1-yl)alkyl]amides (Z = 1-benzotriazolyl) and 1-(*N*-acylamino)alkyl sulfones (Z = SO_2_Ar) require activation with Lewis acids, which are expensive (e.g., ZrCl_4_, VCl_3_, CeCl_3_, Bi(OTf)_3_ or InCl_3_) [11,17,19]. Moreover, the application of Lewis acids can diminish the activity of a reacting nucleophile, and usually requires a labor-intensive aqueous work-up procedure of the reaction mixture. The inevitable equilibrium between the *N*-acyliminium cation **3** and the less reactive uncharged *N*-acylimine **2** also reduces the general reactivity of this reaction system.

Recently, we have described a simple and efficient two-step transformation of *N*-acylated α-amino acids to 1-(*N*-acylamino)alkyltriphenylphosphonium salts **1** (Z **=** PPh_3_^+^ A^−^) [18]. We have also demonstrated that the obtained stable, crystalline phosphonium salts are new, powerful, and easy to use α-amidoalkylating agents, which are active either without the need for a catalyst or in the presence of organic bases (e.g., Hünig’s base) [20–23].

**Scheme 2:**
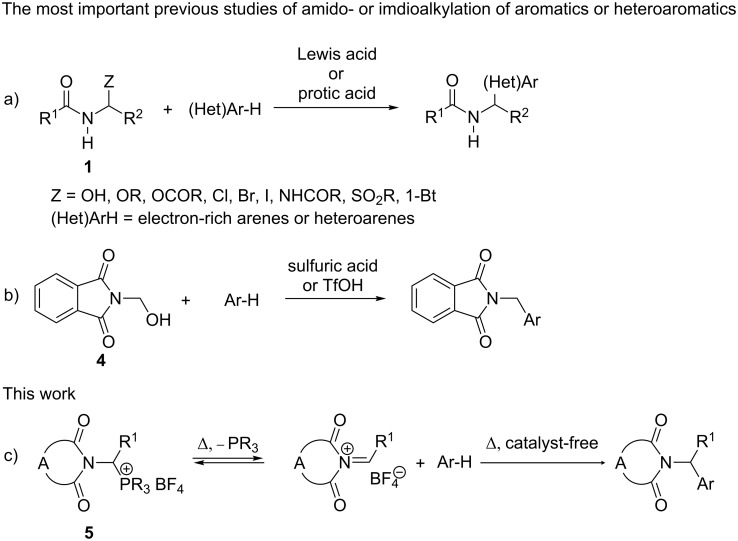
Synthetic routes of α-amido- and α-imidoalkylation of aromatic and heteroaromatic compounds.

It is a well-known problem, that the reactivity of α-amidoalkylating agents toward nucleophiles of low reactivity (e.g., aromatic systems without strong electron-donating substituents) is insufficient. This limits the scope of α-amidoalkylation reactions of aromatic systems (including important intramolecular α-amidoalkylations with the formation of new carbo- or heterocyclic products) to aromatic systems with strong electron-donating substituents (e.g., alkoxy-, polyalkoxy- and aminoarenes), or some activated heterocyclic systems such as indole [3,8,10,24–25]. The α-imidoalkylation reaction using *N*-(1-hydroxyalkyl)imides (almost entirely *N*-hydroxymethylphthalimide) in sulfuric acid, oleum or other strong acids is considered an alternative to α-amidoalkylation (Scheme 2) [3–6,24]. Olah et al. successfully applied *N*-hydroxymethylphthalimide (**4**) in the imidomethylation of benzene, halo-, polyhalo- and halonitrobenzenes using superacidic trifluoromethanesulfonic acid as the solvent [24]. According to the authors, the enhanced reactivity of *N*-hydroxymethylphthalimide (**4**) under these conditions is probably caused by the protonation of the intermediate imidomethylcarbenium ion on the nitrogen or carbonyl oxygen (protosolvation) to the corresponding superelectrophilic dication [24]. To the best of our knowledge, Olah’s imidomethylation in the presence of trifluoromethanesulfonic acid has not been extended to other *N*-(1-hydroxyalkyl)imides. Moreover, the employment of a large amount of superacidic trifluoromethanesulfonic acid as a solvent is expensive and complicates the work-up procedure. Obviously, Olah’s imidoalkylating procedure and other similar imidoalkylating systems using strong acids as catalysts and solvents cannot be applied to aromatics or heteroaromatics of basic character, such as tertiary aromatic amines or azines.

In the present paper we report the synthesis and properties of hitherto unknown 1-imidoalkylphosphonium salts **5** (Scheme 2). We expected that the 1-imidoalkylcarbenium cation generated from these salts (see Scheme 2) would display greater electrophilic reactivity in comparison with *N*-acyliminium cation **3**, due to the strong electron-withdrawing effect of the two carbonyl groups attached to the nitrogen atom. Furthermore, the tertiary nitrogen atom of the 1-imidocarbenium cation cannot be deprotonated to the less reactive uncharged form.

Subsequently we demonstrate the strong imidoalkylating properties of the obtained 1-imidoalkylphosphonium salts **5** applied in the Tscherniac–Einhorn-type reaction with aromatic hydrocarbons as a model reaction. The obtained *N*-(1-arylalkyl)imides can easily be transformed to the corresponding primary 1-arylalkylamines, following the well-known procedures developed for the Gabriel synthesis of primary amines. Moreover, this class of compounds is itself attracting significant attention from chemists and biochemists due to their bioactivity as herbicides, and anticancer, anti-inflammatory, analgesic or anticonvulsant agents [26–31]. Especially interesting is apremilast (brand name Otezla), a tumor necrosis factor-α (TNF-α) inhibitor used for the treatment of psoriasis, psoriatic dermatitis and other inflammatory diseases related to the immune system [31].

## Results and Discussion

1-Imidoalkylphosphonium salts **5** were synthesized in a three-step procedure starting from α-amino acids and cyclic dicarboxylic acid anhydrides. 2-(*N*-Imido)alkanecarboxylic acids **6** were obtained in good to very good yields by melting phthalic or succinic acid anhydride with the corresponding amino acid at 140–170 °C, according to the McKenzie and Walker’s procedure (Table 1) [32]. To the best of our knowledge, attempts at an electrochemical decarboxylative α-methoxylation of 2-imidoalkanecarboxylic acids have been reported only twice in the literature [33–34]. The reactions were carried out in MeOH in the presence of sodium methoxide. Unfortunately, because of the low reaction selectivity related to side reactions (for example Kolbe-dimerization), the yields were only poor (10–35%) [33–34]. According to our previously reported procedure for the electrochemical decarboxylative α-methoxylation of *N*-acyl-α-amino acids [18], amino acid derivatives **6** were converted to *N*-(1-methoxyalkyl)imides **7**. The reaction was performed in an undivided cylindrical glass electrolyzer in MeOH at 0 °C in the presence of 3-(1-piperidino)propyl-functionalized silica gel (SiO_2_-Pip) as the base, at a constant current density, and at the charge consumption of 2.70–3.75 F/mol. Under these conditions the expected *N*-(1-methoxyalkyl)imides **7** were obtained in most cases in moderate to good yields (Table 1).

**Table 1:** Synthesis of 2-(*N*-imido)alkanecarboxylic acids **6** and *N*-(1-methoxy)alkylimides **7**.

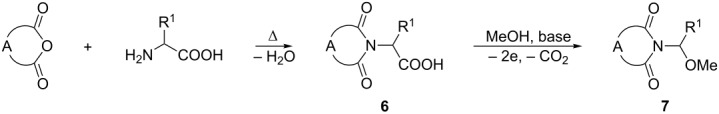								

A	R^1^	2-(*N*-imido)alkanecarboxylic acids **6**				*N*-(1-methoxyalkyl)imides **7**		
		
		time, h	*T*, °C	**6**	yield, %	charge, F/mol	**7**	yield, %

*o*-C_6_H_4_	H	1.0	170	**6a**	50	2.7	**7a**	28
*o*-C_6_H_4_	Me	2.5	170	**6b**	94	3.0	**7b**	65
*o*-C_6_H_4_	Bn	2.5	170	**6c**	93	2.9	**7c**	42
*o*-C_6_H_4_	Ph	2.5	170	**6d**	75	2.9	**7d**	60
*o*-C_6_H_4_	iBu	2.5	170	**6e**	90	2.9	**7e**	46
(CH_2_)_2_	H	6.5	140	**6f**	46	2.7	**7f**	33
(CH_2_)_2_	Me	6.5	140	**6g**	67	3.5	**7g**	85
1,8-C_10_H_6_	Me	5.0	120	**6h**	84^a^	3.75	**7h**	52

^a^2-(1,8-Naphthalimido)propionic acid (**6h**) was obtained according to Reger’s procedure [35].

The synthesis of 1-imidoalkyltriarylphosphonium tetrafluoroborates **5** represents a substitution of the methoxy group of *N*-(1-methoxyalkyl)imides **7** by the triarylphosphonium group. For this, a mixture of *N*-(1-methoxyalkyl)imide **7** with triarylphosphonium tetrafluoroborate was melted at 85–140 °C in the presence of NaBr as a catalyst under reduced pressure (0.1–0.2 mmHg) for 0.5–10 h (Table 2).

**Table 2:** Synthesis of 1-imidoalkyltriarylphosphonium tetrafluoroborates **5**.

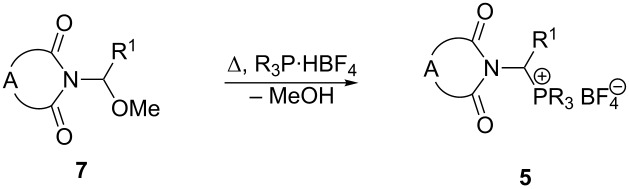						

A	R^1^	R	1-imidoalkyltriarylphosphonium salts **5**			
			
			time, h	*T*, °C	**5**	yield, %

*o*-C_6_H_4_	H	Ph	6	140	**5a**	49
*o*-C_6_H_4_	Me	Ph	7	90	**5b**	92
*o*-C_6_H_4_	Me	*m*-C_6_H_4_Cl	8	95	**5c**	83
*o*-C_6_H_4_	Me	*p*-C_6_H_4_Cl	6	95	**5d**	89
*o*-C_6_H_4_	Me	*p*-C_6_H_4_CF_3_	7	95	**5e**	95
*o*-C_6_H_4_	Ph	Ph	4	90	**5f**	94
*o*-C_6_H_4_	Ph	*m*-C_6_H_4_Cl	8	95	**5g**	88
*o*-C_6_H_4_	Ph	*p*-C_6_H_4_CF_3_	7	95	**5h**	80
*o*-C_6_H_4_	iBu	Ph	5.5	95	**5i**	79
*o*-C_6_H_4_	iBu	*m*-C_6_H_4_Cl	7	95	**5j**	75
(CH_2_)_2_	H	Ph	7	140	**5k**	65
(CH_2_)_2_	Me	Ph	10	85	**5l**	58
(CH_2_)_2_	Me	*m*-C_6_H_4_Cl	7	95	**5m**	28
(CH_2_)_2_	Me	*p*-C_6_H_4_CF_3_	0.5	120	**5n**	51
1,8-C_10_H_6_	Me	Ph	2	120	**5o**	<10^a^

^a^Yield estimated by ^1^H NMR. 1-Methoxyethyltriphenylphosphonium tetrafluoroborate (**8**) was isolated from the reaction mixture as the main product in 73% yield.

Phthalimide-derived phosphonium salts **5** (A = *o*-C_6_H_4_) were usually obtained in good to excellent yields, whereas the yields of succinimide-derived products **5** (A = (CH_2_)_2_) were generally lower. In the case of *N*-(1-methoxyethyl)-1,8-naphthalimide the triphenylphosphine attack on the α-carbon resulted in the splitting of the C_α_–N bond instead of the C_α_–OMe bond and the corresponding 1-methoxyethyltriphenylphosphonium tetrafluoroborate was isolated in 73% yield accompanied by only trace amounts of the expected phosphonium salt **5o** (Table 2, last entry). It is assumed that both the extraordinary effective resonance stabilization of the imide anion and the excessive steric congestion in the transition state make the splitting of the C_α_–N bond instead of the C_α_–OMe bond more favorable in this case.

The phosphonium salts **5** were purified by crystallization, usually from a mixture of CH_2_Cl_2_/Et_2_O. However, in a few cases preliminary purification by column chromatography proved to be necessary. Their structures were confirmed by ^1^H, ^13^C, ^31^P and ^19^F NMR spectroscopy, as well as IR spectroscopy and HRMS spectrometry.

Next the phosphonium salts were used in the Tscherniac–Einhorn–type imidoalkylation of aromatic hydrocarbons of diverse reactivity. The reaction was carried out without a catalyst, using a considerable excess of the reacting hydrocarbon as a solvent (the molar ratio of reactants was about 1:75 to 1:90 and depended on the molecular mass of hydrocarbon). In some cases, the additional application of unreactive nitrobenzene or chlorobenzene as cosolvents was found to be necessary to improve the solubility of the phosphonium salts (Table 3).

**Table 3:** Synthesis of *N*-(1-arylalkyl)imides **9**.

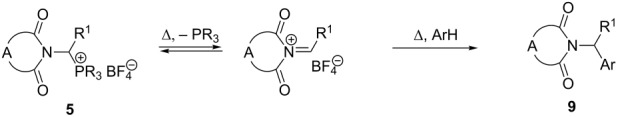													

entry	1-imidoalkyltriaryl- phosphonium salts				ArH	solvent	*T*, °C	time, h	MW	US^a^	**9**	yield, %	*o*:*p*^b^
										
	**5**	A	R^1^	R									

1	**5b**	*o*-C_6_H_4_	Me	Ph	anisole	**–**	180	2	**–**	**–**	**9aa**+**9ab**	72	1.0:1.2
2	**5b**	*o*-C_6_H_4_	Me	Ph		**–**	150	1	70 W^c^	**–**		66	1.0:1.1
3	**5c**	*o*-C_6_H_4_	Me	*m*-ClC_6_H_4_		**–**	100	3.5	**–**	**–**		77	1.0:1.2
4	**5c**	*o*-C_6_H_4_	Me	*m*-ClC_6_H_4_		**–**	75	1	10 W^c^	**–**		78	1.0:1.1
5	**5e**	*o*-C_6_H_4_	Me	*p*-CF_3_C_6_H_4_		**–**	90	2	**–**	**–**		91	1.0:1.1
6	**5e**	*o*-C_6_H_4_	Me	*p*-CF_3_C_6_H_4_		**–**	90	1	**–**	+		78	1.0:1.1
7	**5e**	*o*-C_6_H_4_	Me	*p*-CF_3_C_6_H_4_		**–**	60	4	6 W^c^	**–**		82	1.0:1.0
		
8	**5f**	*o*-C_6_H_4_	Ph	Ph		**–**	150	2.5	**–**	**–**	**9ba**+**9bb**	58	1.0:1.6
9	**5f**	*o*-C_6_H_4_	Ph	Ph		**–**	130	1	25 W^c^	**–**		60	1.0:2.8
10	**5h**	*o*-C_6_H_4_	Ph	*p*-CF_3_C_6_H_4_		**–**	90	2	**–**	**–**		81	1.0:2.3
11	**5h**	*o*-C_6_H_4_	Ph	*p*-CF_3_C_6_H_4_		**–**	90	1	**–**	+		83	1.0:1.9
		
12	**5i**	*o*-C_6_H_4_	iBu	Ph		**–**	170	2	**–**	**–**	**9ca**+**9cb**	58	1.0:3.0
13	**5j**	*o*-C_6_H_4_	iBu	*m*-ClC_6_H_4_		**–**	100	2	**–**	**–**		88	1.0:2.1
		
14	**5l**	(CH_2_)_2_	Me	Ph		**–**	195	2	**–**	**–**	**9da**+**9db**	41	1.4:1.0
15	**5n**	(CH_2_)_2_	Me	*p*-CF_3_C_6_H_4_		**–**	140	2	**–**	**–**		56	1.3:1.0

16	**5b**	*o*-C_6_H_4_	Me	Ph	1,3-dimethoxy- benzene	**–**	170	2.5	**–**	**–**	**9e**	71	**–**
17	**5c**	*o*-C_6_H_4_	Me	*m*-ClC_6_H_4_		C_6_H_5_NO_2_	100	4	**–**	**–**		73	**–**
18	**5e**	*o*-C_6_H_4_	Me	*p*-CF_3_C_6_H_4_		C_6_H_5_Cl	80	4	**–**	**–**		76	**–**
19	**5e**	*o*-C_6_H_4_	Me	*p*-CF_3_C_6_H_4_		C_6_H_5_Cl	80	3	**–**	+		82	**–**
		
20	**5f**	*o*-C_6_H_4_	Ph	Ph		C_6_H_5_NO_2_	150	0.5	**–**	**–**	**9f**	60	**–**
		
21	**5i**	*o*-C_6_H_4_	iBu	Ph		**–**	170	2	**–**	**–**	**9g**	76	**–**
22	**5j**	*o*-C_6_H_4_	iBu	*m*-ClC_6_H_4_			100	2	**–**	**–**		56	**–**
23	**5j**	*o*-C_6_H_4_	iBu	*m*-ClC_6_H_4_		C_6_H_5_Cl	100	2	**–**	**–**		63	**–**

24	**5e**	*o*-C_6_H_4_	Me	*p*-CF_3_C_6_H_4_	1,3,5-trimethoxy- benzene	**–**	110	2	**–**	**–**	**–**	–^d^	**–**

25	**5e**	*o*-C_6_H_4_	Me	*p*-CF_3_C_6_H_4_	toluene	**–**	130	2	**–**	**–**	**9ha**+**9hb**	23^e^	1.0:2.6
26	**5e**	*o*-C_6_H_4_	Me	*p*-CF_3_C_6_H_4_		**–**	120	0.25	**–**	**–**		50^f^	1.0:3.4
27	**5e**	*o*-C_6_H_4_	Me	*p*-CF_3_C_6_H_4_		**–**	100	0.25	100 W^c^	**–**		46^f^	1.0:4.0
		
28	**5h**	*o*-C_6_H_4_	Ph	*p*-CF_3_C_6_H_4_		C_6_H_5_NO_2_	130	2	**–**	**–**	**9ia**+**9ib**	51	1.0:2.3
29	**5h**	*o*-C_6_H_4_	Ph	*p*-CF_3_C_6_H_4_		C_6_H_5_NO_2_	120	2	140 W^c^	**–**		60	1.0:3.8

^a^The exposition to ultrasound irradiation. ^b^Molar ratio of *ortho* to *para* isomer. ^c^The average microwave power that provides the desired reaction temperature. ^d^1-(2,4,6-trimethoxyphenyl)ethyltris(4-trifluoromethylphenyl)phosphonium tetrafluoroborate (**10**) was isolated from the reaction mixture in 73% yield as the main product. ^e^*N*-Vinylphthalimide was also obtained as side product in 54% yield. ^f^The reaction was carried out in the presence of an equimolar amount of tetrafluoroboric acid.

The reaction of 1-phthalimidoethyltriphenylphosphonium tetrafluoroborate (**5b**) with anisole required a relatively high temperature (180 °C; Table 3, entry 1). The reaction temperature could be reduced considerably to 150 °C together with a reduced reaction time using microwave irradiation in a sealed glass vial (Table 3, entry 2). The reaction with the much more reactive 1,3-dimethoxybenzene required only a slightly lower temperature (170 °C; Table 3, entry 16). The reactivity of 1-phthalimido-3-methylbutyltriphenylphosphonium salt **5i** was similar to that displayed by 1-phthalimidoethyltriphenylphosphonium salt **5b** (cf Table 3, entries 1 against 12 and 16 against 21). As expected, the reaction of anisole with 1-phthalimido-1-phenylmethyltriphenylphosphonium salt **5f**, which generates a more stable benzyl-type carbenium cation, started at a lower temperature (150 °C; Table 3, entry 8). Under microwave conditions, the reaction had already started at about 130 °C (Table 3, entry 9). Again, the reaction of the same phosphonium salt with the much more reactive 1,3-dimethoxybenzene started at the same temperature (150 °C; Table 3, entry 20). This observation suggests that the generation of a 1-imidocarbenium cation via the thermal splitting of the C_α_–P^+^ bond (Scheme 2) is the rate-determining step in the Tscherniac–Einhorn–type imidoalkylation of the relatively reactive hydrogen carbons such as anisole or 1,3-dimethoxybenzene. Consequently, the higher reactivity of 1,3-dimethoxybenzene in relation to anisole does not exert an impact on the reaction rate. As expected, microwave irradiation effectively facilitates the splitting of the highly polar C_α_–P^+^ bond.

In order to reduce the C_α_–P^+^ bond strength, and to facilitate the generation of the corresponding 1-imidocarbenium cation, 1-imidoalkylphosphonium salts derived from triarylphosphines with electron-withdrawing substituents such as tris(*m*-chorophenyl)phosphine, tris(*p*-chlorophenyl)phosphine and tris[*p*-(trifluoromethyl)phenyl]phosphine were synthesized and tested in the reaction. As expected, the phosphonium salts with a reduced C_α_–P^+^ bond strength reacted with anisole and 1,3-dimethoxybenzene at much lower temperatures (Table 3, entries 3–7, 10, 11, 13, 17–19, 22 and 23). The most clear-cut results were obtained for tris[*p*-(trifluoromethyl)phenyl]phosphine derivatives. 1-Phthalimidoethyltris[*p*-(trifluoromethyl)phenyl]phosphonium tetrafluoroborate (**5e**) and 1-phthalimido-1-phenylmethyltris[*p*-(trifluoromethyl)phenyl]phosphonium tetrafluoroborate (**5h**) already react with anisole at 90 °C (Table 3, entries 5 and 10) and even at 60 °C when using microwave irradiation (Table 3, entry 7); the reaction of **5e** with 1,3-dimethoxybenzene started at 80 °C (Table 3, entry 18).

**Scheme 3:**
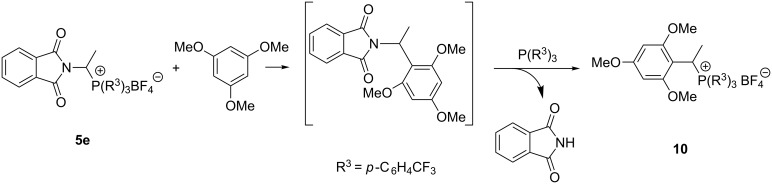
Reaction of imidophosphonium salt **5e** with 1,3,5-trimethoxybenzene.

All attempts to carry out the imidoalkylation of 1,3,5-trimethoxybenzene with phosphonium salt **5e** failed. In this case 1-(2,4,6-trimethoxyphenyl)ethyltris(4-trifluoromethylphenyl)phosphonium tetrafluoroborate (**10**) was isolated from the reaction mixture in 73% yield as the only reaction product. It seems that the steric congestion caused by two methoxy groups in *ortho* positions to the attacking carbon caused the displacement of a phthalimide anion by triarylphosphine in the primary reaction product and thus leading to the phosphonium salt **10** as an unexpected side product (Scheme 3).

The imidoalkylation of anisole and toluene gave the expected products as mixtures of *ortho* and *para* isomers in molar ratios ranging from 1.4:1.0 to 1.0:4.0 (Table 3). It seems that the steric congestion is the main factor governing the molar ratio of the regioisomers. Thus an excess of *ortho* isomers was obtained only in the case of 1-imidoalkylphosphonium salts derived from succinimide, with a relatively small imide ring (Table 3, entries 14 and 15). On the other hand, phosphonium salts derived from phthalimide gave in all cases some excess of the *para* isomers. Bulky phenyl or isobutyl substituents at the α-position favored the formation of *para* isomers (cf. Table 3, entries 1, 8 and 12 as well as 3 and 13). Toluene, with the relatively rigid methyl group gave a greater ratio of *para* isomers than anisole, with the more flexible methoxy group.

The 1-imidocarbenium cation derived from succinimide should be a molecule of higher energy and reactivity compared to the corresponding cation derived from phthalimide, in which the positive charge is delocalized into the aryl ring that provides some additional resonance stability, but reduces its electrophilicity. Consequently, the generation of the corresponding imidocarbenium cation from 1-succinimidoalkylphosphonium salts in the reaction with anisole started at a considerably higher temperature compared to the corresponding phthalimide derivatives (cf. Table 3, entry 14 against 1 and entry 15 against 5). However, the reaction yields were lower, probably because of the instability of phosphonium salt at these elevated temperatures.

To demonstrate the enhanced reactivity of 1-imidoalkylphosphonium salts as imidoalkylating agents, we successfully carried out their reactions with toluene (Table 3, entries 25–29), which was shown to be unreactive toward the most commonly used amidoalkylating agents [9–10]. In the reaction of toluene with phosphonium salt **5e** the elimination of triarylphosphonium tetrafluoroborate with formation of *N*-vinylphthalimide as a side product was observed. Fortunately, the addition of an equimolar amount of tetrafluoroboric acid to the reaction mixture effectively suppressed this side reaction.

The solubility of phosphonium salts in some aromatic hydrocarbons is limited. In such cases, to ensure an effective mass transfer of reactants, sonification of the reaction mixture was examined. The use of ultrasound reduced the reaction time and afforded slightly higher yields in two out of the three investigated reactions (Table 3, entries 6, 11 and 19).

## Conclusion

A convenient method for the synthesis of 1-imidoalkylphosphonium salts as a new class of reactive α-imidoalkylating agents was developed. The imidoalkylating properties of the obtained 1-imidoalkylphosphonium salts were tested in the Tscherniac–Einhorn-type reaction with aromatic hydrocarbons as a model reaction. The generation of 1-imidocarbenium cations through the thermal splitting of the C_α_–P^+^ bond seems to be the crucial step in the imidoalkylation of relatively reactive hydrogen carbons such as anisole or 1,3-dimethoxybenzene. Reactions with imidoalkylphosphonium salts that generate more stable carbenium cations, e.g., benzyl-type carbenium cations, started at a considerably lower temperature. The C_α_–P^+^ bond strength can be considerably reduced and consequently the generation of the corresponding 1-imidocarbenium cations can be markedly facilitated using 1-imidoalkylphosphonium salts derived from triarylphosphines with electron-withdrawing substituents such as tris(*m*-chorophenyl)phosphine, tris(*p*-chlorophenyl)phosphine and tris[*p*-(trifluoromethyl)phenyl]phosphine. Phosphonium salts with a reduced C_α_–P^+^ bond strength react with aromatic hydrocarbons at a much lower temperature. In addition, microwave irradiation considerably facilitates the splitting of the highly polar C_α_–P^+^ bond. The enhanced reactivity of 1-imidoalkylphosphonium salts as imidoalkylating agents was demonstrated by the successful imidoalkylation of the scarcely reactive toluene. The 1-imidoalkylphosphonium salts derived from electron-withdrawing triarylphosphines can be considered a new interesting class of imidoalkylating agents of high reactivity without the need of a catalyst. Starting from a wide range of amino acids they are easily synthesized and isolated by simple work-up procedures as crystalline and stable compounds.

## Experimental

**General methods:** Melting points were determined in capillaries and are uncorrected. IR spectra were measured on an FT IR spectrophotometer (ATR method). ^1^H and ^13^C NMR spectra were recorded at operating frequencies of 400 and 100 MHz, respectively, using TMS as internal standard. ^31^P and ^19^F NMR spectra were recorded at operating frequencies of 161.9 and 376 MHz, respectively, without the resonance shift standard, with respect to H_3_PO_4_ and CFCl_3,_ respectively, as zero ppm. All chemical shifts (δ) are reported in ppm, and coupling constants (*J*) are given in hertz (Hz). High-resolution mass spectrometry (HRMS) analyses were performed on a Waters Xevo G2 Q-TOF mass spectrometer equipped with an ESI source operating in the positive ion mode. The accurate mass and composition for the molecular ion adducts were calculated using the MassLynx software incorporated within the instrument. Spectroscopic properties of all synthesized compounds as well as ^1^H NMR, ^13^C NMR, ^31^P NMR and ^19^F NMR spectra of all new compounds are given in Supporting Information File 1.

**Synthesis of 2-imidoalkanecarboxylic acids 6 from α-amino acids:** The synthesis of 2-imidoalkanecarboxylic acids **6** was carried out based on the modified procedure described by McKenzie and Walker [32]. The corresponding α-amino acid (10 mmol) was heated with phthalic or succinic anhydride (11 mmol) at 170 °C or 140 °C under reduced pressure for the time given in Table 1. The crude product was recrystallized from toluene.

**Synthesis of 2-(1,8-naphthalimido)propionic acid:** The synthesis of 2-(1,8-naphthalimido)propionic acid was carried out based on the procedure described by Reger [35]. To a stirred aqueous solution of L-alanine (1.96 g, 22 mmol) potassium hydroxide (1.12 g, 20 mmol) was added. After 20 min of stirring, 1,8-naphthalic anhydride (3.96 g, 20 mmol) and ethanol (75 mmol) were added to the amino acid solution. The reaction mixture was heated under reflux for 5 h. Then a solution of 1 M HCl (20 cm^3^, 20 mmol) was added. The precipitated product was filtered and washed with distilled water (4 × 50 cm^3^) and ice-cold absolute ethanol (50 cm^3^). The obtained solid was dried under reduced pressure to give analytically pure 2-(1,8-naphthalimido)propionic acid in 84% yield.

**Decarboxylative α-methoxylation of 2-imidoalkanecarboxylic acids 6 to *N*-(1-methoxyalkyl)imides 7** [18]**:** To an undivided electrolyzer (100 cm^3^) with a thermostatic jacket equipped with a magnetic stirrer, a cylindrical Pt mesh anode (47 cm^2^) and cathode (44 cm^2^), methanol (30 cm^3^), 2-(*N*-imido)alkanecarboxylic acids **6** (3.0 mmol) and silica gel-supported piperidine (SiO_2_-Pip; 200 mg, 0.22 mmol) were added. The electrolysis was carried out under stirring at a current density of 0.3 A/dm^2^ at 0 °C until a 2.7–3.75 F/mol charge had passed. Solid SiO_2_-Pip was filtered off, methanol was evaporated under reduced pressure, and the product was isolated by column chromatography (CH_2_Cl_2_/MeOH/Et_3_N 80:1:1, v/v/v). The crystalline crude compounds **7a**, **7b** and **7h** were recrystallized from toluene.

**Transformation of *N*-(1-methoxyalkyl)imides 7 to phosphonium salts 5; general procedure:** To a solution of triarylphosphine (1 mmol) in DCM, tetrafluoroboric acid diethyl ether complex (136 µL, 161.9 mg, 1 mmol) was added at 0 °C. After 2 h of stirring at room temperature, *N*-(1-methoxyalkyl)imides **7** (1 mmol) and sodium bromide (15.4 mg, 0.15 mmol) were added to the reaction mixture and the solvent was evaporated to dryness under reduced pressure. The residue was then heated at 85–140 °C under reduced pressure for the time given in Table 2. The crude product was dissolved in DCM, sodium bromide was removed by decantation and phosphonium salt **5** was precipitated with Et_2_O, which was separated by filtration and dried under reduced pressure.

**Transformation of 1-(*N*-imido)alkyltriarylphosphonium salts 5 to *N*-(1-arylalkyl)imides 9; general procedure:** A suspension of 1-(*N*-imido)alkyltriarylphosphonium salt **5** (0.1 mmol) in an aromatic compound (1 cm^3^) was placed in a glass vial sealed with a screw-cap. To improve the solubility of the phosphonium salts in some cases an additional solvent was added (Table 3). In the reaction of toluene with phosphonium salt **5e** the addition of tetrafluoroboric acid diethyl ether complex (HBF_4_·Et_2_O, 13.6 µL, 16.2 mg, 0.1 mmol) to the reaction mixture was necessary to suppress the side reaction of *N*-vinylphthalimide formation. The reaction mixture was vigorously stirred and heated under the conditions given in Table 3. The reaction mixture was evaporated to dryness under reduced pressure and the product was isolated by column chromatography (toluene/ethyl acetate 10:1, v/v). The crude crystalline compounds **9aa**, **9ab**, **9bb**, **9db**, **9e**, **9f**, **9g**, **9ia** were recrystallized from toluene or toluene/hexane 1:1, v/v.

**Microwave assisted synthesis of *N*-(1-arylalkyl)imides 9:** A suspension of the 1-(*N*-imido)alkyltriarylphosphonium salt **5** (0.25 mmol) in an aromatic compound (2.5 cm^3^) was placed in a glass vial sealed with a screw-cap. To improve the solubility of the phosphonium salts, in some cases an additional solvent was added (Table 3). In the reaction of toluene with phosphonium salt **5e** the addition of tetrafluoroboric acid diethyl ether complex (HBF_4_·Et_2_O, 17.0 µL, 20.3 mg, 0.125 mmol) to the reaction mixture was necessary to suppress the side reaction of *N*-vinylphthalimide formation. The reaction mixture was vigorously stirred and exposed to microwave irradiation in a CEM Matthews microwave reactor under the conditions given in Table 3. The reaction mixture was then evaporated to dryness under reduced pressure and the product was isolated by column chromatography (toluene/ethyl acetate 10:1, v/v). The crude crystalline compounds **9aa**, **9ab**, **9bb**, **9ia** were recrystallized from toluene or toluene/hexane 1:1, v/v.

**Ultrasound assisted synthesis of *N*-(1-arylalkyl)imides 9:** A suspension of 1-(*N*-imido)alkyltriarylphosphonium salt **5** (0.1 mmol) in an aromatic compound (1 cm^3^) was placed in a glass vial sealed with a screw-cap and sonicated using an Elmasonic 10H laboratory ultrasonic bath (37 kHz, 30 W), at the temperature given in Table 3. To improve the solubility of the phosphonium salts, in some cases an additional solvent was added (Table 3). The solvent was then removed under reduced pressure and the product was isolated by column chromatography (toluene/ethyl acetate 10:1, v/v). The crude crystalline compounds **9aa**, **9ab**, **9bb** and **9e** were recrystallized from toluene or toluene/hexane 1:1, v/v.

**Reaction of phosphonium tetrafluoroborate 5e with 1,3,5-trimethoxybenzene:** To a solution of 1-(*N*-phthalimido)ethyltris(4-trifluoromethylphenyl)phosphonium tetrafluoroborate (**5e**, 0.5 mmol, 363.6 mg) in DCM (2 cm^3^), 1,3,5-trimethoxybenzene (2.5 mmol, 420.5 mg) was added. After stirring, the mixture was evaporated to dryness under reduced pressure and the residue was heated at 110 °C for 2 h. The crude product was washed with toluene (50 °C, 3 × 3 cm^3^). The crude phosphonium salt **10** was dissolved in DCM (1 cm^3^), precipitated with Et_2_O, separated by filtration and dried under reduced pressure to give 1-(2,4,6-trimethoxyphenyl)ethyltris(4-trifluoromethylphenyl)phosphonium tetrafluoroborate (**10**) in 73% yield.

## Supporting Information

File 1 Spectroscopic properties of all synthesized compounds **5**–**10** and ^1^H, ^13^C, ^31^P, and ^19^F NMR spectra of all new compounds.
