# Supplementary material for: 1-Imidoalkylphosphonium salts with modulated Cα–P+ bond strength: synthesis and application as new active α-imidoalkylating agents
Source: Beilstein J Org Chem. 2017 Jul 24;13:1446–55. doi: 10.3762/bjoc.13.142 (PMC5550811; doi:10.3762/bjoc.13.142)
Supplement: File 1 — Spectroscopic properties of all synthesized compounds 5–10 and 1H, 13C, 31P, and 19F NMR spectra of all new compounds. [file Beilstein_J_Org_Chem-13-1446-s001.pdf]

Supporting information

for

1-Imidoalkylphosphonium salts with modulated C_α–P^+ bond strength: synthesis and application as new active α-imidoalkylating agents

Jakub Adamek[*1,2,§], Roman Mazurkiewicz[1,2], Anna Węgrzyk[1,2], and Karol Erfurt[3]

Address: [1]Department of Organic Chemistry, Bioorganic Chemistry and Biotechnology, Silesian University of Technology, B. Krzywoustego 4, 44-100 Gliwice, Poland, [2]Biotechnology Centre of Silesian University of Technology, B. Krzywoustego 8, 44-100 Gliwice, Poland and [3]Department of Chemical Organic Technology and Petrochemistry, Silesian University of Technology, B. Krzywoustego 4, 44-100 Gliwice, Poland

Email: Jakub Adamek - jakub.adamek@polsl.pl

[*]Corresponding author:

[§]Tel: +48 32 237 10 81; Fax: +48 32 237 20 94

Spectroscopic properties of all synthesized compounds 5–10 and 1H, 13C, 31P, and 19F NMR spectra of all new compounds

Table of content

Spectroscopic properties of all synthesized compounds **5**–**10** S2

References S10

^{1}H NMR, ^{13}C NMR, ^{31}P NMR, ^{19}F NMR of all new compounds **5**–**10** S11

IR spectra for selected compounds S90

Phthalimidoacetic acid[1] **(6a).** Colorless crystals (1.03 g, 50% yield), mp 188.5-190.5°C. ^{1}H NMR (400 MHz, DMSO-d6) δ 7.98-7.85 (m, 4H), 4.32 (s, 2H) ppm; ^{13}C NMR (100 MHz, DMSO-d6) δ 168.8, 167.2, 134.8, 131.4, 123.4, 38.9 ppm; IR (ATR) 2937, 1772, 1706, 1409, 1389, 1244 cm^{-1}.

2-Phthalimidopropionic acid[1] **(6b).** Colorless crystals (2.06 g, 94% yield), mp 166.0-168.0°C. ^{1}H NMR (400 MHz, DMSO-d6) δ 7.95-7.85 (m, 4H), 4.87 (q, J = 7.3 Hz, 1H), 1.56 (d, J = 7.3 Hz, 3H) ppm; ^{13}C NMR (100 MHz, DMSO-d6) δ 171.0, 167.1, 134.7, 131.3, 123.3, 46.1, 14.8 ppm; IR (ATR) 2557, 1764, 1704, 1387 cm^{-1}.

3-Phenyl-2-phthalimidopropionic acid[1] **(6c).** Colorless crystals (2.75 g, 93% yield), mp 176.0-177.0°C. ^{1}H NMR (400 MHz, $CDCl_3$) δ 9.73 (s, 1H), 7.81-7.62 (m, 4H), 7.21-7.09 (m, 5H), 5.23 (dd, J_1 = 8.8 Hz, J_2 = 7.8 Hz, 1H), 3.59 (d, J = 8.5 Hz, 2H) ppm; ^{13}C NMR (100 MHz, $CDCl_3$) δ 174.5, 167.4, 136.4, 134.1, 131.4, 128.8, 128.6, 126.9, 123.5, 53.0, 34.4 ppm; IR (ATR) 3027, 2606, 1774, 1705, 1382, 1272, 1255 cm^{-1}.

2-Phenyl-2-phthalimidoacetic acid[1] **(6d).** Colorless crystals (2.11 g, 75% yield), mp 170.0-171.0°C. ^{1}H NMR (400 MHz, DMSO-d6) δ 12.19 (s, 1H), 6.74-6.62 (m, 4H), 6.28-6.20 (m, 2H), 6.17-6.05 (m, 3H), 4.79 (s, 1H) ppm; ^{13}C NMR (100 MHz, DMSO-d6) δ 168.9, 166.9, 135.0, 135.0, 130.1, 129.1, 128.1, 127.9, 123.5, 55.1 ppm; IR (ATR) 2599, 1773, 1705, 1378 cm^{-1}.

4-Methyl-2-phthalimidopentanoic acid[1] **(6e).** Colorless crystals (2.35 g, 90% yield), mp 126.5-129.0°C. ^{1}H NMR (400 MHz, $CDCl_3$) δ 10.10 (s, 1H), 7.89-7.82 (m, 2H), 7.76-7.70 (m, 2H), 4.99 (dd, J_1 = 11.5 Hz, J_2 = 4,4 Hz, 1H), 2.36 (ddd, J_1 = 14.3 Hz, J_2 = 11.5 Hz, J_3 = 4.2 Hz, 1H), 1.95 (ddd, J_1 = 14.4 Hz, J_2 = 10.2 Hz, J_3 = 4.4 Hz, 1H), 1.56-1.43 (m, 1H), 0.92 (d, J = 6.7 Hz, 3H), 0.94 (d, J = 6.6 Hz, 3H) ppm; ^{13}C NMR (100 MHz, $CDCl_3$) δ 175.7, 167.6, 134.2, 131.7, 123.6, 50.4, 37.0, 25.1, 23.1, 21.0 ppm; IR (ATR) 2962, 1779, 1707, 1384, 1280 cm^{-1}.

Succinimidoacetic acid[1] **(6f).** Colorless crystals (722.8 mg, 46% yield), mp 104.0-106.0°C. ^{1}H NMR (400 MHz, DMSO-d6) δ 4.06 (s, 2H), 2.72 (s, 4H) ppm; ^{13}C NMR (100 MHz, DMSO-d6) δ 177.4, 168.8, 39.7, 28.4 ppm; IR (ATR) 2944, 1776, 1733, 1656, 1421, 1201, 1177 cm^{-1}.

2-Succinimidopropionic acid[1] **(6g).** Colorless crystals (1.15 g, 67% yield), mp 106.0-108.0°C. ^{1}H NMR (400 MHz, DMSO-d6) δ 4.87 (q, J = 7.3 Hz, 1H), 2.76 (s, 4H), 1.58 (d, J = 7.3 Hz, 3H) ppm; ^{13}C NMR (100 MHz, DMSO-d6) δ 176.2, 174.0, 47.8, 28.1, 14.1 ppm; IR (ATR) 2946, 1690, 1396, 1196 cm^{-1}.

2-(1,8-Naphthalimido)propionic acid[2] **(6h).** Colorless crystals (4.52 g, 84% yield), mp 260.5-261.5°C. ^{1}H NMR (400 MHz, $CDCl_3$/DMSO-d6) δ 8.60-8.50 (m, 2H), 8.27-8.23 (m, 2H), 7.80-7.73 (m, 2H), 5.71 (q, J = 6.9 Hz, 1H), 1.67 (d, J = 7.2 Hz, 3H) ppm; ^{13}C NMR (100 MHz, $CDCl_3$/DMSO-d6) δ 171.5, 162.6, 133.5, 130.8, 130.5, 127.3, 126.2, 121.6, 48.2, 14.0 ppm; IR (ATR) 3157, 2957, 1755, 1698, 1666, 1639, 1584, 1379, 1344, 1236, 1205, 1087 cm^{-1}.

***N*-Methoxymethylphthalimide**[3] **(7a).** Colorless crystals (160.6 mg, 28% yield), mp 118.0-120.0°C. ^{1}H NMR (400 MHz, $CDCl_3$) δ 7.95-7.89 (m, 2H), 7.80-7.74 (m, 2H), 5.10 (s, 2H), 3.42 (s, 3H) ppm; ^{13}C NMR (100 MHz, $CDCl_3$) δ 167.9, 134.4, 131.9, 123.7, 68.7, 57.4 ppm; IR (ATR) 2933, 1774, 1709, 1345, 1326, 1164, 1082 cm^{-1}.

***N*-(1-Methoxyethyl)phthalimide**[4] **(7b).** Colorless crystals (400.2 mg, 65% yield), mp 113.0-115.5°C. ^{1}H NMR (400 MHz, $CDCl_3$) δ 7.91-7.86 (m, 2H), 7.78-7.72 (m, 2H), 5.48 (q, J = 6.3 Hz, 1H), 3.34 (s, 3H), 1.79 (d, J = 6.3 Hz, 3H) ppm; ^{13}C NMR (100 MHz, $CDCl_3$) δ 167.9, 134.2, 131.7, 123.5, 79.7, 56.3, 19.1 ppm; IR (ATR) 2915, 1775, 1698, 1324, 1117, 1047 cm^{-1}.

***N*-(1-Methoxy-2-phenylethyl)phthalimide (7c).** Yellow oil (354.5 mg, 42% yield). ^{1}H NMR (400 MHz, $CDCl_3$) δ 7.87-7.80 (m, 2H), 7.75-7.68 (m, 2H), 7.24-7.13 (m, 5H), 5.53 (dd, J_1 = 7.7 Hz, J_2 = 6.8 Hz, 1H), 3.56 (dd, J_1 = 14.5 Hz, J_2 = 7.3 Hz, 1H), 3.51 (dd, J_1 = 14.1 Hz, J_2 = 7.8 Hz, 1H), 3.35 (s, 3H) ppm; ^{13}C NMR (100 MHz, $CDCl_3$) δ 168.0, 136.3, 134.2, 131.5, 129.2, 128.5, 126.7, 123.5, 84.1, 56.7, 38.6 ppm; IR (ATR) 2931, 1775, 1709, 1349, 1322, 1112, 1070 cm^{-1}; HRMS (ESI-TOF) calcd for $C_{17}H_{15}NO_3Na$ $[M + Na]^+$ 304.0950 found 304.0952.

***N*-(1-Methoxy-1-phenylmethyl)phthalimide (7d).** Yellow oil (481.1 mg, 60% yield). ^{1}H NMR (400 MHz, $CDCl_3$) δ 7.89-7.82 (m, 2H), 7.77-7.71 (m, 2H), 7.57-7.52 (m, 2H), 7.39-7.28 (m, 3H), 6.38 (s, 1H), 3.54 (s, 3H) ppm; ^{13}C NMR (100 MHz, $CDCl_3$) δ 167.4, 136.9, 134.3, 131.7, 128.4, 128.2, 126.2, 123.6, 82.8, 56.8 ppm; IR (ATR) 2935, 1773, 1712, 1317, 1088, 1074 cm^{-1}; HRMS (ESI-TOF) calcd for $C_{16}H_{13}NO_3Na$ $[M + Na]^+$ 290.0793 found 290.0797.

***N*-(1-Methoxy-3-methylbutyl)phthalimide**[4] **(7e).** Yellow oil (341.3 mg, 46% yield). ^{1}H NMR (400 MHz, $CDCl_3$) δ 7.92-7.86 (m, 2H), 7.80-7.74 (m, 2H), 5.37 (t, J = 7.1 Hz, 1H), 3.35 (s, 3H), 2.22-2.00 (m, 2H), 1.76-1.59 (m, 1H), 0.96 (d, J = 2.3 Hz, 3H), 0.94 (d, J = 2.3 Hz, 3H) ppm; ^{13}C NMR (100 MHz, $CDCl_3$) δ 168.1, 134.2, 131.7, 123.5, 82.4, 56.4, 41.0, 24.9, 22.5, 22.4 ppm; IR (ATR) 2956, 1774, 1709, 1349, 1320, 1114, 1070 cm^{-1}.

***N*-Methoxymethylsuccinimide**[3] **(7f).** Colorless oil (141.7 mg, 33% yield). ^{1}H NMR (400 MHz, $CDCl_3$) δ 4.89 (s, 2H), 3.38 (s, 3H), 2.77 (s, 4H) ppm; ^{13}C NMR (100 MHz, $CDCl_3$) δ 176.8, 69.2, 57.7, 28.1 ppm; IR (ATR) 2943, 1779, 1701, 1351, 1244, 1183, 1111, 1079 cm^{-1}.

***N*-(1-Methoxyethyl)succinimide (7g).** Colorless oil (400.8 mg, 85% yield). ^{1}H NMR (400 MHz, $CDCl_3$) δ 5.32 (q, J = 6.3 Hz, 1H), 3.30 (s, 3H), 2.72 (s, 4H), 1.67 (d, J = 6.4 Hz, 3H) ppm; ^{13}C NMR (100 MHz, $CDCl_3$) δ 176.8, 80.5, 56.5, 28.0, 18.2 ppm; IR (ATR) 2942, 1697, 1350, 1196, 1097 cm^{-1}; HRMS (ESI-TOF) calcd for $C_7H_{11}NO_3Na$ $[M + Na]^+$ 180.0637 found 180.0634.

***N*-(1-Methoxyethyl)-1,8-naphthalimide (7h).** Pale yellow crystals (398.3 mg, 52% yield), mp 123.0-125.0°C. ^{1}H NMR (400 MHz, $CDCl_3$) δ 8.62 (dd, J_1 = 7.3 Hz, J_2 = 1.1 Hz, 2H), 8.22 (dd, J_1 = 8.4 Hz, J_2 = 1.0 Hz, 2H), 7.77 (dd, J_1 = 8.2 Hz, J_2 = 7.3 Hz, 2H), 6.39 (q, J = 6.3 Hz, 1H), 3.42 (s, 3H), 1.90 (d, J = 6.3 Hz, 3H) ppm; ^{13}C NMR (100 MHz, $CDCl_3$) δ 164.3,

133.8, 131.5, 131.3, 128.4, 127.0, 122.8, 82.7, 56.7, 19.5 ppm; IR (ATR) 2941, 1698, 1656, 1586, 1334, 1237, 1099 cm^{-1}; HRMS (ESI-TOF) calcd for $C_{15}H_{13}NO_3Na$ $[M + Na]^+$ 278.0793 found 278.0795.

1-(*N*-Phthalimido)methyltriphenylphosphonium tetrafluoroborate (5a). Colorless crystals (249.5 mg, 49% yield), mp 238.0-240.0°C. ^{1}H NMR (400 MHz, CD_3CN) δ 7.95-7.67 (m, 19H), 5.46 (d, J = 4.1 Hz, 2H) ppm; ^{13}C NMR (100 MHz, CD_3CN) δ 167.7, 136.8 (d, J = 3.1 Hz), 136.1, 135.5 (d, J = 10.3 Hz), 132.2, 131.3 (d, J = 12.8 Hz), 124.7, 117.0 (d, J = 85.4 Hz), 35.6 (d, J = 60.3 Hz) ppm; ^{31}P NMR (161.9 MHz, CD_3CN) δ 24.7 ppm; IR (ATR) 2920, 1776, 1719, 1387, 1369, 1038 cm^{-1}; HRMS (ESI-TOF) calcd for $C_{27}H_{21}NO_2P$ $[M^+]$ 422.1310 found 422.1327.

1-(*N*-Phthalimido)ethyltriphenylphosphonium tetrafluoroborate (5b). Colorless crystals (481.4 mg, 92% yield), mp 200.0-201.5°C. ^{1}H NMR (400 MHz, $CDCl_3$) δ 7.91-7.60 (m, 19H), 6.22 (dq, J_1 = 10.5 Hz, J_2 = 7.5 Hz, 1H), 2.05 (dd, J_1 = 16.7 Hz, J_2 = 7.5 Hz, 3H) ppm; ^{13}C NMR (100 MHz, $CDCl_3$) δ 166.6 (d, J = 1.2 Hz), 135.9 (d, J = 3.1 Hz), 135.3, 134.4 (d, J = 9.8 Hz), 130.7 (d, J = 12.6 Hz), 130.4, 124.1, 116.1 (d, J = 82.8 Hz), 44.6 (d, J = 54.0 Hz), 16.5 (d, J = 4.7 Hz) ppm; ^{31}P NMR (161.9 MHz, $CDCl_3$) δ 28.7 ppm; IR (ATR) 3066, 1779, 1716, 1380, 1060, 1039 cm^{-1}; HRMS (ESI-TOF) calcd for $C_{28}H_{23}NO_2P$ $[M^+]$ 436.1466 found 436.1467.

1-(*N*-Phthalimido)ethyltris(3-chlorophenyl)phosphonium tetrafluoroborate (5c). Colorless crystals (520.1 mg, 83% yield), mp 141.5-143.0°C. ^{1}H NMR (400 MHz, CD_3CN) δ 7.93-7.89 (m, 3H), 7.87-7.79 (m, 7H), 7.77-7.66 (m, 6H), 6.25 (dq, J_1 = 10.0 Hz, J_2 = 7.4 Hz, 1H), 2.00 (dd, J_1 = 17.2 Hz, J_2 = 7.4 Hz, 3H) ppm; ^{13}C NMR (100 MHz, CD_3CN) δ 167.9 (d, J = 1.1 Hz), 137.4 (d, J = 17.0 Hz), 137.2 (d, J = 3.0 Hz), 136.4, 134.9 (d, J = 11.3 Hz), 134.4 (d, J = 9.6 Hz), 133.2 (d, J = 13.9 Hz), 131.8, 124.9, 118.7 (d, J = 84.3 Hz), 45.4 (d, J = 54.3 Hz), 15.8 (d, J = 3.5 Hz) ppm; ^{31}P NMR (161.9 MHz, CD_3CN) δ 26.5 ppm; IR (ATR) 3067, 1774, 1707, 1381, 1040 cm^{-1}; HRMS (ESI-TOF) calcd for $C_{28}H_{20}Cl_3NO_2P$ $[M^+]$ 538.0297 found 538.0296.

1-(*N*-Phthalimido)ethyltris(4-chlorophenyl)phosphonium tetrafluoroborate (5d). Colorless crystals (557.7 mg, 89% yield), mp 141.0-142.5°C. ^{1}H NMR (400 MHz, $CDCl_3$) δ 7.83-7.64 (m, 16H), 6.24 (dq, J_1 = 10.0 Hz, J_2 = 7.4 Hz, 1H), 2.02 (dd, J_1 = 17.2 Hz, J_2 = 7.4 Hz, 3H) ppm; ^{13}C NMR (100 MHz, $CDCl_3$) δ 166.5 (d, J = 1.2 Hz), 143.5 (d, J = 3.7 Hz), 135.7 (d, J = 11.1 Hz), 135.5, 131.3 (d, J = 13.6 Hz), 130.4, 124.3, 113.9 (d, J = 86.0 Hz), 44.5 (d, J = 54.3 Hz), 16.2 (d, J = 3.8 Hz) ppm; ^{31}P NMR (161.9 MHz, $CDCl_3$) δ 28.6 ppm; IR (ATR) 3097, 1780, 1717, 1380, 1047, 1008 cm^{-1}; HRMS (ESI-TOF) calcd for $C_{28}H_{20}Cl_3NO_2P$ $[M^+]$ 538.0297 found 538.0298.

1-(*N*-Phthalimido)ethyltris(4-trifluoromethylphenyl)phosphonium tetrafluoroborate (5e). Colorless crystals (690.9 mg, 95% yield), mp 126.0-127.5°C. ^{1}H NMR (400 MHz, $CDCl_3$) δ 8.10-8.00 (m, 6H), 7.99-7.92 (m, 6H), 7.81-7.71 (m, 4H), 6.49 (dq, J_1 = 9.6 Hz, J_2 = 7.3 Hz, 1H), 2.07 (dd, J_1 = 17.7 Hz, J_2 = 7.4 Hz, 3H) ppm; ^{13}C NMR (100 MHz, $CDCl_3$) δ 166.4 (d, J = 1.2 Hz), 137.5 (qd, J_1 = 33.9 Hz, J_2 = 3.3 Hz), 135.6, 135.4 (d, J = 10.6 Hz), 130.3, 127.7 (dq, J_1 = 13.1 Hz, J_2 = 3.6 Hz), 124.3, 122.6 (qd, J_1 = 273.6 Hz, J_2 = 1.2 Hz), 119.7 (d, J = 81.8 Hz), 44.2 (d, J = 52.1 Hz), 16.3 (d, J = 3.8 Hz) ppm; ^{31}P

NMR (161.9 MHz, $CDCl_3$) δ 28.9 ppm; ^{19}F NMR (376 MHz, $CDCl_3$) δ -63.9 (s, CF_3) ppm; IR (ATR) 3107, 1782, 1720, 1318, 1131, 1060, 1012 cm^{-1}; HRMS (ESI-TOF) calcd for $C_{31}H_{20}F_9NO_2P$ [M^+] 640.1088 found 640.1088.

1-(*N*-Phthalimido)phenylmethyltriphenylphosphonium tetrafluoroborate (5f). Colorless crystals (550.2 mg, 94% yield), mp 226.0-228.0°C. ^1H NMR (400 MHz, $CDCl_3$) δ 7.91-7.80 (m, 7H), 7.75-7.61 (m, 12H), 7.43-7.23 (m, 5H), 7.18 (d, J = 15.4 Hz, 1H) ppm; ^{13}C NMR (100 MHz, $CDCl_3$) δ 166.8 (d, J = 2.3 Hz), 135.9 (d, J = 3.1 Hz), 135.8, 134.5 (d, J = 9.7 Hz), 133.9 (d, J = 14.6 Hz), 130.6 (d, J = 12.7 Hz), 130.3, 129.7 (d, J = 1.5 Hz), 129.6 (d, J = 5.2 Hz), 129.4 (d, J = 2.1 Hz), 124.5, 116.8 (d, J = 83.6 Hz), 52.5 (d, J = 54.3 Hz) ppm; ^{31}P NMR (161.9 MHz, $CDCl_3$) δ 27.0 ppm; IR (ATR) 3077, 1784, 1719, 1329, 1047 cm^{-1}; HRMS (ESI-TOF) calcd for $C_{33}H_{25}NO_2P$ [M^+] 498.1623 found 498.1620.

1-(*N*-Phthalimido)phenylmethyltris(3-chlorophenyl)phosphonium tetrafluoroborate (5g). Resin (606.1 mg, 88% yield). ^1H NMR (400 MHz, $CDCl_3$) δ 7.90-7.83 (m, 4H), 7.82-7.72 (m, 9H), 7.51-7.40 (m, 4H), 7.37-7.30 (m, 2H), 7.28-7.20 (m, 3H) ppm; ^{13}C NMR (100 MHz, $CDCl_3$) δ 166.7 (d, J = 2.8 Hz), 136.7 (d, J = 16.8 Hz), 136.3 (d, J = 3.0 Hz), 135.8, 133.3 (d, J = 4.2 Hz), 133.2 (d, J = 5.6 Hz), 132.7 (d, J = 14.2 Hz), 131.2 (d, J = 2.7 Hz), 130.3, 129.9 (d, J = 1.9 Hz), 129.6 (d, J = 5.2 Hz), 128.7 (d, J = 1.2 Hz), 124.7, 118.4 (d, J = 83.6 Hz), 52.6 (d, J = 53.7 Hz) ppm; ^{31}P NMR (161.9 MHz, $CDCl_3$) δ 26.0 ppm; IR (ATR) 3075, 1790, 1719, 1380, 1051 cm^{-1}; HRMS (ESI-TOF) calcd for $C_{33}H_{22}Cl_3NO_2P$ [M^+] 600.0454 found 600.0449.

1-(*N*-Phthalimido)phenylmethyltris(4-trifluoromethylphenyl)phosphonium tetrafluoroborate (5h). Colorless crystals (631.4 mg, 80% yield), mp 131.5-132.5°C. ^1H NMR (400 MHz, $CDCl_3$) δ 8.04-7.94 (m, 6H), 7.92-7.85 (m, 6H), 7.79 (s, 4H), 7.55 (d, J = 14.2 Hz, 1H), 7.43-7.37 (m, 1H), 7.31-7.23 (m, 2H), 7.17-7.10 (m, 2H) ppm; ^{13}C NMR (100 MHz, $CDCl_3$) δ 166.4, 136.8 (qd, J_1 = 33.5 Hz, J_2 = 3.5 Hz), 135.6, 135.3 (d, J = 10.2 Hz), 131.1 (d, J = 2.7 Hz), 130.5, 129.9 (d, J = 2.2 Hz), 129.5 (d, J = 5.3 Hz), 129.0, 127.2 (dq, J_1 = 13.3 Hz, J_2 = 3.6 Hz), 124.7, 122.6 (q, J = 271.9 Hz), 121.7 (d, J = 83.8 Hz), 51.9 (d, J = 54.1 Hz) ppm; ^{31}P NMR (161.9 MHz, $CDCl_3$) δ 26.4 ppm; ^{19}F NMR (376 MHz, $CDCl_3$) δ -63.8 (s, CF_3) ppm; IR (ATR) 3109, 1717, 1319, 1130, 1060, 1010 cm^{-1}; HRMS (ESI-TOF) calcd for $C_{36}H_{22}F_9NO_2P$ [M^+] 702.1244 found 702.1246.

3-Methyl-1-(*N*-phthalimido)butyltriphenylphosphonium tetrafluoroborate (5i). Colorless crystals (446.6 mg, 79% yield), mp 240.0-241.5°C. ^1H NMR (400 MHz, $CDCl_3$) δ 7.90-7.65 (m, 19H), 6.01 (td, J_1 = 12.7 Hz, J_2 = 2.7 Hz, 1H), 2.83-2.73 (m, 1H), 1.71-1.61 (m, 1H), 1.61-1.51 (m, 1H), 1.05 (d, J = 6.4 Hz, 3H), 0.93 (d, J = 6.5 Hz, 3H) ppm; ^{13}C NMR (100 MHz, $CDCl_3$) δ 166.9 (d, J = 1.1 Hz), 136.1 (d, J = 3.1 Hz), 135.7, 134.2 (d, J = 9.8 Hz), 130.8 (d, J = 12.6 Hz), 130.1, 124.3, 115.8 (d, J = 82.9 Hz), 47.5 (d, J = 53.2 Hz), 37.9 (d, J = 3.1 Hz), 26.0 (d, J = 11.5 Hz), 22.9, 20.6 ppm; ^{31}P NMR (161.9 MHz, $CDCl_3$) δ 31.3 ppm; IR (ATR) 2970, 1782, 1719, 1372, 1050 cm^{-1}; HRMS (ESI-TOF) calcd for $C_{31}H_{29}NO_2P$ [M^+] 478.1936 found 478.1938.

3-Methyl-1-(*N*-phthalimido)butyltris(3-chlorophenyl)phosphonium tetrafluoroborate (5j). Colorless crystals (501.5 mg, 75% yield), mp 192.0-194.0°C. ^{1}H NMR (400 MHz, CD_3CN) δ 7.95-7.66 (m, 16H), 6.03 (td, J_1 = 12.4 Hz, J_2 = 2.5 Hz, 1H), 2.75 (dddd, J_1 = 14.2 Hz, J_2 = 12.8 Hz, J_3 = 4.9 Hz, J_4 = 3.3 Hz, 1H), 1.84 (dddd, J_1 = 14.3 Hz, J_2 = 10.4 Hz, J_3 = 5.1 Hz, J_4 = 2.5 Hz, 1H), 1.71-1.58 (m, 1H), 1.03 (d, J = 6.5 Hz, 3H), 0.89 (d, J = 6.6 Hz, 3H) ppm; ^{13}C NMR (100 MHz, CD_3CN) δ 168.1 (d, J = 1.2 Hz), 137.4 (d, J = 17.0 Hz), 137.2 (d, J = 3.0 Hz), 136.7, 134.9 (d, J = 11.4 Hz), 134.3 (d, J = 9.6 Hz), 133.3 (d, J = 14.0 Hz), 131.5, 125.1, 118.7 (d, J = 82.7 Hz), 48.7 (d, J = 53.1 Hz), 37.4 (d, J = 3.2 Hz), 26.5 (d, J = 12.1 Hz), 23.0, 20.7 ppm; ^{31}P NMR (161.9 MHz, CD_3CN) δ 26.4 ppm; IR (ATR) 2963, 2901, 1780, 1719, 1371, 1328, 1131, 1050 cm^{-1}; HRMS (ESI-TOF) calcd for $C_{31}H_{26}Cl_3NO_2P$ [M^+] 580.0767 found 580.0767.

1-(*N*-Succinimido)methyltriphenylphosphonium tetrafluoroborate (5k). Colorless crystals (299.8 mg, 65% yield), mp 215.0-217.0°C. ^{1}H NMR (400 MHz, CD_3CN) δ 7.96-7.88 (m, 3H), 7.81-7.70 (m, 12H), 5.20 (d, J = 5.2 Hz, 2H), 2.53 (d, J = 1.2 Hz, 4H) ppm; ^{13}C NMR (100 MHz, CD_3CN) δ 177.3, 136.8 (d, J = 3.2 Hz), 135.4 (d, J = 10.3 Hz), 131.3 (d, J = 12.9 Hz), 117.1 (d, J = 85.6 Hz), 35.6 (d, J = 60.1 Hz), 28.8 ppm; ^{31}P NMR (161.9 MHz, CD_3CN) δ 20.0 ppm; IR (ATR) 3107, 1714, 1443, 1383, 1112, 1044, 996 cm^{-1}; HRMS (ESI-TOF) calcd for $C_{23}H_{21}NO_2P$ [M^+] 374.1310 found 374.1312.

1-(*N*-Succinimido)ethyltriphenylphosphonium tetrafluoroborate (5l). Colorless crystals (275.6 mg, 58% yield), mp 241.0-242.5°C. ^{1}H NMR (400 MHz, CD_3CN) δ 7.97-7.88 (m, 3H), 7.80-7.69 (m, 12H), 5.95 (dq, J_1 = 10.7 Hz, J_2 = 7.4 Hz, 1H), 2.59-2.39 (m, 4H), 1.82 (dd, J_1 = 16.6 Hz, J_2 = 7.4 Hz, 3H) ppm; ^{13}C NMR (100 MHz, CD_3CN) δ 177.7 (d, J = 1.1 Hz), 136.7 (d, J = 3.1 Hz), 135.6 (d, J = 9.9 Hz), 131.4 (d, J = 12.8 Hz), 117.5 (d, J = 83.6 Hz), 45.4 (d, J = 55.6 Hz), 28.6, 15.3 (d, J = 2.5 Hz) ppm; ^{31}P NMR (161.9 MHz, CD_3CN) δ 27.3 ppm; IR (ATR) 2918, 1778, 1705, 1387, 1049, 1036 cm^{-1}; HRMS (ESI-TOF) calcd for $C_{24}H_{23}NO_2P$ [M^+] 388.1466 found 388.1465.

1-(*N*-Succinimido)ethyltris(3-chlorophenyl)phosphonium tetrafluoroborate (5m). Resin (162.0 mg, 28% yield). ^{1}H NMR (400 MHz, CD_3CN) δ 8.00-7.89 (m, 3H), 7.85-7.60 (m, 9H), 6.01 (dq, J_1 = 10.2 Hz, J_2 = 7.4 Hz, 1H), 2.65-2.45 (m, 4H), 1.81 (dd, J_1 = 17.4 Hz, J_2 = 7.4 Hz, 3H) ppm; ^{13}C NMR (100 MHz, CD_3CN) δ 177.8 (d, J = 1.4 Hz), 137.3 (d, J = 17.0 Hz), 137.2 (d, J = 3.0 Hz), 134.9 (d, J = 11.4 Hz), 134.2 (d, J = 9.6 Hz), 133.3 (d, J = 14.1 Hz), 119.0 (d, J = 83.5 Hz), 45.1 (d, J = 54.8 Hz), 28.7, 15.3 (d, J = 2.6 Hz) ppm; ^{31}P NMR (161.9 MHz, CD_3CN) δ 27.7 ppm; IR (ATR) 3076, 1780, 1706, 1034 cm^{-1}; HRMS (ESI-TOF) calcd for $C_{24}H_{20}Cl_3NO_2P$ [M^+] 490.0297 found 490.0299.

1-(*N*-Succinimido)ethyltris(4-trifluoromethylphenyl)phosphonium tetrafluoroborate (5n). Colorless crystals (346.4 mg, 51% yield), mp 222.0-223.5°C. ^{1}H NMR (400 MHz, CD_3CN) δ 8.09-8.02 (m, 6H), 8.00-7.92 (m, 6H), 6.08 (dq, J_1 = 10.3 Hz, J_2 = 7.3 Hz, 1H), 2.68-2.43 (m, 4H), 1.85 (dd, J_1 = 17.5 Hz, J_2 = 7.3 Hz, 3H) ppm; ^{13}C NMR (100 MHz, CD_3CN) δ 177.7 (d, J = 1.5 Hz), 137.4 (qd, J_1 = 33.4 Hz, J_2 = 3.3 Hz), 136.9 (d, J = 10.7 Hz), 128.5-128.2 (m), 124.2 (qd, J_1 = 272.7 Hz, J_2 = 1.4 Hz), 121.4 (dq, J_1 = 83.1 Hz, J_2 = 1.1 Hz), 45.1 (d, J = 54.5 Hz), 28.8, 15.5 (d, J = 2.4 Hz) ppm; ^{31}P NMR

(161.9 MHz, CD_3CN) δ 27.8 ppm; ^{19}F NMR (376 MHz, CD_3CN) δ -64.3 (s, CF_3) ppm; IR (ATR) 3108, 1790, 1713, 1400, 1320, 1061, 1036 cm^{-1}; HRMS (ESI-TOF) calcd for $C_{27}H_{20}F_9NO_2P$ [M^+] 592.1088 found 592.1083.

1-Methoxyethyltriphenylphosphonium tetrafluoroborate[5] **(8).** Resin (298.0 mg, 73% yield). ^1H NMR (400 MHz, $CDCl_3$) δ 7.89-7.60 (m, 15H), 5.69 (dq, J_1 = 6.7 Hz, J_2 = 4.7 Hz, 1H), 3.56 (s, 3H), 1.66 (dd, J_1 = 18.2 Hz, J_2 = 6.7 Hz, 3H) ppm; ^{13}C NMR (100 MHz, $CDCl_3$) δ 135.3 (d, J = 3.0 Hz), 134.4 (d, J = 9.3 Hz), 130.5 (d, J = 12.3 Hz), 116.7 (d, J = 83.2 Hz), 73.0 (d, J = 68.2 Hz), 59.6 (d, J = 11.3 Hz), 15.1 (d, J = 3.2 Hz) ppm; ^{31}P NMR (161.9 MHz, $CDCl_3$) δ 17.9 ppm; IR (ATR) 3066, 2939, 1588, 1486, 1439, 1110, 1047, 996 cm^{-1}.

***N*-[1-(4-Methoxyphenyl)ethyl]phthalimide (9aa) and *N*-[1-(2-methoxyphenyl)ethyl]phthalimide (9ab).** A mixture of two isomers (25.6 mg, 91% yield). Isomers were separated by fractional crystallization from toluene. ***N*-[1-(4-Methoxyphenyl)ethyl]phthalimide (9aa).** Colorless crystals, mp 66.5-68.5°C. ^1H NMR (400 MHz, $CDCl_3$) δ 7.81-7.77 (m, 2H), 7.70-7.65 (m, 2H), 7.48-7.42 (m, 2H), 6.88-6.83 (m, 2H), 5.53 (q, J = 7.4 Hz, 1H), 3.77 (s, 3H), 1.90 (d, J = 7.3 Hz, 3H) ppm; ^{13}C NMR (100 MHz, $CDCl_3$) δ 168.2, 159.0, 133.8, 132.5, 132.0, 128.8, 123.1, 113.7, 55.2, 49.2, 17.7 ppm; IR (ATR) 2983, 1773, 1701, 1606, 1510, 1387, 1352, 1330, 1246, 1046 cm^{-1}; HRMS (ESI-TOF) calcd for $C_{17}H_{15}NO_3Na$ $[M + Na]^+$ 304.0950 found 304.0951.

***N*-[1-(2-Methoxyphenyl)ethyl]phthalimide (9ab).** Colorless crystals, mp 120-121°C. ^1H NMR (400 MHz, $CDCl_3$) δ 7.87-7.74 (m, 2H), 7.71-7.58 (m, 3H), 7.33-7.20 (m, 1H), 7.04-6.93 (m, 1H), 6.87-6.79 (m, 1H), 5.86 (q, J = 7.2 Hz, 1H), 3.75 (s, 3H), 1.83 (d, J = 7.2 Hz, 3H) ppm; ^{13}C NMR (100 MHz, $CDCl_3$) δ 168.0, 156.8, 133.6, 132.1, 128.8, 128.4, 127.8, 123.0, 120.2, 110.3, 55.4, 43.9, 17.3 ppm; IR (ATR) 2976, 1758, 1701, 1604, 1492, 1464, 1393, 1354, 1334, 1247 cm^{-1}; HRMS (ESI-TOF) calcd for $C_{17}H_{15}NO_3Na$ $[M + Na]^+$ 304.0950 found 304.0956.

***N*-[1-(4-Methoxyphenyl)-1-phenylmethyl]phthalimide (9ba) and *N*-[1-(2-methoxyphenyl)-1-phenylmethyl]phthalimide (9bb).** A mixture of two isomers (28.5 mg, 83% yield). Isomers were separated by column chromatography (toluene:ethyl acetate; 20:1, v/v). ***N*-[1-(4-Methoxyphenyl)-1-phenylmethyl]phthalimide (9ba).** Oil. ^1H NMR (400 MHz, $CDCl_3$) δ 7.90-7.81 (m, 2H), 7.75-7.67 (m, 2H), 7.40-7.30 (m, 7H), 6.90-6.83 (m, 2H), 6.67 (s, 1H), 3.79 (s, 3H) ppm; ^{13}C NMR (100 MHz, $CDCl_3$) δ 167.9, 159.1, 138.5, 134.0, 131.9, 130.3, 130.2, 128.3, 128.3, 127.5, 123.3, 113.7, 57.3, 55.2 ppm; IR (ATR) 3030, 2836, 1768, 1709, 1610, 1511, 1382, 1353, 1326, 1247 cm^{-1}; HRMS (ESI-TOF) calcd for $C_{22}H_{17}NO_3Na$ $[M + Na]^+$ 366.1106 found 366.1111. ***N*-[1-(2-Methoxyphenyl)-1-phenylmethyl]phthalimide (9bb).** Colorless crystals, mp 104-106°C. ^1H NMR (400 MHz, $CDCl_3$) δ 7.87-7.74 (m, 2H), 7.72-7.66 (m, 2H), 7.40-7.30 (m, 6H), 7.30-7.18 (m, 1H), 6.97 (s, 1H), 6.94-6.81 (m, 2H), 3.74 (s, 3H) ppm; ^{13}C NMR (100 MHz, $CDCl_3$) δ 168.1, 157.1, 138.4, 133.9, 132.0, 130.9, 129.1, 128.3, 128.3, 127.4, 126.5, 123.3, 120.1, 110.2, 55.6, 52.6 ppm; IR (ATR) 2934, 2836, 1764, 1704, 1601, 1492, 1464, 1438, 1386, 1353, 1329, 1249 cm^{-1}; HRMS (ESI-TOF) calcd for $C_{22}H_{17}NO_3Na$ $[M + Na]^+$ 366.1106 found 366.1108.

***N*-[1-(4-Methoxyphenyl)-3-methylbutyl]phthalimide (9ca)** and ***N*-[1-(2-methoxyphenyl)-3-methylbutyl]phthalimide (9cb).** A mixture of two isomers (28.5 mg, 88% yield). Attempts to separate isomers by fractional crystallization or column chromatography failed. Major *p*-isomer (**9ca**): ^{1}H NMR (400 MHz, $CDCl_3$) δ 7.85-7.73[a] (m, 2H), 7.71-7.62[a] (m, 2H), 7.54-7.44 (m, 2H), 6.88-6.80[a] (m, 2H), 5.40 (dd, J_1 = 10.0 Hz, J_2 = 6.5 Hz, 1H), 3.77 (s, 3H), 2.60-2.46[a] (m, 1H), 2.08-1.97 (m, 1H), 1.58-1.45[a] (m, 1H), 0.97[a] (d, J = 6.4 Hz, 3H), 0.96[a] (d, J = 6.8 Hz, 3H) ppm; ^{13}C NMR (100 MHz, $CDCl_3$) δ 168.4, 159.0, 133.8, 132.1, 131.9, 129.5, 123.1, 113.7, 55.2, 52.5, 39.9, 25.5, 22.9, 22.0 ppm. Minor *o*-isomer (**9cb**): ^{1}H NMR (400 MHz, $CDCl_3$) δ 7.85-7.73[a] (m, 2H), 7.71-7.62[a] (m, 3H), 7.26-7.19 (m, 1H), 6.99-6.92 (m, 1H), 6.88-6.80[a] (m, 1H), 5.87 (dd, J_1 = 10.5 Hz, J_2 = 5.4 Hz, 1H), 3.79 (s, 3H), 2.60-2.46[a] (m, 1H), 1.96-1.86 (m, 1H), 1.58-1.45[a] (m, 1H), 1.01 (d, J = 6.5 Hz, 3H), 0.95[a] (d, J = 6.8 Hz, 3H) ppm; ^{13}C NMR (100 MHz, $CDCl_3$) δ 168.3, 156.9, 133.6, 132.0, 128.9, 128.7, 127.8, 123.0, 120.2, 110.4, 55.5, 46.4, 39.7, 25.4, 23.2, 21.9 ppm. [a]Overlapping signals of two isomers.

***N*-[1-(4-Methoxyphenyl)ethyl]succinimide (9da) and *N*-[1-(2-methoxyphenyl)ethyl]succinimide (9db).** A mixture of two isomers (13.1 mg, 56% yield). Isomers were separated by column chromatography (toluene:ethyl acetate; 5:1, v/v). ***N*-[1-(4-Methoxyphenyl)ethyl]succinimide (9da).** Oil. ^{1}H NMR (400 MHz, $CDCl_3$) δ 7.46-7.35 (m, 2H), 6.92-6.80 (m, 2H), 5.38 (q, J = 7.4 Hz, 1H), 3.78 (s, 3H), 2.62 (s, 4H), 1.90 (d, J = 7.3 Hz, 3H) ppm; ^{13}C NMR (100 MHz, $CDCl_3$) δ 177.0, 159.1, 131.8, 129.0, 113.7, 55.2, 49.9, 28.1, 16.7 ppm; IR (ATR) 2919, 1698, 1612, 1514, 1390, 1361, 1249, 1189 cm^{-1}; HRMS (ESI-TOF) calcd for $C_{13}H_{15}NO_3Na$ $[M + Na]^+$ 256.0950 found 250.0949. ***N*-[1-(2-Methoxyphenyl)ethyl]succinimide (9db).** Colorless crystals, mp 95-97°C. ^{1}H NMR (400 MHz, $CDCl_3$) δ 7.60-7.53 (m, 1H), 7.30-7.21 (m, 1H), 7.01-6.93 (m, 1H), 6.87-6.80 (m, 1H), 5.68 (q, J = 7.3 Hz, 1H), 3.78 (s, 3H), 2.61 (s, 4H), 1.72 (d, J = 7.3 Hz, 3H) ppm; ^{13}C NMR (100 MHz, $CDCl_3$) δ 176.7, 156.8, 128.8, 128.6, 127.0, 120.1, 110.2, 55.4, 44.5, 28.0, 16.2 ppm; IR (ATR) 2943, 1687, 1600, 1495, 1392, 1355, 1295, 1248, 1208, 1191, 1178 cm^{-1}; HRMS (ESI-TOF) calcd for $C_{13}H_{15}NO_3Na$ $[M + Na]^+$ 256.0950 found 256.0955.

***N*-[1-(2,4-Dimethoxyphenyl)ethyl]phthalimide (9e).** Colorless crystals (25.5 mg, 82% yield), mp 129.5-131.5°C. ^{1}H NMR (400 MHz, $CDCl_3$) δ 7.77 (dd, J_1 = 5.4 Hz, J_2 = 3.1 Hz, 2H), 7.66 (dd, J_1 = 5.4 Hz, J_2 = 3.0 Hz, 2H), 7.57 (d, J = 8.5 Hz, 1H), 6.51 (dd, J_1 = 8.5 Hz, J_2 = 2.4 Hz, 1H), 6.39 (d, J = 2.4 Hz, 1H), 5.79 (q, J = 7.3 Hz, 1H), 3.79 (s, 3H), 3.72 (s, 3H), 1.81 (d, J = 7.3 Hz, 3H) ppm; ^{13}C NMR (100 MHz, $CDCl_3$) δ 168.0, 160.4, 157.9, 133.6, 132.1, 129.1, 122.9, 120.2, 103.6, 98.3, 55.4, 55.3, 43.4, 17.3 ppm; IR (ATR) 2976, 1771, 1699, 1608, 1463, 1387, 1272, 1116, 1031 cm^{-1}; HRMS (ESI-TOF) calcd for $C_{18}H_{17}NO_4Na$ $[M + Na]^+$ 334.1055 found 334.1058.

***N*-[1-(2,4-Dimethoxyphenyl)-1-phenylmethyl]phthalimide (9f).** Colorless crystals (22.4 mg, 60% yield), mp 134.5-136.5°C. ^{1}H NMR (400 MHz, $CDCl_3$) δ 7.84-7.78 (m, 2H), 7.73-7.66 (m, 2H), 7.39-7.27 (m, 5H), 7.15-7.10 (m, 1H), 6.92 (s, 1H), 6.46-6.39 (m, 2H), 3.78 (s, 3H), 3.72 (s, 3H) ppm; ^{13}C NMR (100 MHz, $CDCl_3$) δ 168.2, 160.5, 158.1, 138.7, 133.8,

132.0, 131.8, 128.3, 128.1, 127.3, 123.2, 119.0, 103.7, 98.1, 55.6, 55.3, 52.2 ppm; IR (ATR) 2933, 1764, 1708, 1614, 1589, 1387, 1356, 1207, 1109, 1062 cm^{-1}; HRMS (ESI-TOF) calcd for $C_{23}H_{19}NO_4Na$ $[M^+]$ 396.1212 found 396.1218.

***N*-[1-(2,4-Dimethoxyphenyl)-3-methylbutyl]phthalimide (9g).** Colorless crystals (26.9 mg, 76% yield), mp 85.5-87.5°C. ^1H NMR (400 MHz, $CDCl_3$) δ 7.82-7.73 (m, 2H), 7.70-7.64 (m, 2H), 7.61 (d, J = 8.6 Hz, 1H), 6.49 (dd, J_1 = 8.6 Hz, J_2 = 2.5 Hz, 1H), 6.39 (d, J = 2.5 Hz, 1H), 5.79 (dd, J_1 = 10.3 Hz, J_2 = 5.8 Hz, 1H), 3.78 (s, 3H), 3.75 (s, 3H), 2.46 (ddd, J_1 = 13.7 Hz, J_2 = 10.3 Hz, J_3 = 5.2 Hz, 1H), 1.91 (ddd, J_1 = 14.2 Hz, J_2 = 8.5 Hz, J_3 = 5.6 Hz, 1H), 1.60-1.41 (m, 1H), 1.01 (d, J = 6.6 Hz, 3H), 0.95 (d, J = 6.6 Hz, 3H) ppm; ^{13}C NMR (100 MHz, $CDCl_3$) δ 168.4, 160.2, 158.1, 133.6, 132.1, 129.7, 123.0, 120.1, 103.7, 98.3, 55.5, 55.3, 45.9, 39.9, 25.4, 23.1, 22.0 ppm; IR (ATR) 2962, 1767, 1708, 1614, 1586, 1385, 1126, 1031 cm^{-1}; HRMS (ESI-TOF) calcd for $C_{21}H_{23}NO_4Na$ $[M + Na]^+$ 376.1525 found 376.1525.

***N*-[1-(4-Methylphenyl)ethyl]phthalimide (9ha)** and ***N*-[1-(2-methylphenyl)ethyl]phthalimide[6,7] (9hb).** A mixture of two isomers (13.3 mg, 50% yield). Attempts to separate isomers by fractional crystallization or column chromatography failed. Major *p*-isomer (**9ha**): ^1H NMR (400 MHz, $CDCl_3$) δ 7.83-7.70[a] (m, 2H), 7.72-7.64[a] (m, 2H), 7.43-7.36 (m, 2H), 7.17-7.10 (m, 2H), 5.54 (q, J = 7.3 Hz, 1H), 2.31 (s, 3H), 1.91 (d, J = 7.3 Hz, 3H) ppm; ^{13}C NMR (100 MHz, $CDCl_3$) δ 168.2, 137.4, 137.3, 133.8, 132.0, 129.1, 127.3, 123.1, 49.4, 21.0, 17.5 ppm. Minor *o*-isomer (**9hb**): ^1H NMR (400 MHz, $CDCl_3$) δ 7.83-7.70[a] (m, 2H), 7.72-7.64[a] (m, 2H), 7.32-7.04[a] (m, 4H), 5.72 (q, J = 7.9 Hz, 1H), 2.37 (s, 3H), 1.88 (d, J = 7.3 Hz, 3H) ppm; ^{13}C NMR (100 MHz, $CDCl_3$) δ 168.1, 138.1, 135.9, 133.8, 131.9, 130.4, 128.0, 127.7, 125.9, 124.4, 46.8, 19.5, 18.2 ppm. [a]Overlapping signals of two isomers.

***N*-[1-(4-Methylphenyl)-1-phenylmethyl]phthalimide (9ia)** and ***N*-[1-(2-methylphenyl)-1-phenylmethyl]phthalimide (9ib).** A mixture of two isomers (19.6 mg, 60% yield). The major isomer was isolated by column chromatography (toluene:ethyl acetate; 20:1, v/v) and crystallization from toluene. ***N*-[1-(4-Methylphenyl)-1-phenylmethyl]phthalimide (9ia).** Colorless crystals, mp 150.5-151.5°C. ^1H NMR (400 MHz, $CDCl_3$) δ 7.87-7.73 (m, 2H), 7.72-7.63 (m, 2H), 7.37-7.24 (m, 7H), 7.17-7.05 (m, 2H), 6.65 (s, 1H), 2.30 (s, 3H) ppm; ^{13}C NMR (100 MHz, $CDCl_3$) δ 168.0, 138.4, 137.4, 135.3, 134.0, 131.9, 129.1, 128.7, 128.6, 128.3, 127.6, 123.4, 57.6, 21.1 ppm; IR (ATR) 3033, 2921, 1769, 1713, 1514, 1382, 1354, 1328 cm^{-1}; HRMS (ESI-TOF) calcd for $C_{22}H_{17}NO_2Na$ $[M + Na]^+$ 350.1157 found 350.1156.

1-(2,4,6-Trimethoxyphenyl)ethyltris(4-trifluoromethylphenyl)phosphonium tetrafluoroborate (10). Colorless crystals (273.1 mg, 73% yield), mp 146.0-148.0°C. ^1H NMR (400 MHz, $CDCl_3$) δ 7.96-7.92 (m, 6H), 7.76-7.70 (m, 6H), 6.00 (s, 2H), 5.60-5.40 (m, 1H), 3.82 (s, 3H), 3.35 (br s, 6H), 1.94 (dd, J_1 = 14.4 Hz, J_2 = 8.1 Hz, 3H) ppm; ^{13}C NMR (100 MHz, $CDCl_3$) δ 162.9 (d, J = 3.1 Hz), 159.3, 136.8 (qd, J_1 = 33.6 Hz, J_2 = 3.0 Hz), 135.0 (d, J = 10.0 Hz), 127.0 (dq, J_1 = 12.3 Hz, J_2 = 3.8 Hz), 122.8 (q, J = 274.8 Hz), 122.1 (d, J = 81.6 Hz), 99.8 (d, J = 5.3 Hz), 91.0 (d, J = 2.3 Hz), 55.6, 55.2, 29.4 (d, J = 43.6 Hz), 14.4 ppm; ^{31}P NMR (161.9 MHz, $CDCl_3$) δ 23.3 ppm; ^{19}F NMR (376 MHz, $CDCl_3$) δ -63.7 (s, CF_3) ppm; IR (ATR) 2944, 1608, 1591, 1321, 1127, 1062 cm^{-1}; HRMS (ESI-TOF) calcd for $C_{32}H_{27}F_9O_3P$ $[M^+]$ 661.1554 found 661.1555.

References

[1] Jia, H-Z.; Zhang, P.; Sum, Y-R.; Li, Y. *Chin. J. Org. Chem.*, **2006,** *26*, 99-102.

[2] Reger, D.L.; Horger, J.J.; Debreczeni, A.; Smith, M.D. *Inorg. Chem.*, **2011**, *50*, 10225–10240.

[3] Thomas, H.G.; Kassel, S. *Chem. Ber.*, **1988**, *121*, 1575-1578.

[4] Heβ, U.; Gross, T.; Thiele, R. *Zeitschrift für Chemie*, **1979**, *11*, 195-196.

[5] Okada, H.; Mori, T.; Saikawa, Y.; Nakata, M. *Tetrahedron Lett.*, **2009**, *50*, 1276-1278.

[6] Chavan, S.P.; Bhanage, B.M. *Eur. J. Org. Chem.* **2015**, 2405–2410.

[7] Yang, Q.; Gao, W.; Deng, J.; Zhang, X. *Tetrahedron Lett.* **2006**, *47*, 821–823.

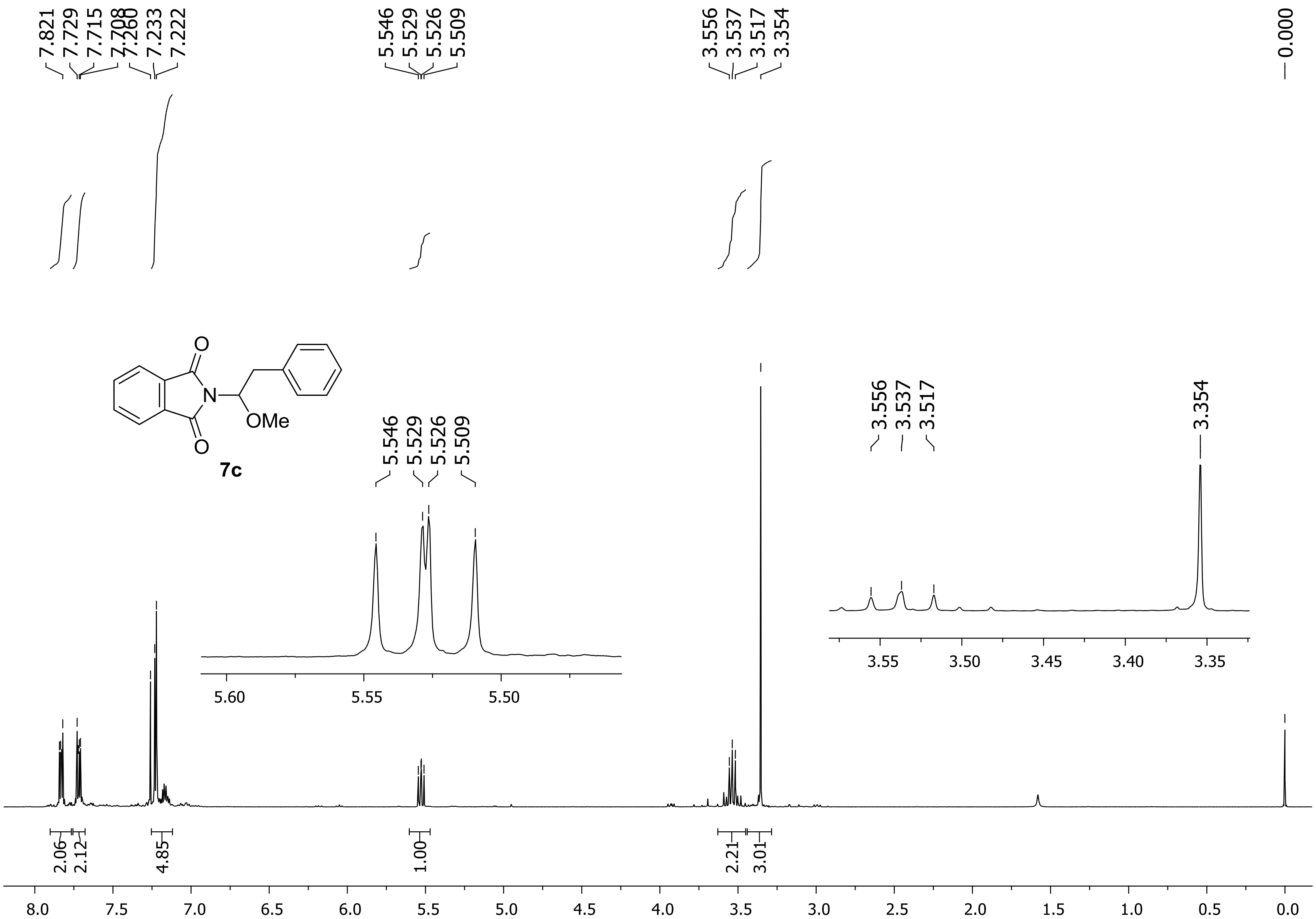

^{1}H NMR spectrum of *N*-(1-methoxy-2-phenylethyl)phthalimide (**7c**); 400 MHz/$CDCl_3$/TMS; δ (ppm).

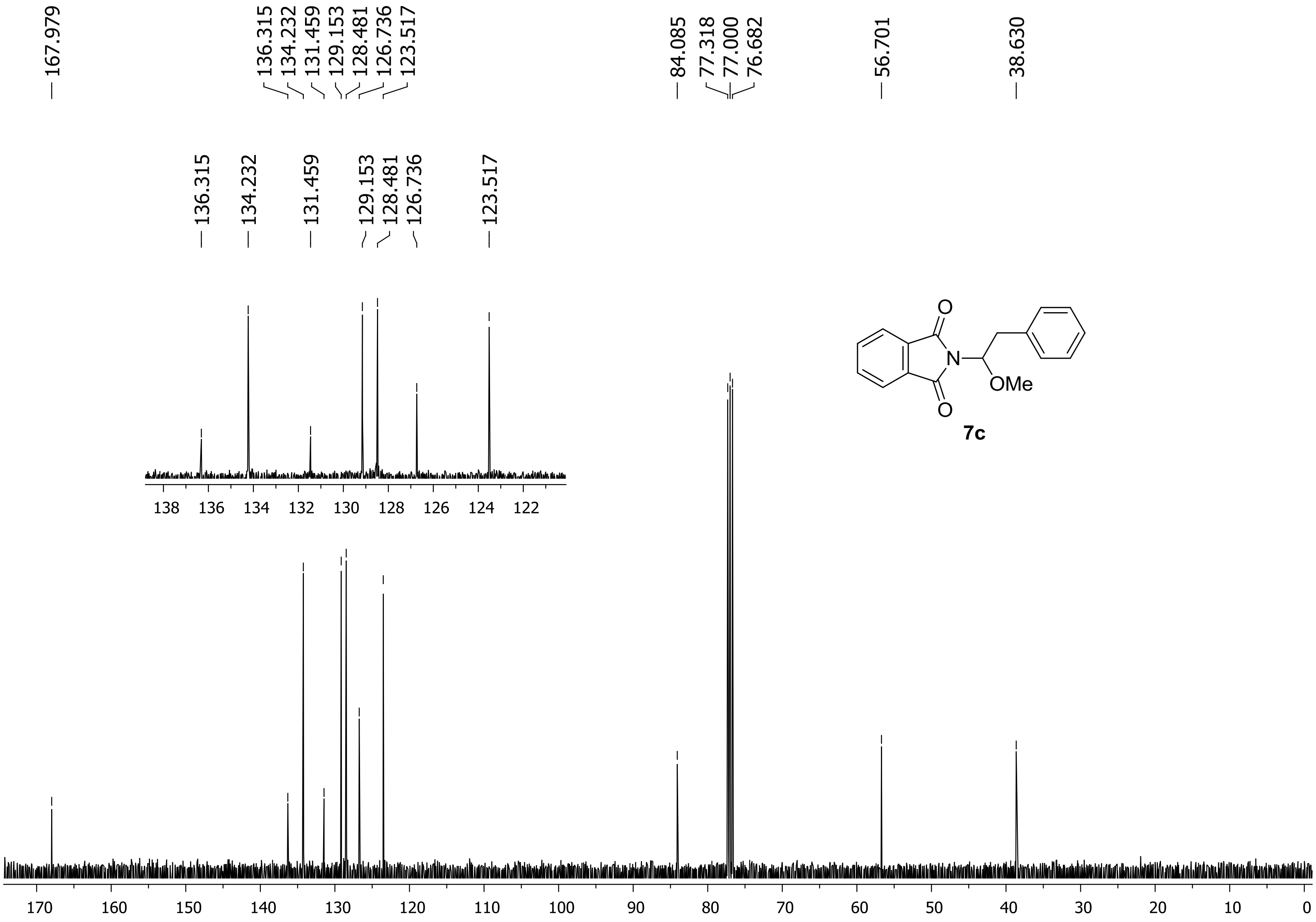

^{13}C NMR spectrum of *N*-(1-methoxy-2-phenylethyl)phthalimide (**7c**); 100 MHz/$CDCl_3$/TMS; δ (ppm).

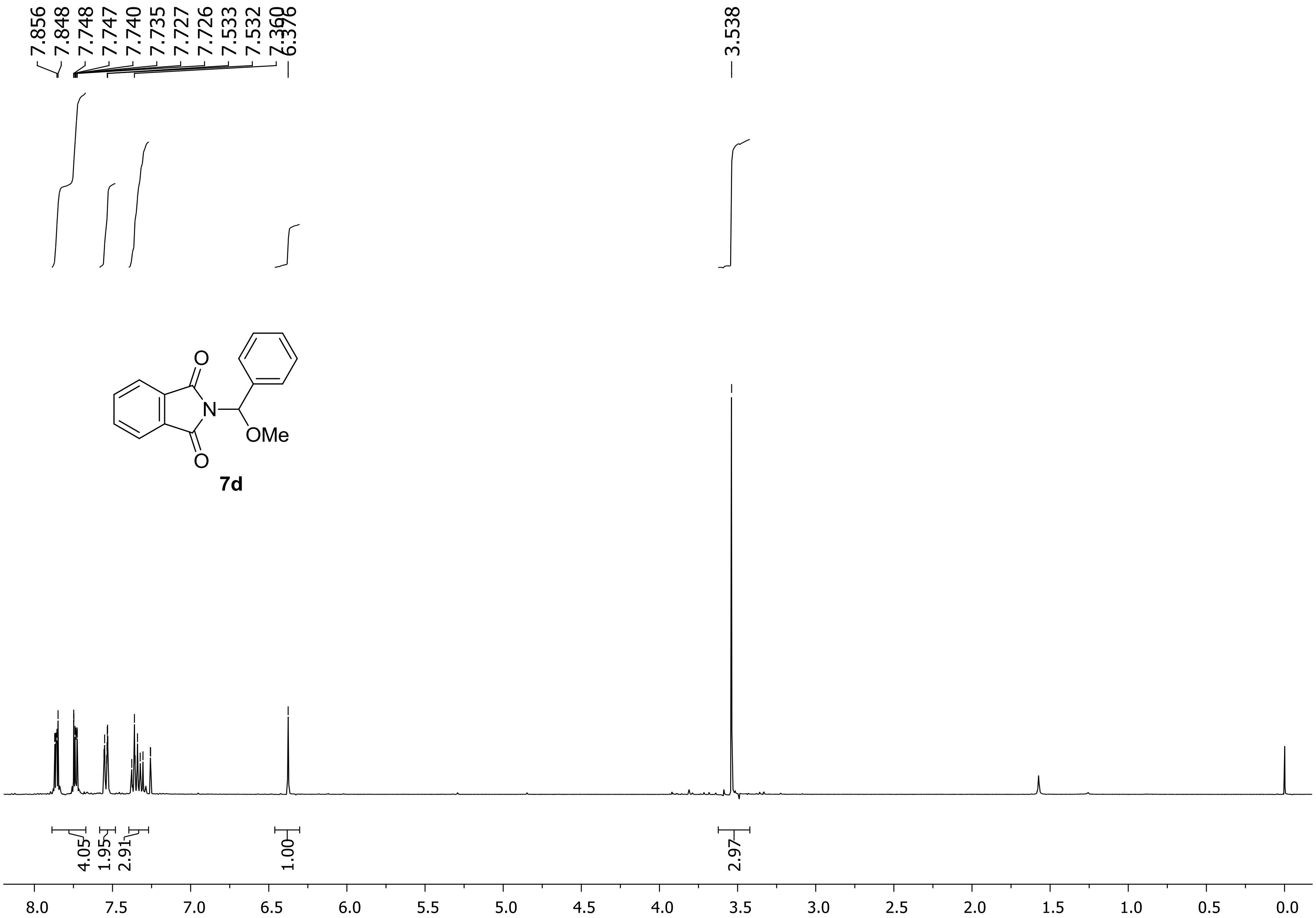

^{1}H NMR spectrum of *N*-(1-methoxy-1-phenylmethyl)phthalimide (**7d**); 400 MHz/$CDCl_3$/TMS; δ (ppm).

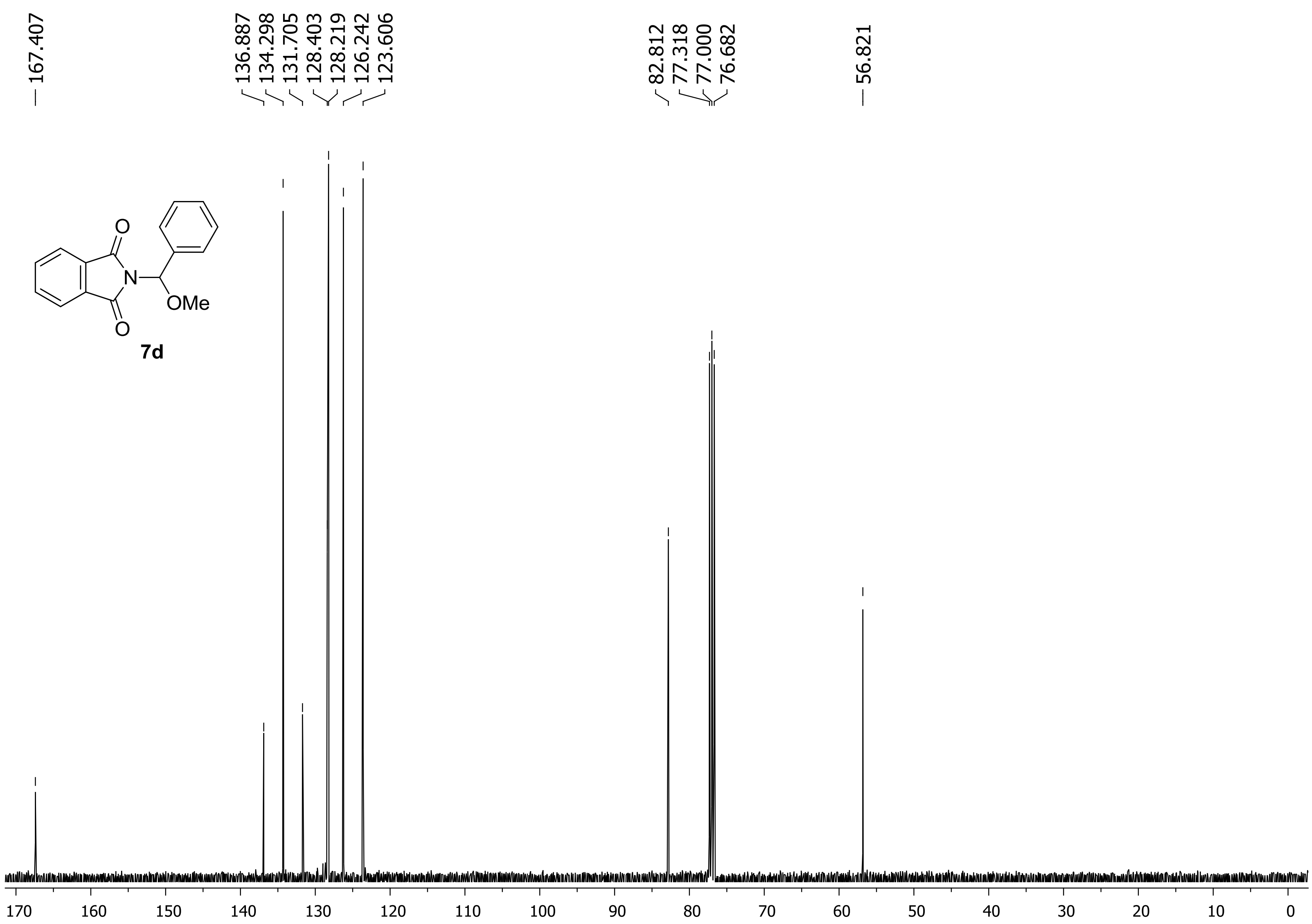

^{13}C NMR spectrum of *N*-(1-methoxy-1-phenylmethyl)phthalimide (**7d**); 100 MHz/$CDCl_3$/TMS; δ (ppm).

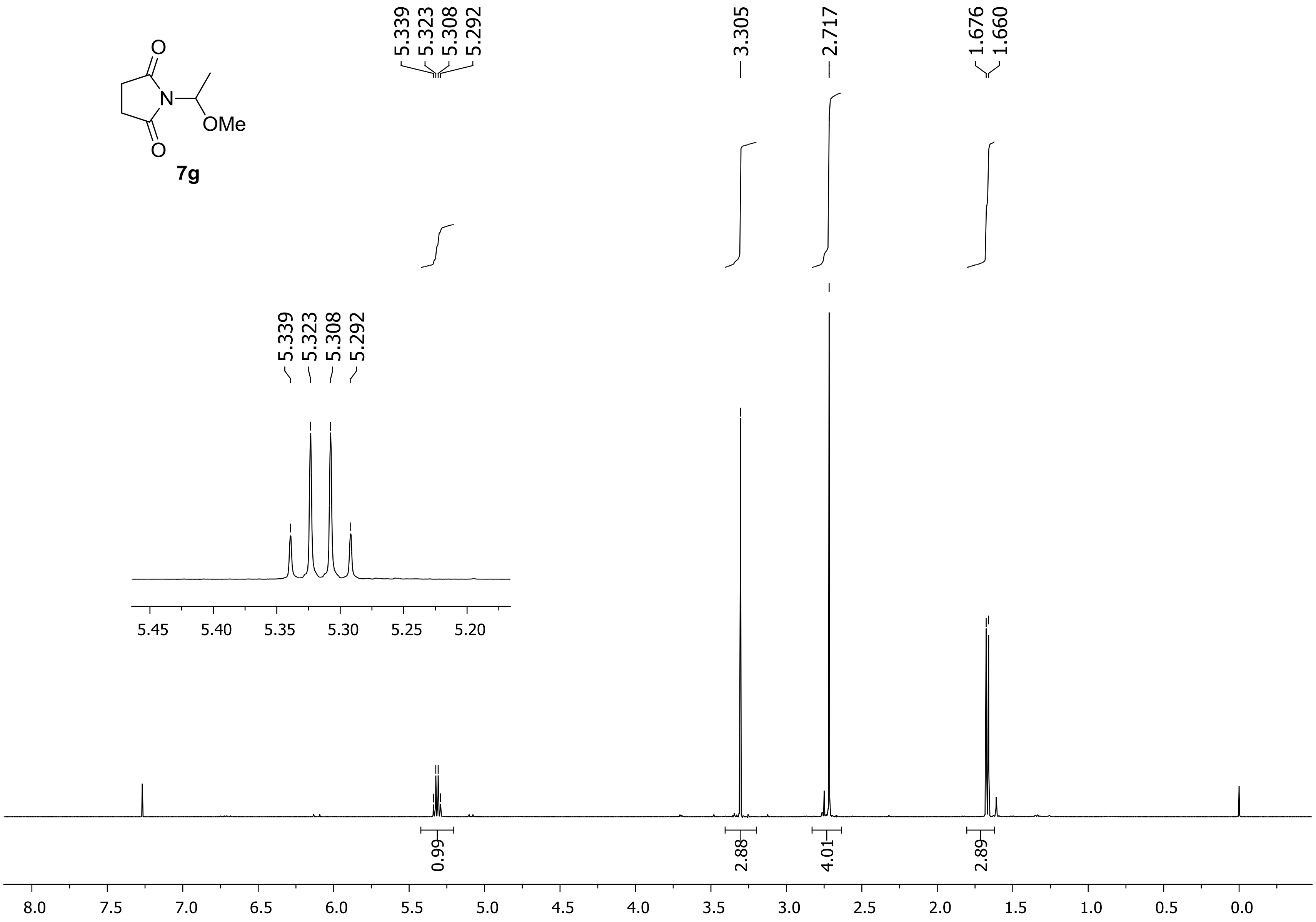

^{1}H NMR spectrum of *N*-(1-methoxyethyl)succinimide (**7g**); 400 MHz/$CDCl_3$/TMS; δ (ppm).

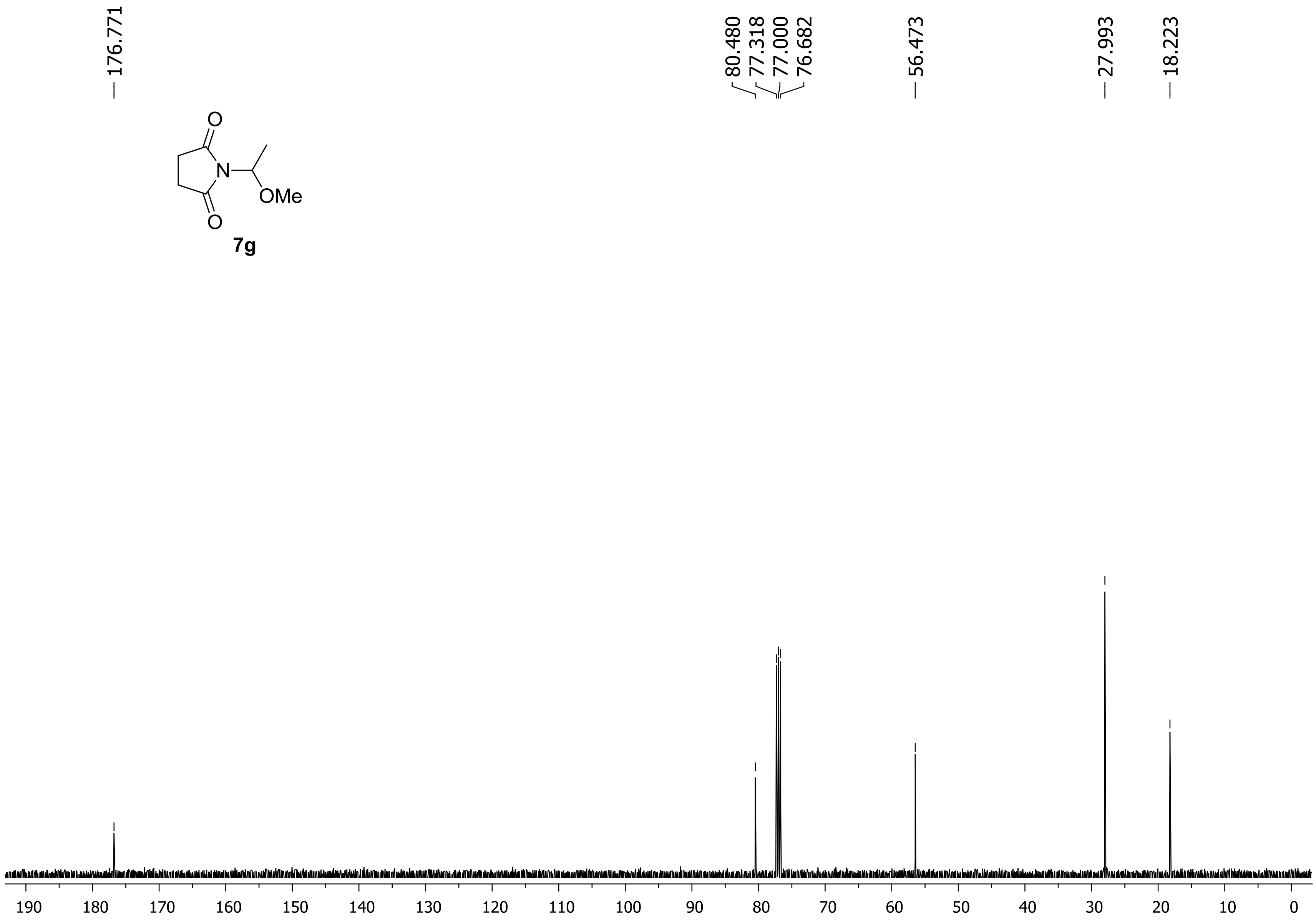

^{13}C NMR spectrum of *N*-(1-methoxyethyl)succinimide (**7g**); 100 MHz/$CDCl_3$/TMS; δ (ppm).

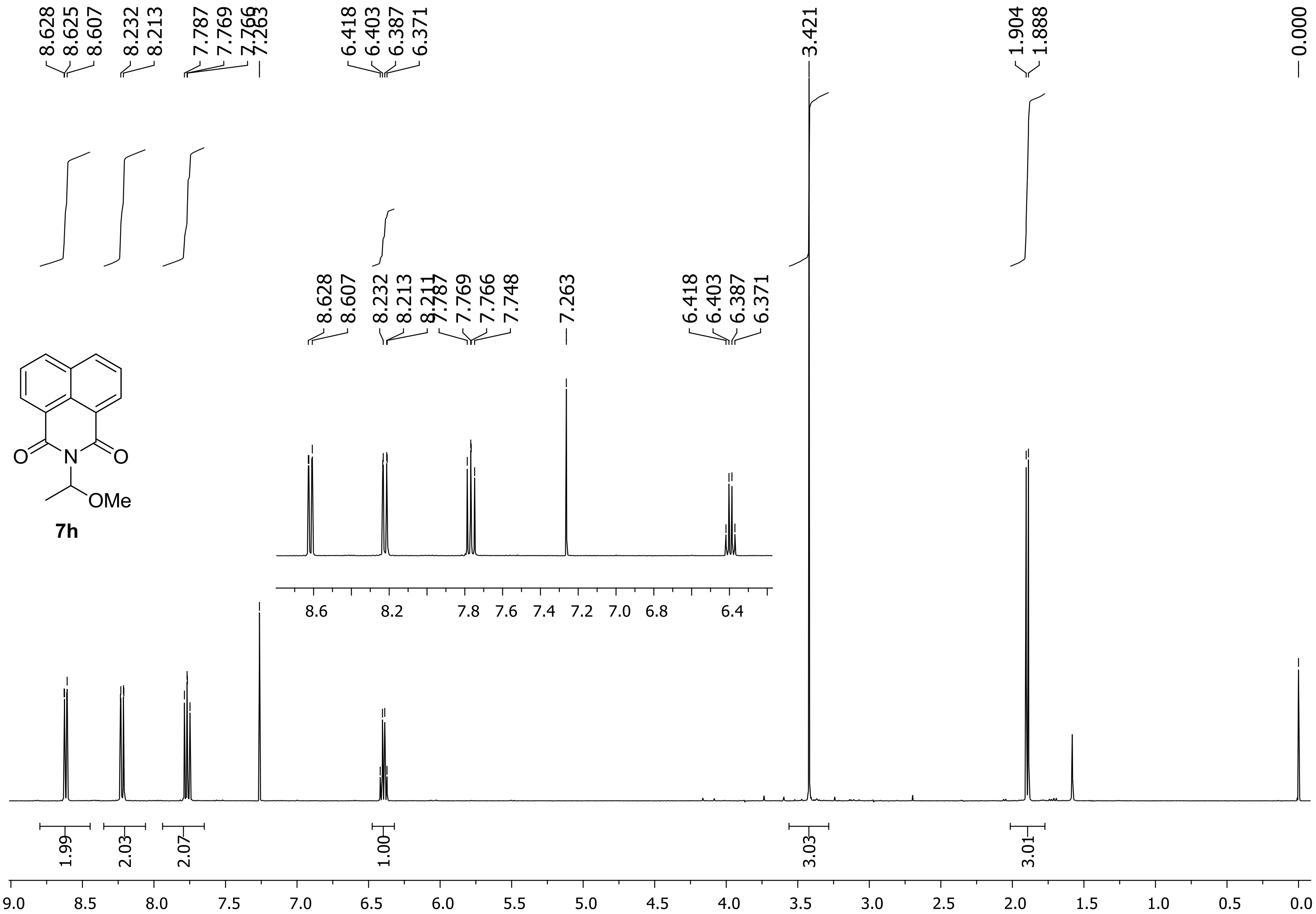

^{1}H NMR spectrum of *N*-(1-methoxyethyl)-1,8-naphthalimide (**7h**); 400 MHz/$CDCl_3$/TMS; δ (ppm).

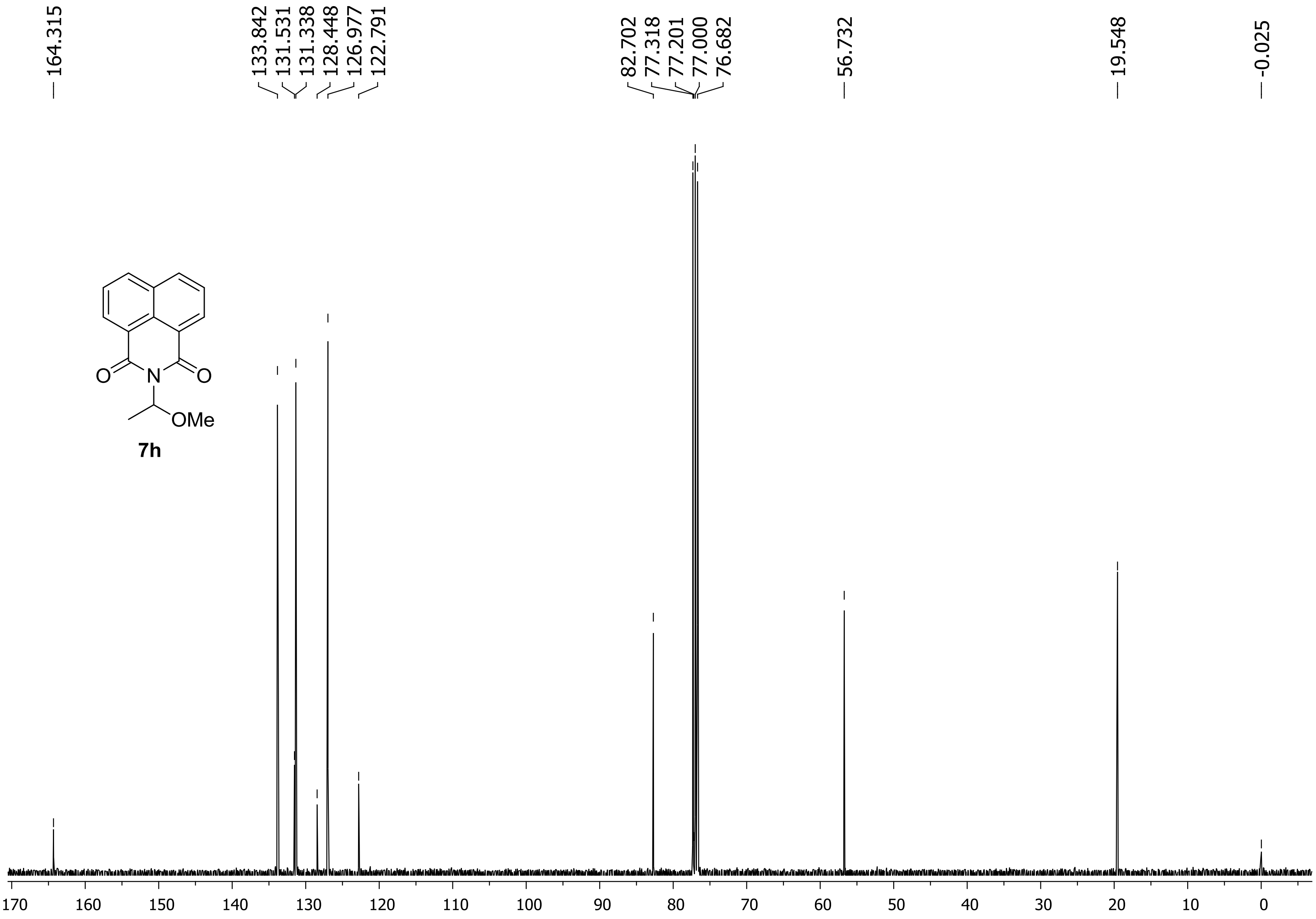

^{13}C NMR spectrum of *N*-(1-methoxyethyl)-1,8-naphthalimide (**7h**); 100 MHz/$CDCl_3$/TMS; δ (ppm).

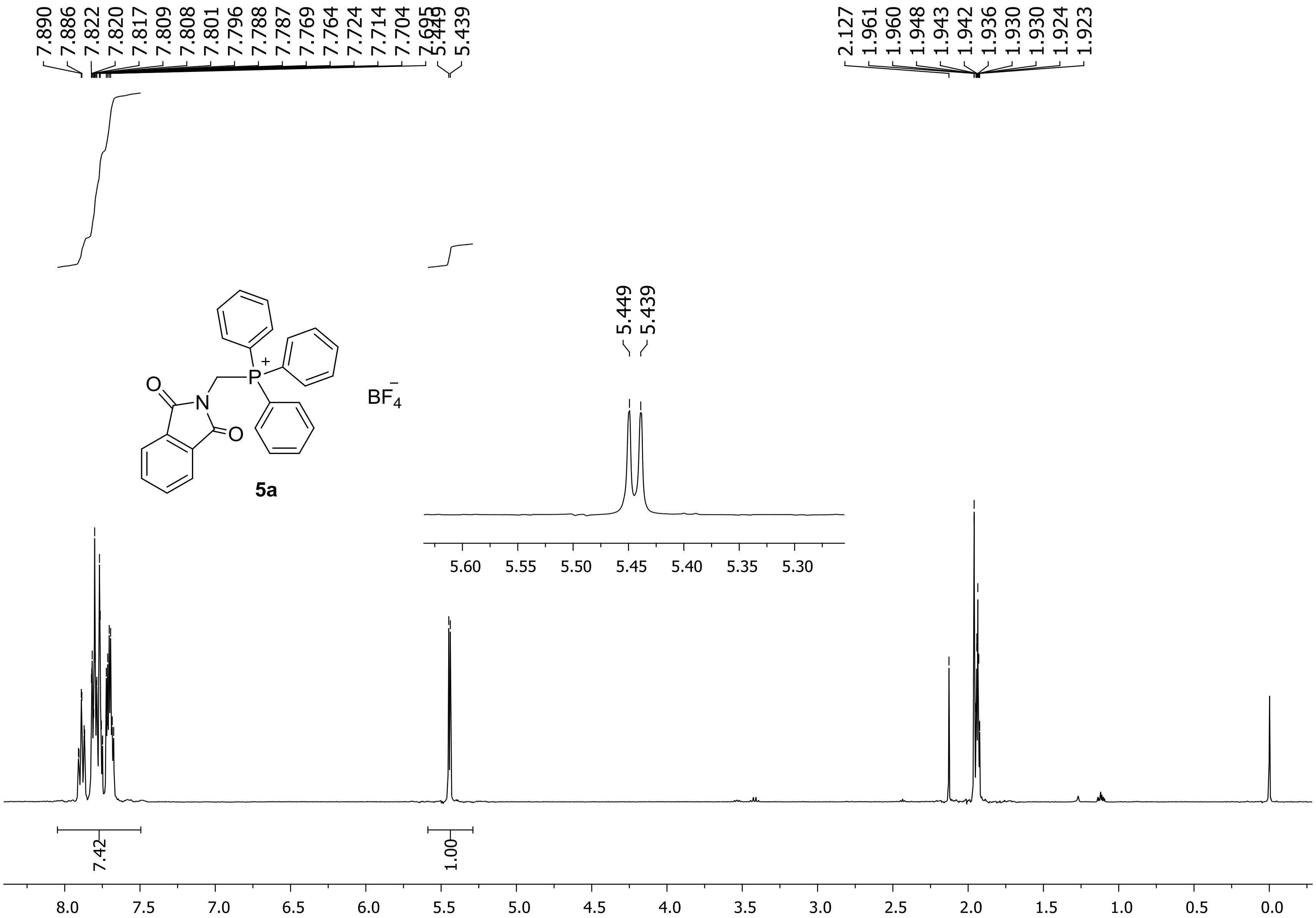

^{1}H NMR spectrum of 1-(*N*-phthalimido)methyltriphenylphosphonium tetrafluoroborate (**5a**); 400 MHz/CD_3CN/TMS; δ (ppm).

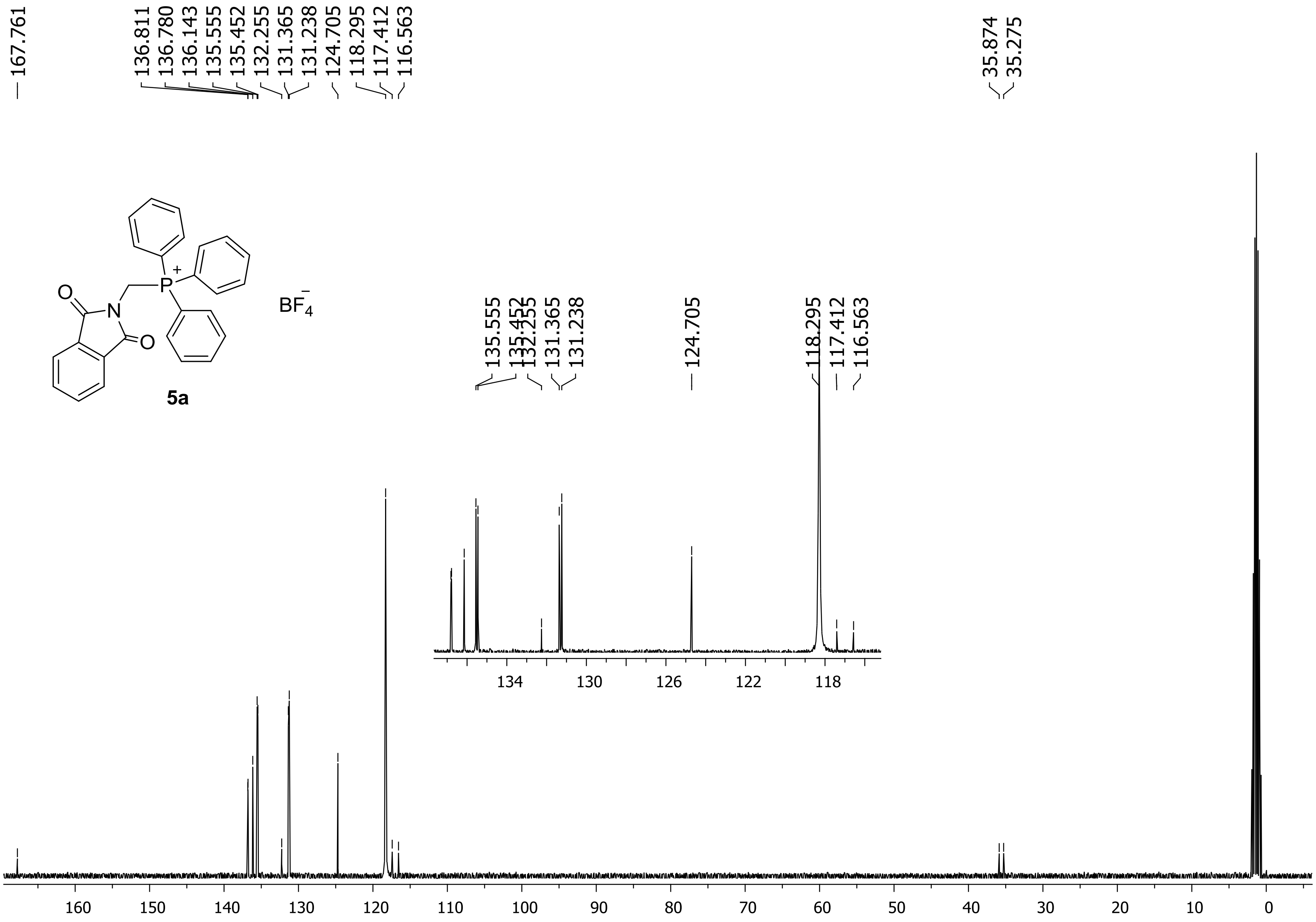

^{13}C NMR spectrum of 1-(*N*-phthalimido)methyltriphenylphosphonium tetrafluoroborate (**5a**); 100 MHz/CD_3CN/TMS; δ (ppm).

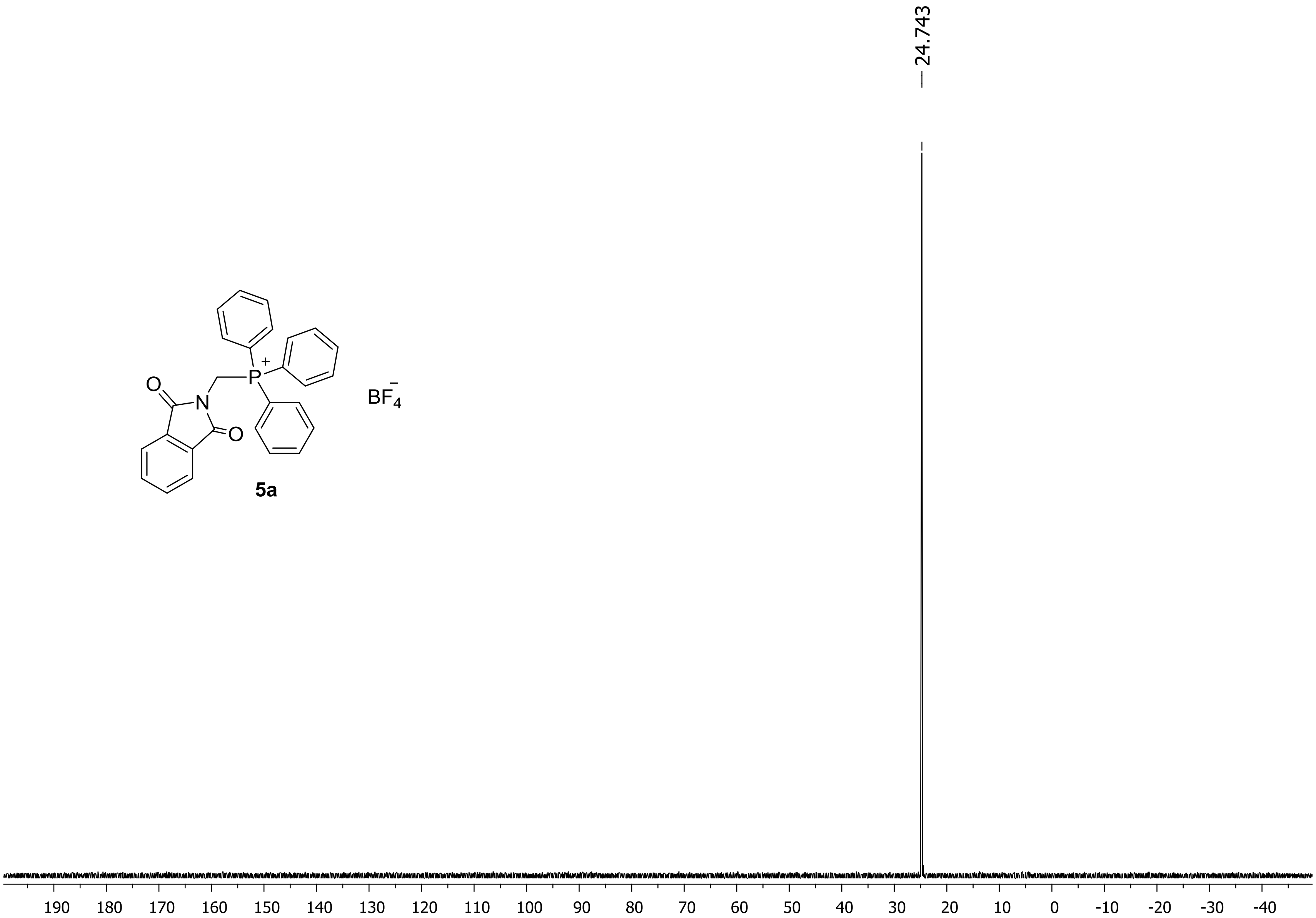

^{31}P NMR spectrum of 1-(*N*-phthalimido)methyltriphenylphosphonium tetrafluoroborate (**5a**); 161.9 MHz/CD_3CN; δ (ppm).

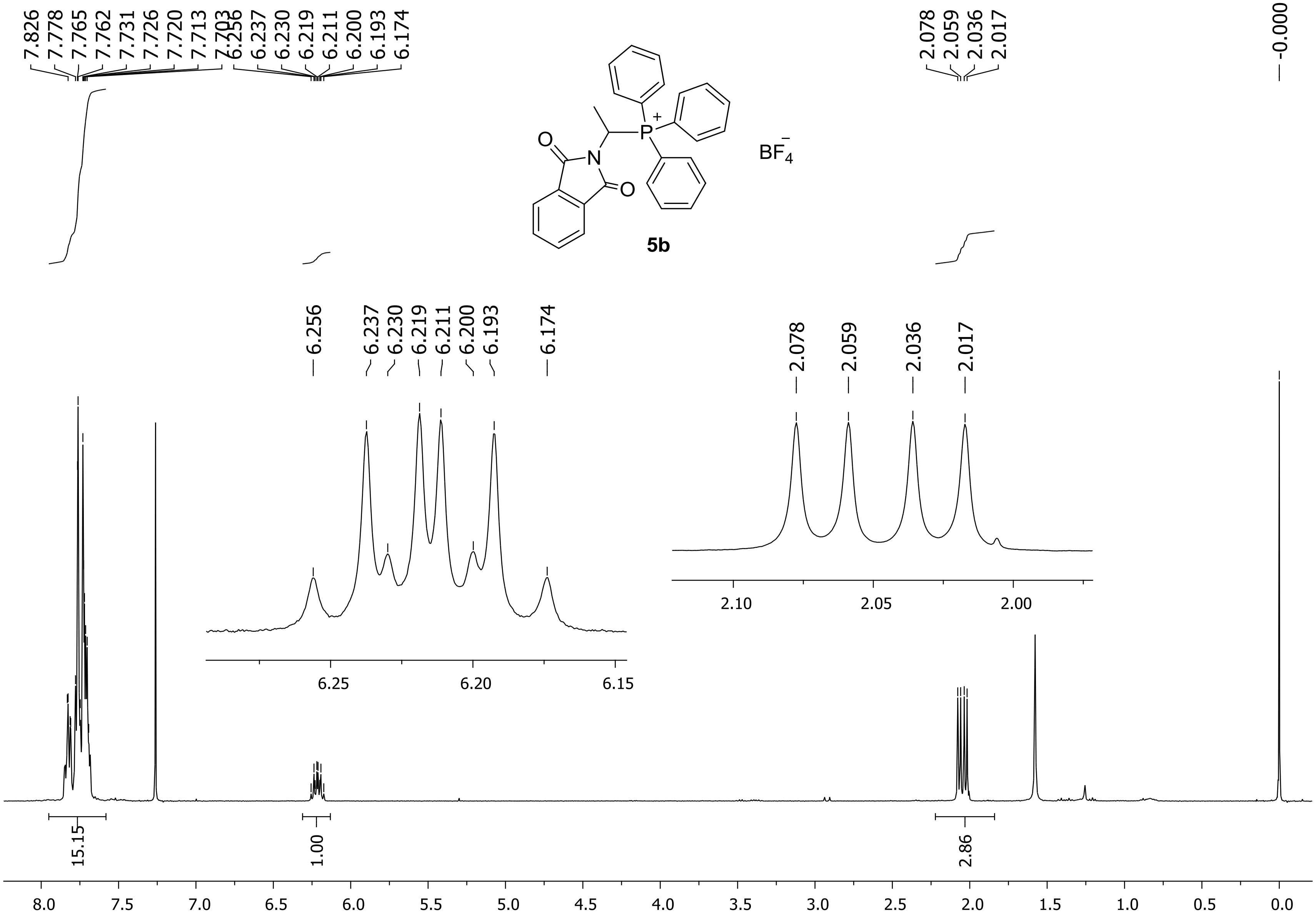

^{1}H NMR spectrum of 1-(*N*-phthalimido)ethyltriphenylphosphonium tetrafluoroborate (**5b**); 400 MHz/$CDCl_3$/TMS; δ (ppm).

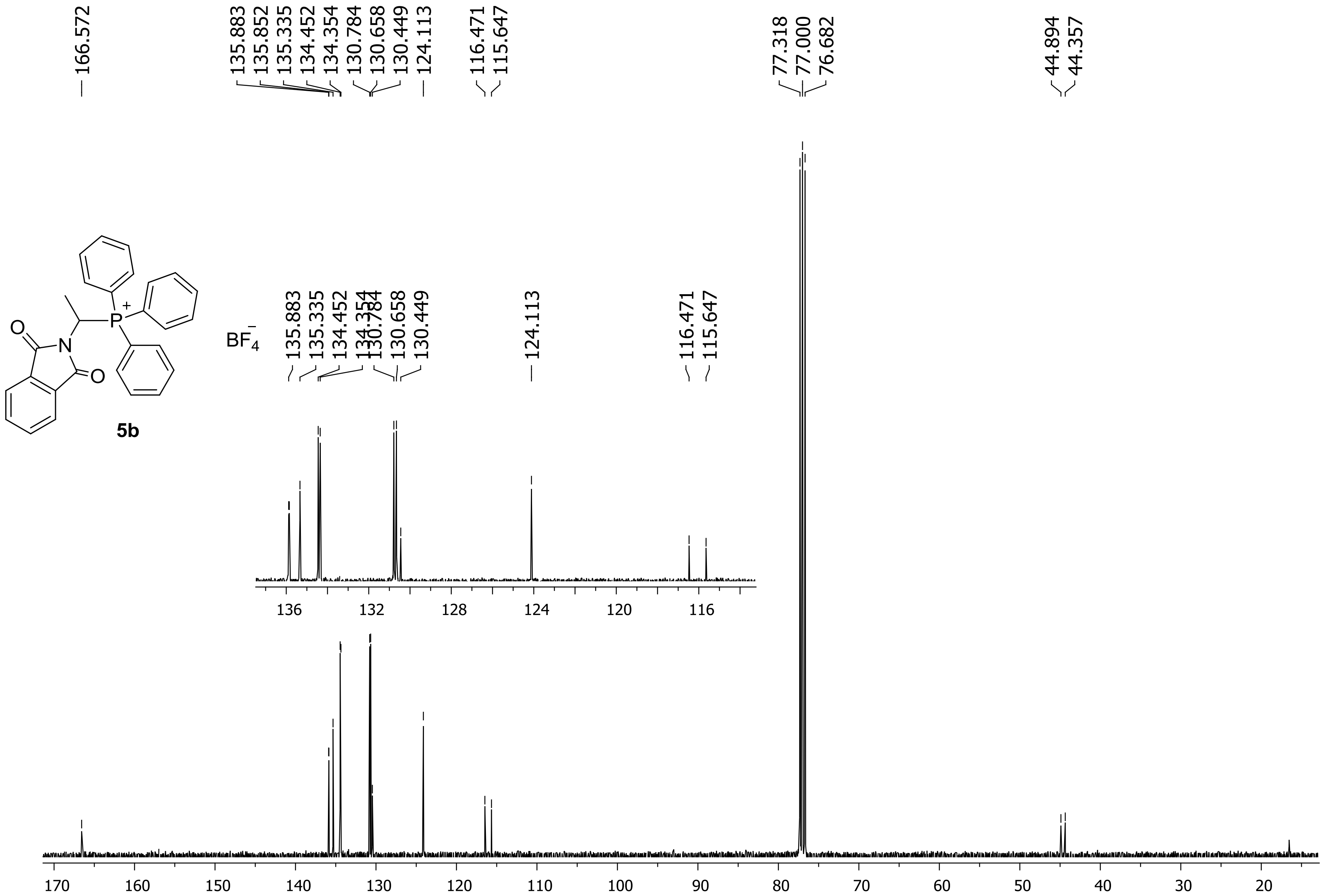

^{13}C NMR spectrum of 1-(*N*-phthalimido)ethyltriphenylphosphonium tetrafluoroborate (**5b**); 100 MHz/$CDCl_3$/TMS; δ (ppm).

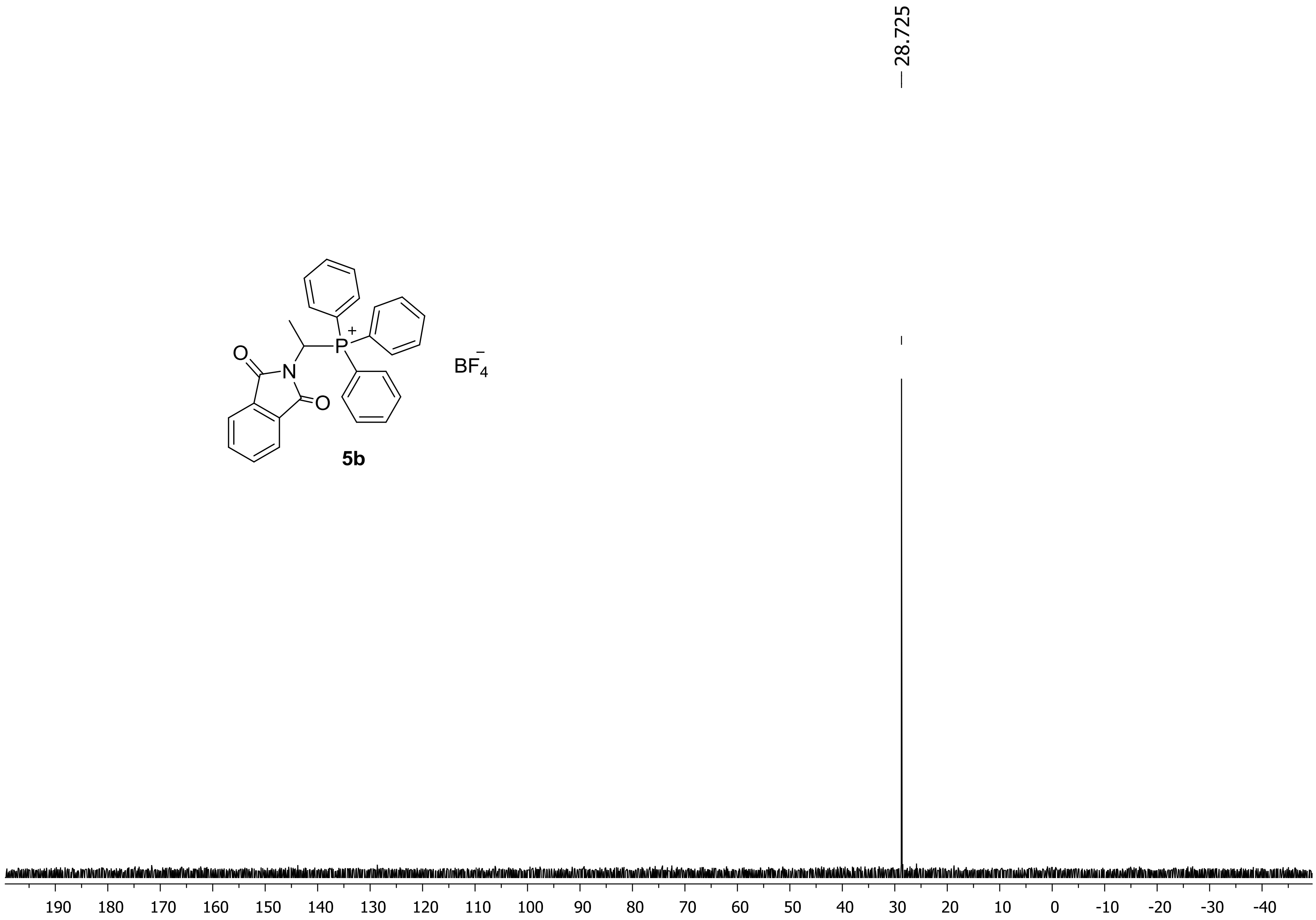

^{31}P NMR spectrum of 1-(*N*-phthalimido)ethyltriphenylphosphonium tetrafluoroborate (**5b**); 161.9 MHz/$CDCl_3$; δ (ppm).

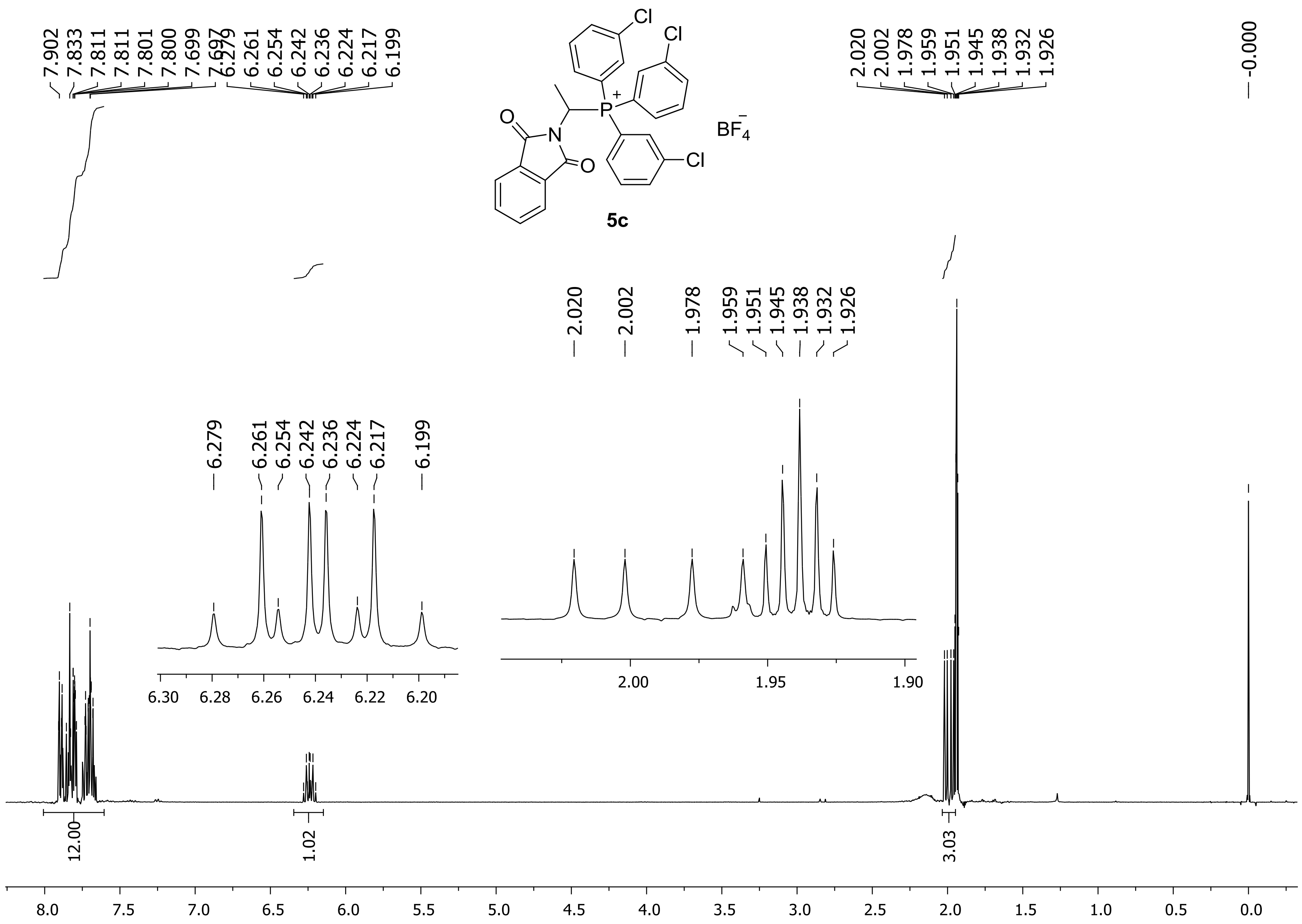

^{1}H NMR spectrum of 1-(*N*-phthalimido)ethyltris(3-chlorophenyl)phosphonium tetrafluoroborate (**5c**); 400 MHz/CD_3CN/TMS; δ (ppm).

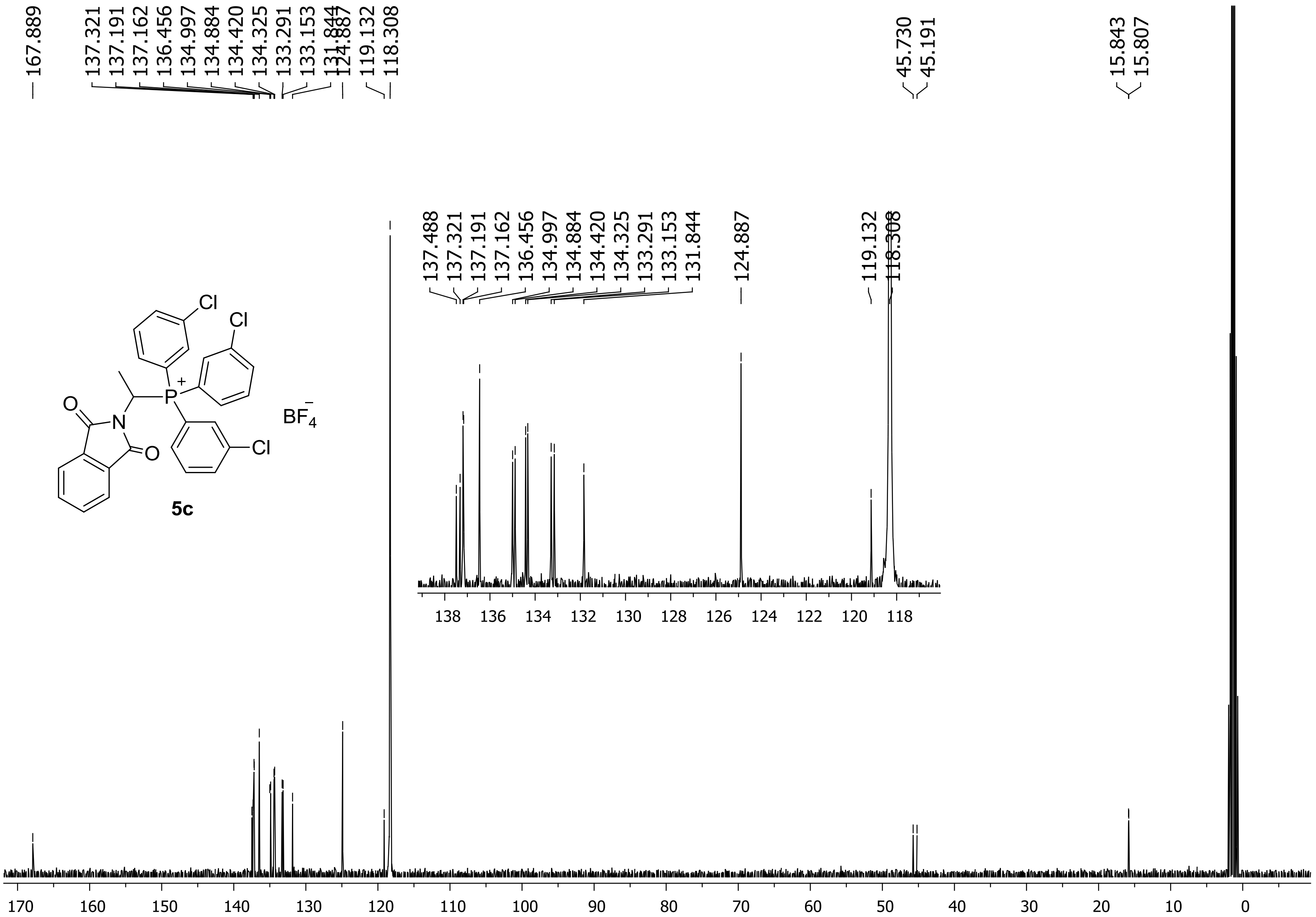

^{13}C NMR spectrum of 1-(*N*-phthalimido)ethyltris(3-chlorophenyl)phosphonium tetrafluoroborate (**5c**); 100 MHz/CD_3CN/TMS; δ (ppm).

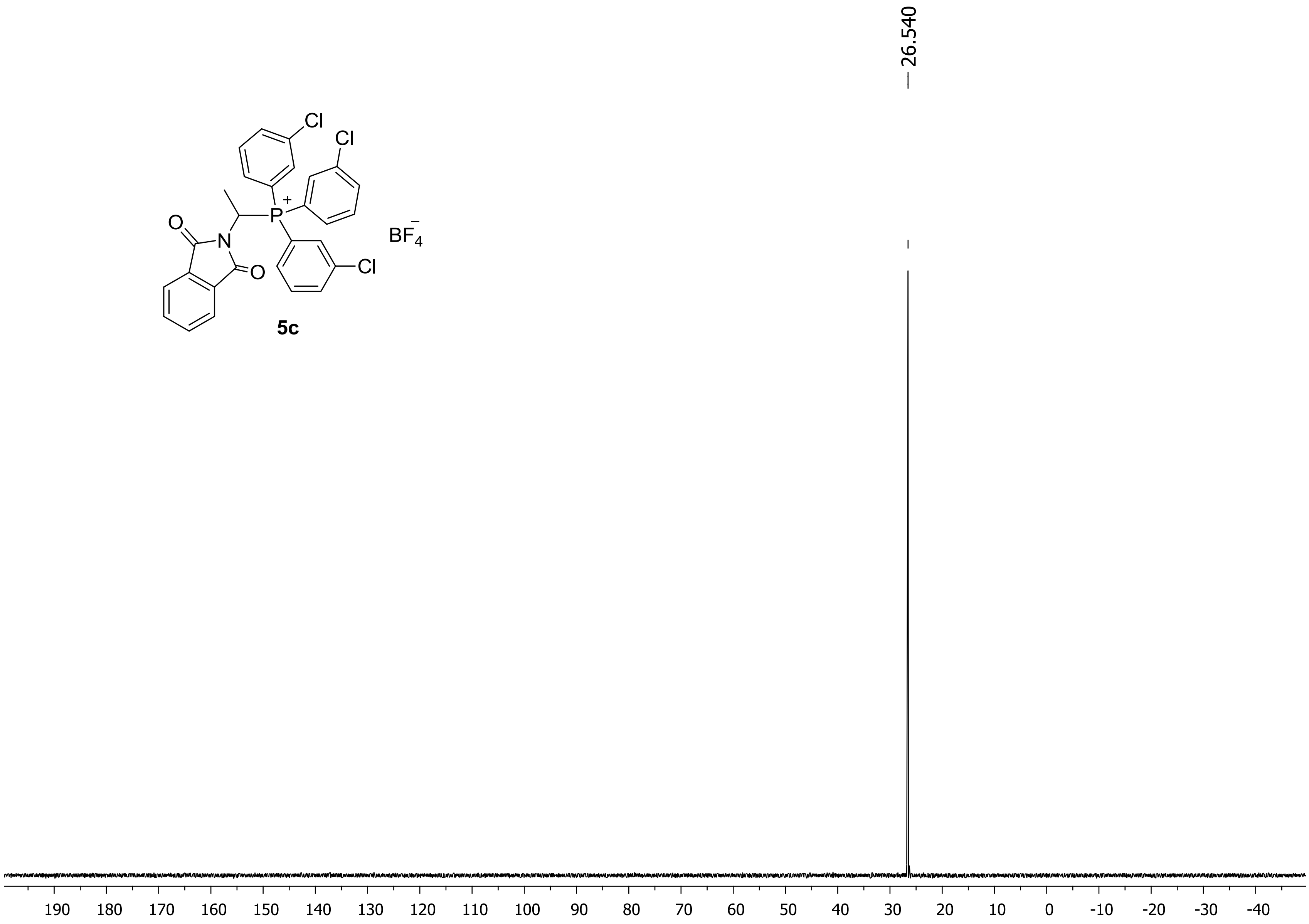

^{31}P NMR spectrum of 1-(*N*-phthalimido)ethyltris(3-chlorophenyl)phosphonium tetrafluoroborate (**5c**); 161.9 MHz/CD_3CN; δ (ppm).

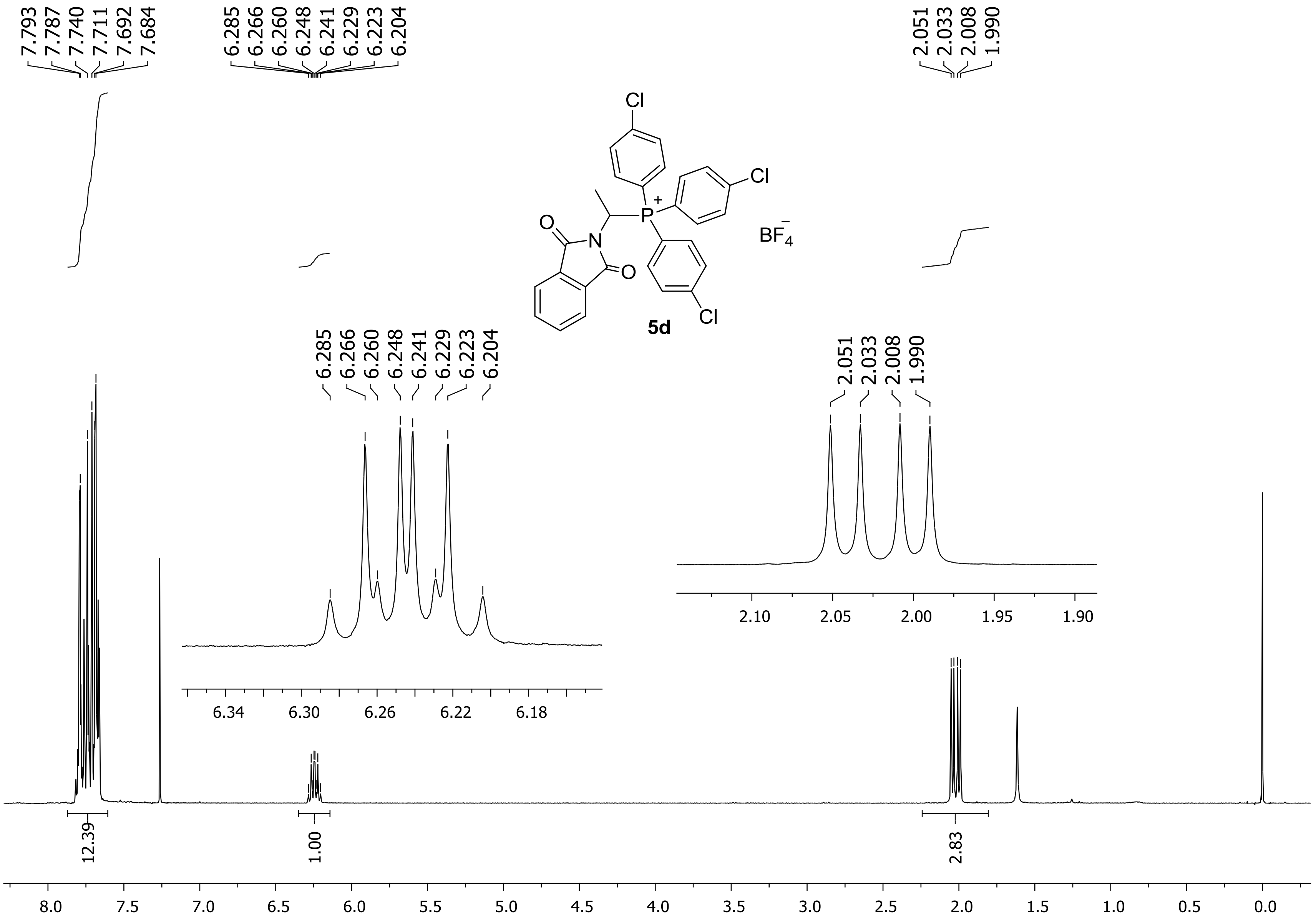

^{1}H NMR spectrum of 1-(*N*-phthalimido)ethyltris(4-chlorophenyl)phosphonium tetrafluoroborate (**5d**); 400 MHz/$CDCl_3$/TMS; δ (ppm).

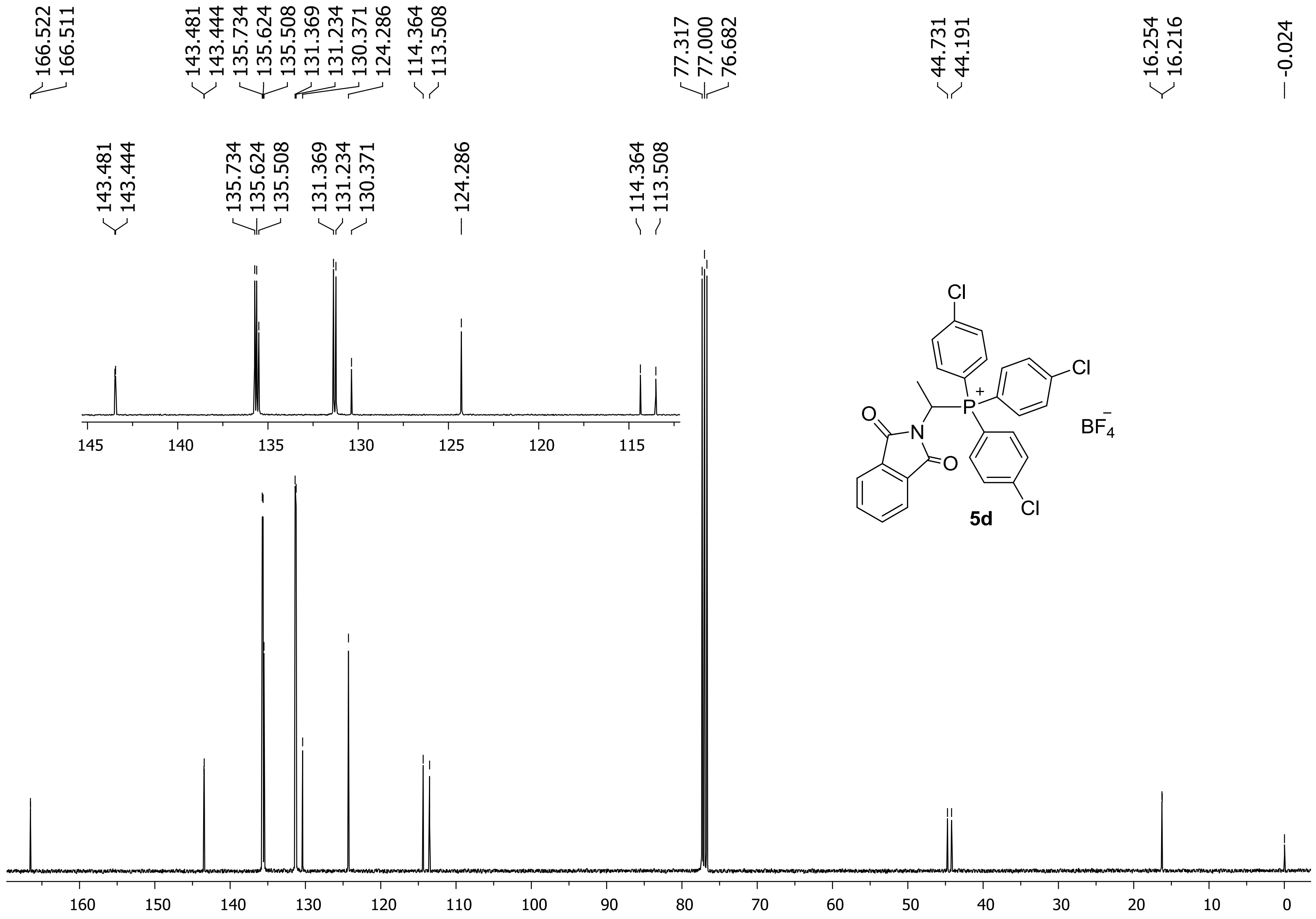

^{13}C NMR spectrum of 1-(*N*-phthalimido)ethyltris(4-chlorophenyl)phosphonium tetrafluoroborate (**5d**); 100 MHz/$CDCl_3$/TMS; δ (ppm).

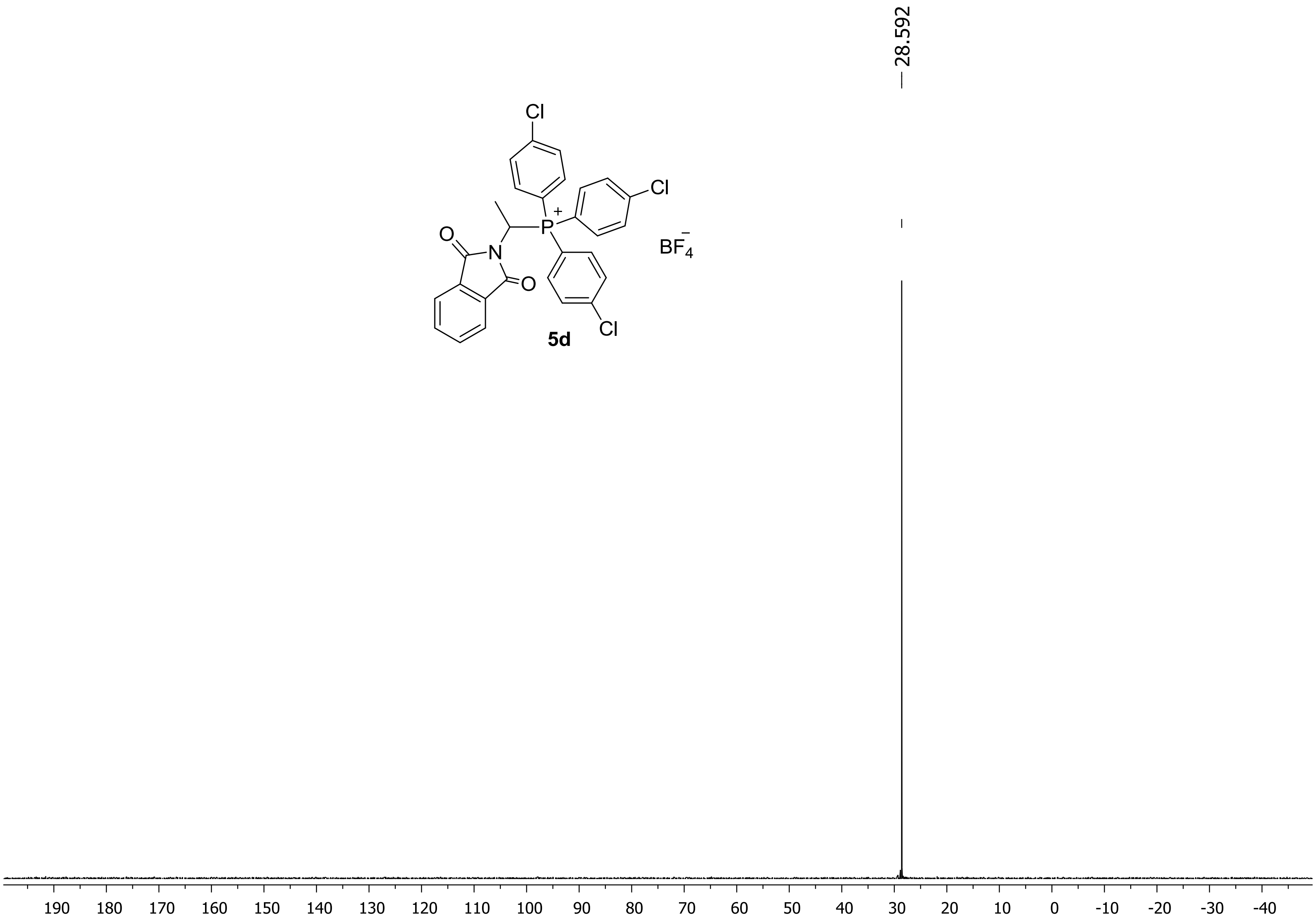

^{31}P NMR spectrum of 1-(*N*-phthalimido)ethyltris(4-chlorophenyl)phosphonium tetrafluoroborate (**5d**); 161.9 MHz/$CDCl_3$; δ (ppm).

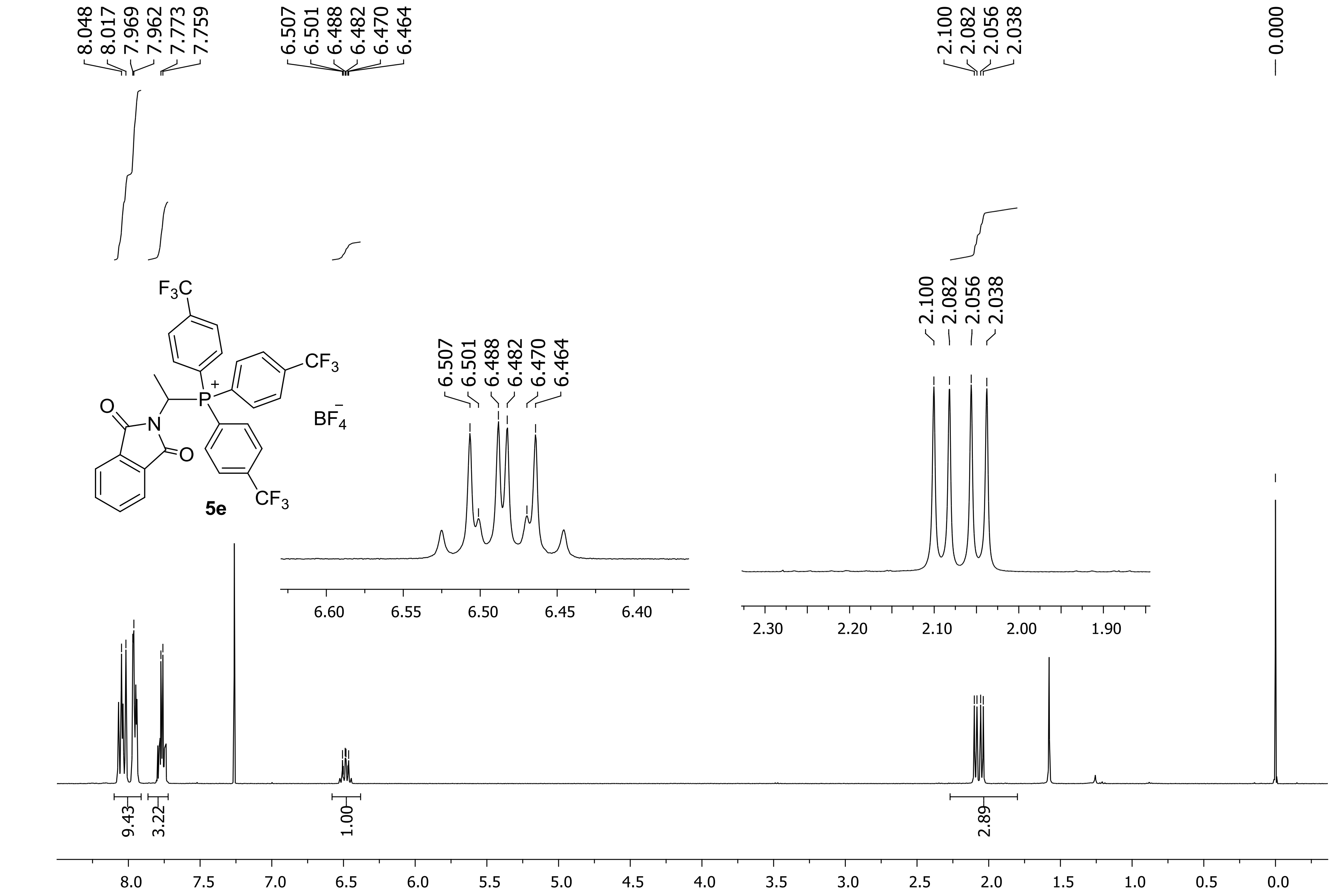

^{1}H NMR spectrum of 1-(*N*-phthalimido)ethyltris(4-trifluoromethylphenyl)phosphonium tetrafluoroborate (**5e**); 400 MHz/$CDCl_3$/TMS; δ (ppm).

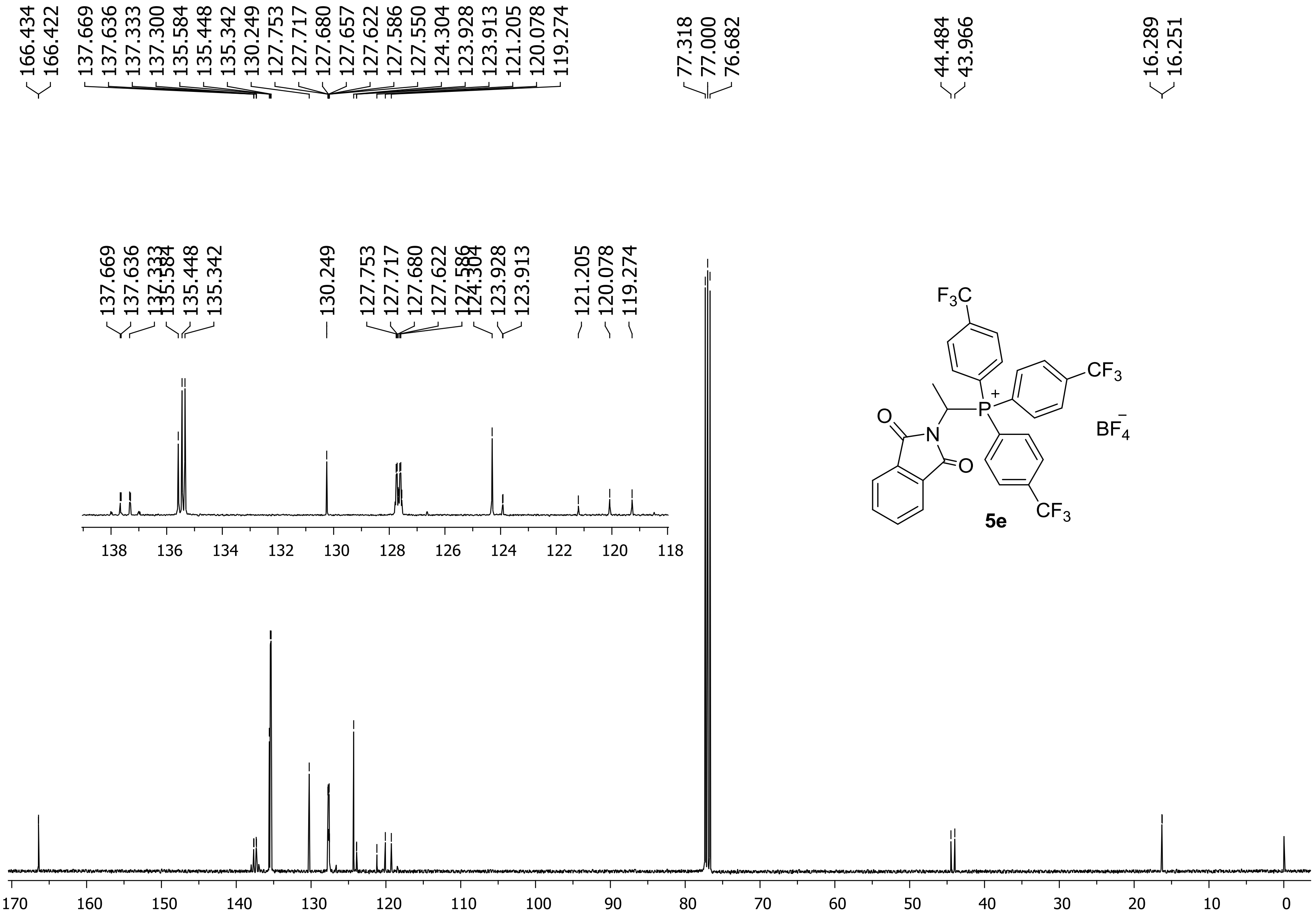

^{13}C NMR spectrum of 1-(*N*-phthalimido)ethyltris(4-trifluoromethylphenyl)phosphonium tetrafluoroborate (**5e**); 100 MHz/$CDCl_3$/TMS; δ (ppm).

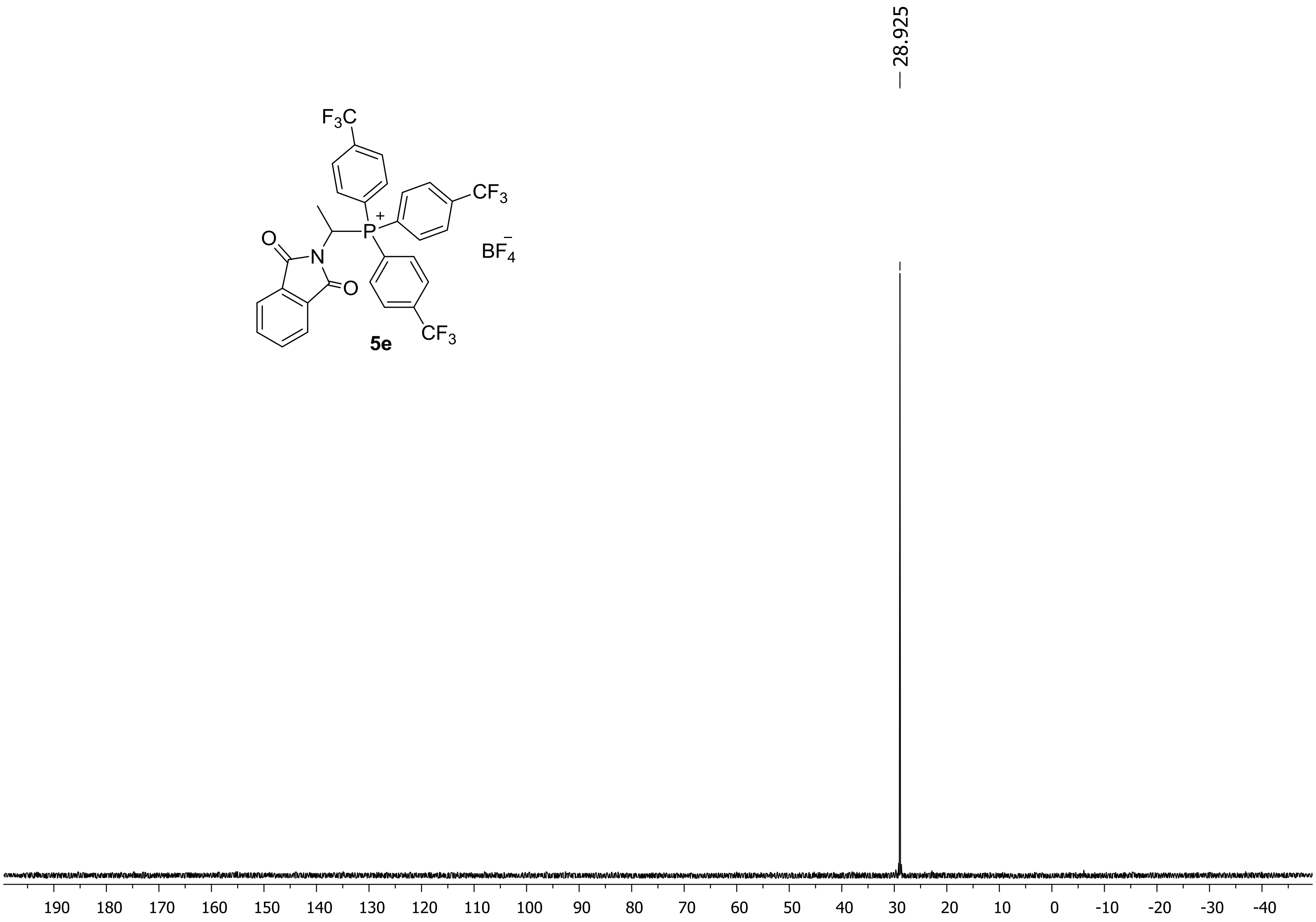

^{31}P NMR spectrum of 1-(*N*-phthalimido)ethyltris(4-trifluoromethylphenyl)phosphonium tetrafluoroborate (**5e**); 161.9 MHz/$CDCl_3$; δ (ppm).

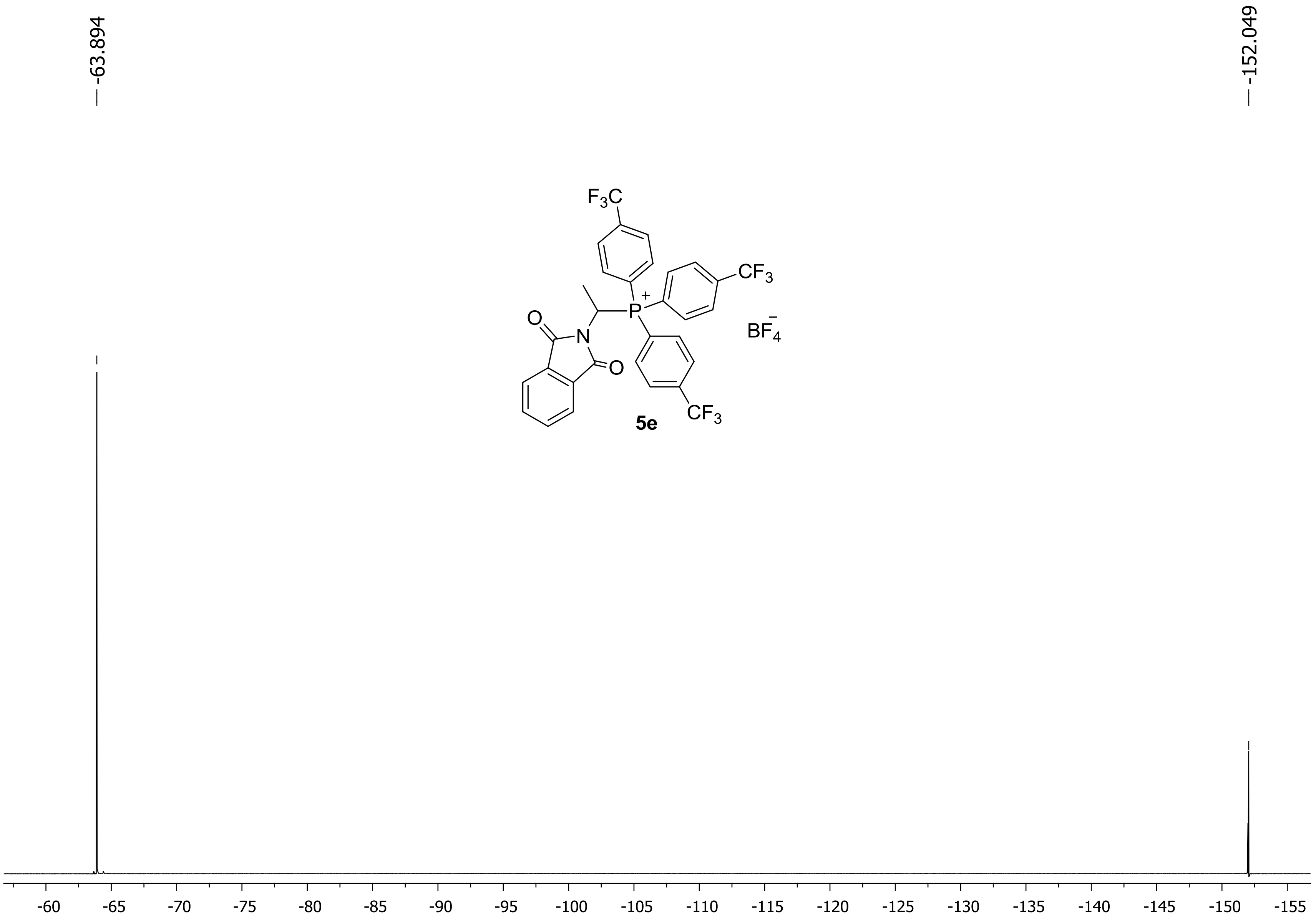

^{19}F NMR spectrum of 1-(*N*-phthalimido)ethyltris(4-trifluoromethylphenyl)phosphonium tetrafluoroborate (**5e**); 376 MHz/$CDCl_3$; δ (ppm).

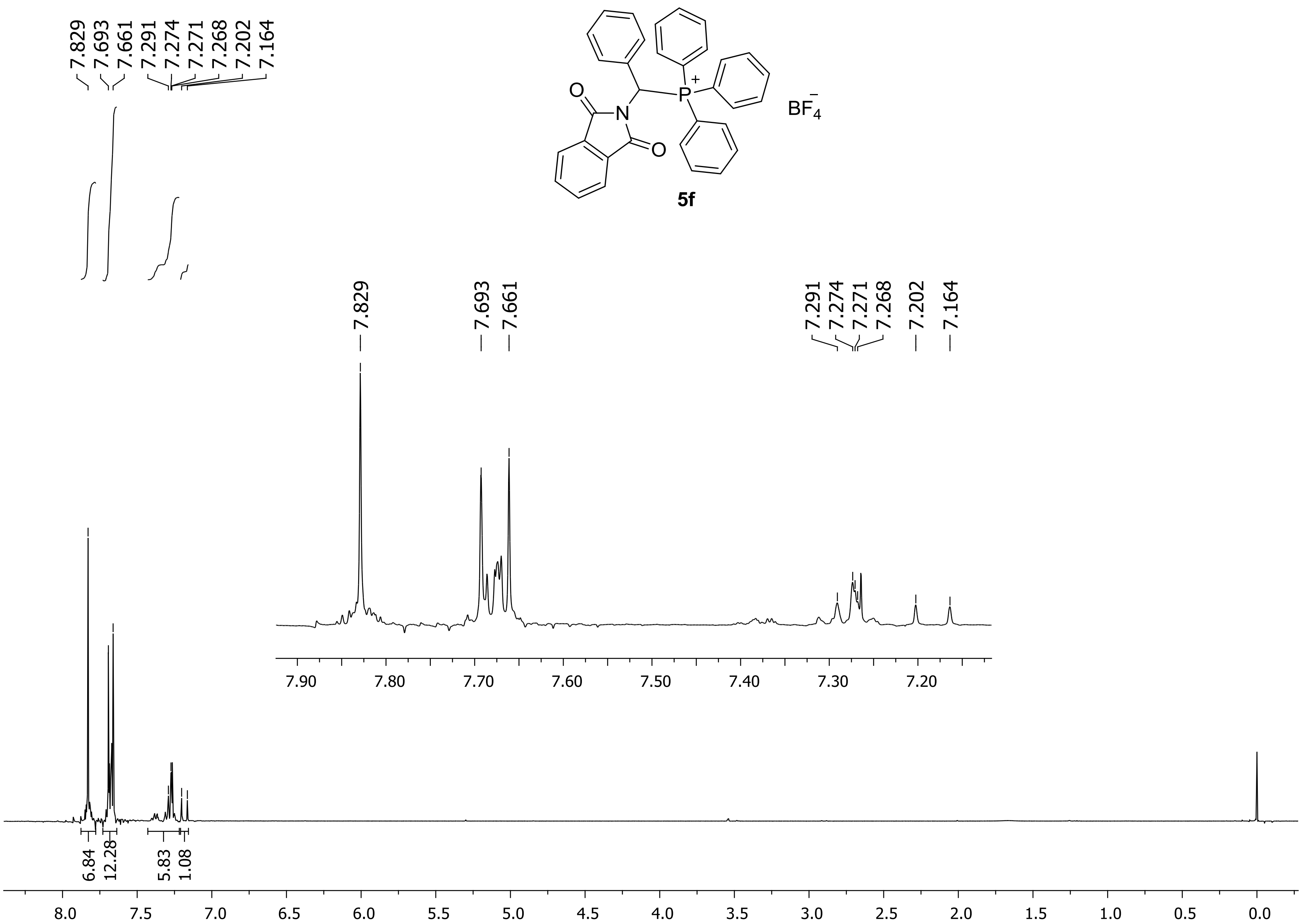

^{1}H NMR spectrum of 1-(*N*-phthalimido)phenylmethyltriphenylphosphonium tetrafluoroborate (**5f**); 400 MHz/$CDCl_3$/TMS; δ (ppm).

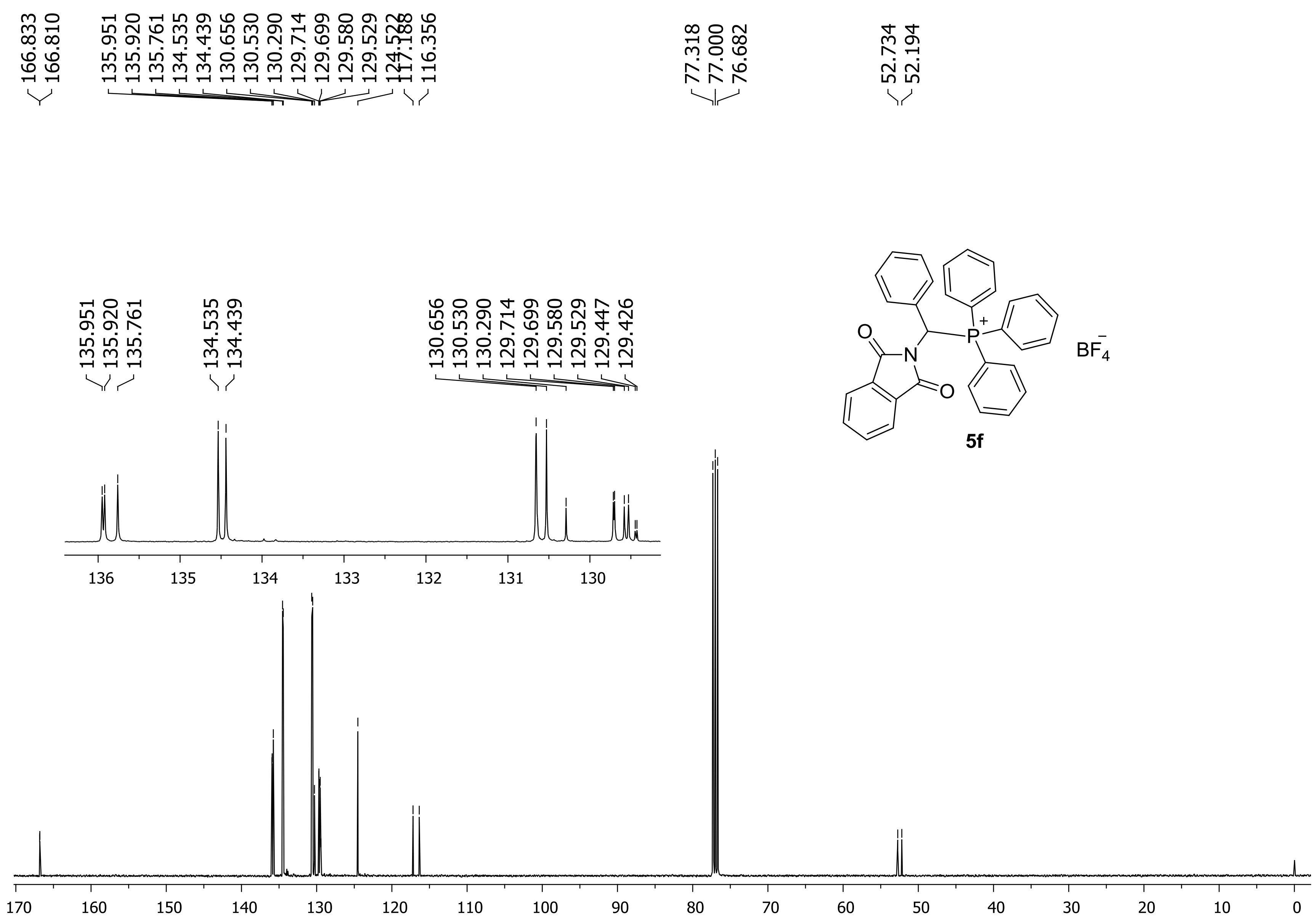

^{13}C NMR spectrum of 1-(*N*-phthalimido)phenylmethyltriphenylphosphonium tetrafluoroborate (**5f**); 100 MHz/$CDCl_3$/TMS; δ (ppm).

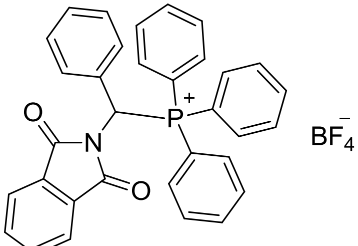

5f

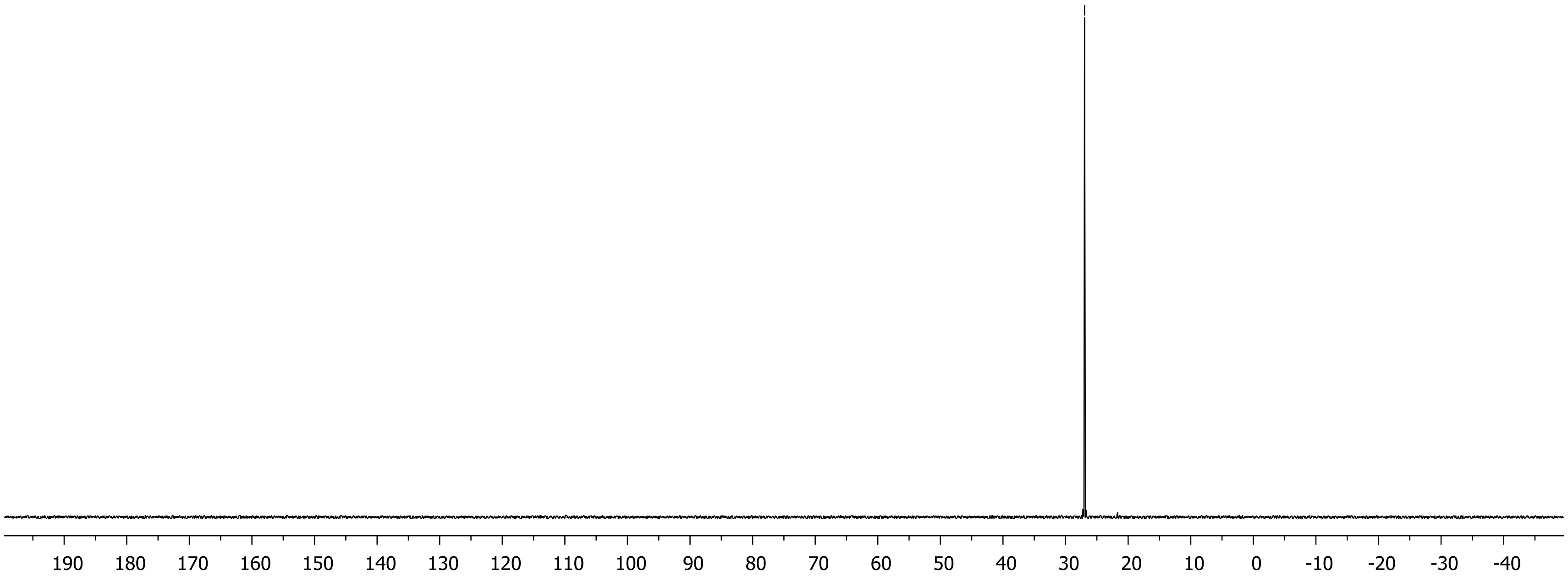

^{31}P NMR spectrum of 1-(*N*-phthalimido)phenylmethyltriphenylphosphonium tetrafluoroborate (**5f**); 161.9 MHz/$CDCl_3$; δ (ppm).

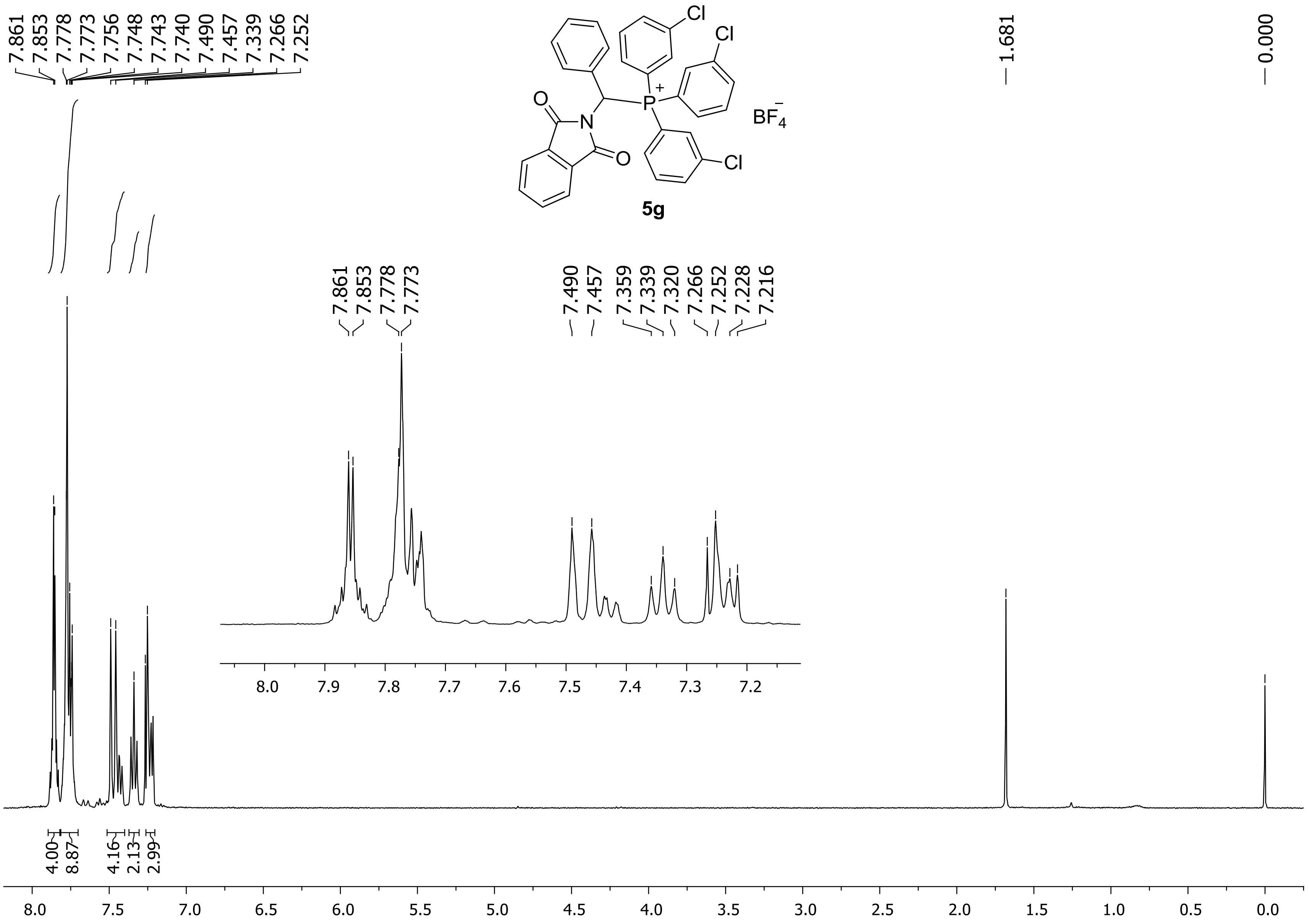

^{1}H NMR spectrum of 1-(*N*-phthalimido)phenylmethyltris(3-chlorophenyl)phosphonium tetrafluoroborate (**5g**); 400 MHz/$CDCl_3$/TMS; δ (ppm).

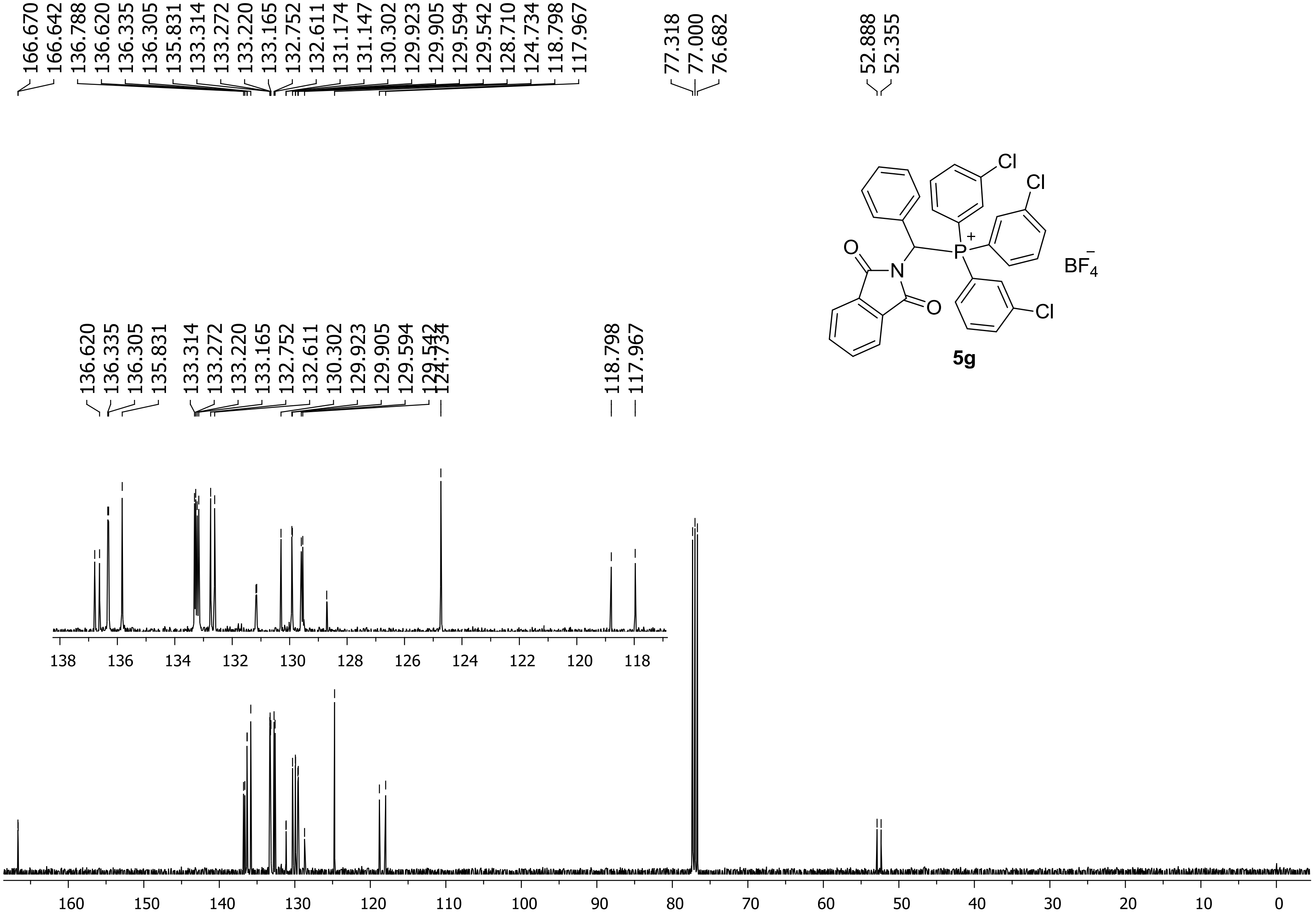

^{13}C NMR spectrum of 1-(*N*-phthalimido)phenylmethyltris(3-chlorophenyl)phosphonium tetrafluoroborate (**5g**); 100 MHz/$CDCl_3$/TMS; δ (ppm).

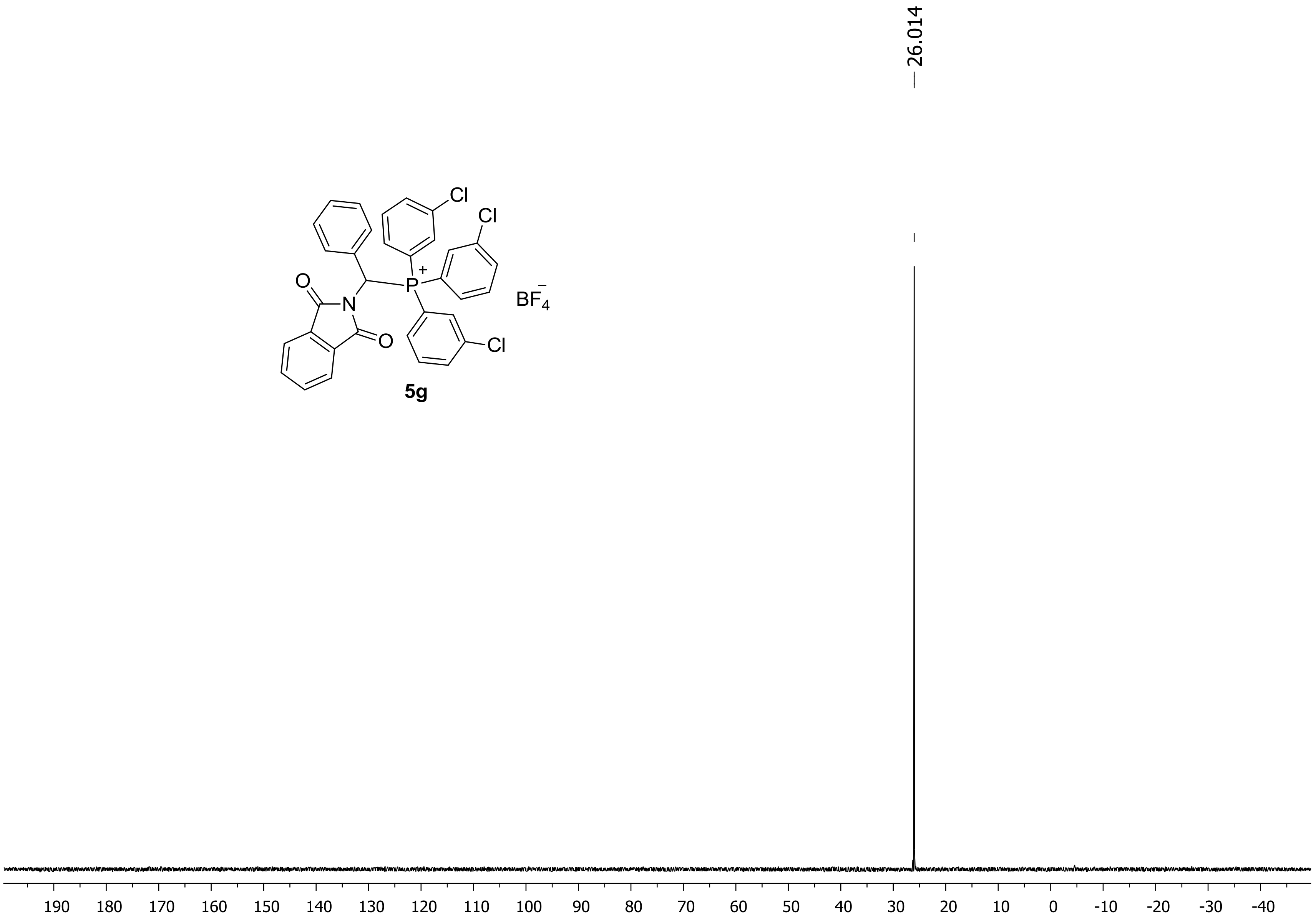

^{31}P NMR spectrum of 1-(*N*-phthalimido)phenylmethyltris(3-chlorophenyl)phosphonium tetrafluoroborate (**5g**); 161.9 MHz/$CDCl_3$; δ (ppm).

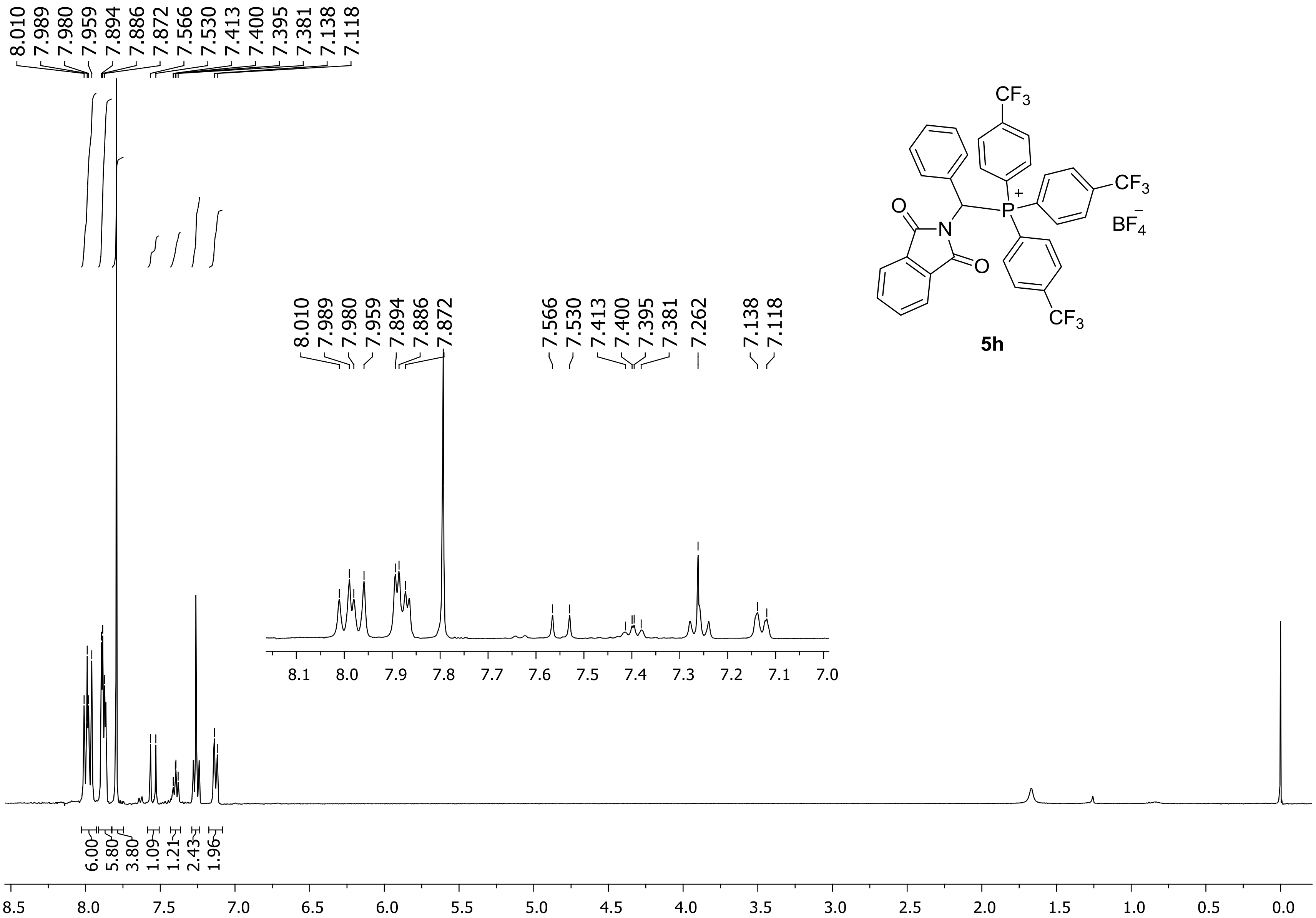

^{1}H NMR spectrum of 1-(*N*-phthalimido)phenylmethyltris(4-trifluoromethylphenyl)phosphonium tetrafluoroborate (**5h**); 400 MHz/$CDCl_3$/TMS; δ (ppm).

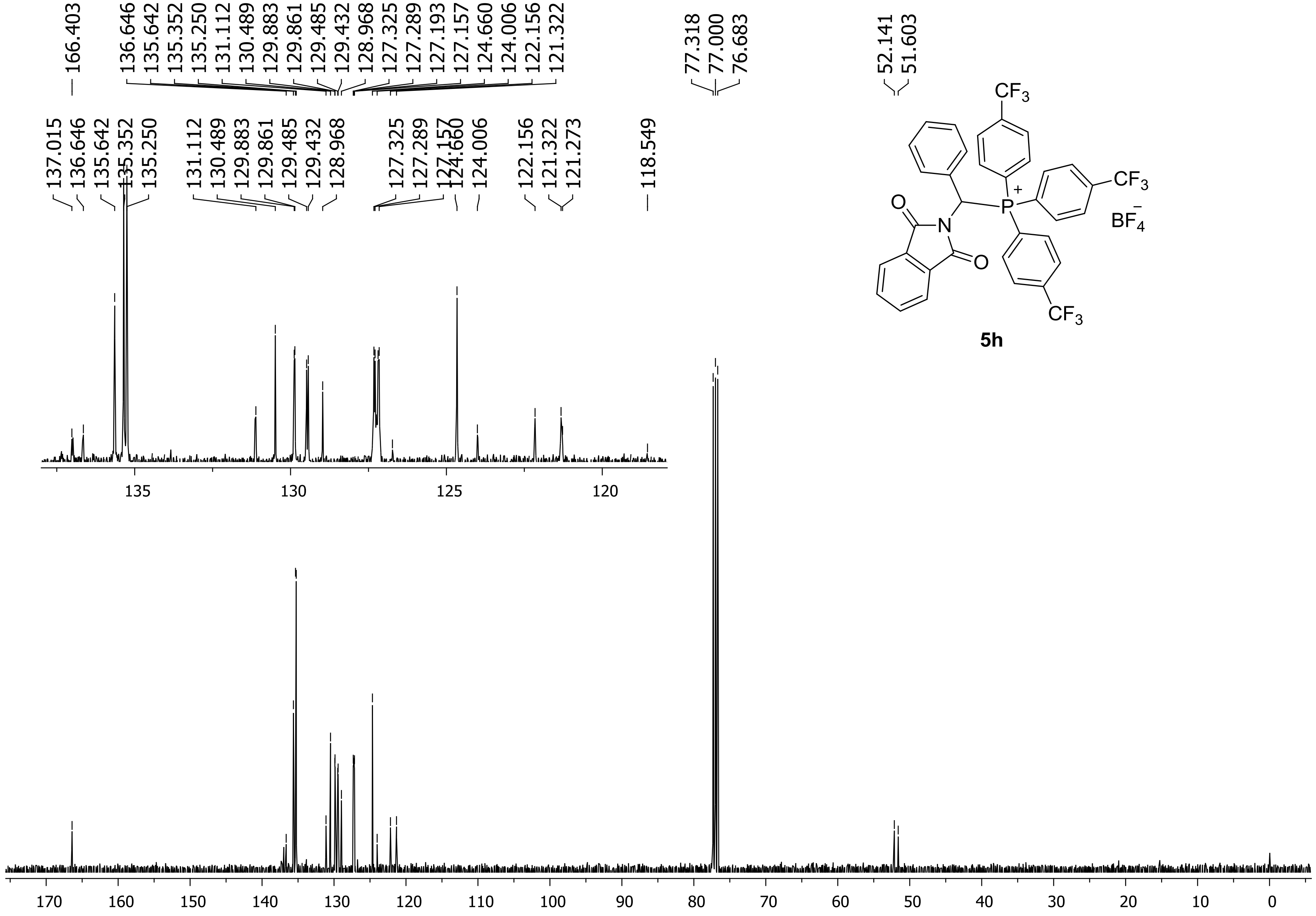

^{13}C NMR spectrum of 1-(*N*-phthalimido)phenylmethyltris(4-trifluoromethylphenyl)phosphonium tetrafluoroborate (**5h**); 100 MHz/$CDCl_3$/TMS; δ (ppm).

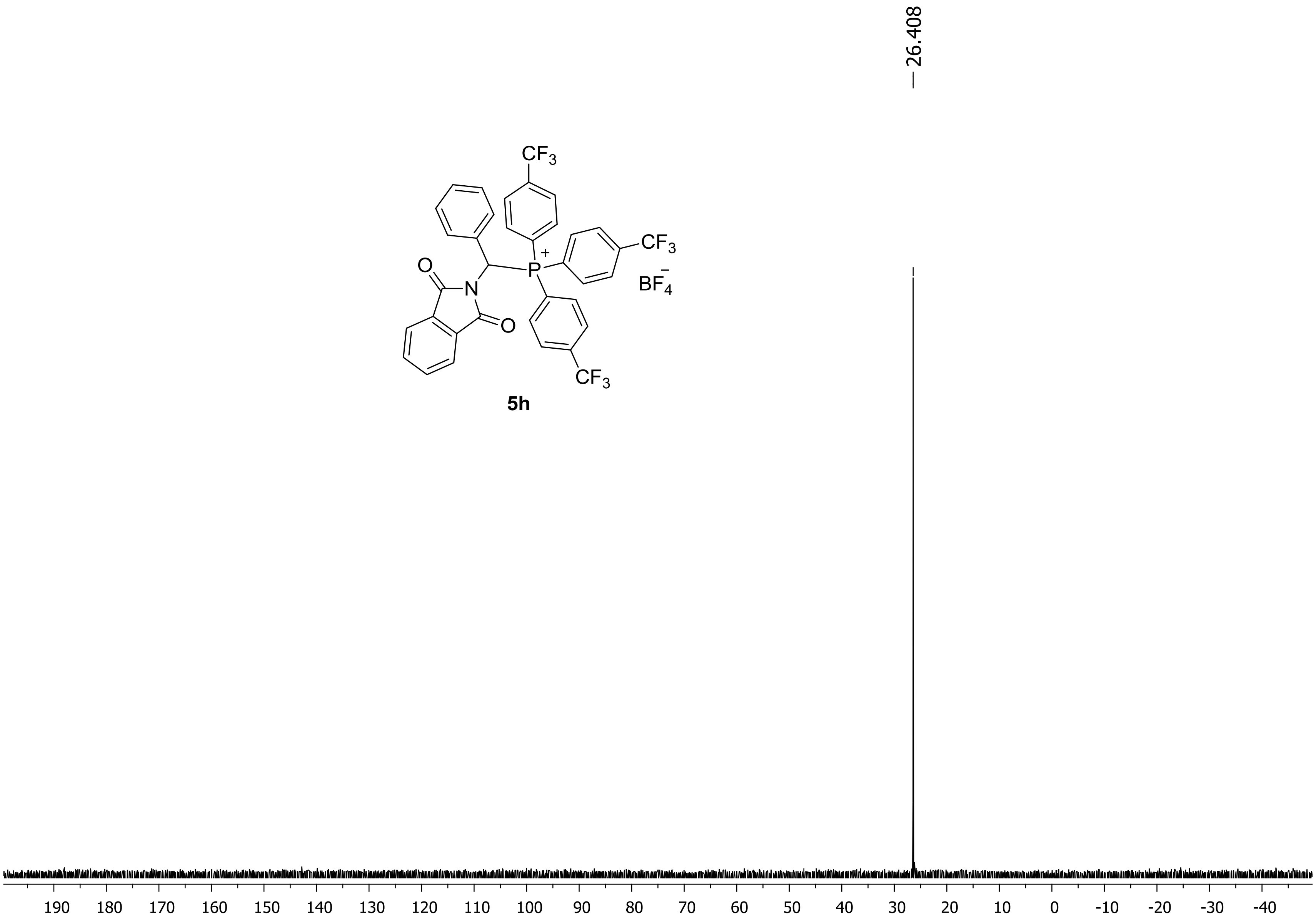

^{31}P NMR spectrum of 1-(*N*-phthalimido)phenylmethyltris(4-trifluoromethylphenyl)phosphonium tetrafluoroborate (**5h**); 161.9 MHz/$CDCl_3$; δ (ppm).

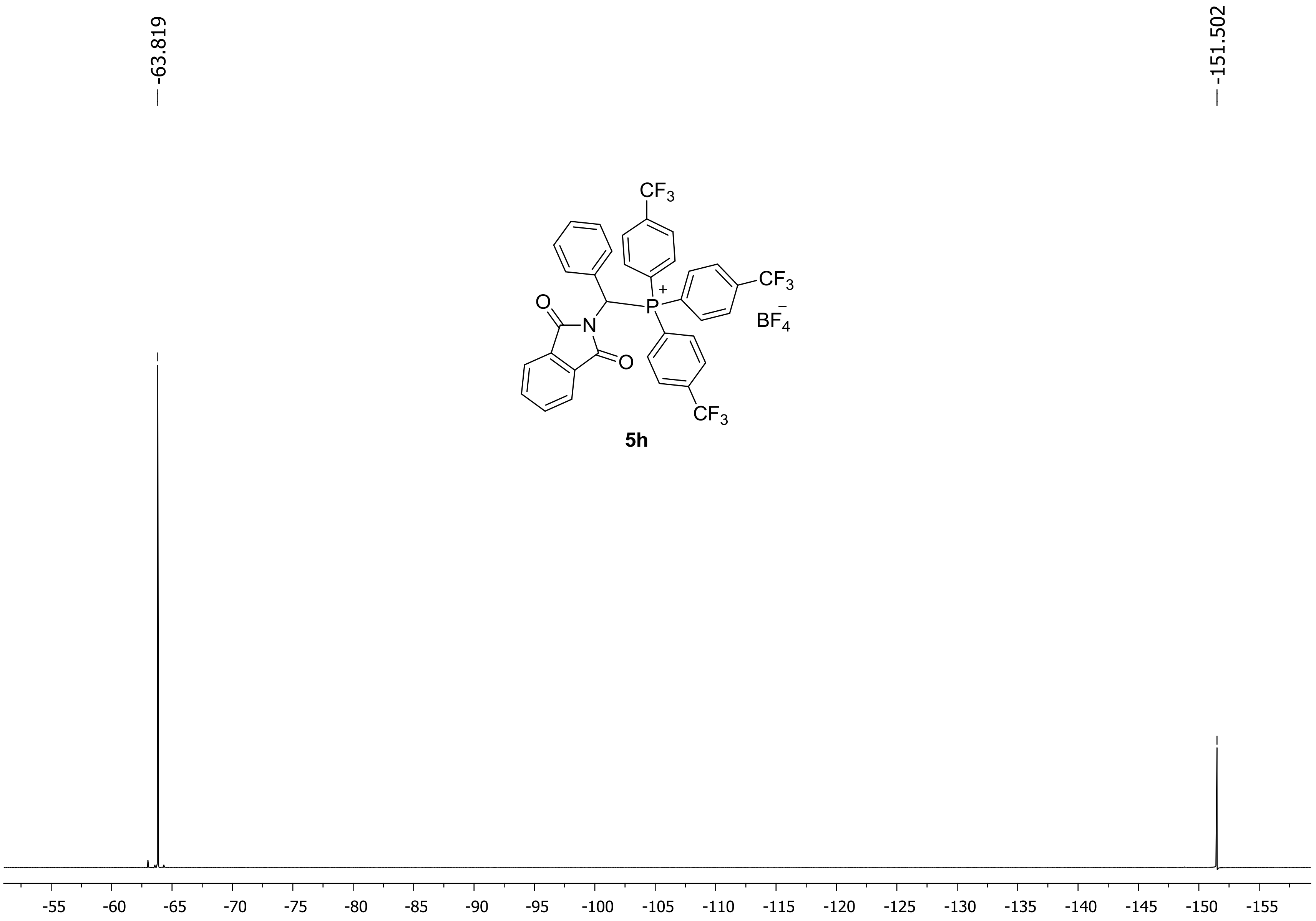

^{19}F NMR spectrum of 1-(*N*-phthalimido)phenylmethyltris(4-trifluoromethylphenyl)phosphonium tetrafluoroborate (**5h**); 376 MHz/$CDCl_3$; δ (ppm).

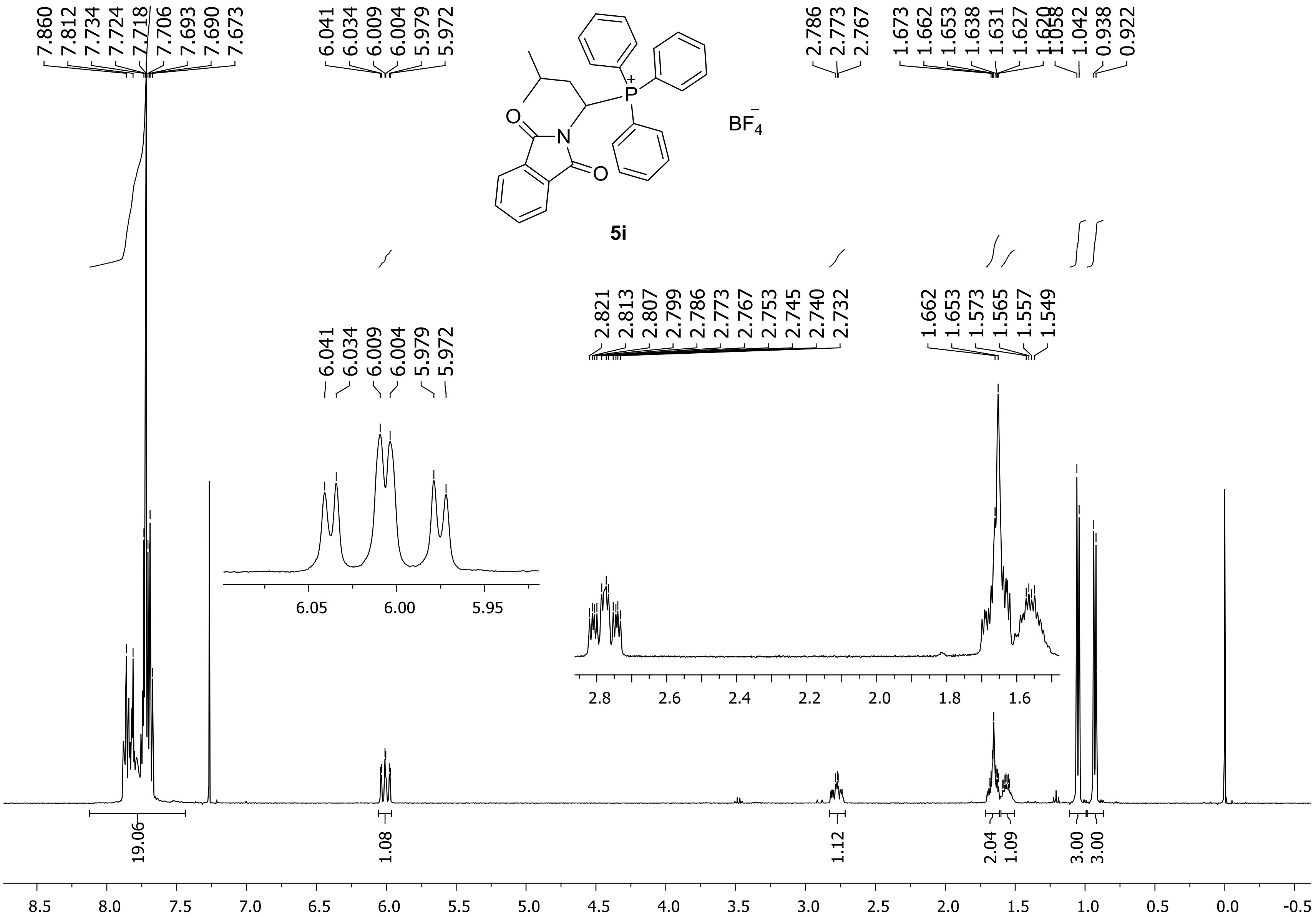

^{1}H NMR spectrum of 3-methyl-1-(*N*-phthalimido)butyltriphenylphosphonium tetrafluoroborate (**5i**); 400 MHz/$CDCl_3$/TMS; δ (ppm).

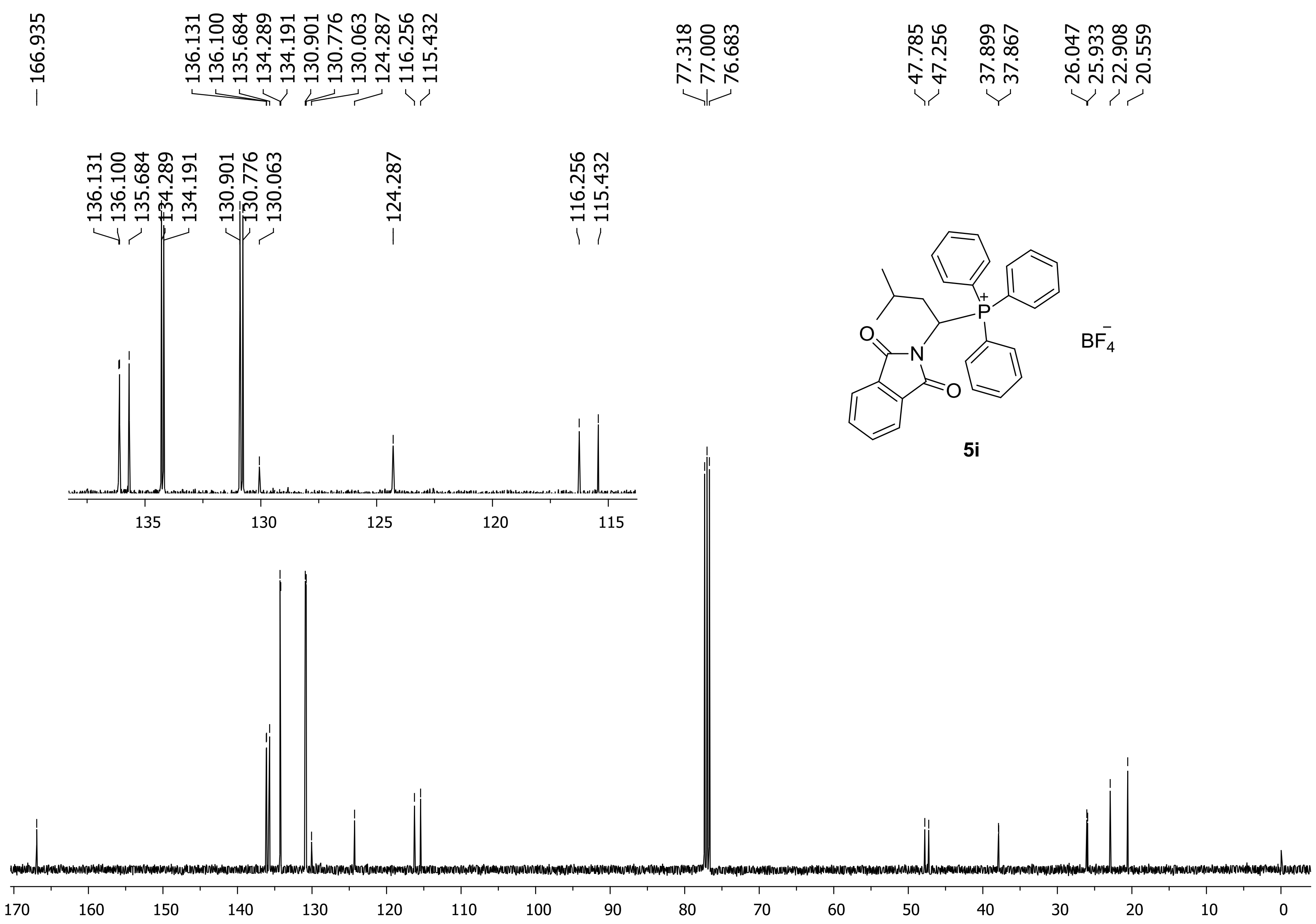

^{13}C NMR spectrum of 3-methyl-1-(*N*-phthalimido)butyltriphenylphosphonium tetrafluoroborate (**5i**); 100 MHz/$CDCl_3$/TMS; δ (ppm).

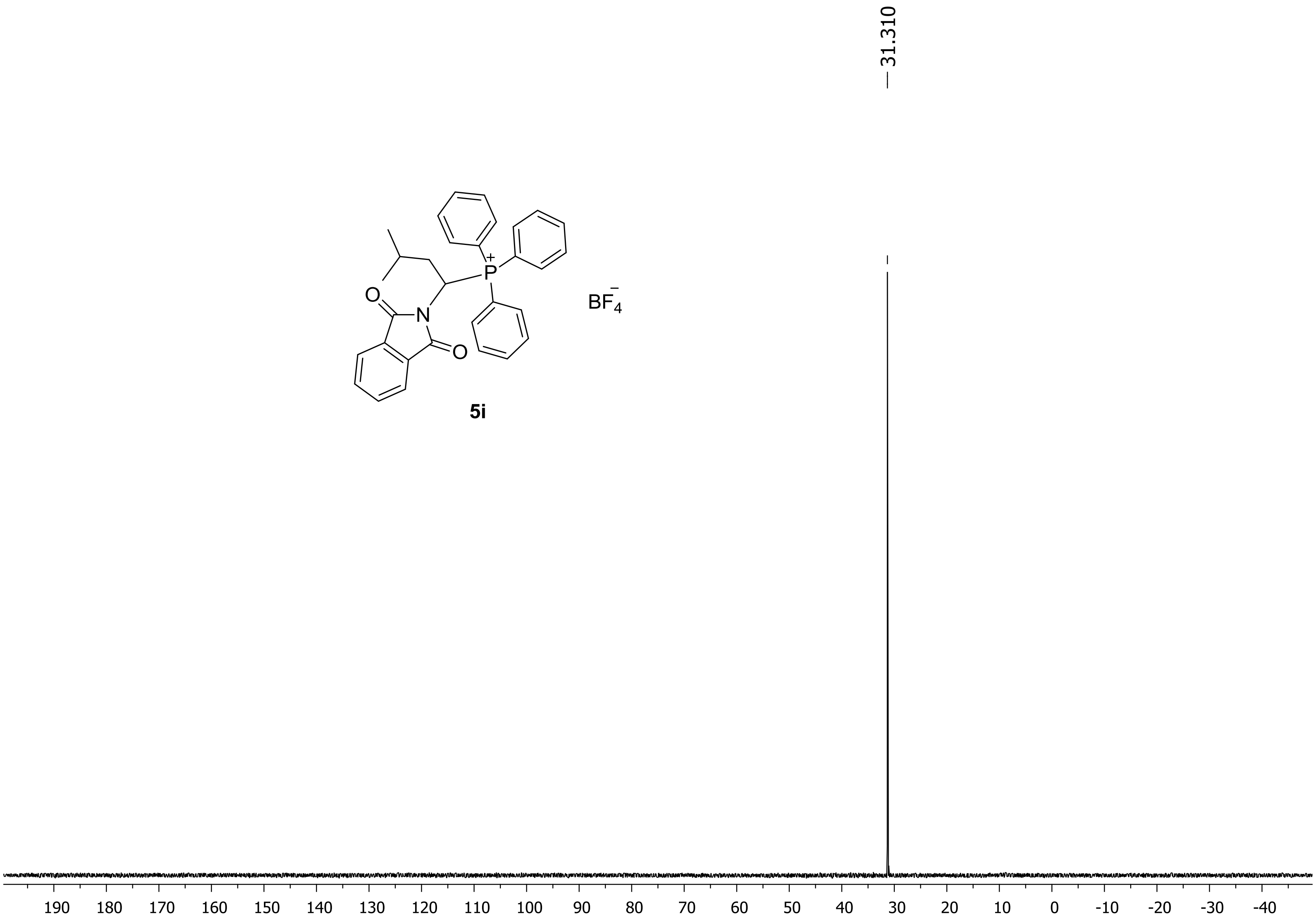

^{31}P NMR spectrum of 3-methyl-1-(*N*-phthalimido)butyltriphenylphosphonium tetrafluoroborate (**5i**); 161.9 MHz/$CDCl_3$; δ (ppm).

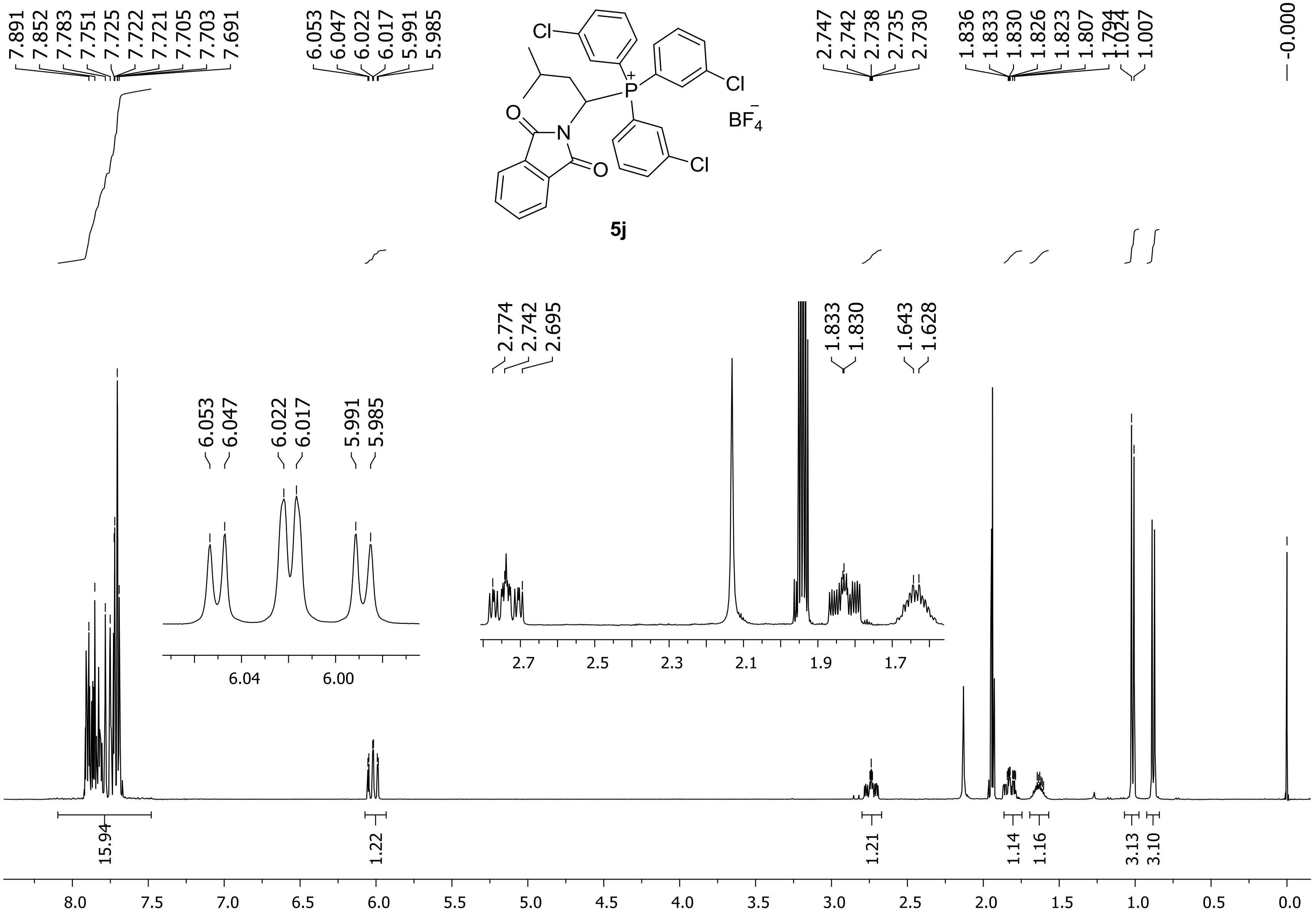

^{1}H NMR spectrum of 3-methyl-1-(*N*-phthalimido)butyltris(3-chlorophenyl)phosphonium tetrafluoroborate (**5j**); 400 MHz/CD_3CN/TMS; δ (ppm).

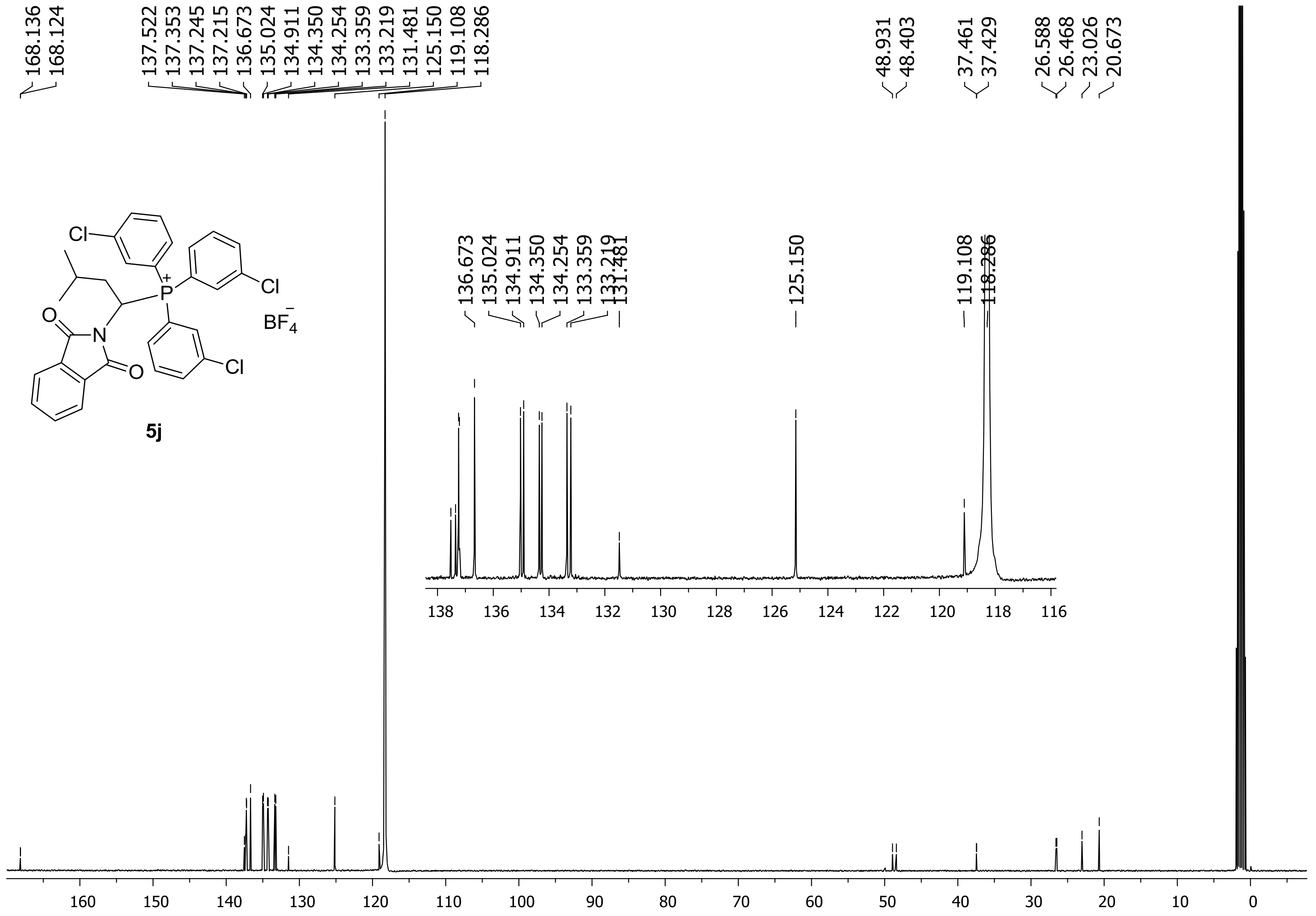

^{13}C NMR spectrum of 3-methyl-1-(*N*-phthalimido)butyltris(3-chlorophenyl)phosphonium tetrafluoroborate (**5j**); 100 MHz/CD_3CN/TMS; δ (ppm).

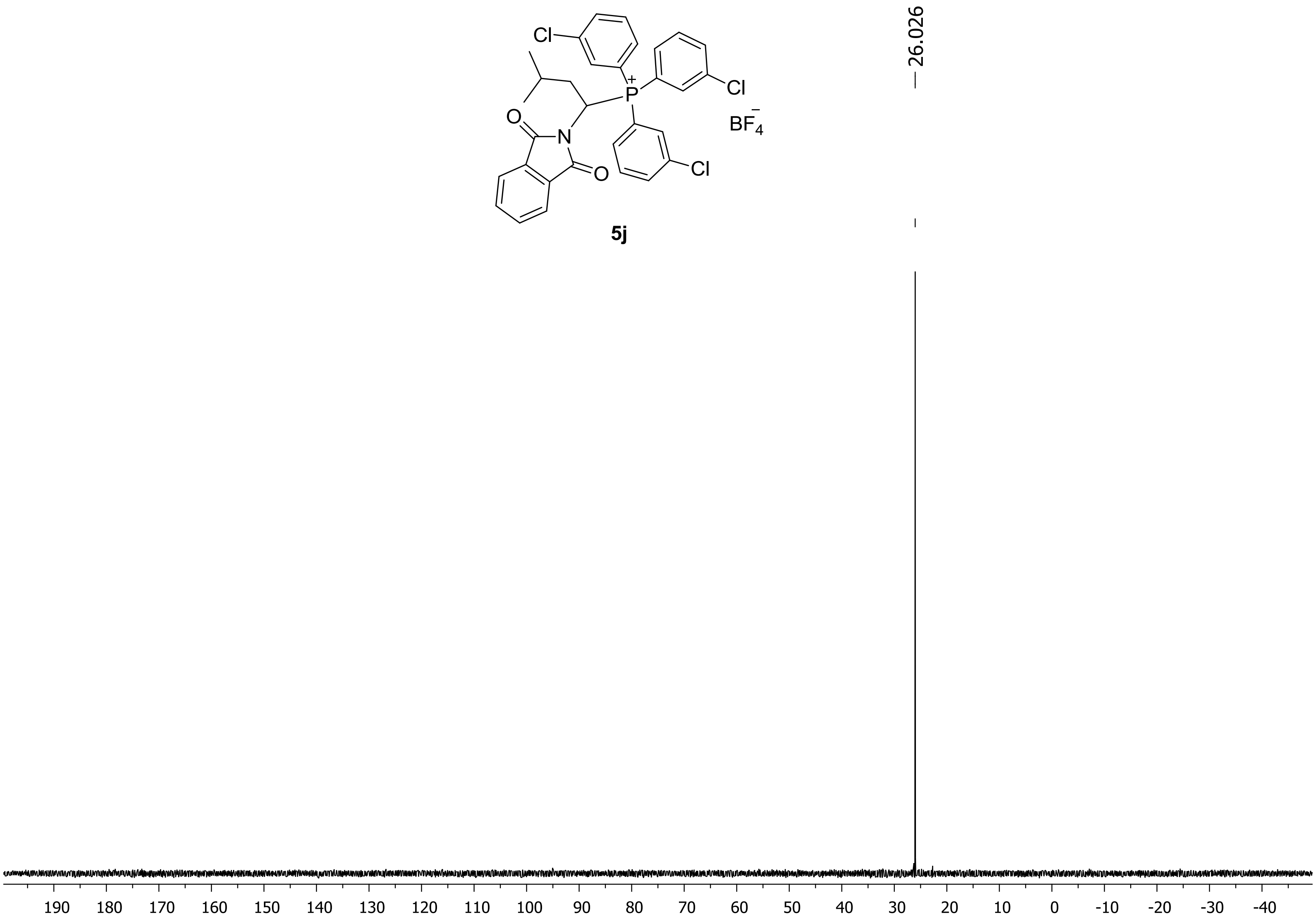

^{31}P NMR spectrum of 3-methyl-1-(*N*-phthalimido)butyltris(3-chlorophenyl)phosphonium tetrafluoroborate (**5j**); 161.9 MHz/CD_3CN; δ (ppm).

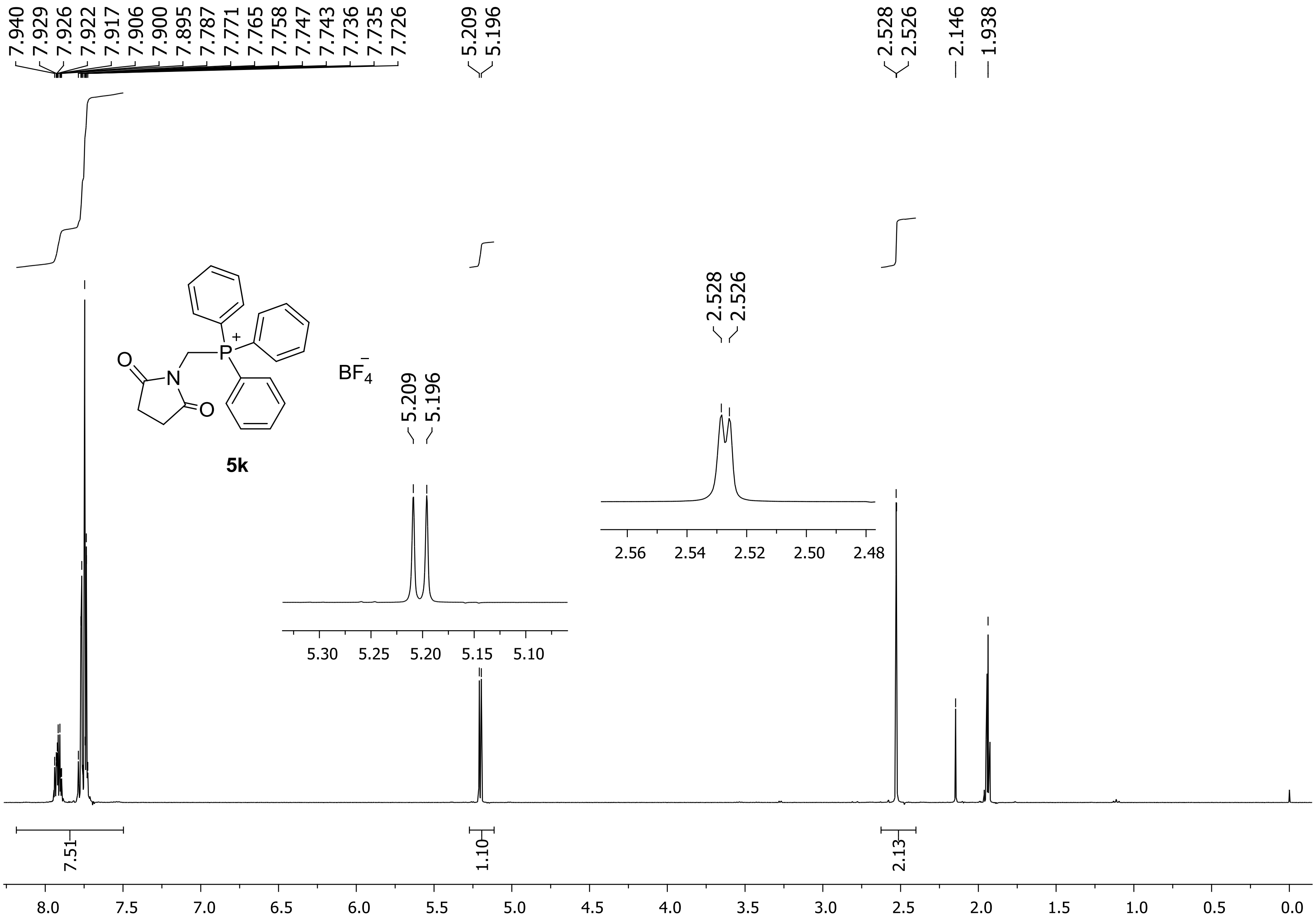

^{1}H NMR spectrum of 1-(*N*-succinimido)methyltriphenylphosphonium tetrafluoroborate (**5k**); 400 MHz/CD_3CN/TMS; δ (ppm).

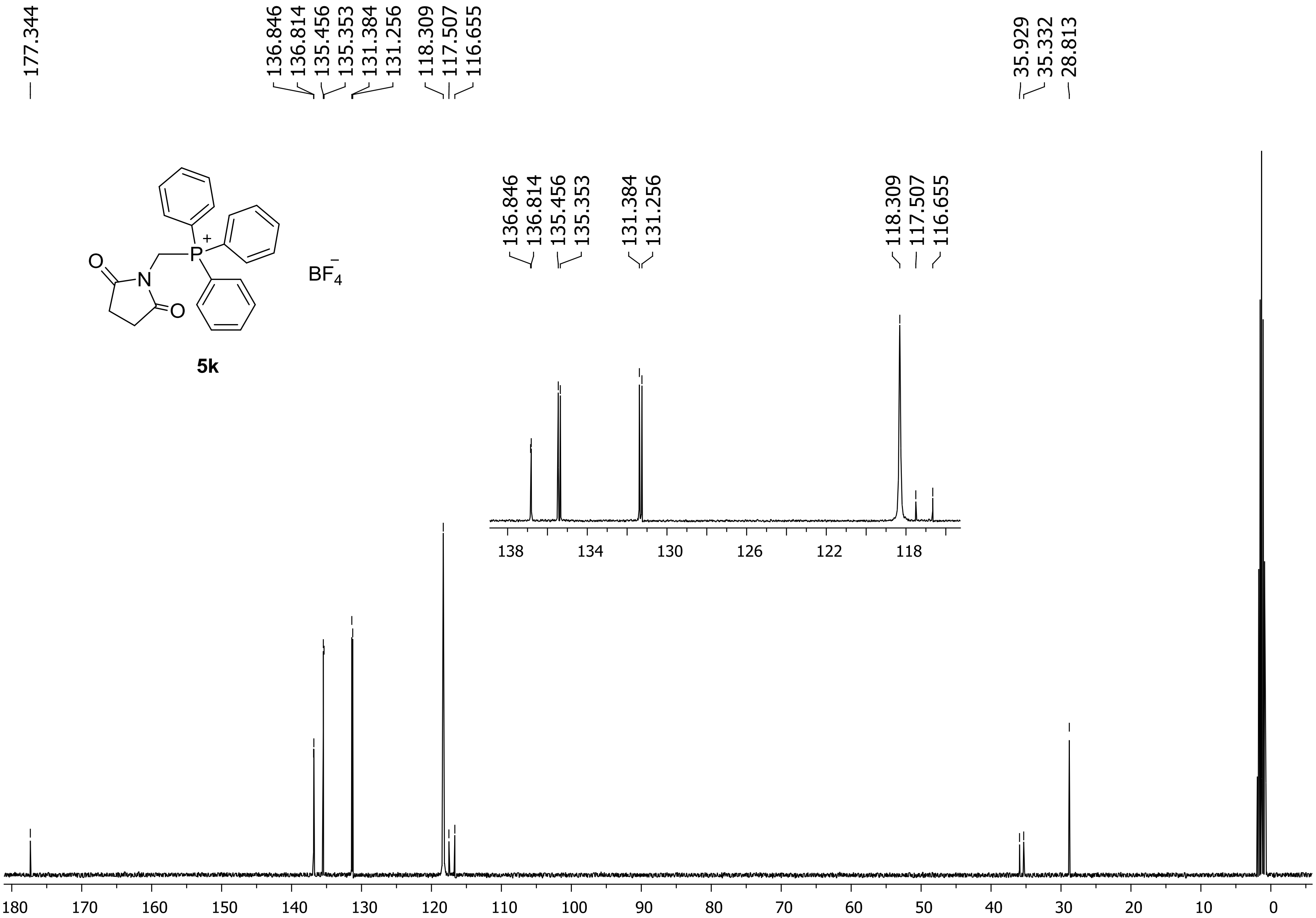

^{13}C NMR spectrum of 1-(*N*-succinimido)methyltriphenylphosphonium tetrafluoroborate (**5k**); 100 MHz/CD_3CN/TMS; δ (ppm).

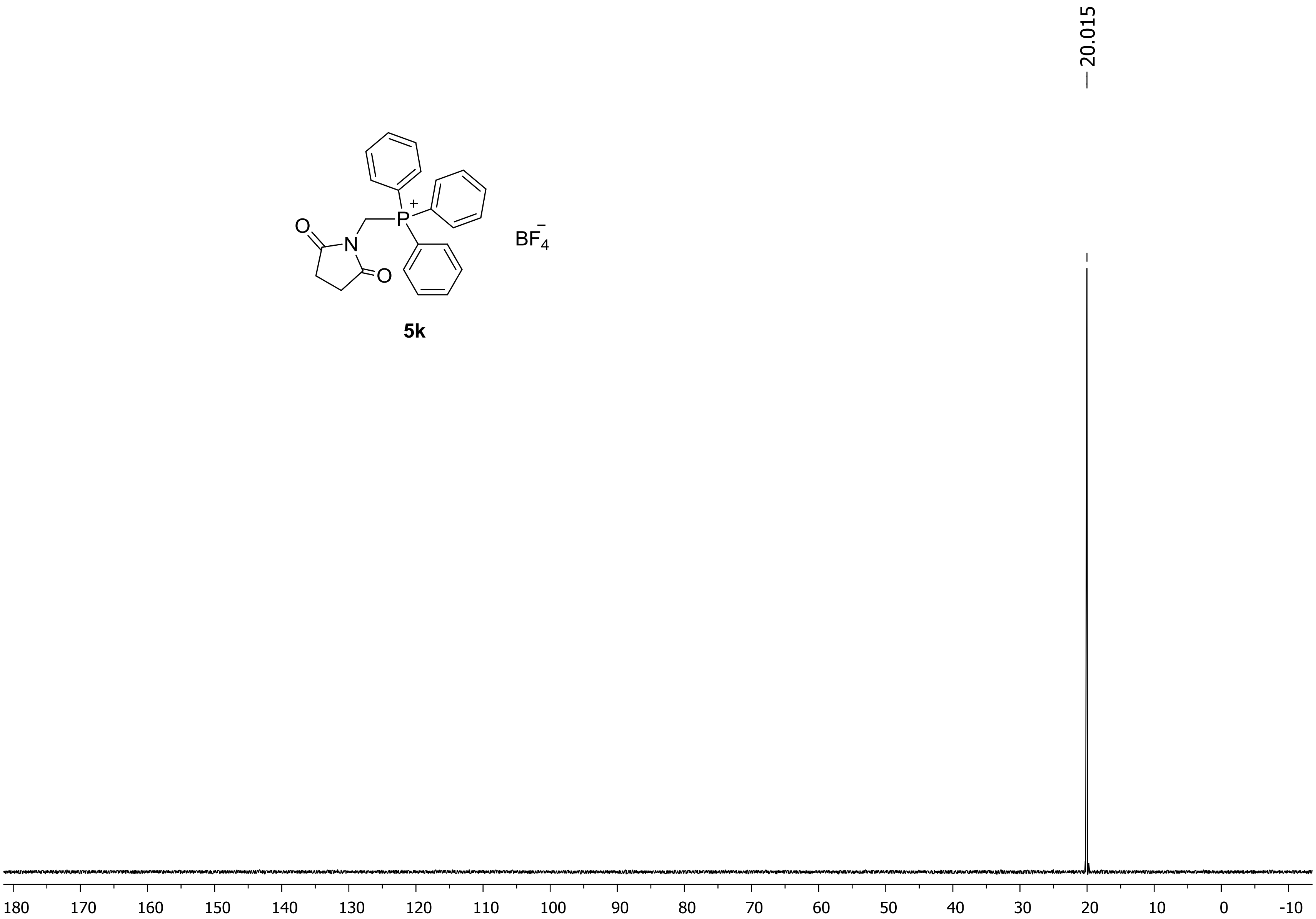

^{31}P NMR spectrum of 1-(*N*-succinimido)methyltriphenylphosphonium tetrafluoroborate (**5k**); 161.9 MHz/CD_3CN; δ (ppm).

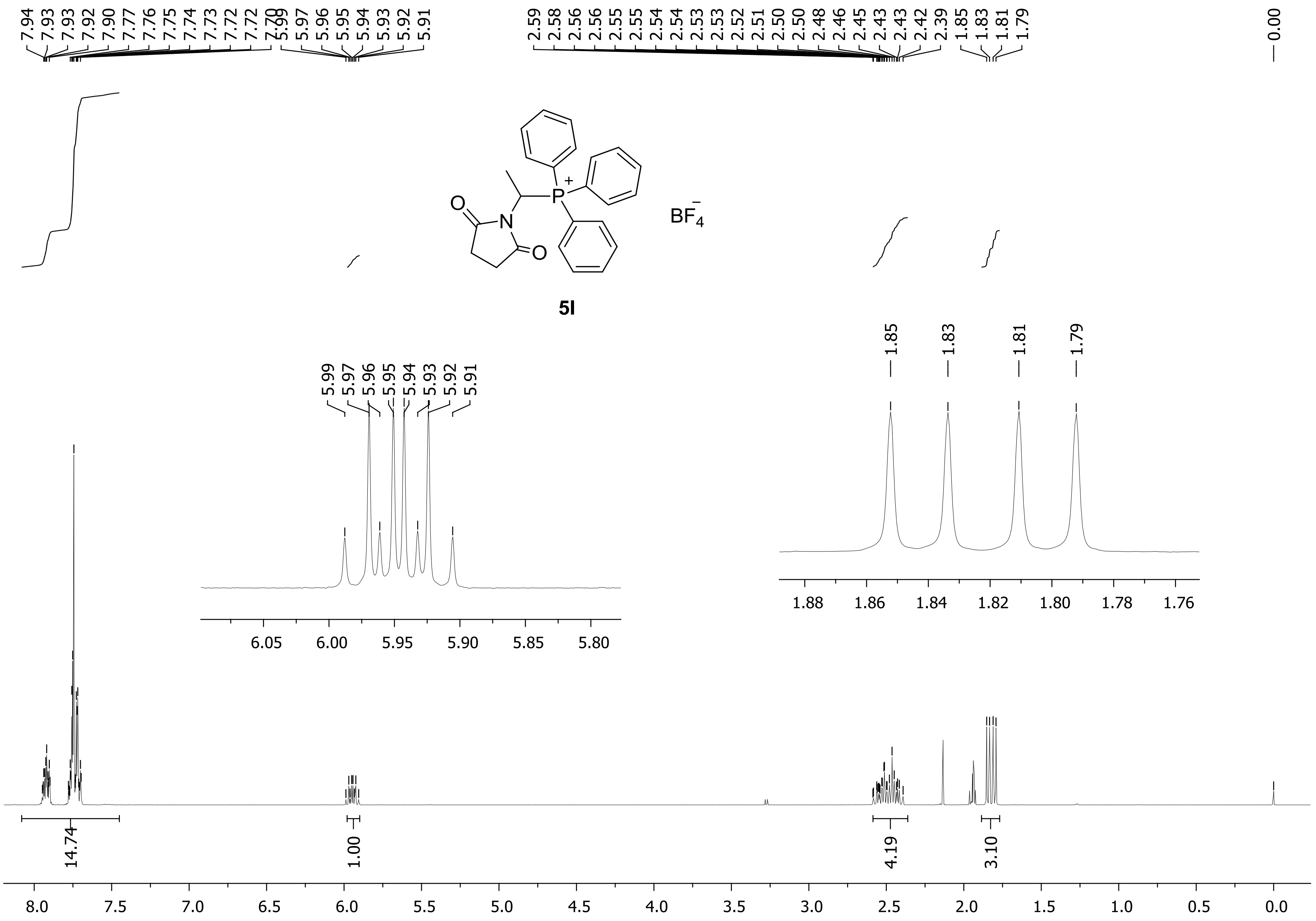

^{1}H NMR spectrum of 1-(*N*-succinimido)ethyltriphenylphosphonium tetrafluoroborate (**5l**); 400 MHz/ CD_3CN/TMS; δ (ppm).

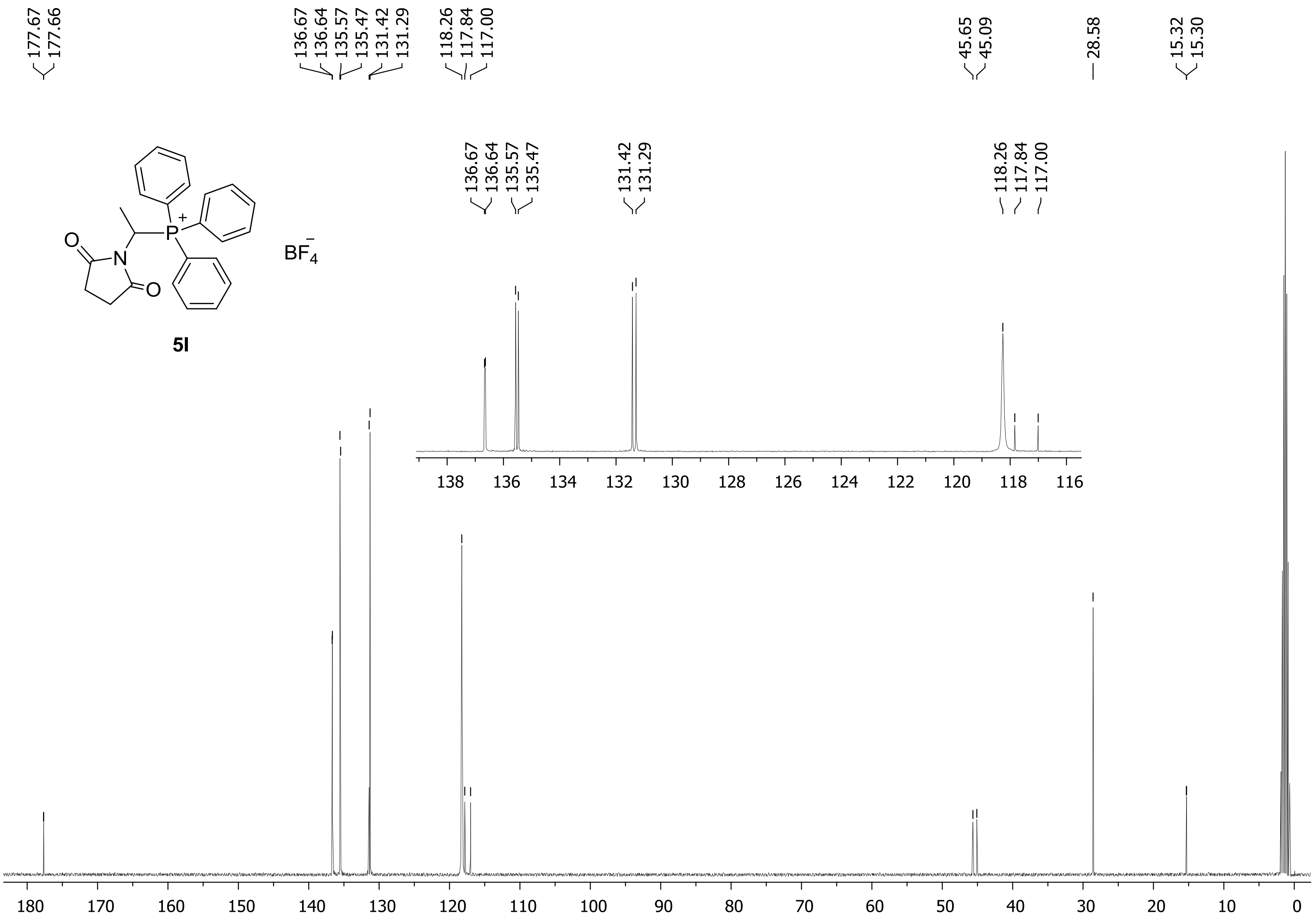

^{13}C NMR spectrum of 1-(*N*-succinimido)ethyltriphenylphosphonium tetrafluoroborate (**5l**); 100 MHz/CD_3CN/TMS; δ (ppm).

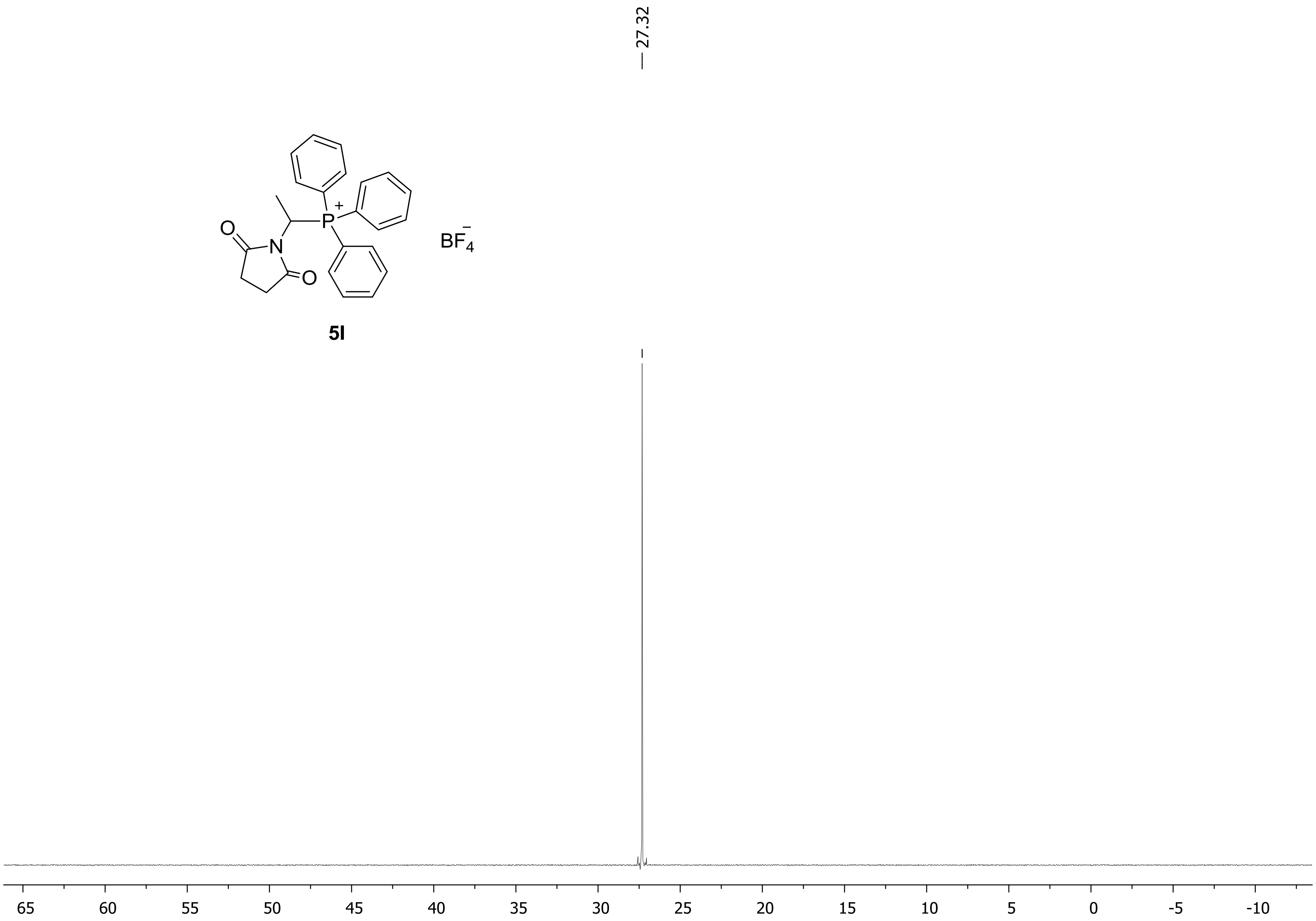

^{31}P NMR spectrum of 1-(*N*-succinimido)ethyltriphenylphosphonium tetrafluoroborate (**5l**); 161.9 MHz/CD_3CN; δ (ppm).

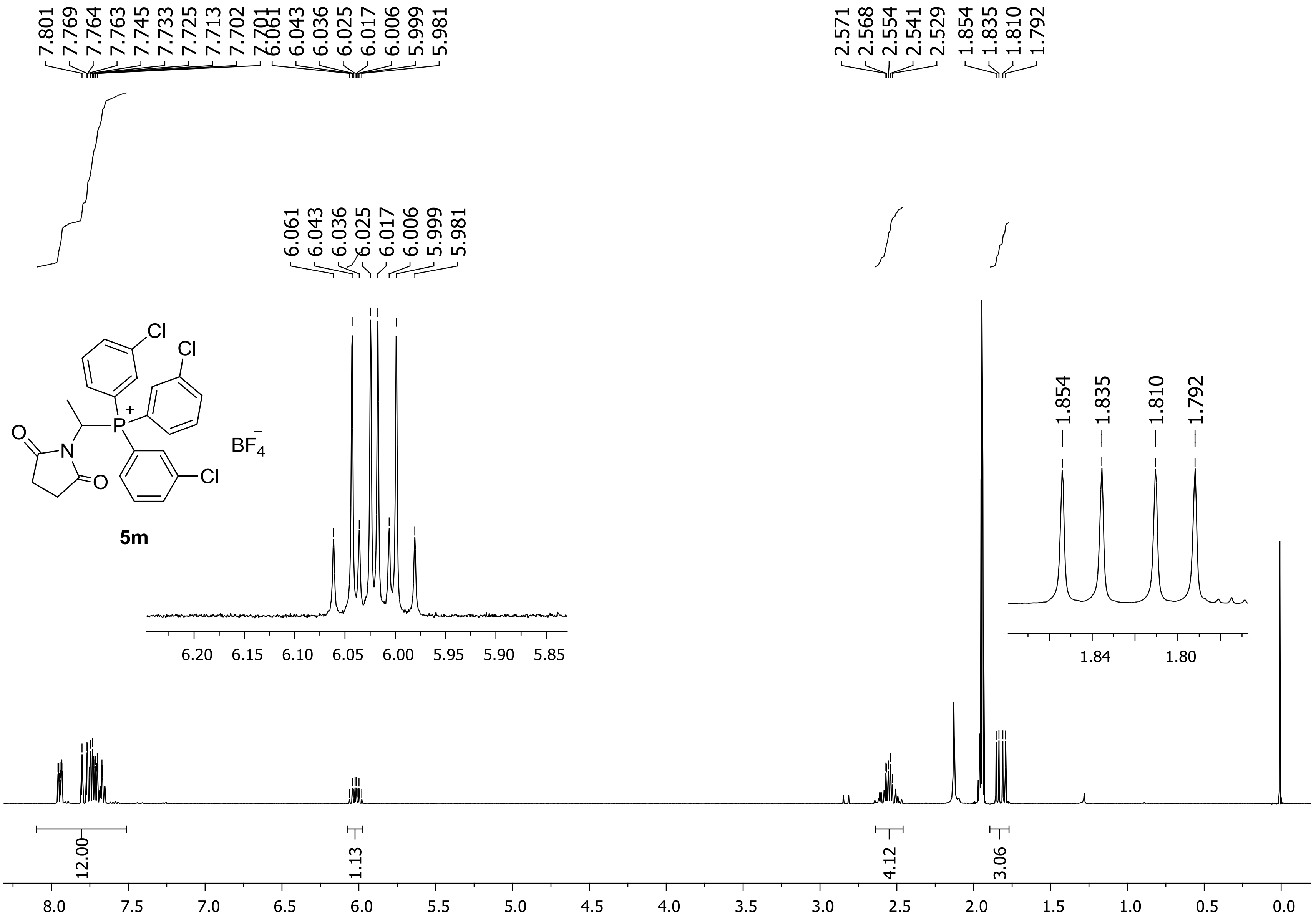

^{1}H NMR spectrum of 1-(*N*-succinimido)ethyltris(3-chlorophenyl)phosphonium tetrafluoroborate (**5m**); 400 MHz/CD_3CN/TMS; δ (ppm).

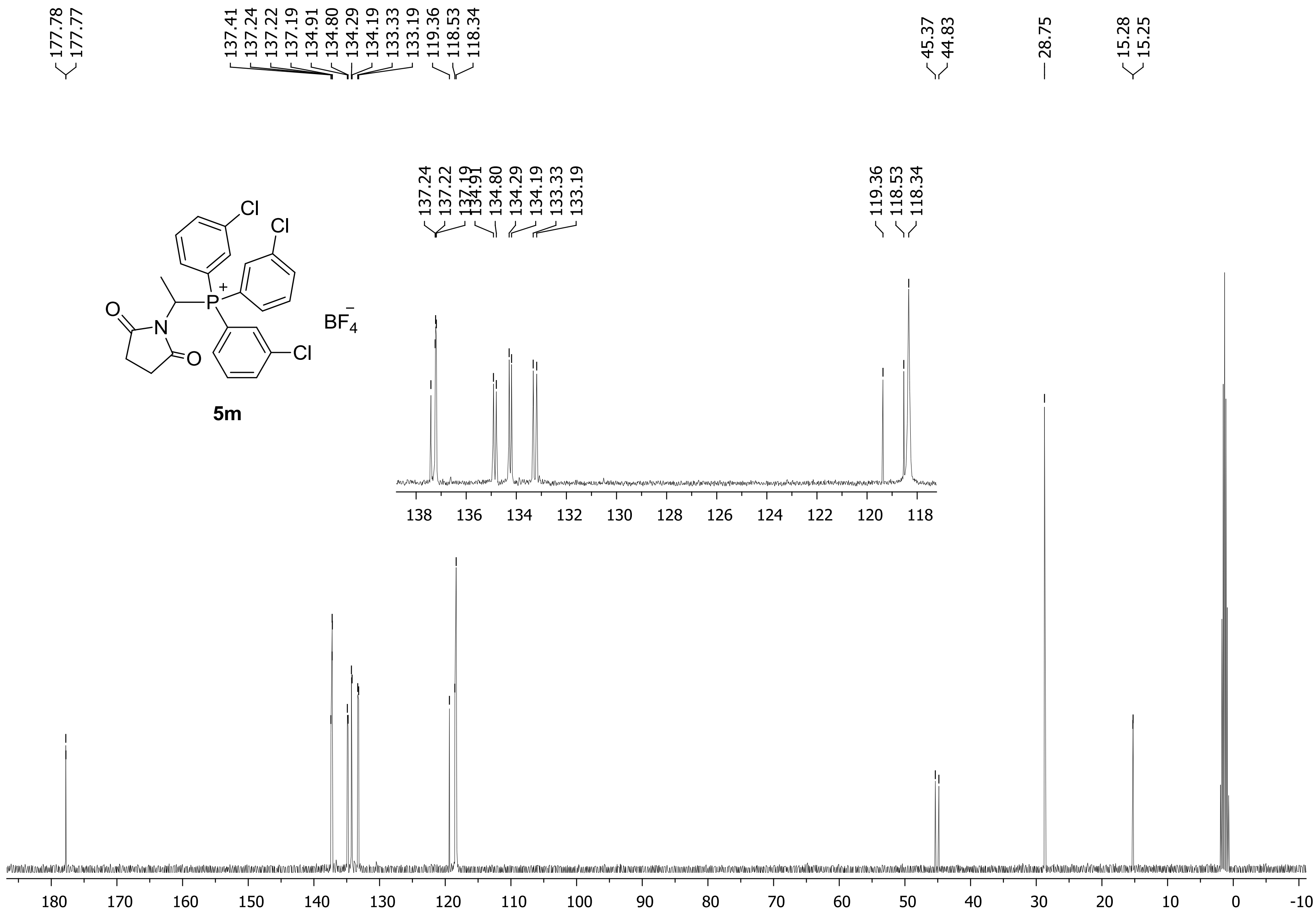

^{13}C NMR spectrum of 1-(*N*-succinimido)ethyltris(3-chlorophenyl)phosphonium tetrafluoroborate (**5m**); 100 MHz/CD_3CN/TMS; δ (ppm).

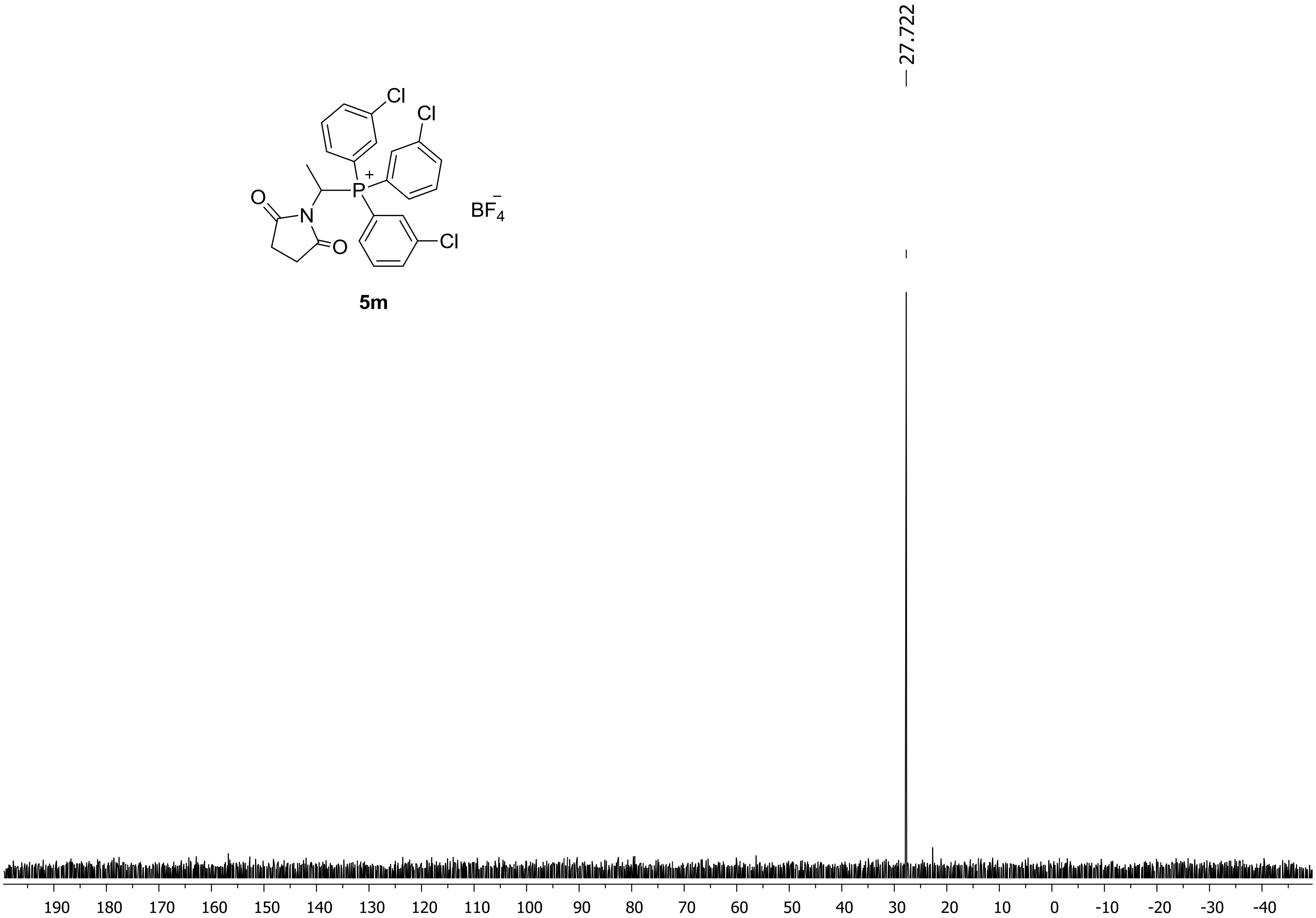

5m

^{31}P NMR spectrum of 1-(*N*-succinimido)ethyltris(3-chlorophenyl)phosphonium tetrafluoroborate (**5m**); 161.9 MHz/CD_3CN; δ (ppm).

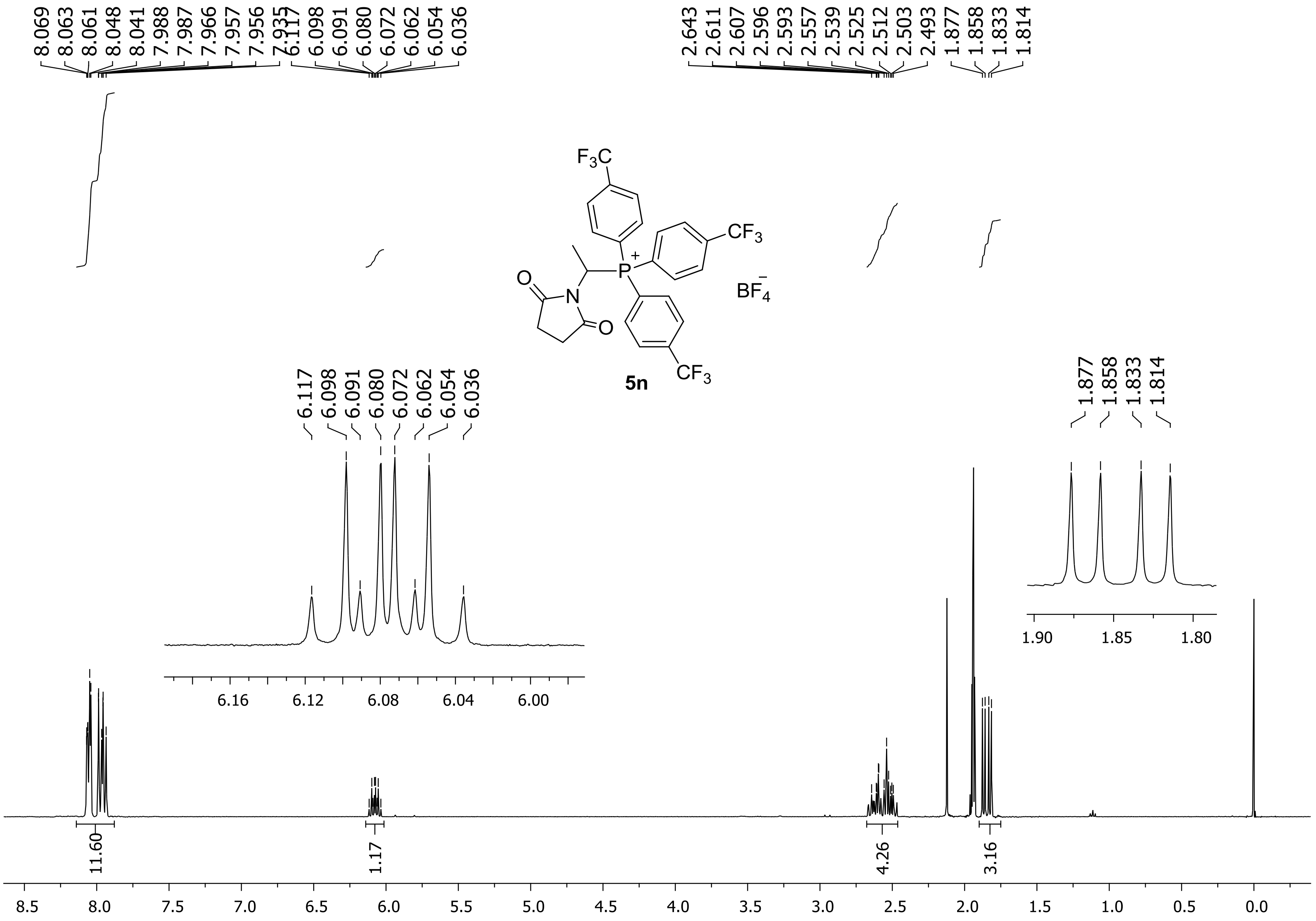

^{1}H NMR spectrum of 1-(*N*-succinimido)ethyltris(4-trifluoromethylphenyl)phosphonium tetrafluoroborate (**5n**); 400 MHz/CD_3CN/TMS; δ (ppm).

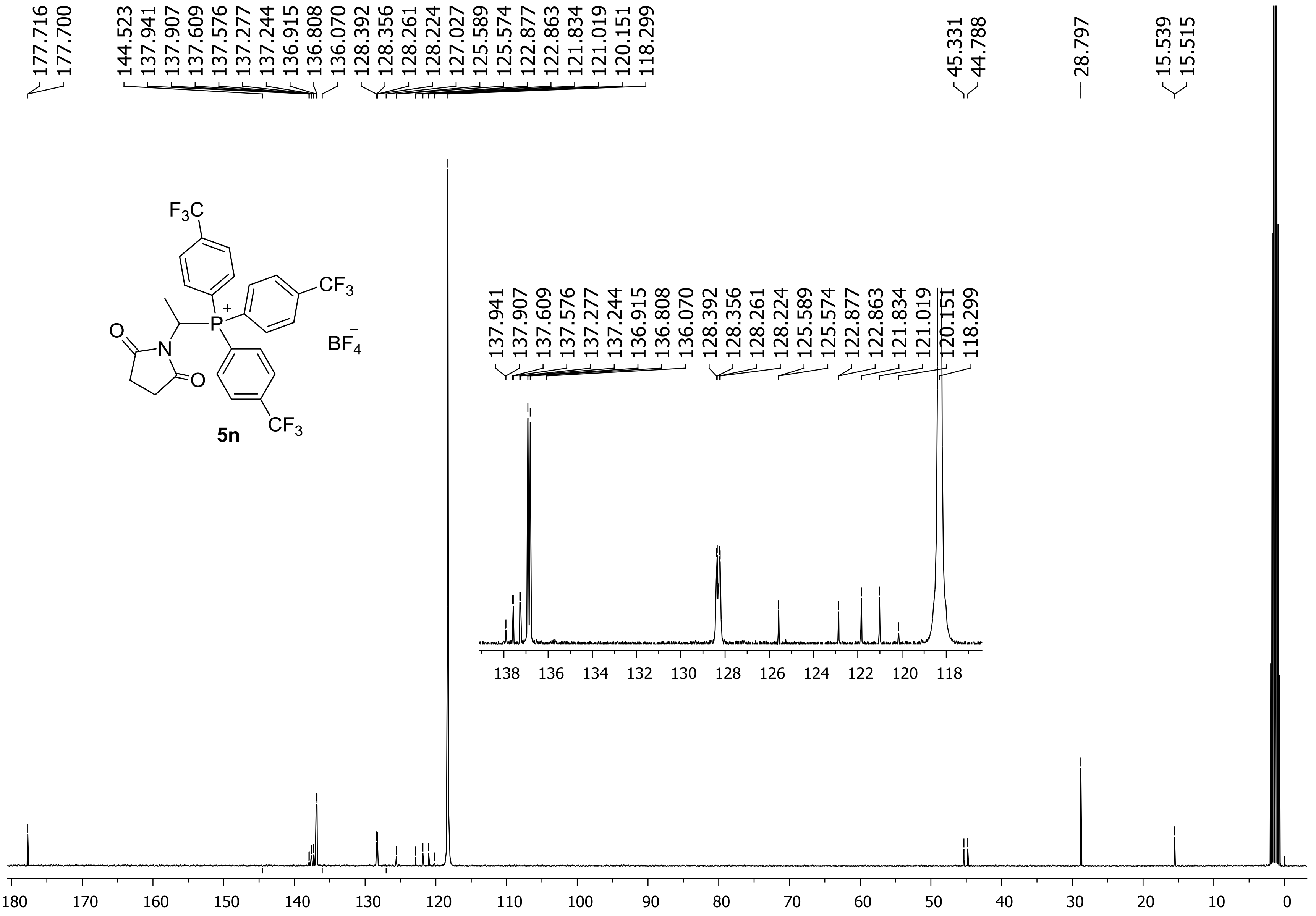

^{13}C NMR spectrum of 1-(*N*-succinimido)ethyltris(4-trifluoromethylphenyl)phosphonium tetrafluoroborate (**5n**); 100 MHz/CD_3CN/TMS; δ (ppm).

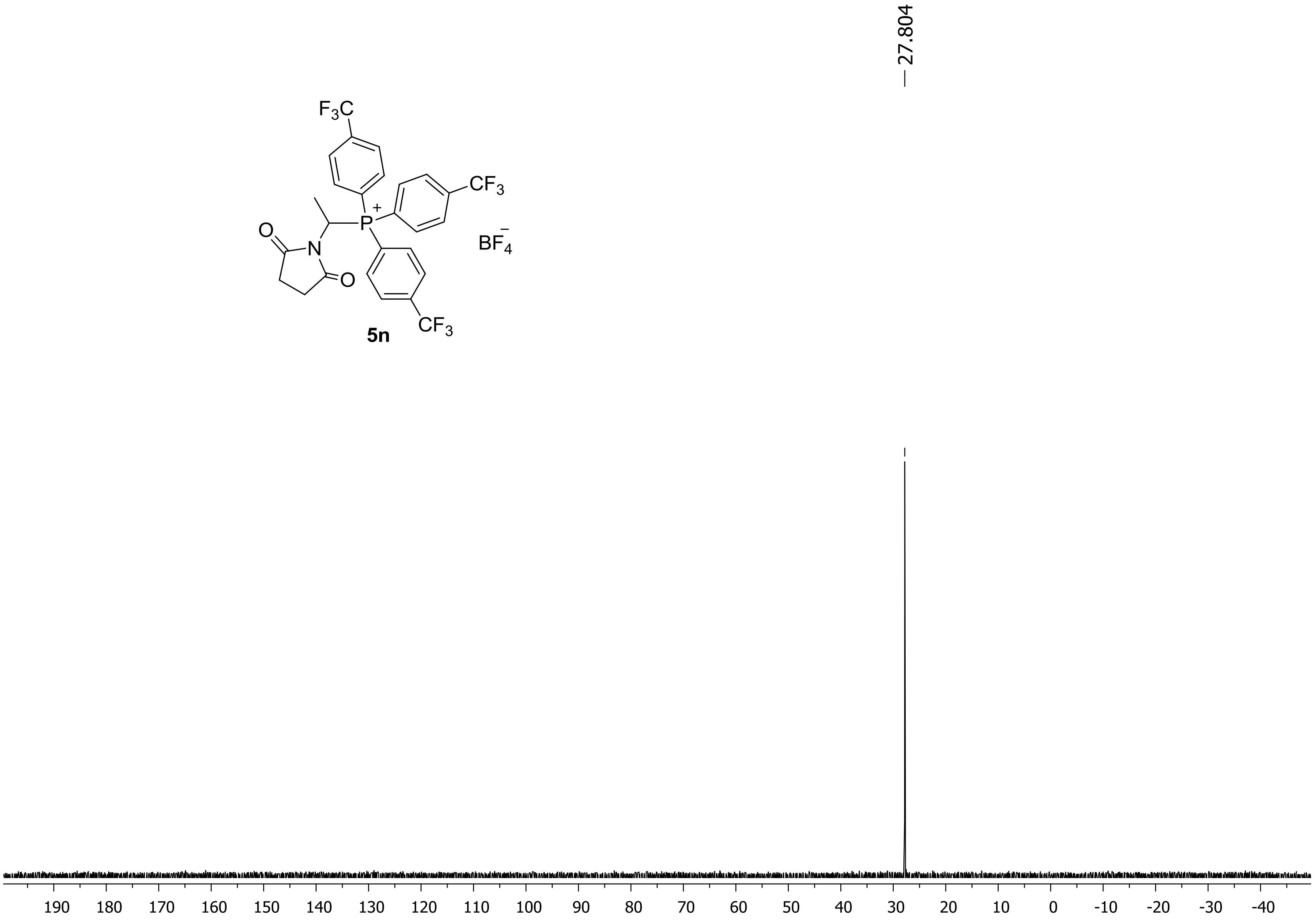

^{31}P NMR spectrum of 1-(*N*-succinimido)ethyltris(4-trifluoromethylphenyl)phosphonium tetrafluoroborate (**5n**); 161.9 MHz/CD_3CN; δ (ppm).

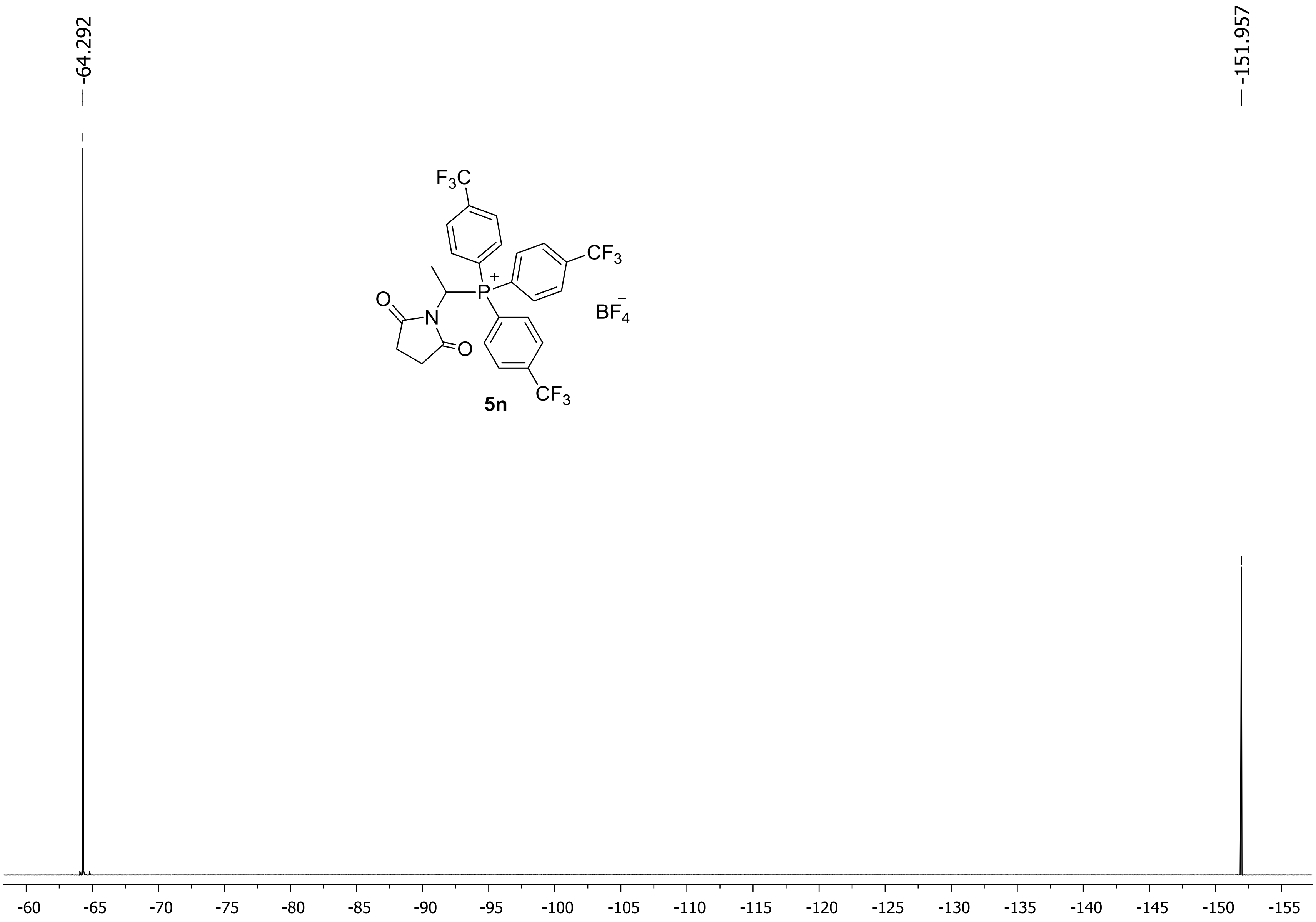

^{19}F NMR spectrum of 1-(*N*-succinimido)ethyltris(4-trifluoromethylphenyl)phosphonium tetrafluoroborate (**5n**); 376 MHz/CD_3CN; δ (ppm).

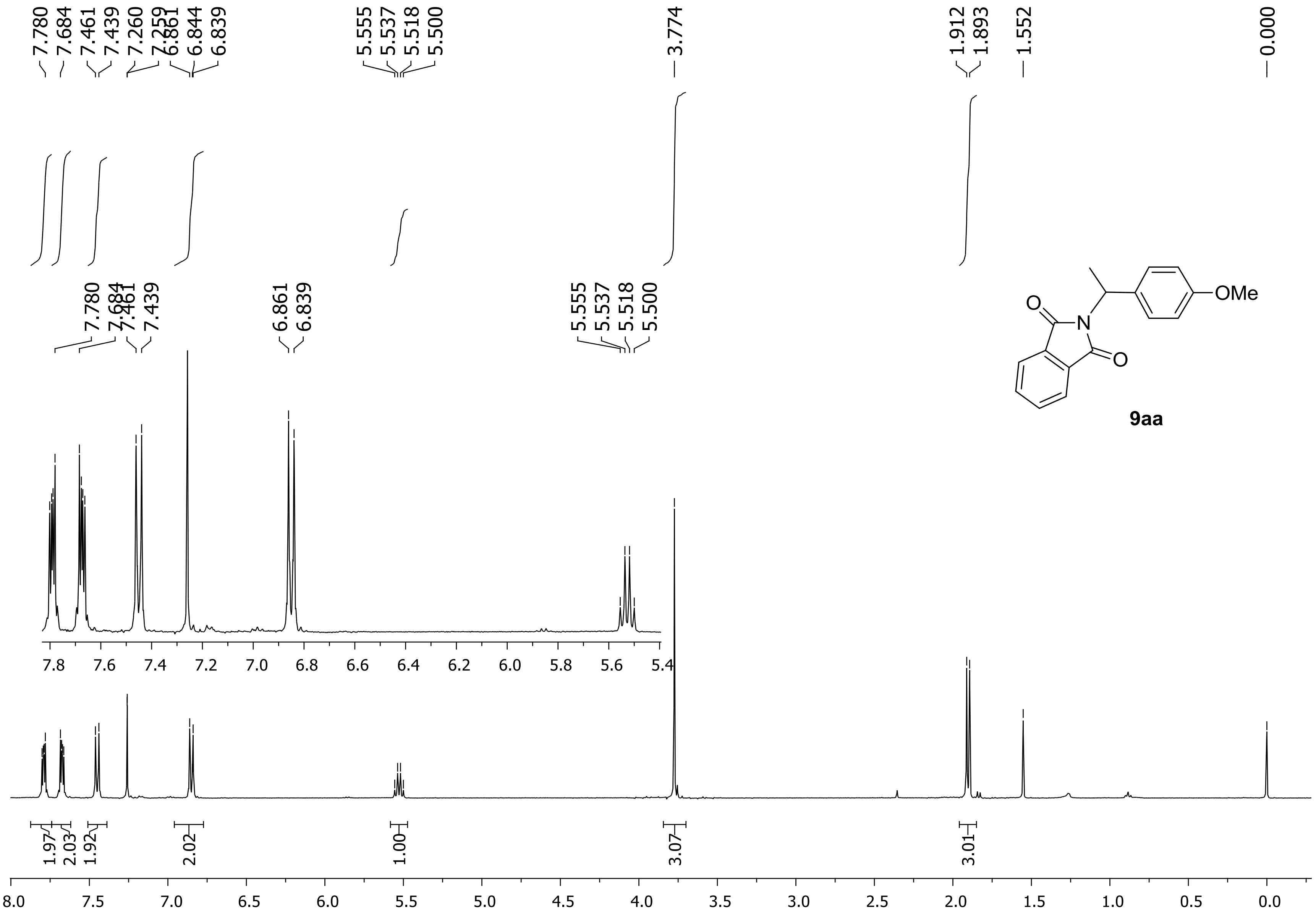

^{1}H NMR spectrum of *N*-[1-(4-methoxyphenyl)ethyl]phthalimide (**9aa**); 400 MHz/$CDCl_3$/TMS; δ (ppm).

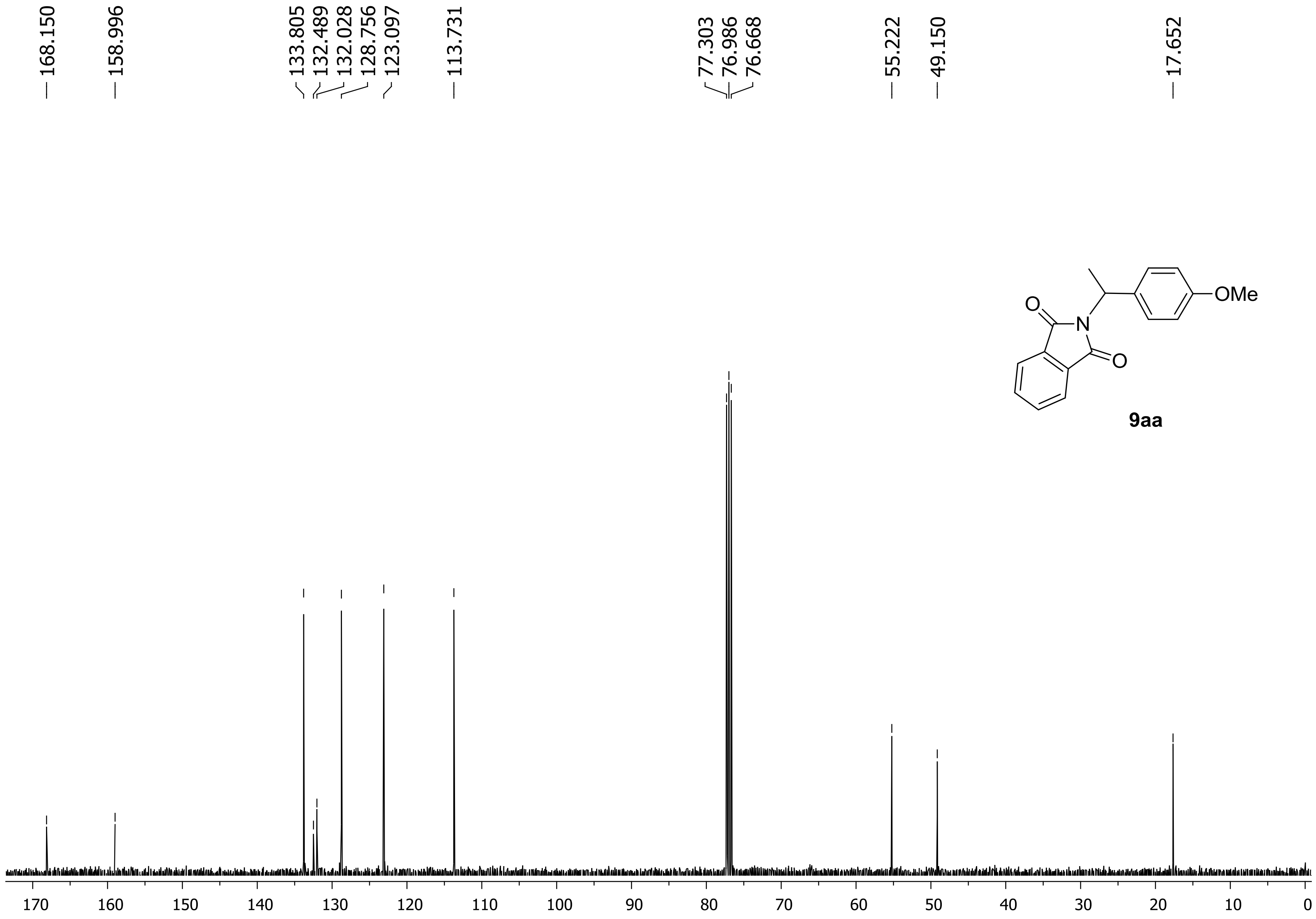

9aa

^{13}C NMR spectrum of *N*-[1-(4-methoxyphenyl)ethyl]phthalimide (**9aa**); 100 MHz/$CDCl_3$/TMS; δ (ppm).

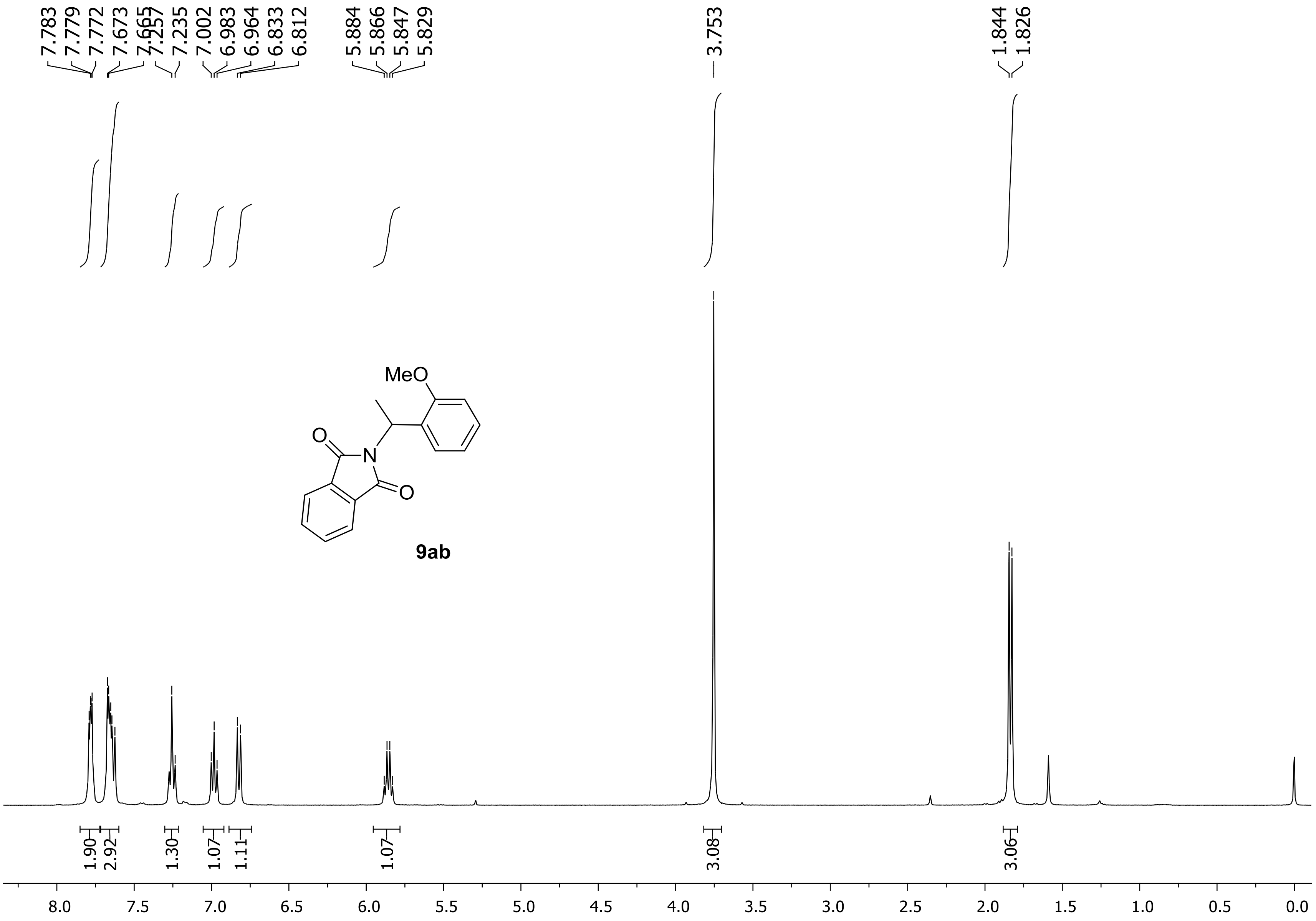

^{1}H NMR spectrum of *N*-[1-(2-methoxyphenyl)ethyl]phthalimide (**9ab**); 400 MHz/$CDCl_3$/TMS; δ (ppm).

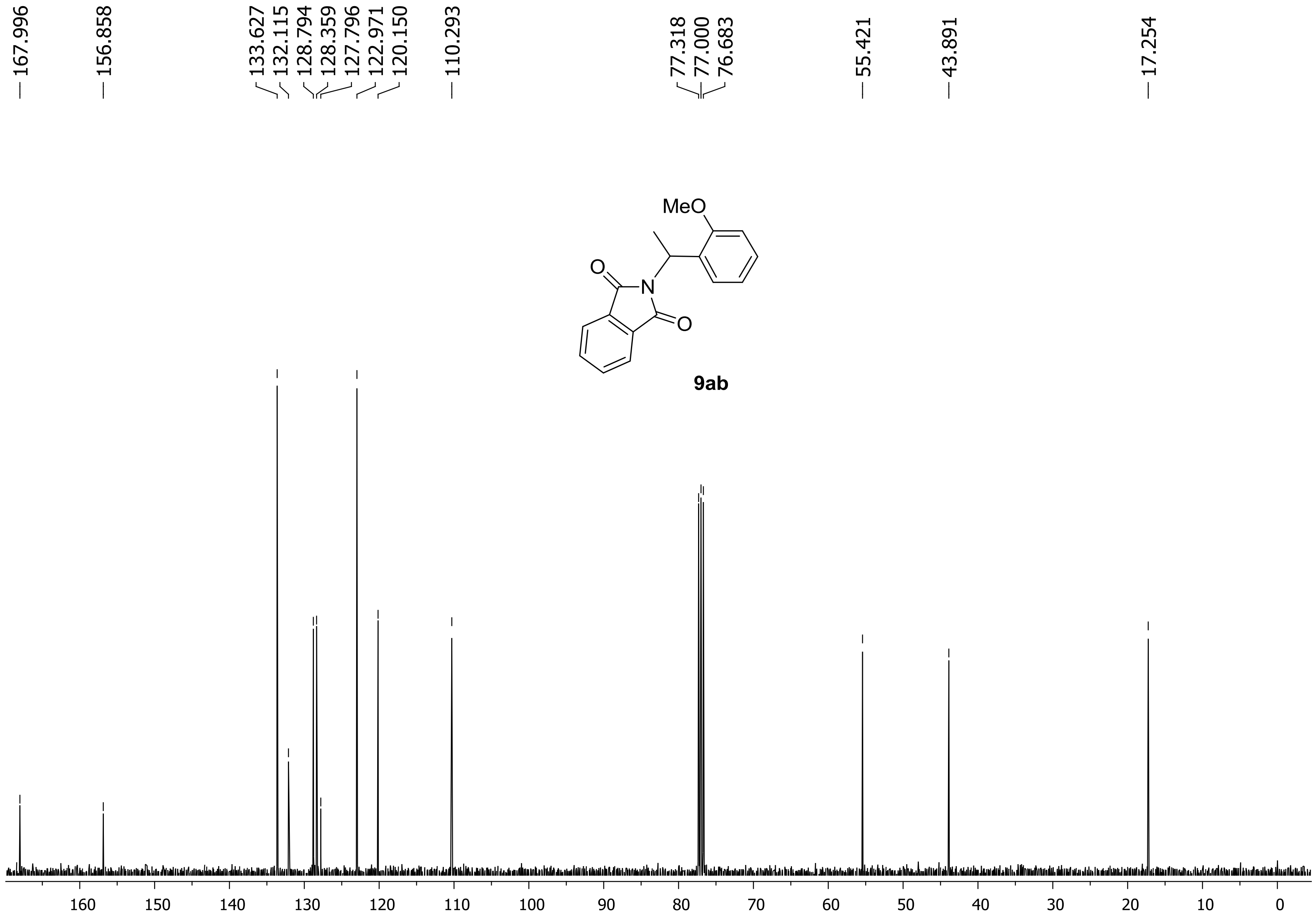

^{13}C NMR spectrum of *N*-[1-(2-methoxyphenyl)ethyl]phthalimide (**9ab**); 100 MHz/$CDCl_3$/TMS; δ (ppm).

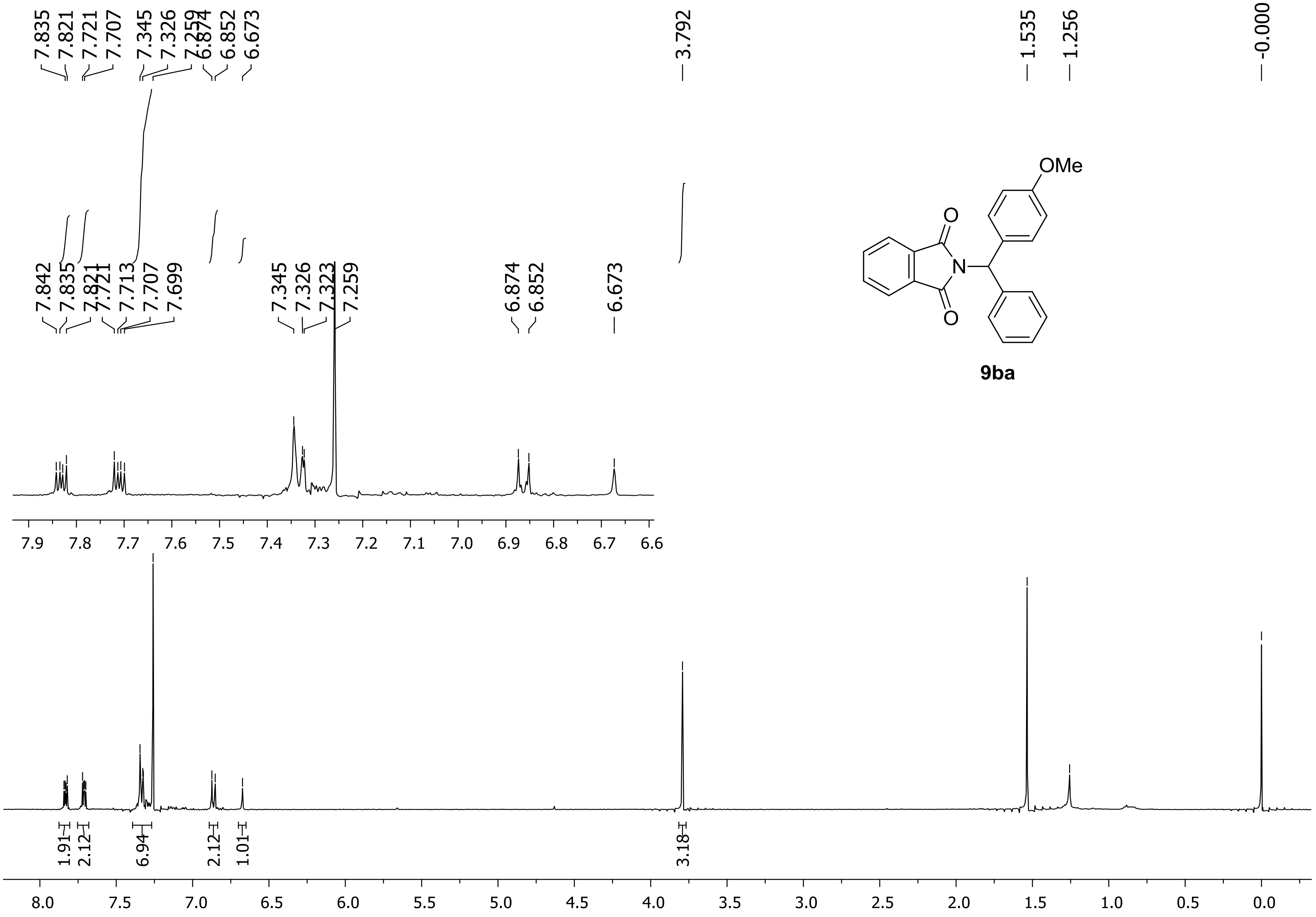

^{1}H NMR spectrum of *N*-[1-(4-methoxyphenyl)-1-phenylmethyl]phthalimide (**9ba**); 400 MHz/$CDCl_3$/TMS; δ (ppm).

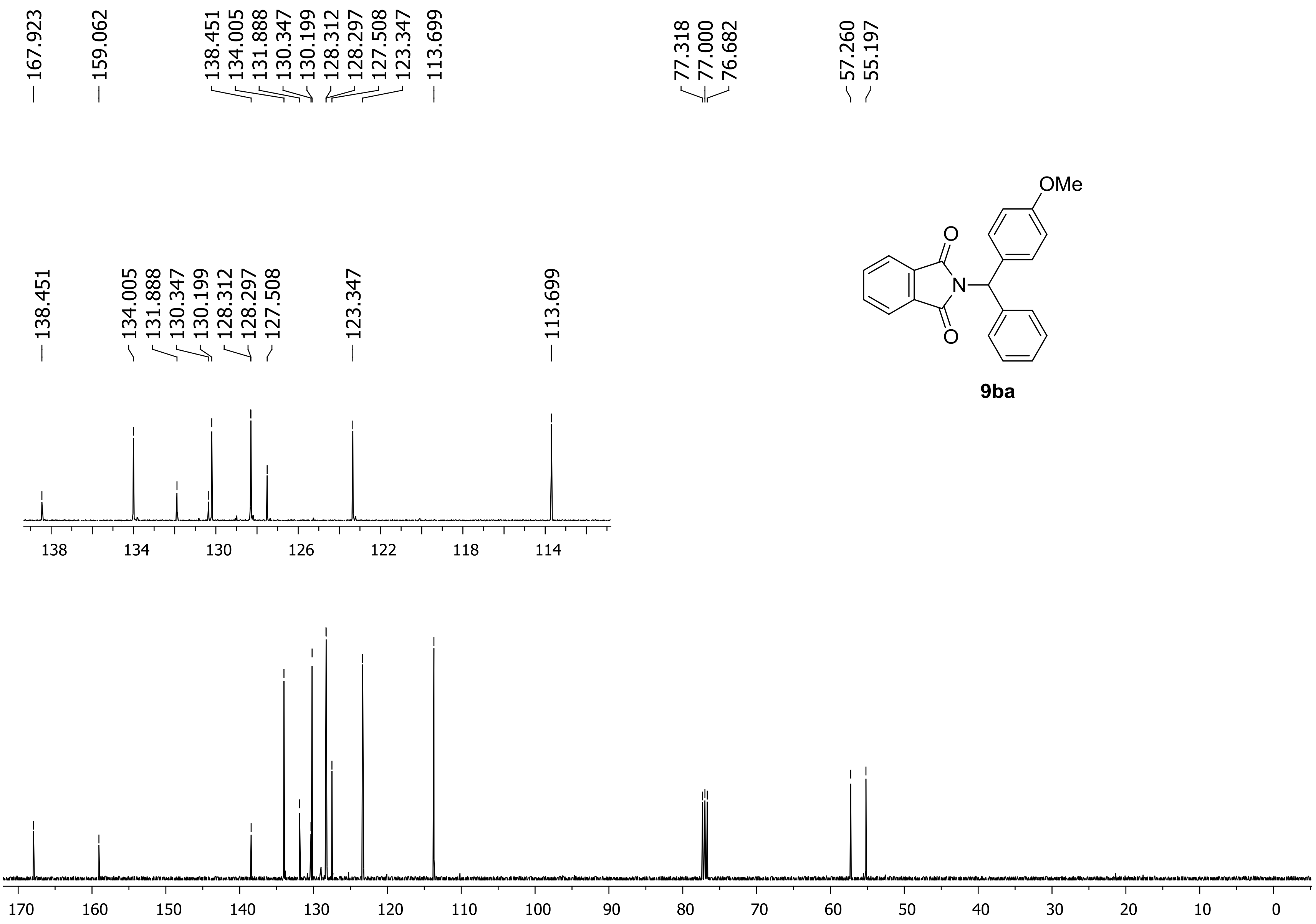

^{13}C NMR spectrum of *N*-[1-(4-methoxyphenyl)-1-phenylmethyl]phthalimide (**9ba**); 100 MHz/$CDCl_3$/TMS; δ (ppm).

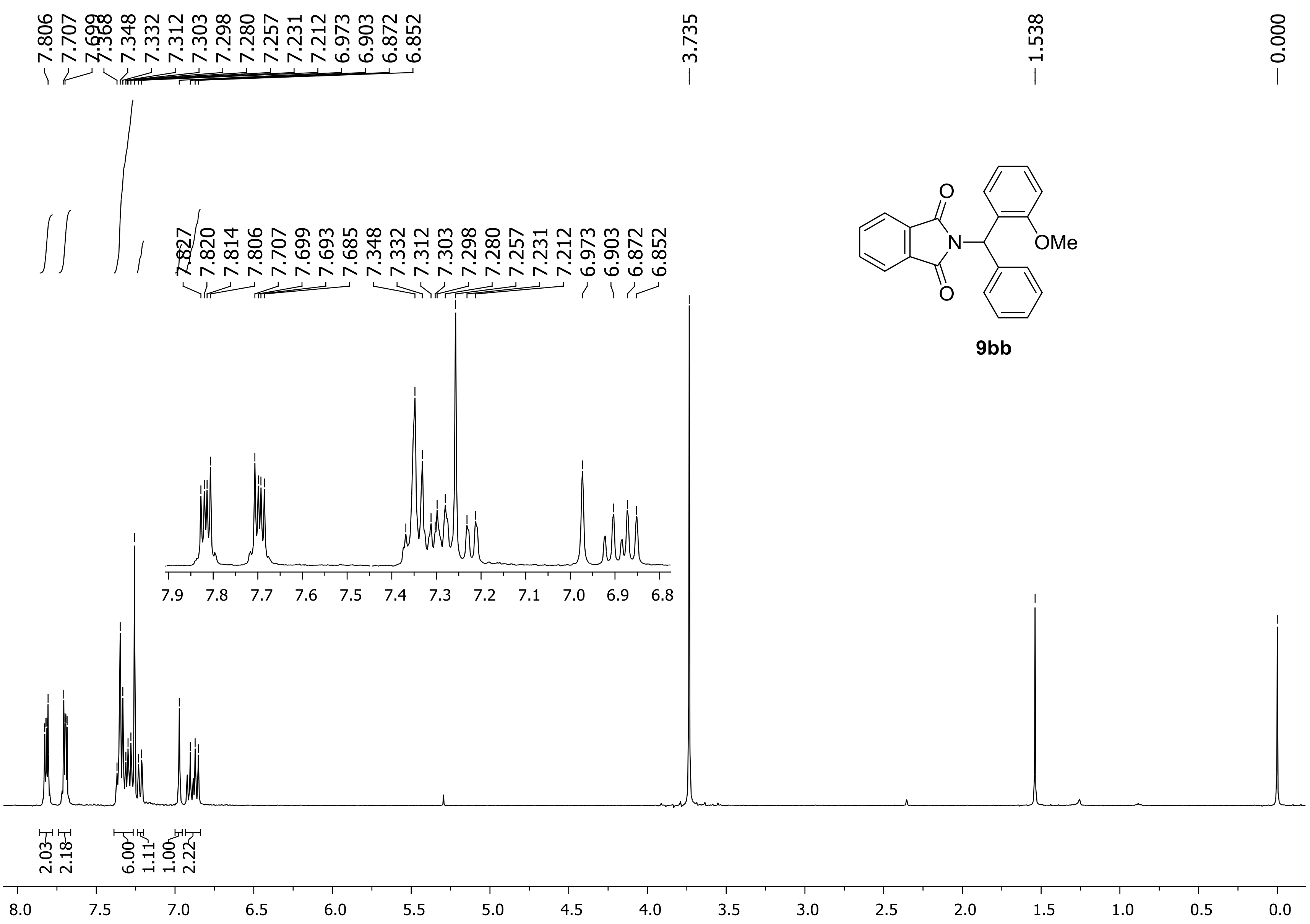

9bb

^{1}H NMR spectrum of *N*-[1-(2-methoxyphenyl)-1-phenylmethyl]phthalimide (**9bb**); 400 MHz/$CDCl_3$/TMS; δ (ppm).

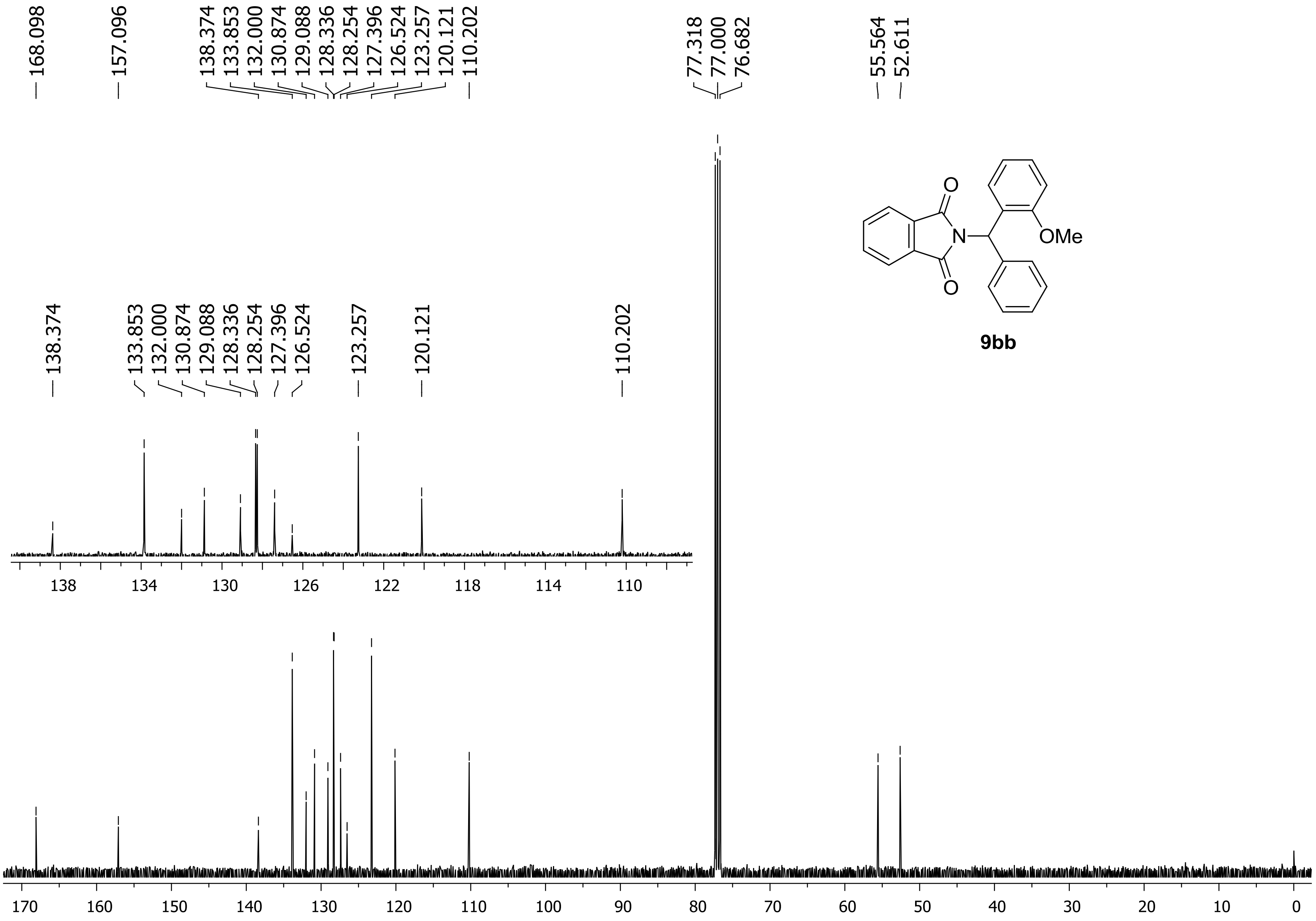

^{13}C NMR spectrum of *N*-[1-(2-methoxyphenyl)-1-phenylmethyl]phthalimide (**9bb**); 100 MHz/$CDCl_3$/TMS; δ (ppm).

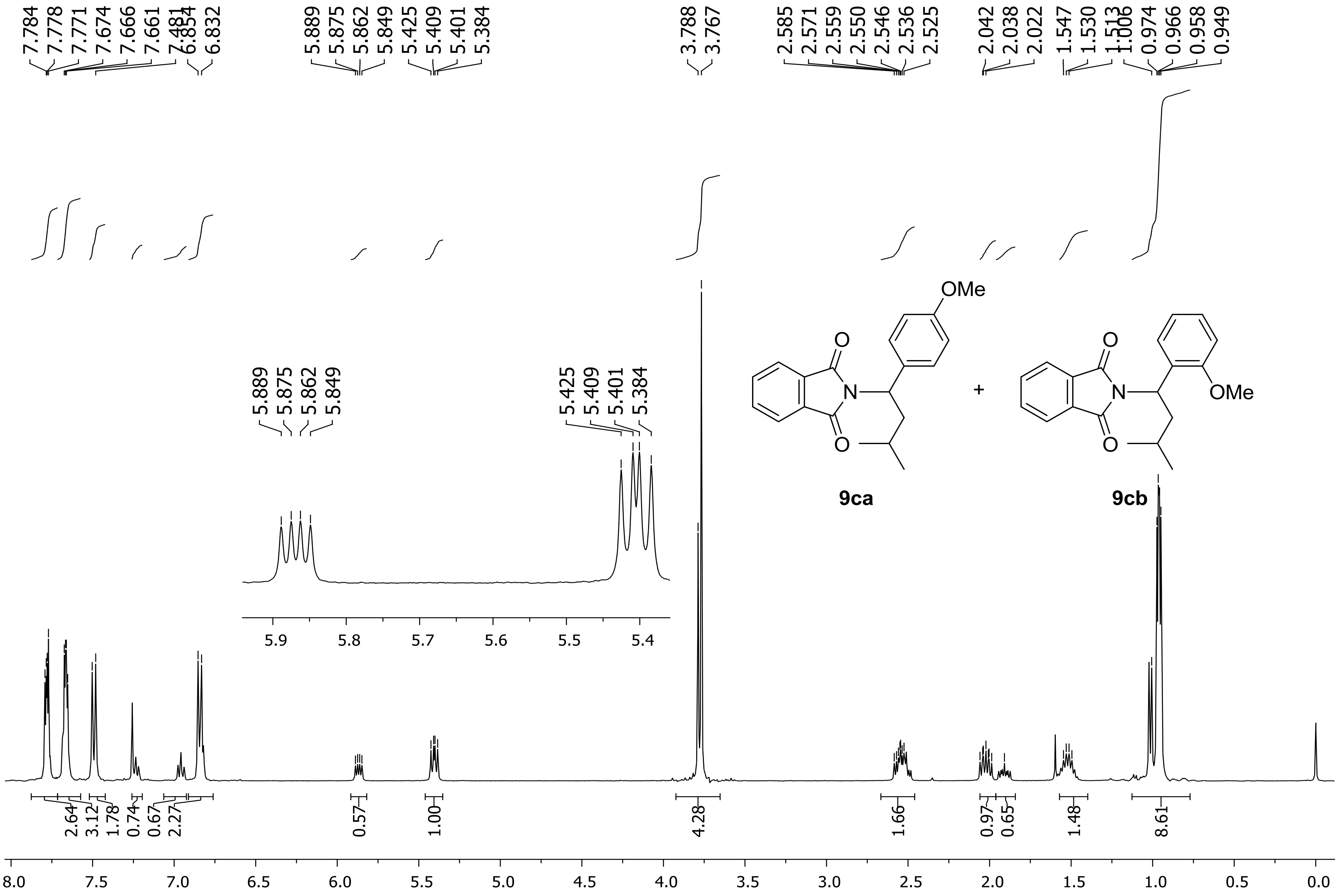

^{1}H NMR spectrum of mixture *N*-[1-(4-methoxyphenyl)-3-methylbutyl]phthalimide **(9ca)** and *N*-[1-(2-methoxyphenyl)-3-methylbutyl]phthalimide **(9cb)**; 400 MHz/$CDCl_3$/TMS; *δ* (ppm).

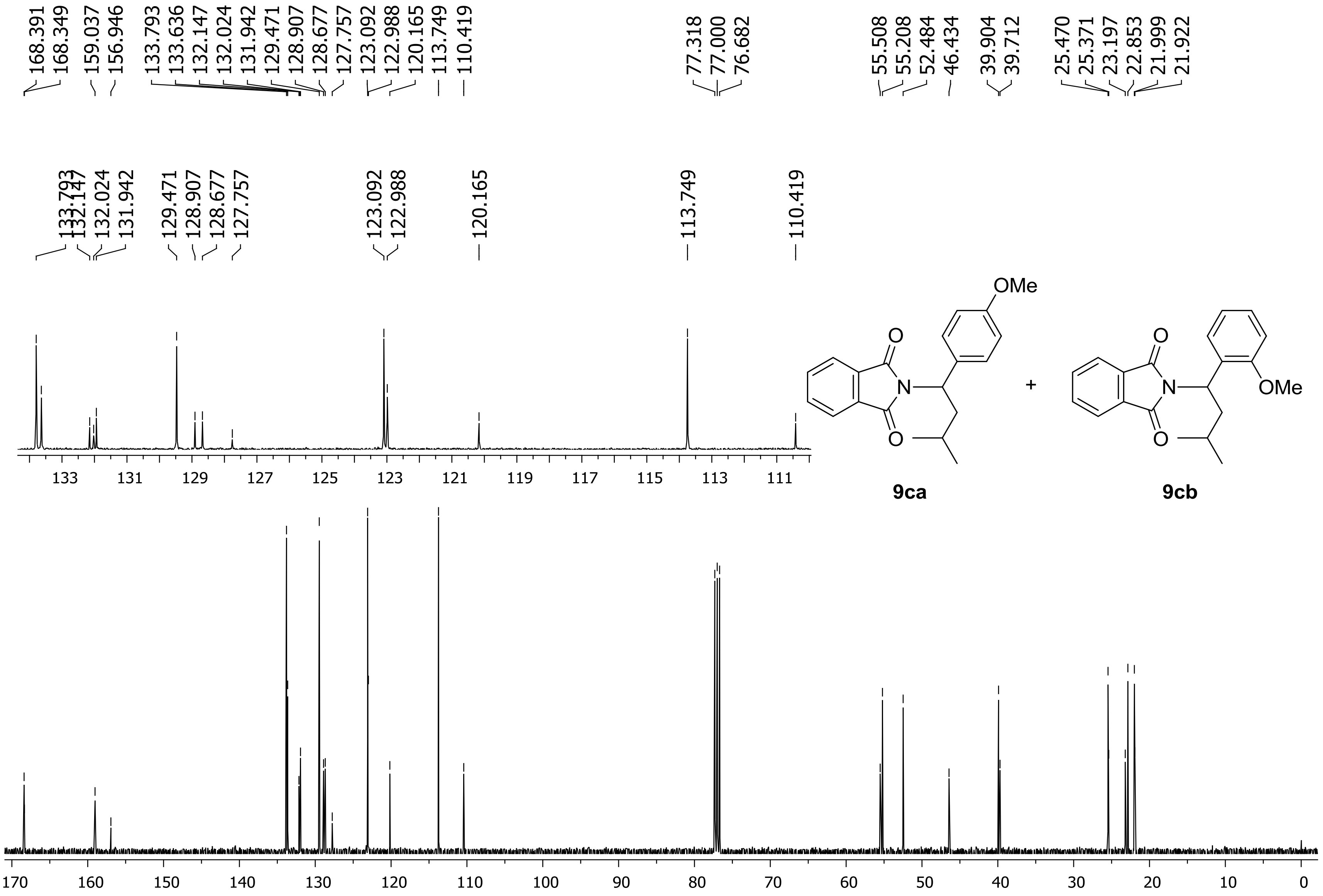

^{13}C NMR spectrum of mixture *N*-[1-(4-methoxyphenyl)-3-methylbutyl]phthalimide **(9ca)** and *N*-[1-(2-methoxyphenyl)-3-methylbutyl]phthalimide **(9cb)**; 100 MHz/$CDCl_3$/TMS; δ (ppm).

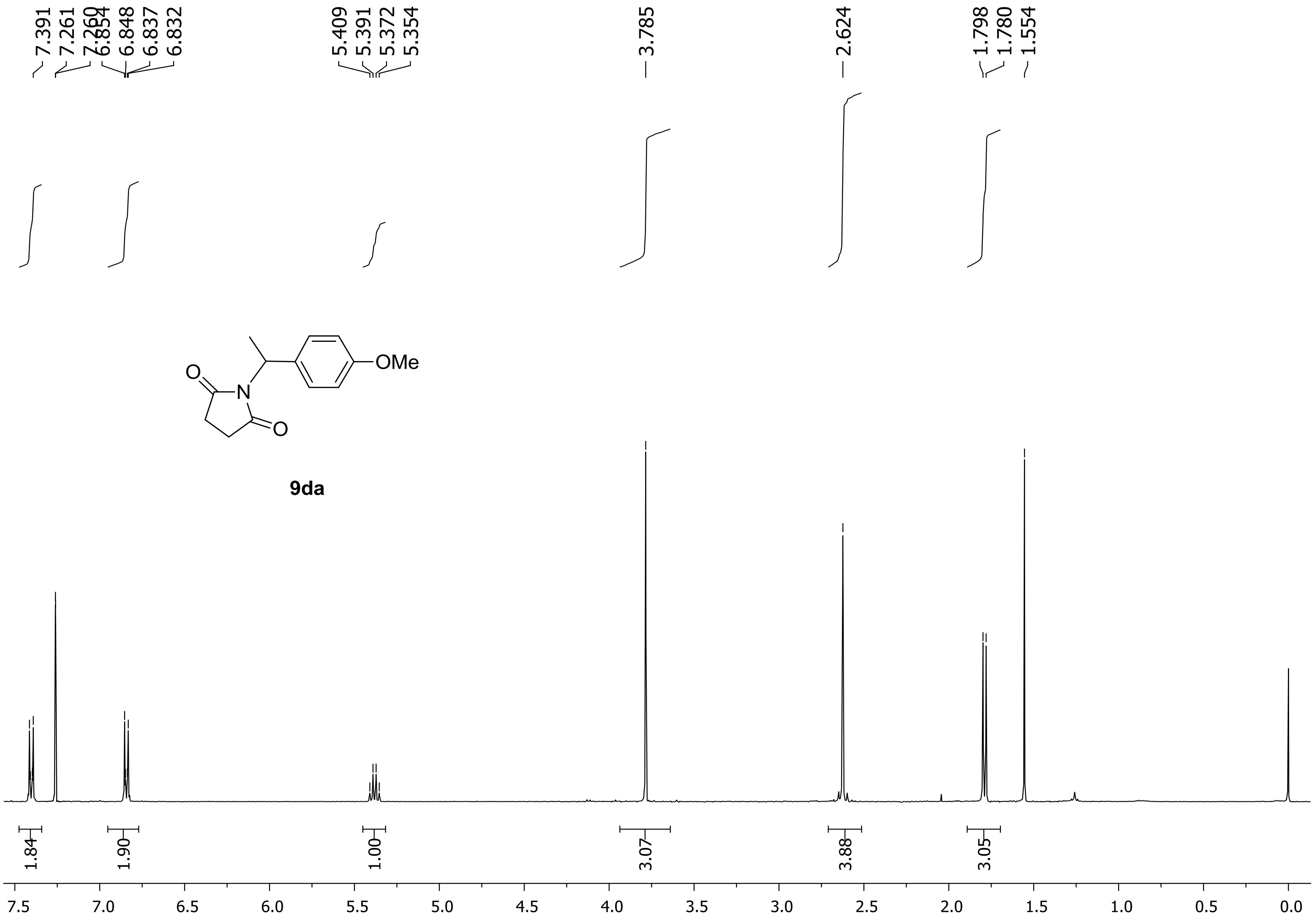

^{1}H NMR spectrum of *N*-[1-(4-methoxyphenyl)ethyl]succinimide (**9da**); 400 MHz/$CDCl_3$/TMS; δ (ppm).

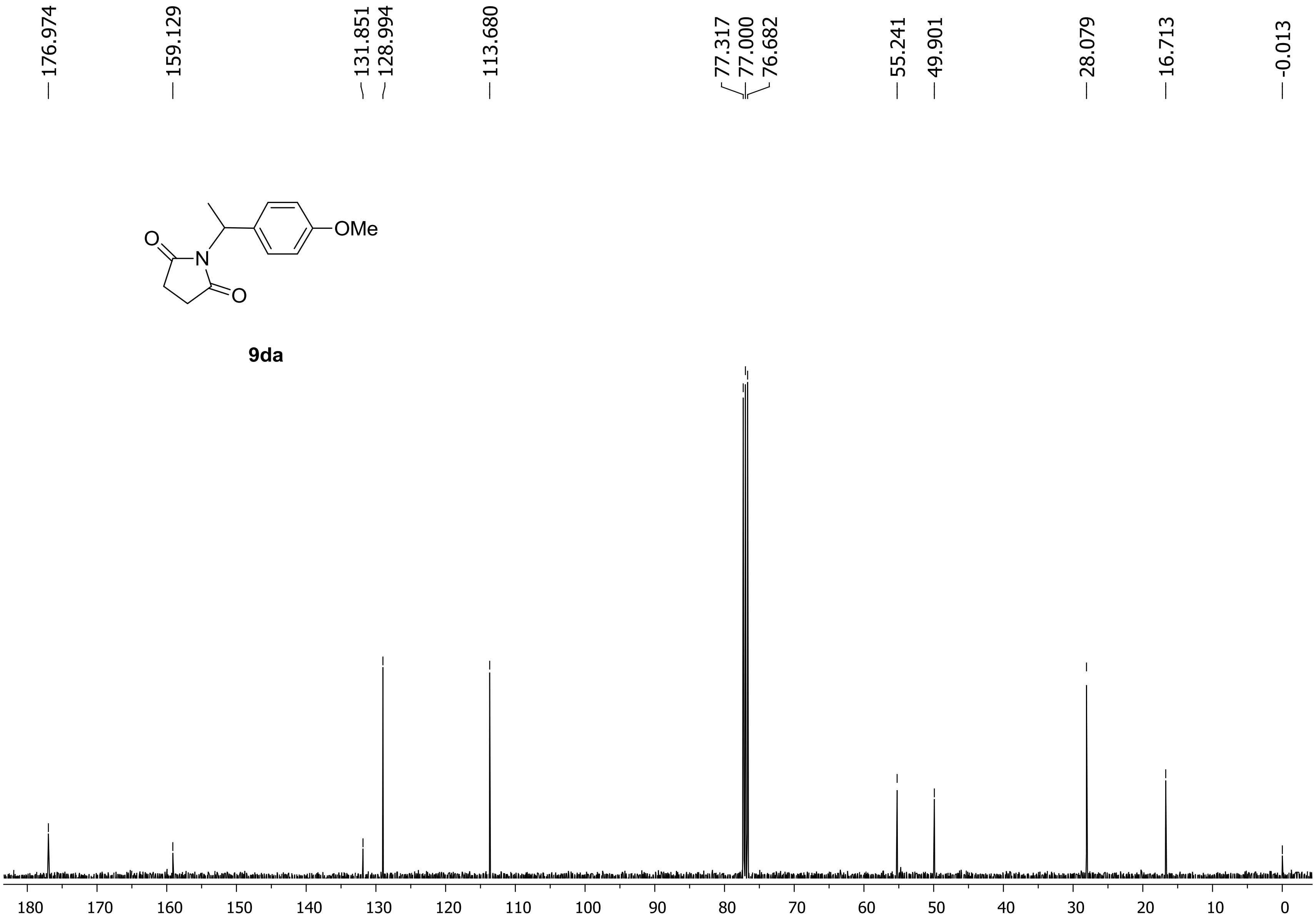

^{13}C NMR spectrum of *N*-[1-(4-methoxyphenyl)ethyl]succinimide (**9da**); 100 MHz/$CDCl_3$/TMS; δ (ppm).

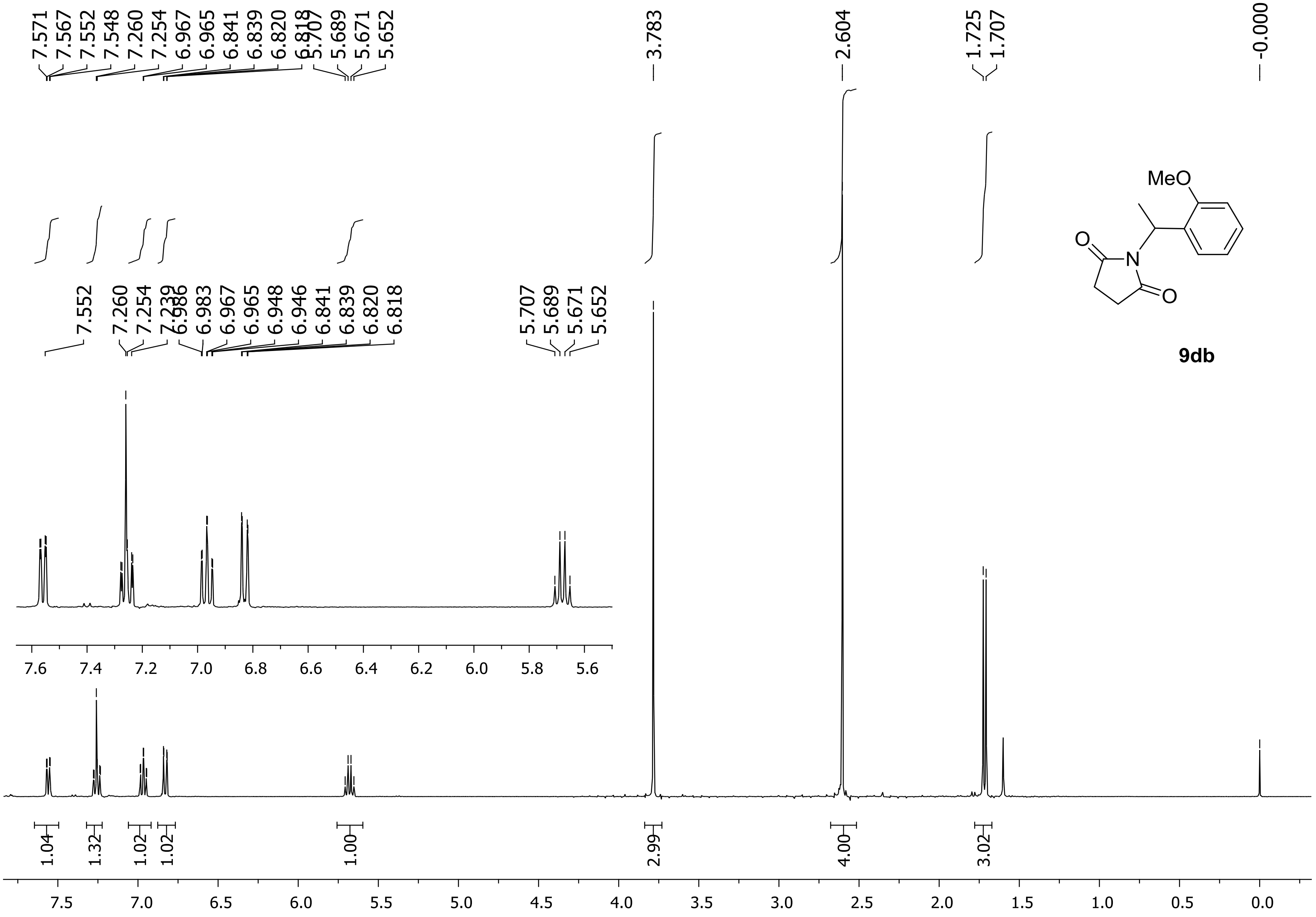

^{1}H NMR spectrum of *N*-[1-(2-methoxyphenyl)ethyl]succinimide (**9db**); 400 MHz/$CDCl_3$/TMS; δ (ppm).

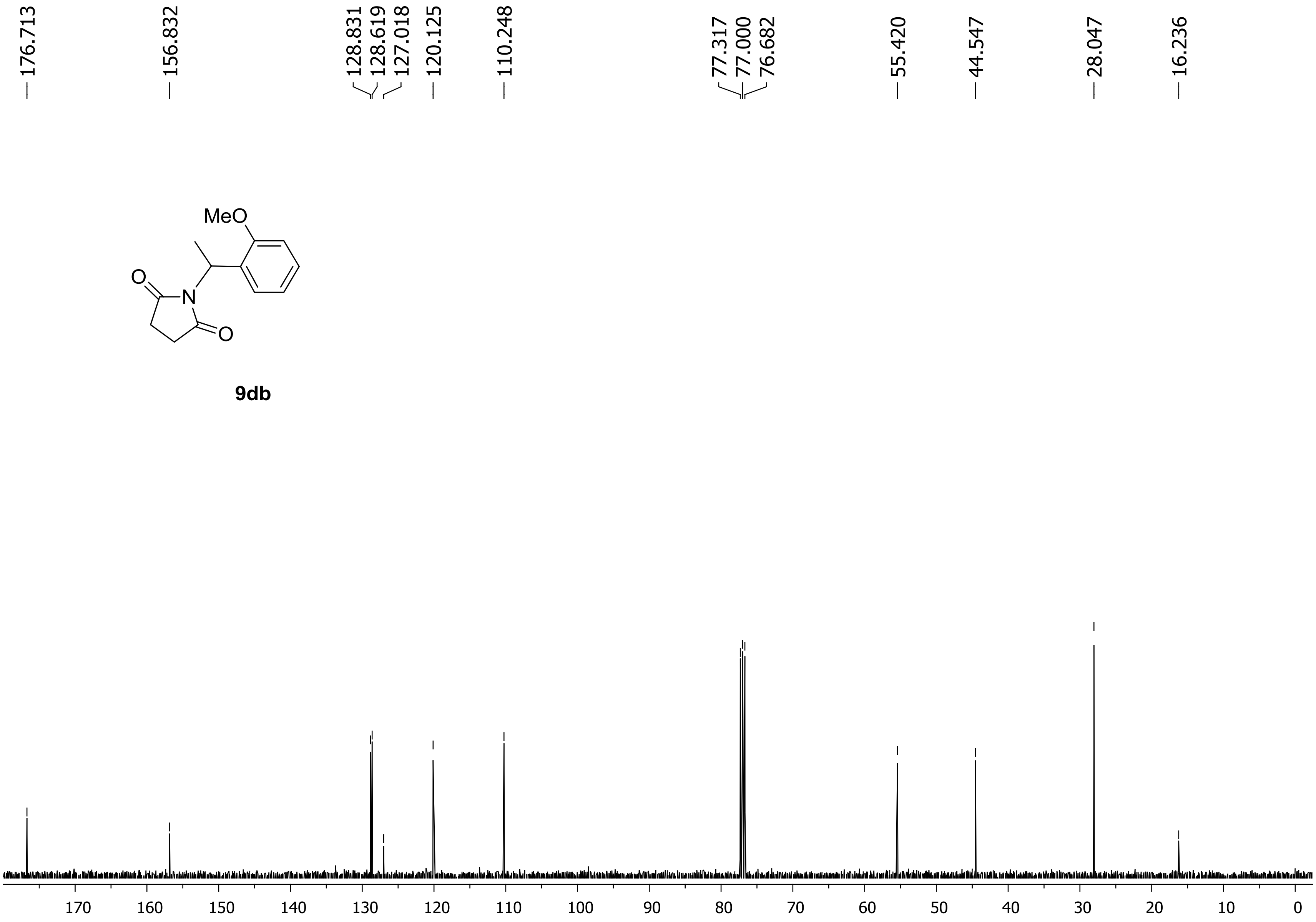

^{13}C NMR spectrum of *N*-[1-(2-methoxyphenyl)ethyl]succinimide (**9db**); 100 MHz/$CDCl_3$/TMS; δ (ppm).

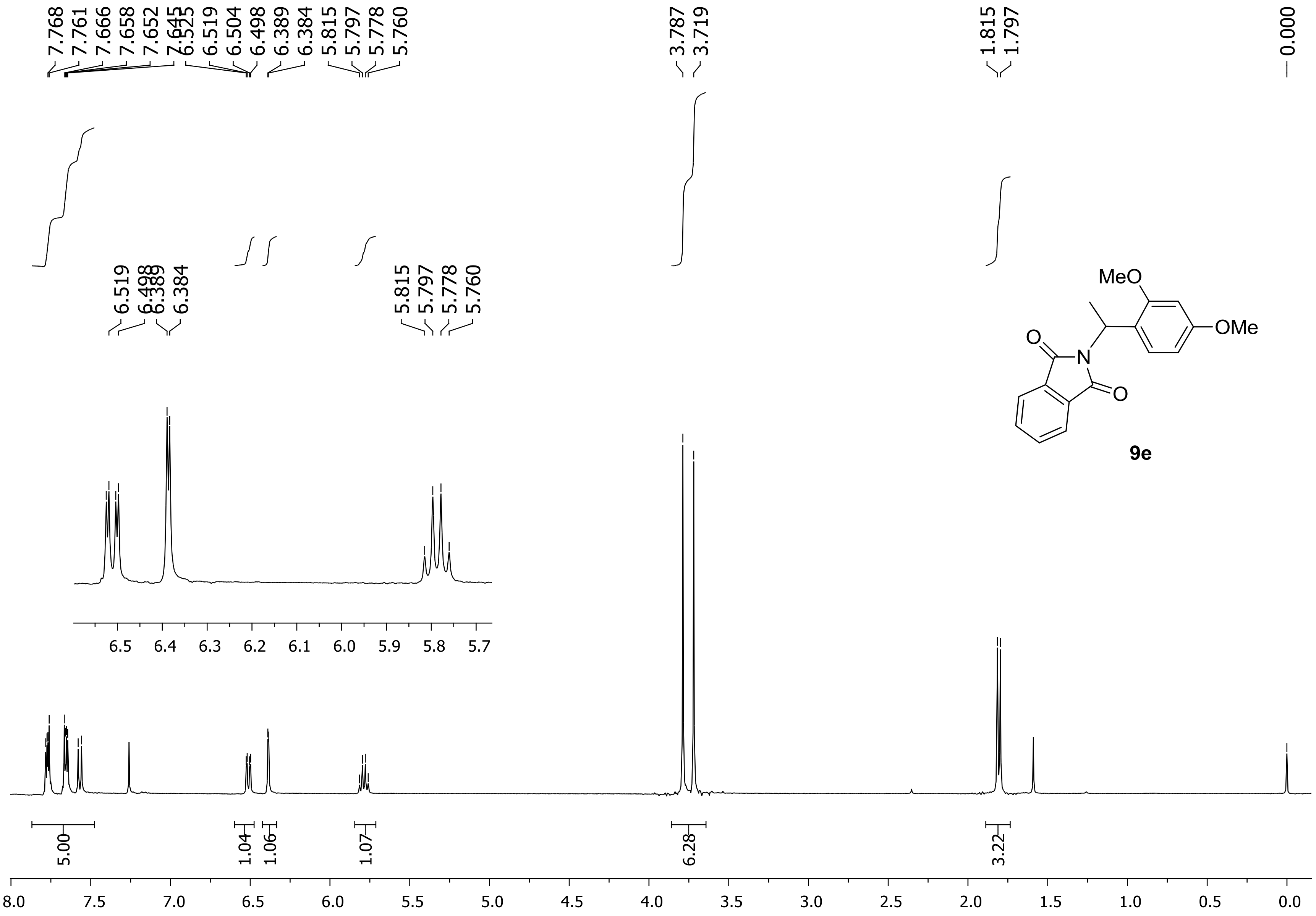

^{1}H NMR spectrum of *N*-[1-(2,4-dimethoxyphenyl)ethyl]phthalimide (**9e**); 400 MHz/$CDCl_3$/TMS; δ (ppm).

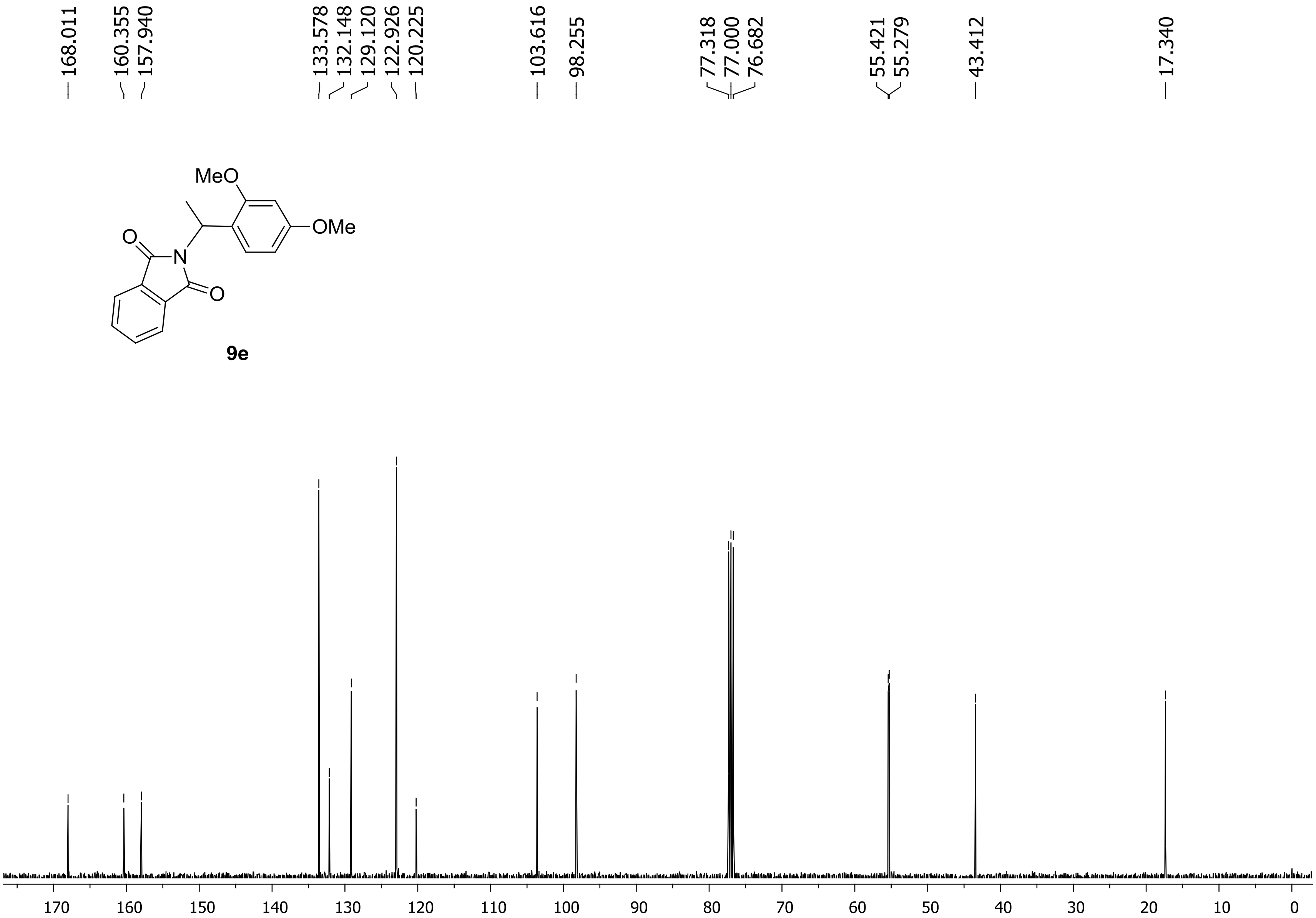

^{13}C NMR spectrum of *N*-[1-(2,4-dimethoxyphenyl)ethyl]phthalimide (**9e**); 100 MHz/$CDCl_3$/TMS; δ (ppm).

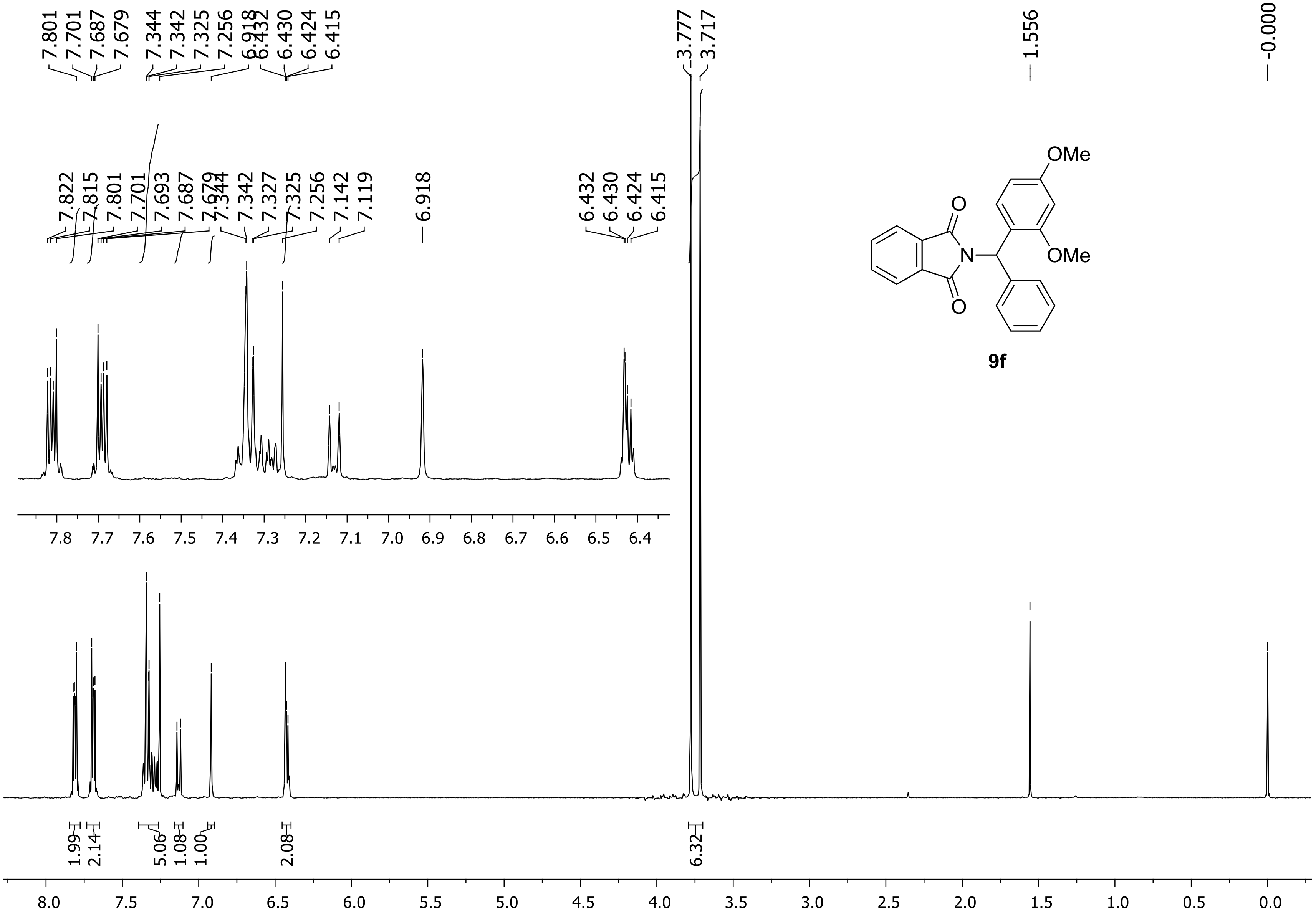

^{1}H NMR spectrum of *N*-[1-(2,4-dimethoxyphenyl)-1-phenylmethyl]phthalimide (**9f**); 400 MHz/$CDCl_3$/TMS; δ (ppm).

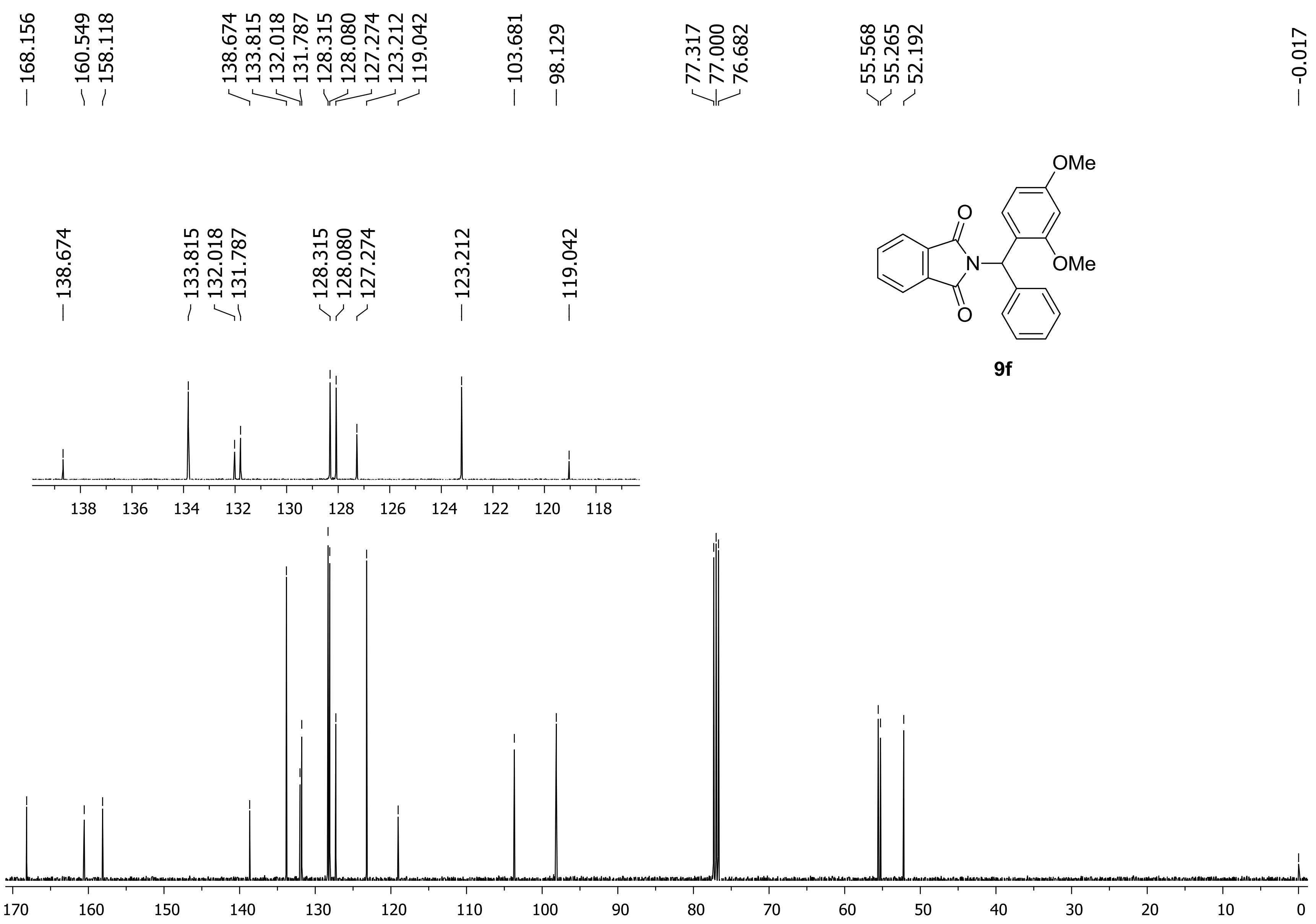

^{13}C NMR spectrum of *N*-[1-(2,4-dimethoxyphenyl)-1-phenylmethyl]phthalimide (**9f**); 100 MHz/$CDCl_3$/TMS; δ (ppm).

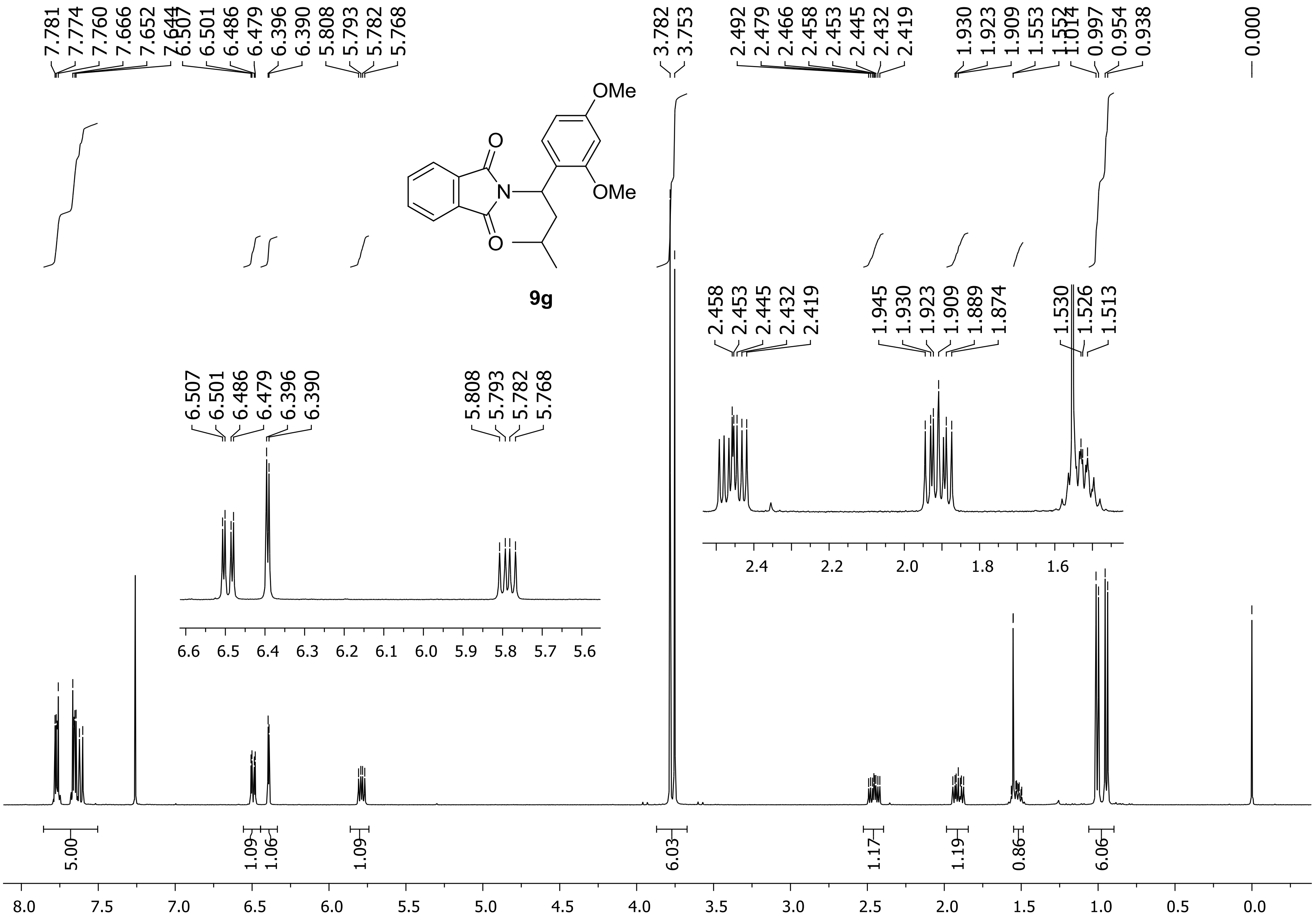

^{1}H NMR spectrum of *N*-[1-(2,4-dimethoxyphenyl)-3-methylbutyl]phthalimide (**9g**); 400 MHz/$CDCl_3$/TMS; δ (ppm).

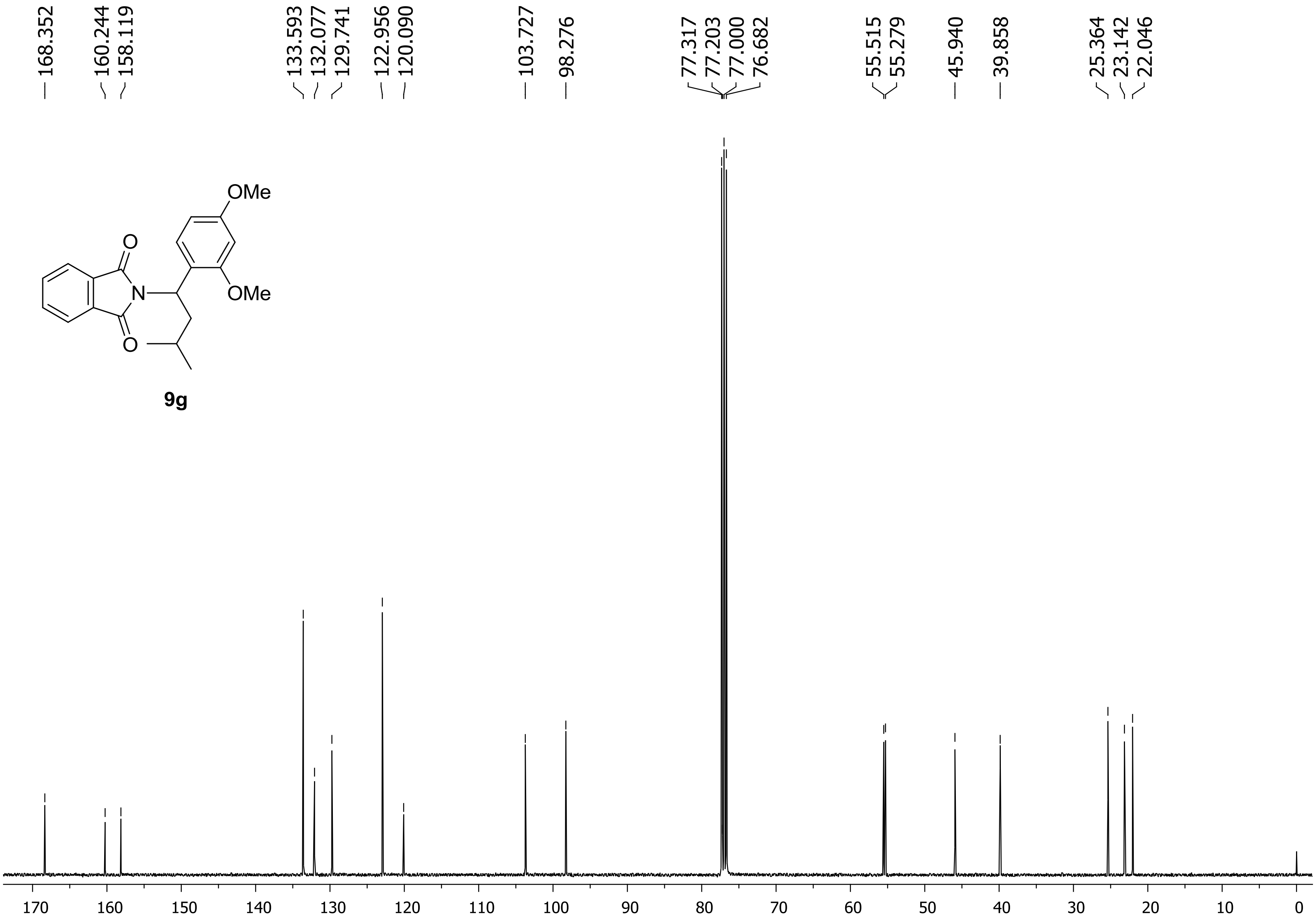

^{13}C NMR spectrum of *N*-[1-(2,4-dimethoxyphenyl)-3-methylbutyl]phthalimide (**9g**); 100 MHz/$CDCl_3$/TMS; δ (ppm).

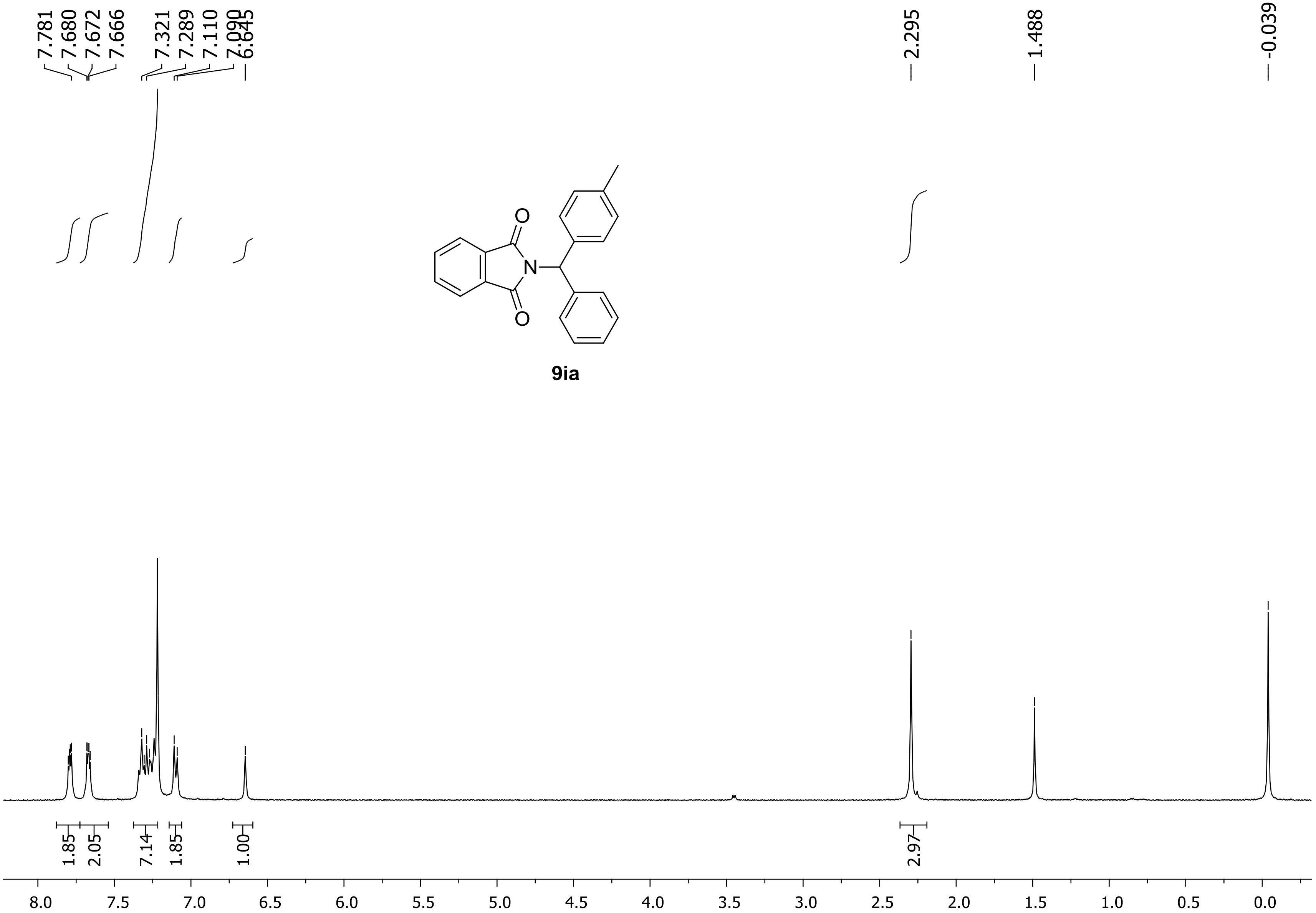

^{1}H NMR spectrum of *N*-[1-(4-methylphenyl)-1-phenylmethyl]phthalimide (**9ia**); 400 MHz/$CDCl_3$/TMS; δ (ppm).

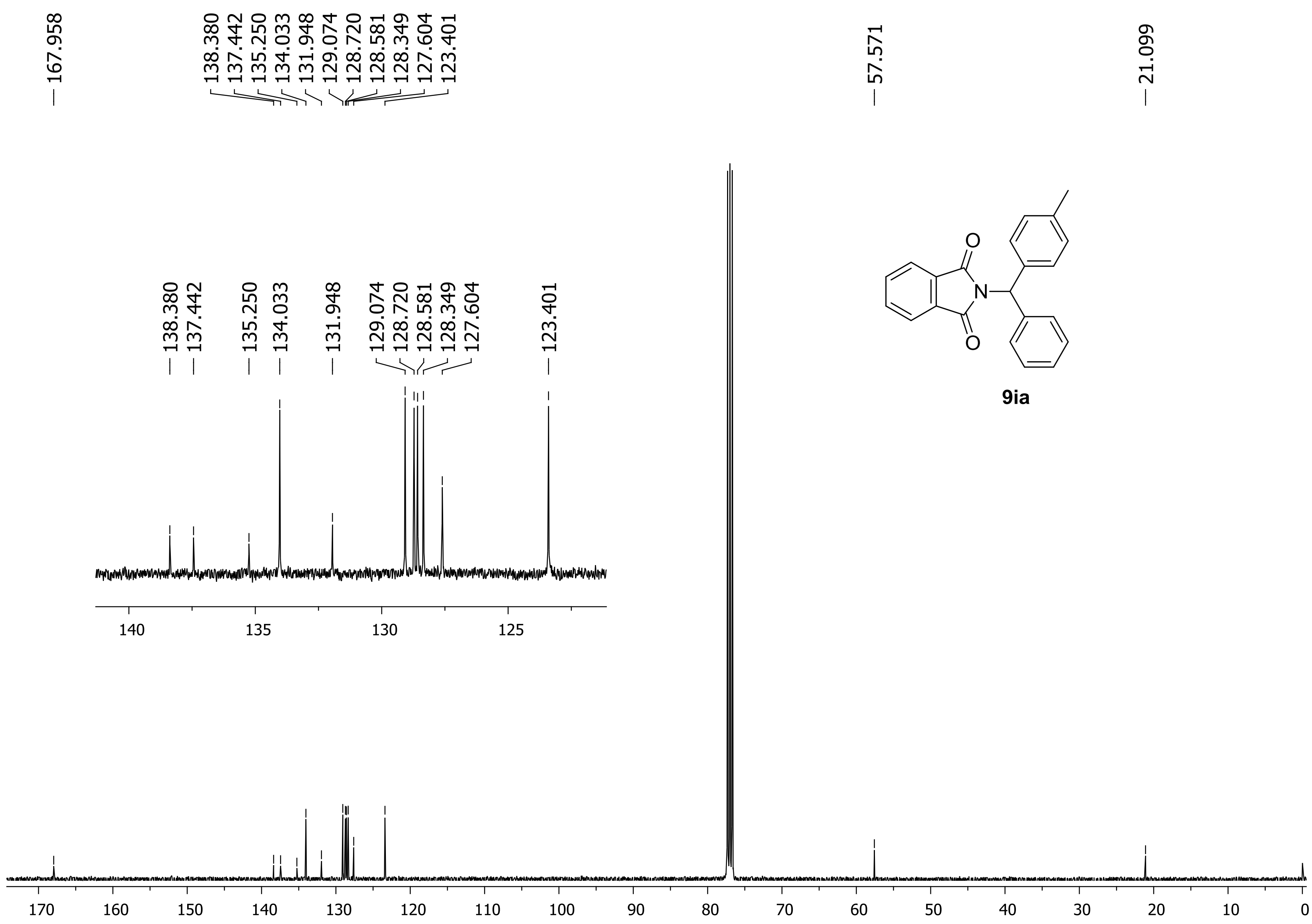

^{13}C NMR spectrum of *N*-[1-(4-methylphenyl)-1-phenylmethyl]phthalimide (**9ia**); 100 MHz/$CDCl_3$/TMS; δ (ppm).

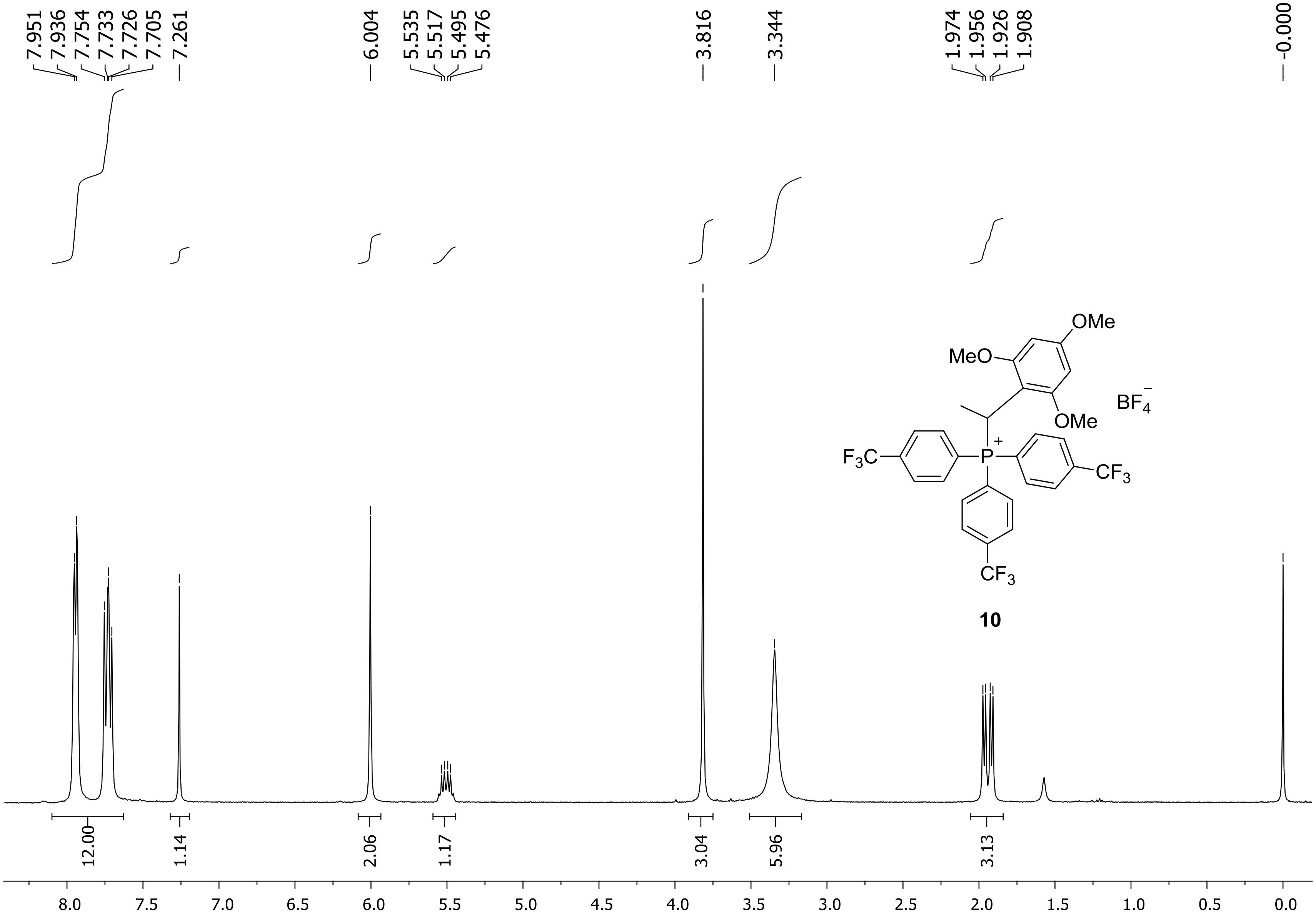

^{1}H NMR spectrum of 1-(2,4,6-trimethoxyphenyl)ethyltris(4-trifluoromethylphenyl)phosphonium tetrafluoroborate (**10**); 400 MHz/$CDCl_3$/TMS; δ (ppm).

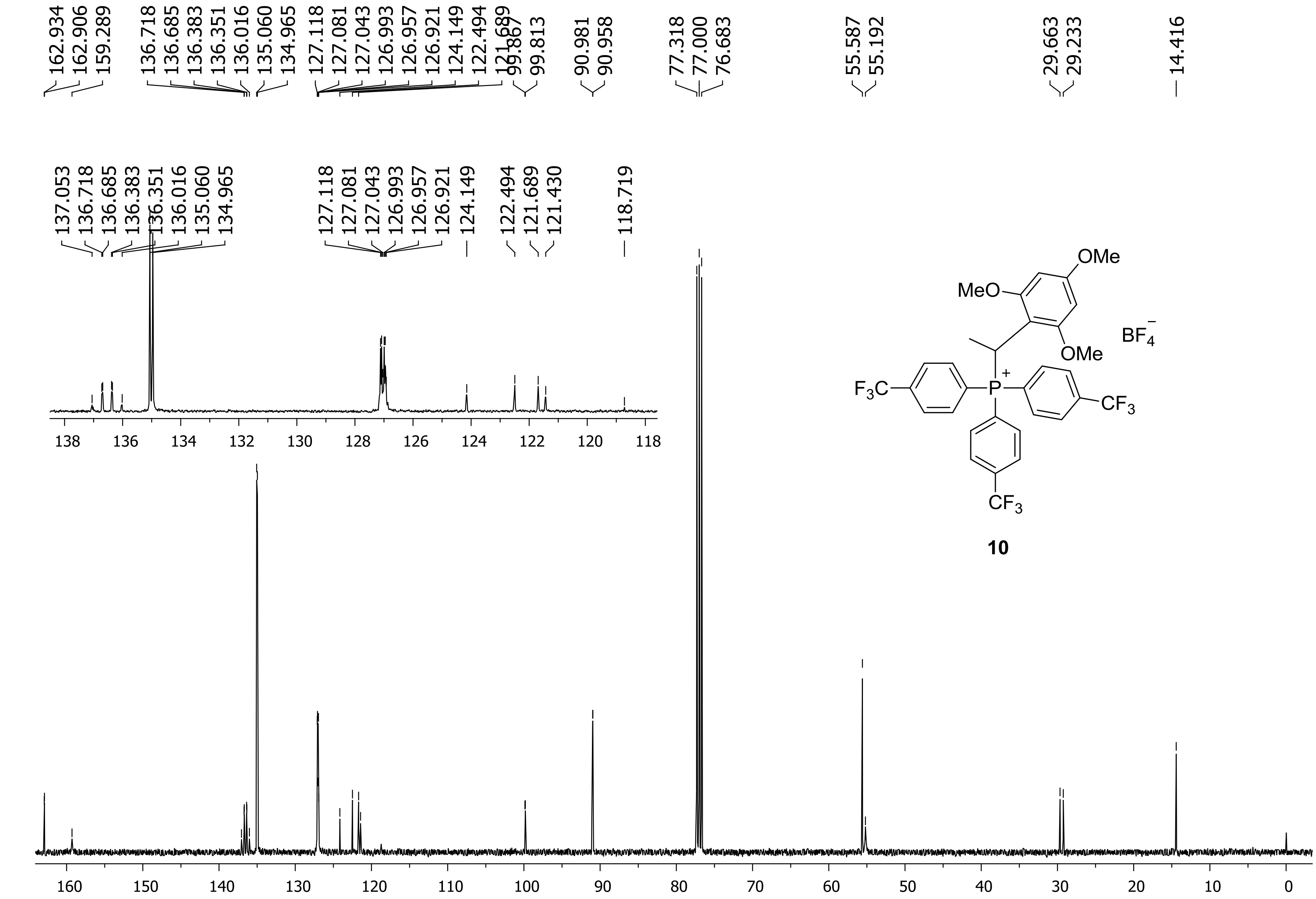

^{13}C NMR spectrum of 1-(2,4,6-trimethoxyphenyl)ethyltris(4-trifluoromethylphenyl)phosphonium tetrafluoroborate (**10**); 100 MHz/$CDCl_3$/TMS; δ (ppm).

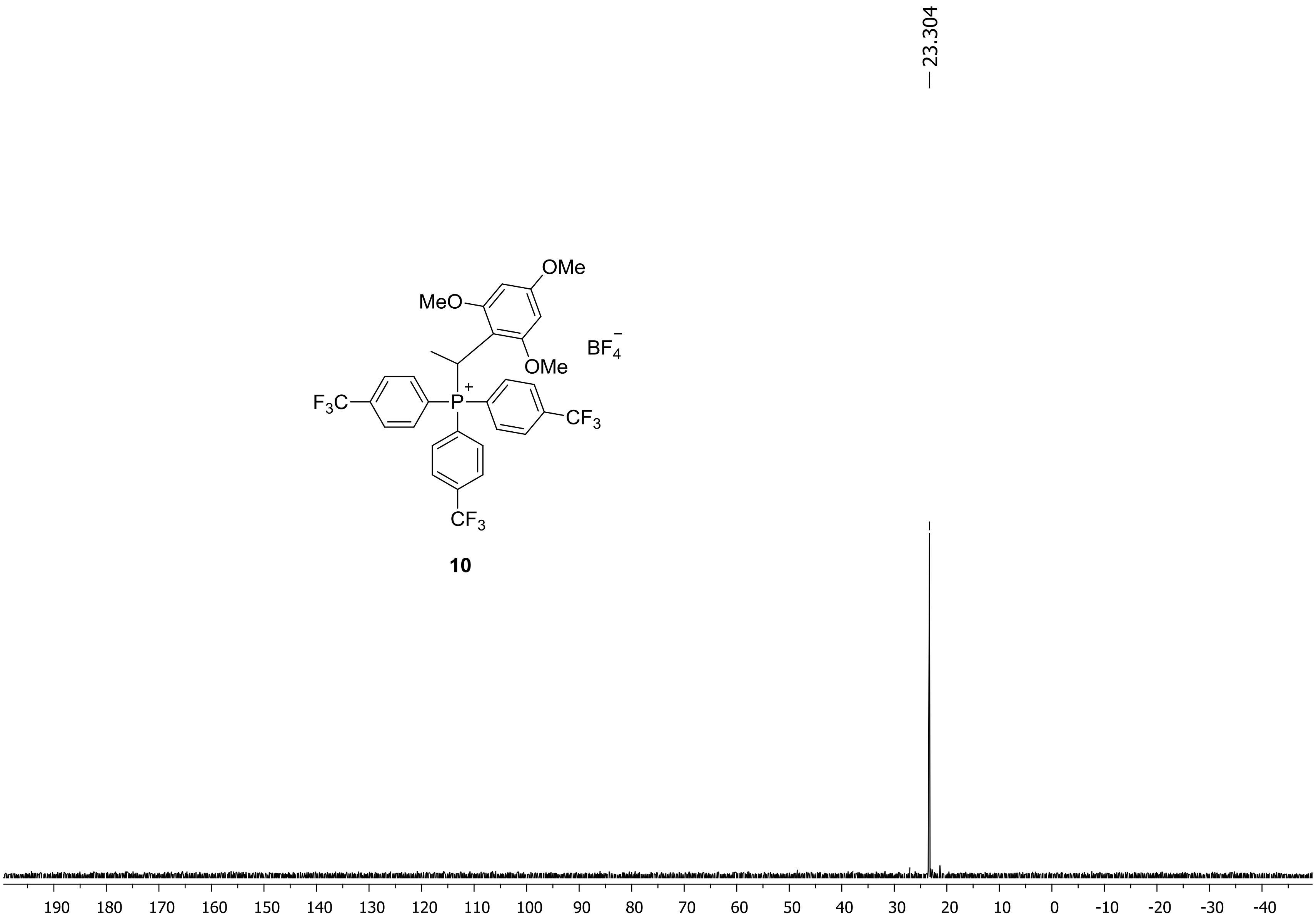

^{31}P NMR spectrum of 1-(2,4,6-trimethoxyphenyl)ethyltris(4-trifluoromethylphenyl)phosphonium tetrafluoroborate (**10**); 161.9 MHz/$CDCl_3$; δ (ppm).

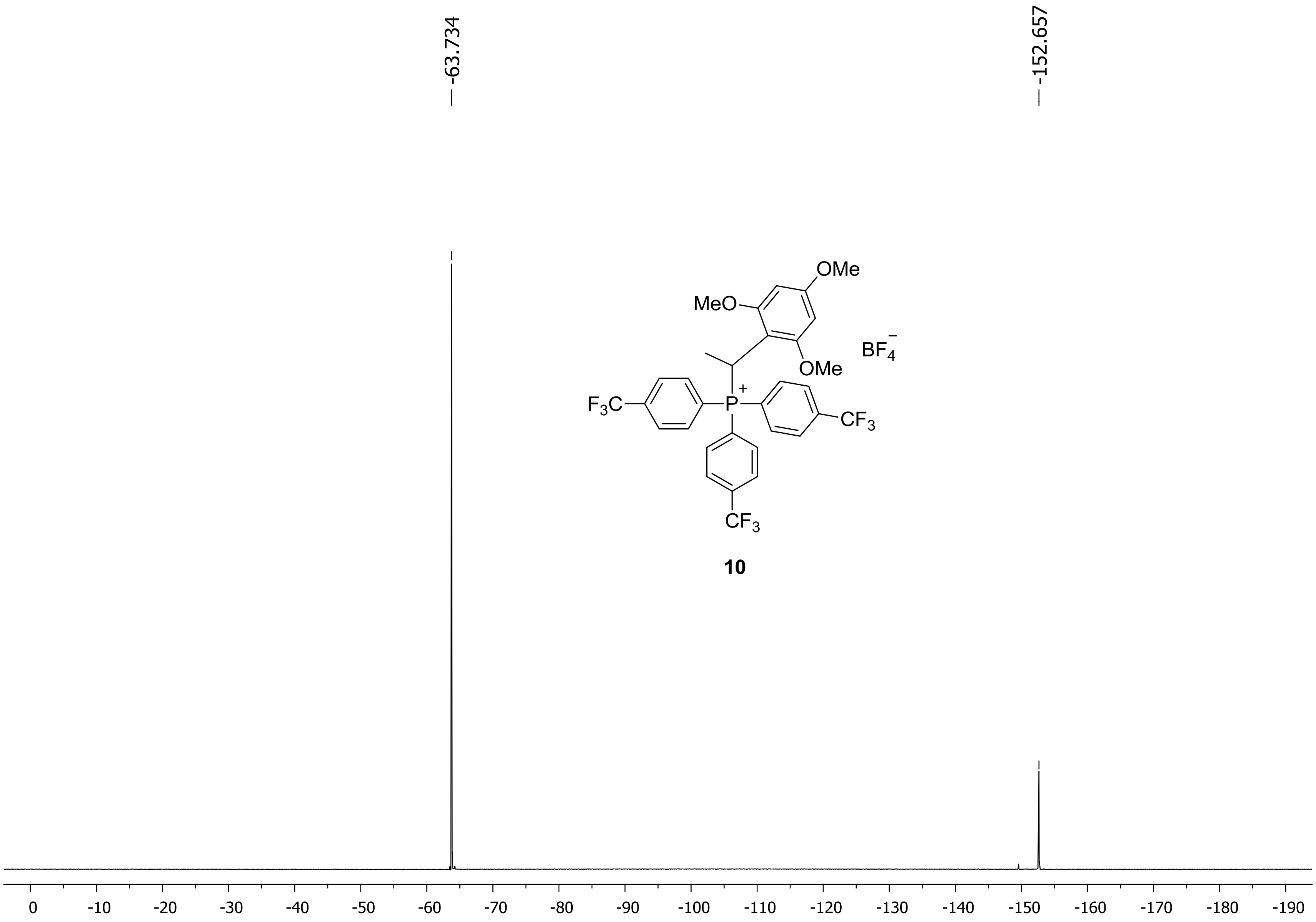

^{19}F NMR spectrum of 1-(2,4,6-trimethoxyphenyl)ethyltris(4-trifluoromethylphenyl)phosphonium tetrafluoroborate (**10**); 376 MHz/$CDCl_3$; δ (ppm).

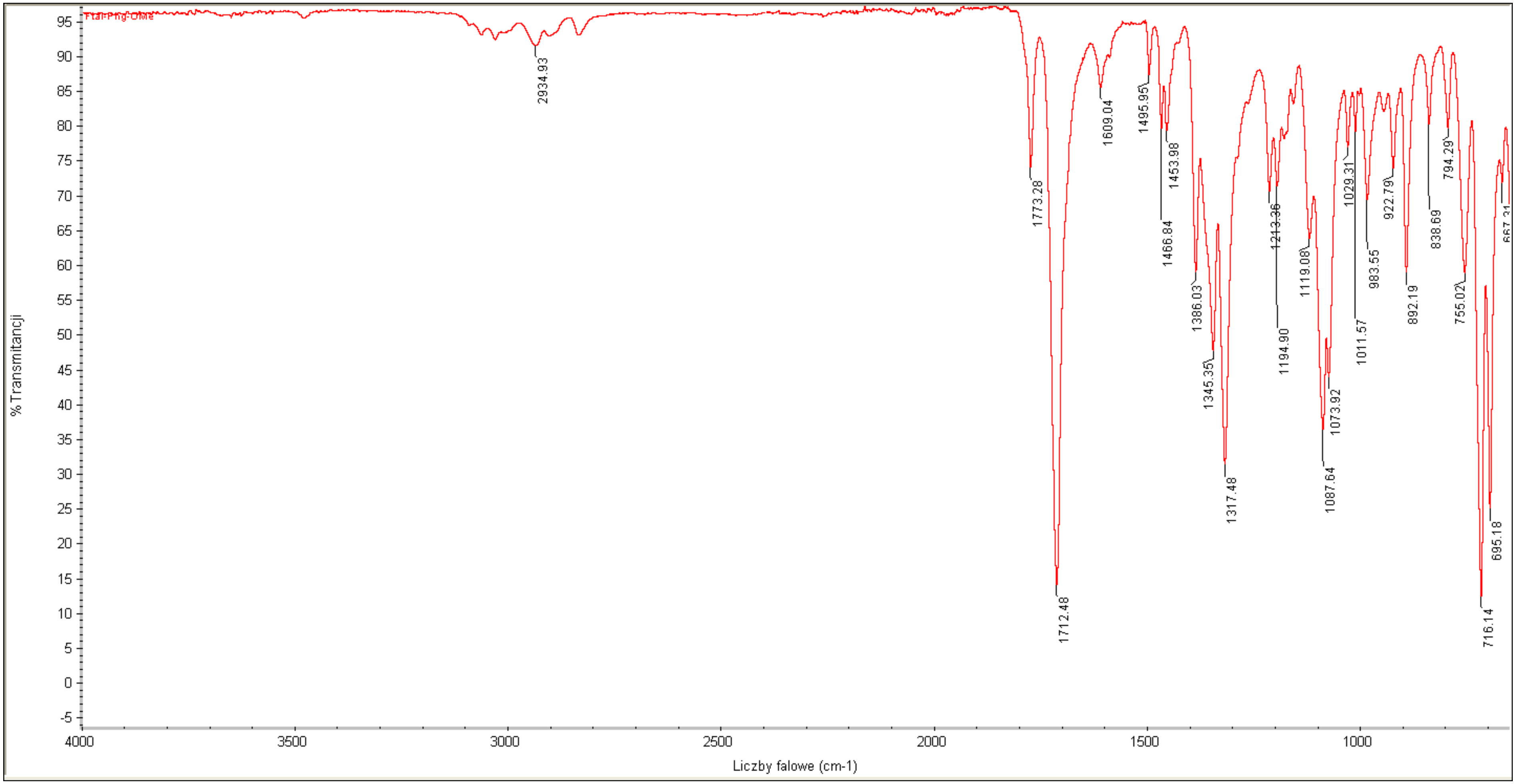

IR spectrum of *N*-(1-methoxy-1-phenylmethyl)phthalimide (**7c**); ATR, (cm^{-1}).

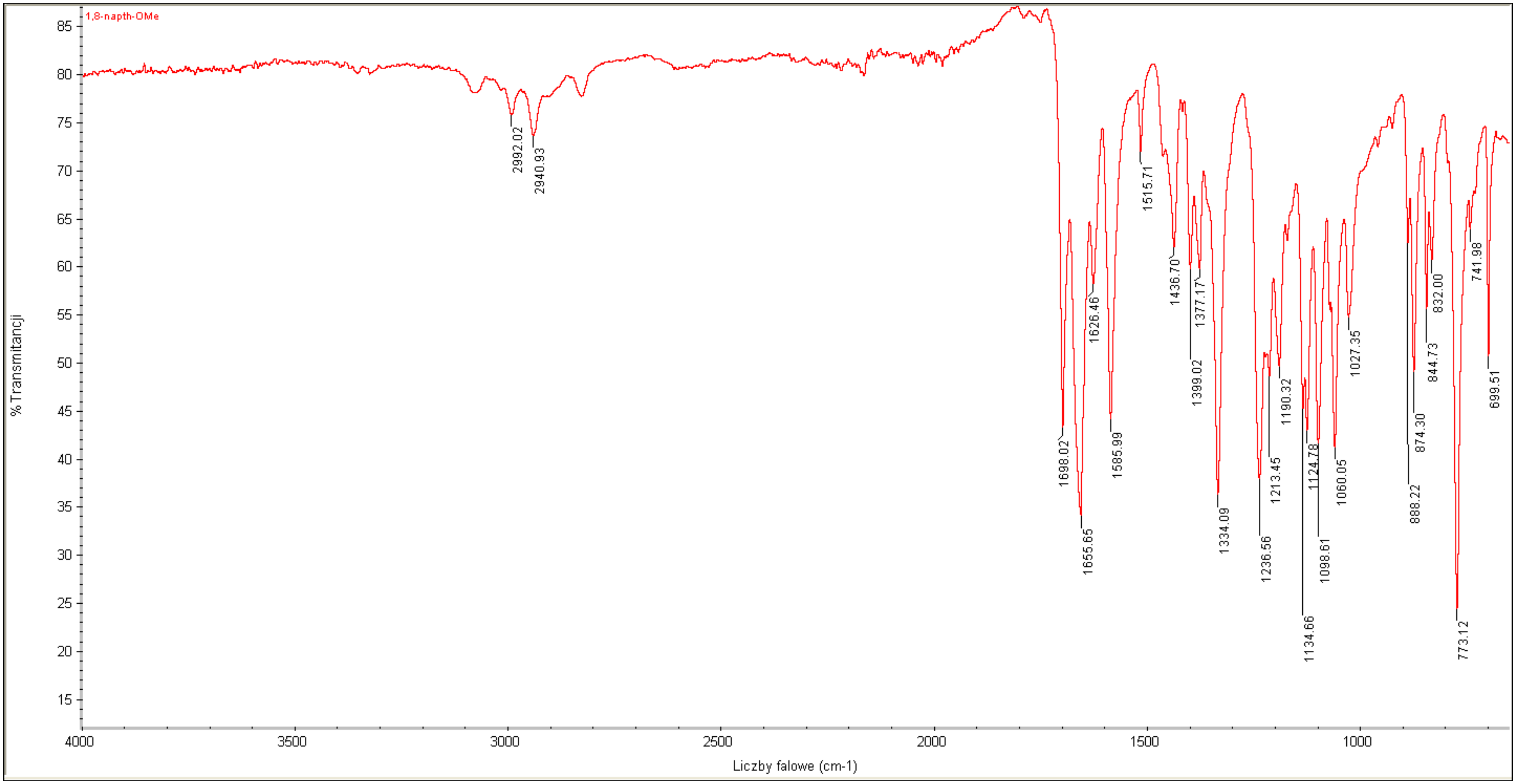

IR spectrum of *N*-(1-methoxyethyl)-1,8-naphthalimide (**7h**); ATR (cm^{-1}).

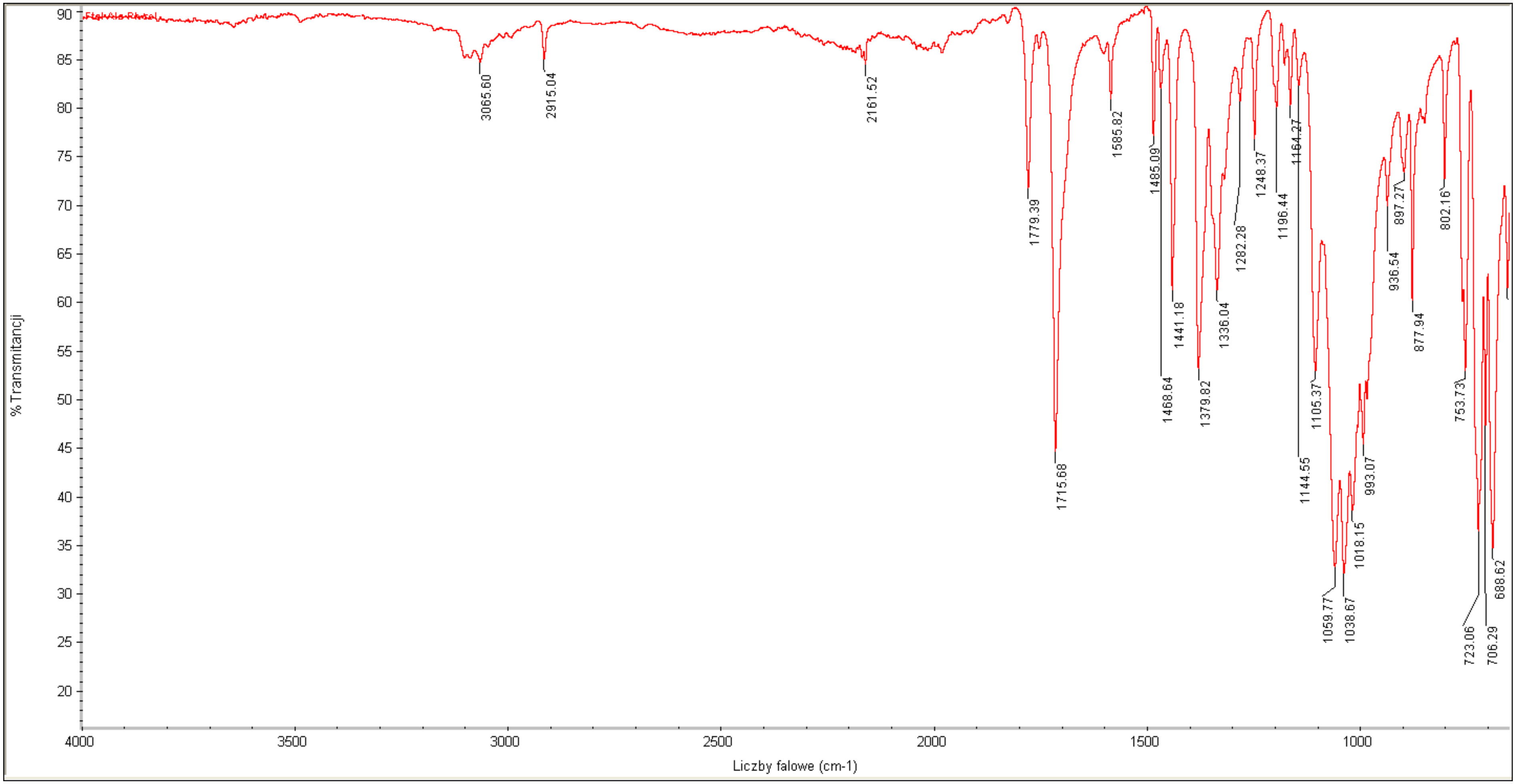

IR spectrum of 1-(*N*-phthalimido)ethyltriphenylphosphonium tetrafluoroborate (**5b**); ATR (cm^{-1}).

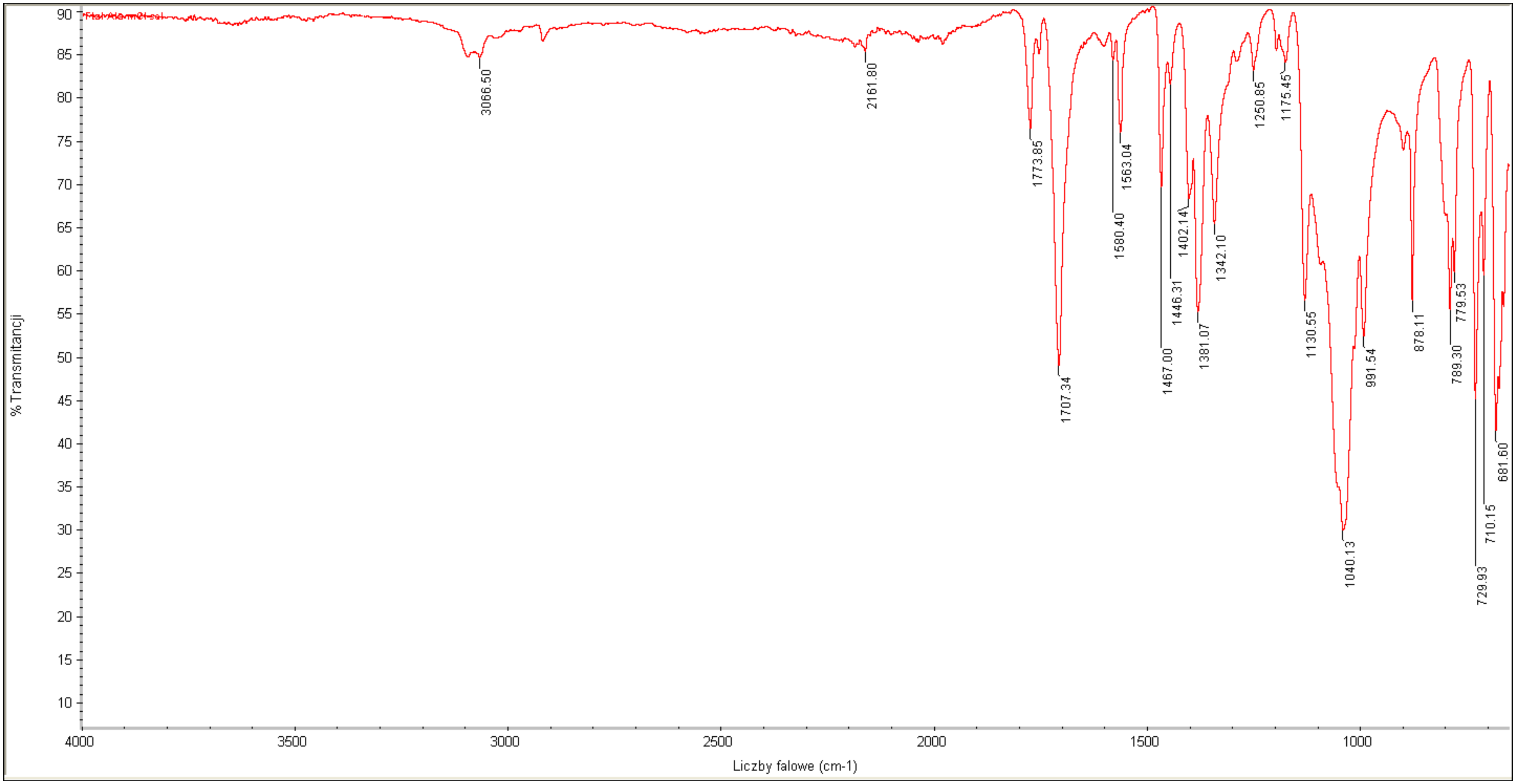

IR spectrum of 1-(*N*-phthalimido)ethyltris(3-chlorophenyl)phosphonium tetrafluoroborate (**5c**); ATR (cm^{-1}).

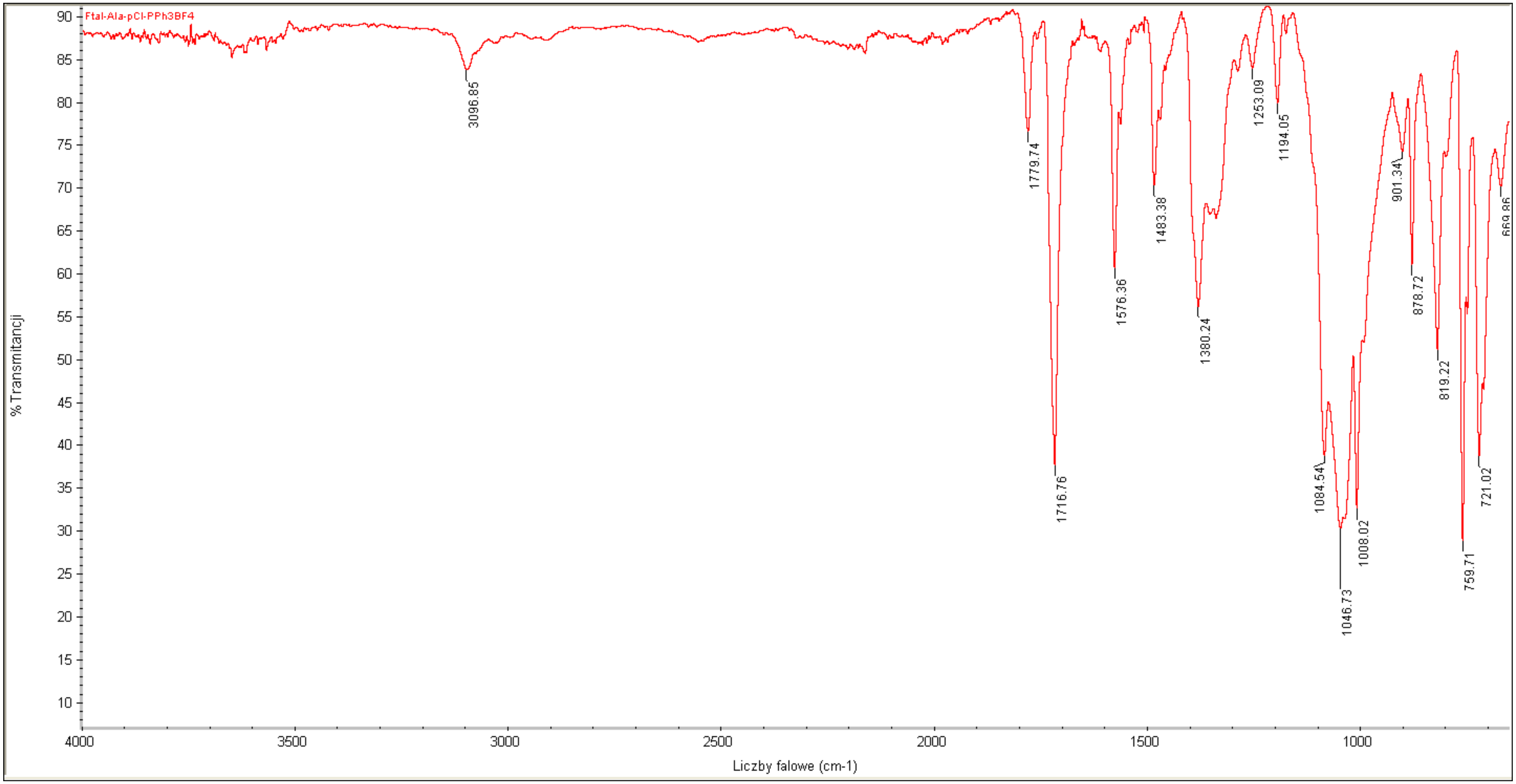

IR spectrum of 1-(*N*-phthalimido)ethyltris(4-chlorophenyl)phosphonium tetrafluoroborate (**5d**); ATR (cm^{-1}).

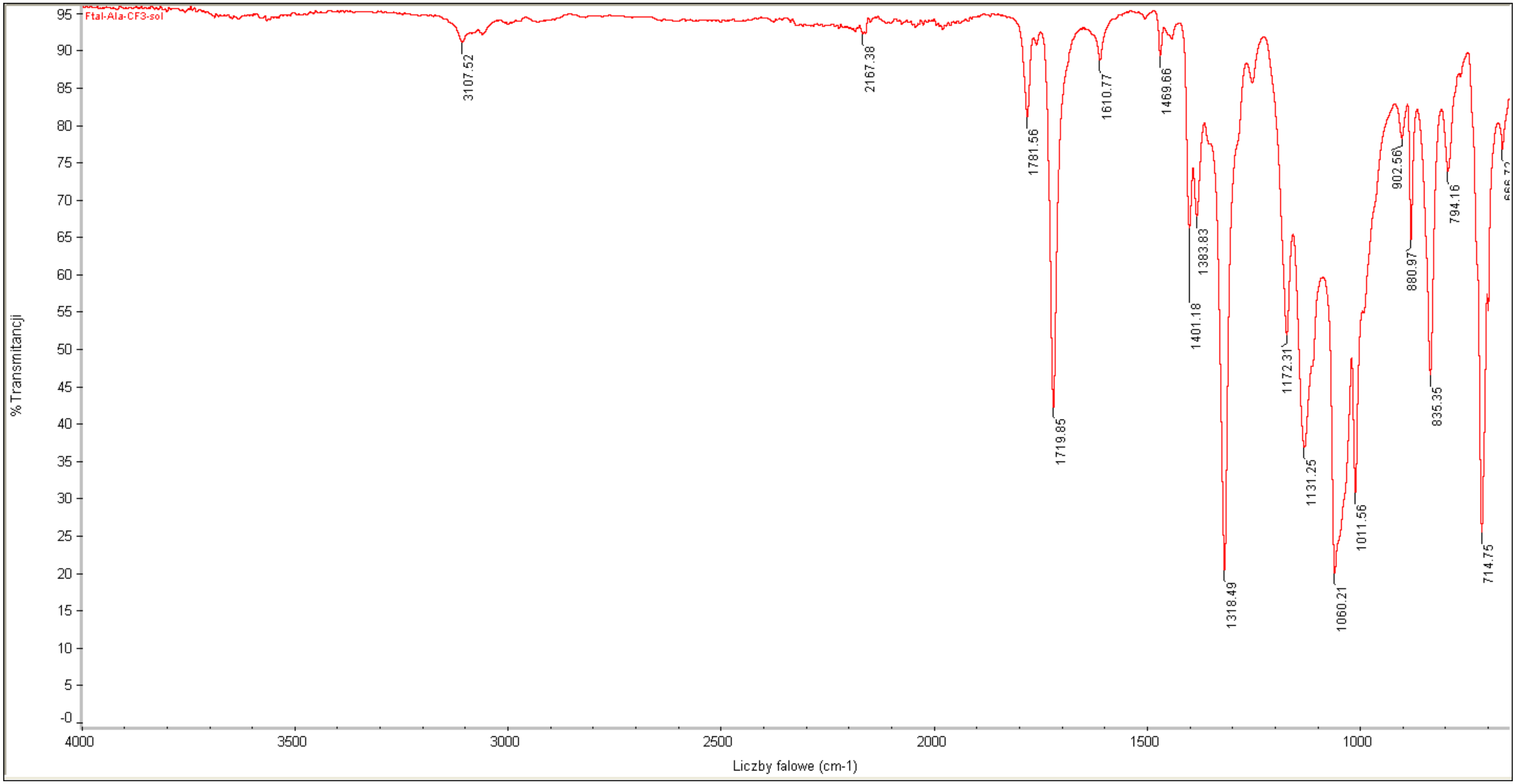

IR spectrum of 1-(*N*-phthalimido)ethyltris(4-trifluoromethylphenyl)phosphonium tetrafluoroborate (**5e**); ATR (cm^{-1}).

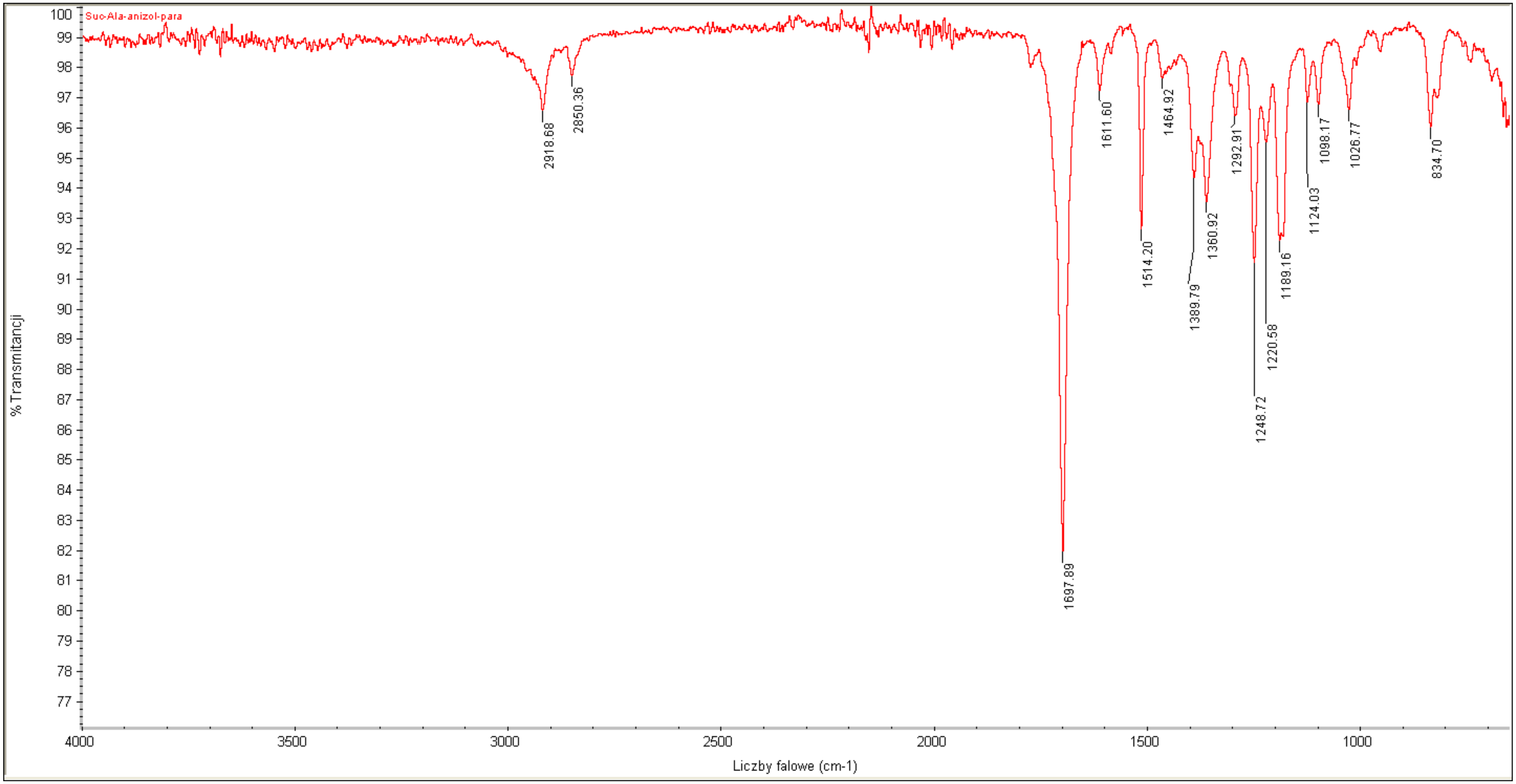

IR spectrum of *N*-[1-(4-methoxyphenyl)ethyl]succinimide (**9da**); ATR (cm^{-1}).

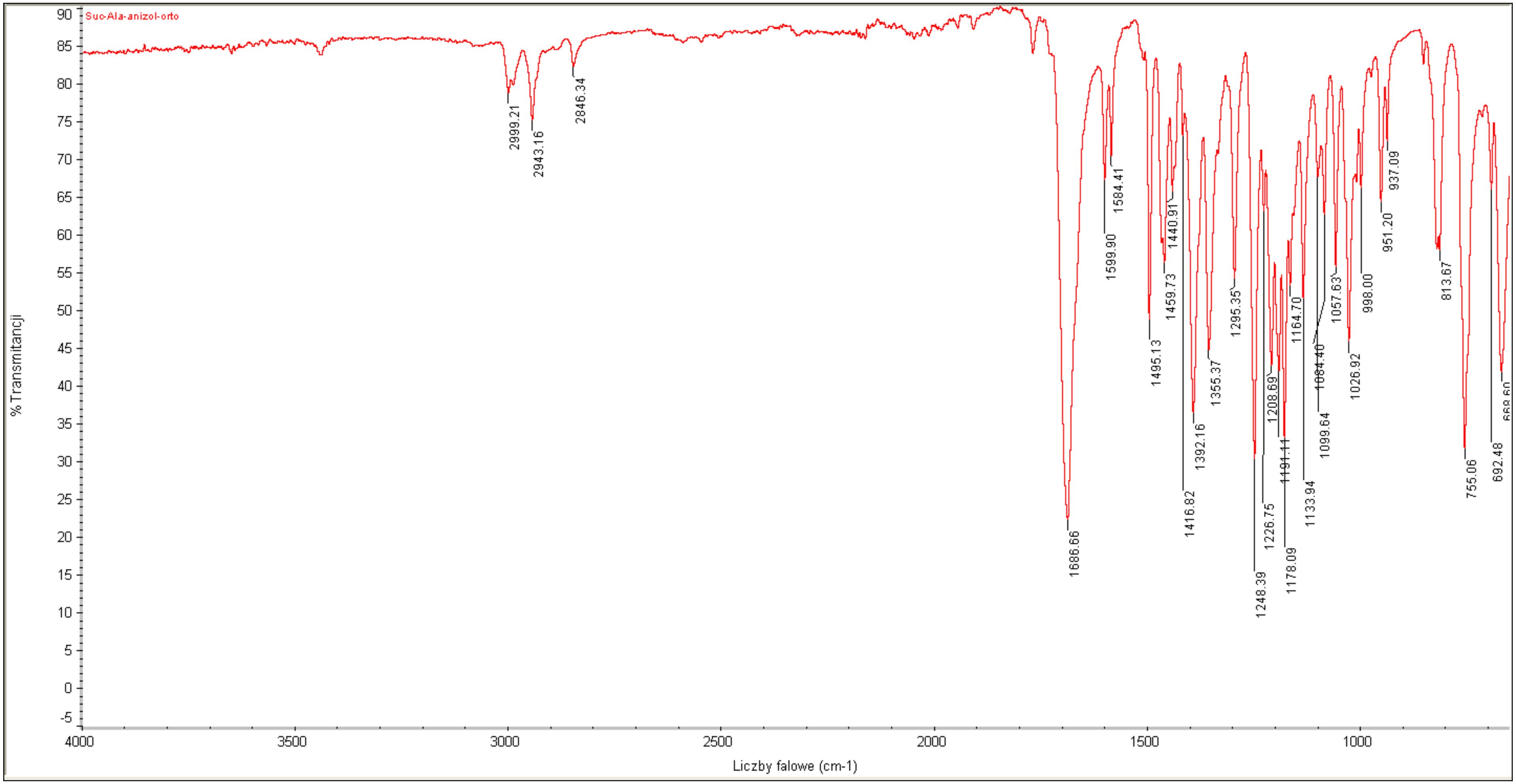

IR spectrum of *N*-[1-(2-methoxyphenyl)ethyl]succinimide (**9db**); ATR (cm^{-1}).

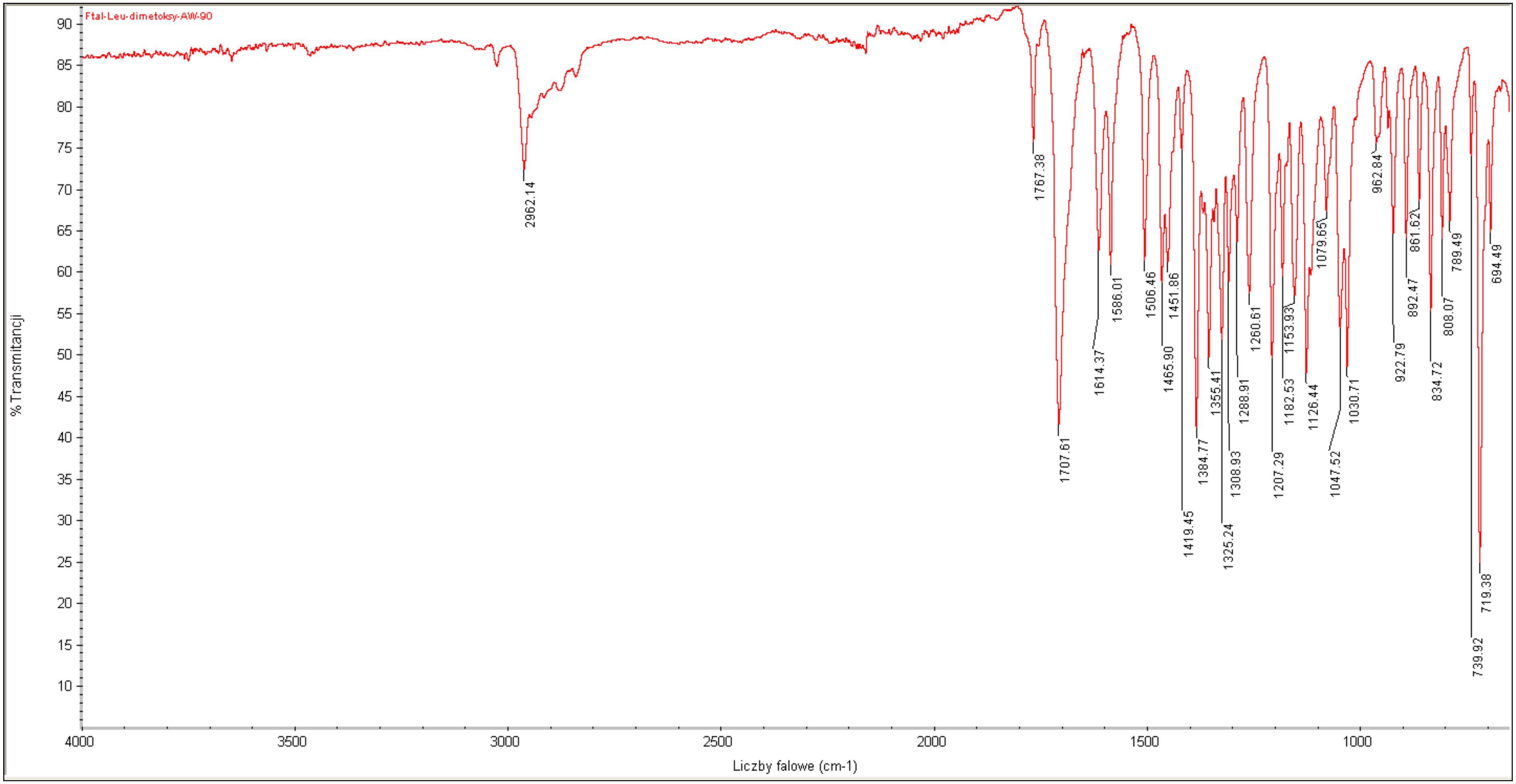

IR spectrum of *N*-[1-(2,4-dimethoxyphenyl)-3-methylbutyl]phthalimide (**9g**); ATR (cm^{-1}).

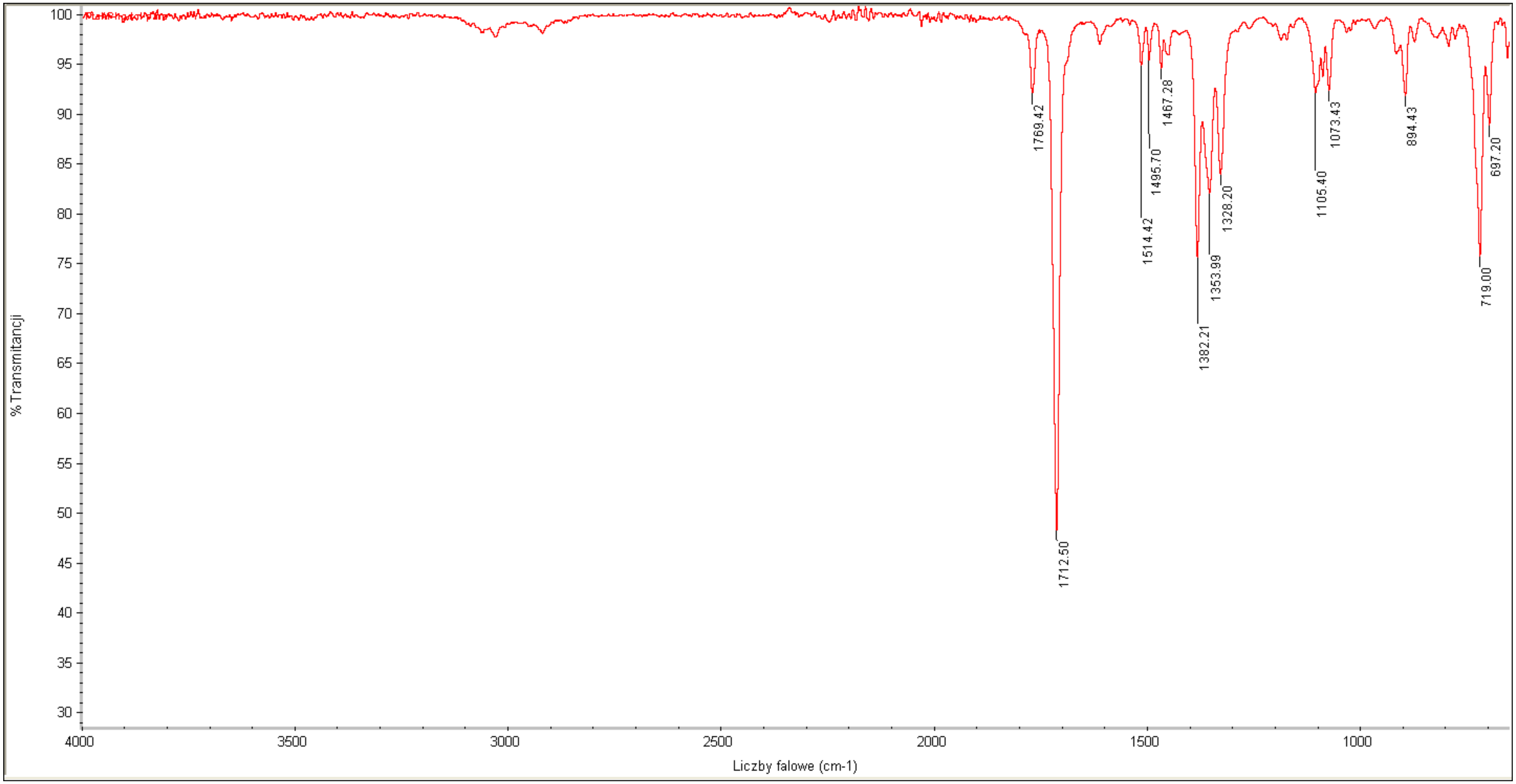

IR spectrum of *N*-[1-(4-methylphenyl)-1-phenylmethyl]phthalimide (**9ia**); ATR (cm^{-1}).

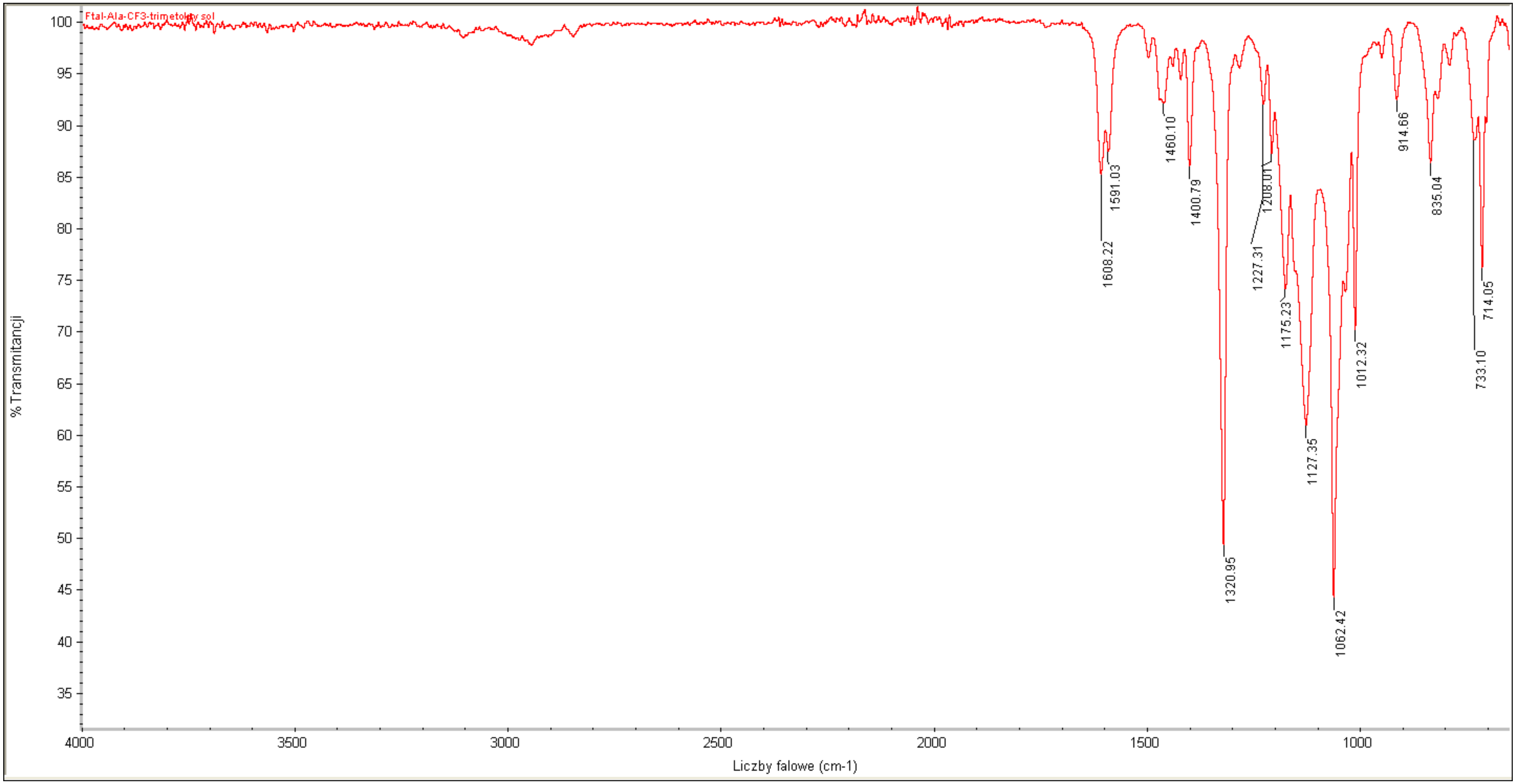

IR spectrum of 1-(2,4,6-trimethoxyphenyl)ethyltris(4-trifluoromethylphenyl)phosphonium tetrafluoroborate (**10**); ATR (cm^{-1}).
